# Supplementary material for: Challenges of COVID-19 Case Forecasting in the US, 2020–2021
Source: PLoS Comput Biol. 2024 May 6;20(5):e1011200. doi: 10.1371/journal.pcbi.1011200 (PMC11098513; doi:10.1371/journal.pcbi.1011200)

<u>Supporting Information 2</u>: Revision and outlier sensitivity analyses

S2 Fig A. To assess the influence of data revisions on our evaluation of forecast skill, we compared daily differences in cumulative reported cases during the week they were first reported to reported case counts for the same week in the complete data as of April 2, 2022. In total 721 weeks had at least one day with a revised case count (17% of all weeks, n=4,241 weeks) and revisions occurred in 43 of 51 jurisdictions. These jurisdiction-specific plots compare cases reported as of the date in the subtitle (in red) compared to cases reported as of April 2, 2022 (in black).

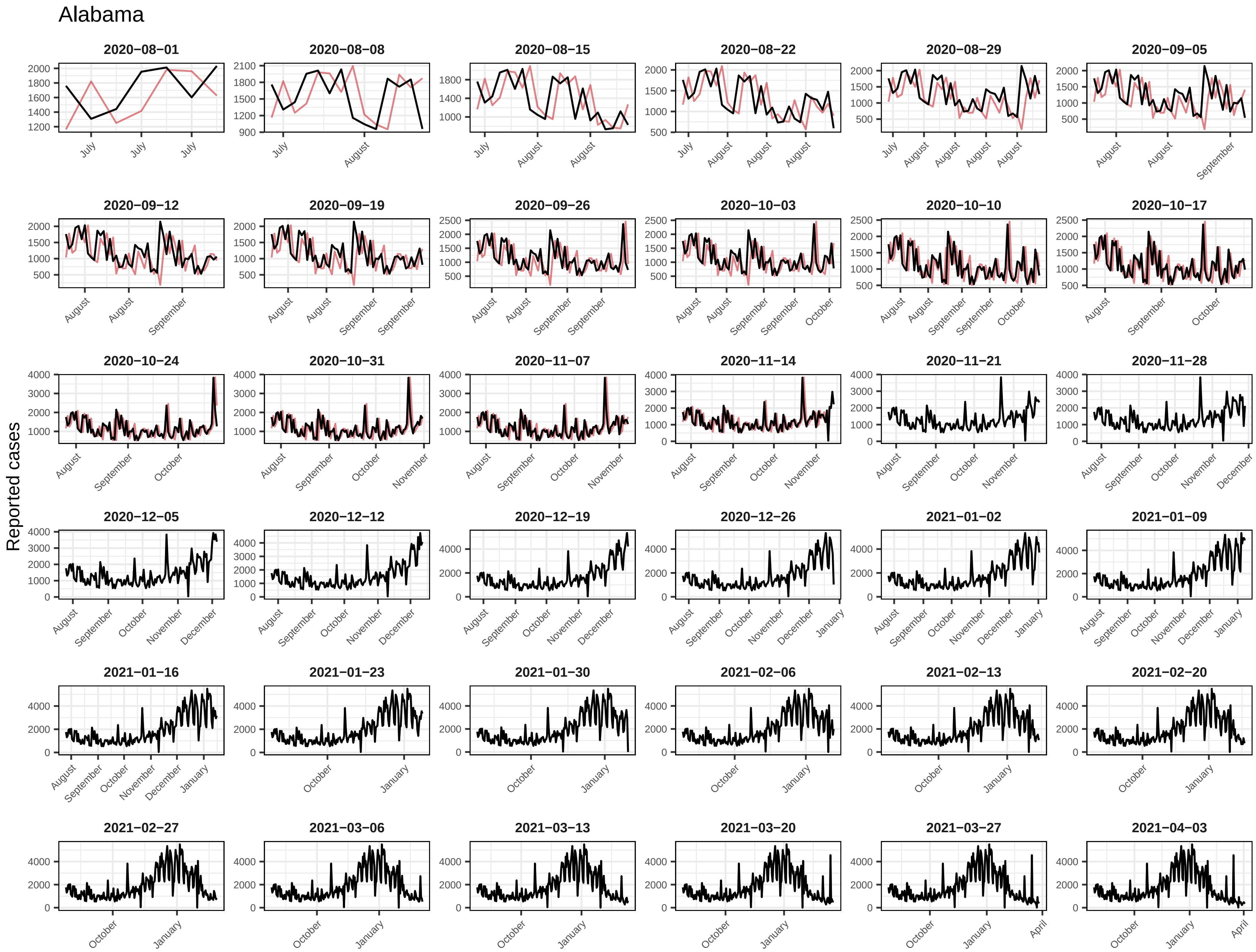

Alabama
Reported cases
2020−08−01
2020−08−08
2020−08−15
2020−08−22
2020−08−29
2020−09−05
2020−09−12
2020−09−19
2020−09−26
2020−10−03
2020−10−10
2020−10−17
2020−10−24
2020−10−31
2020−11−07
2020−11−14
2020−11−21
2020−11−28
2020−12−05
2020−12−12
2020−12−19
2020−12−26
2021−01−02
2021−01−09
2021−01−16
2021−01−23
2021−01−30
2021−02−06
2021−02−13
2021−02−20
2021−02−27
2021−03−06
2021−03−13
2021−03−20
2021−03−27
2021−04−03

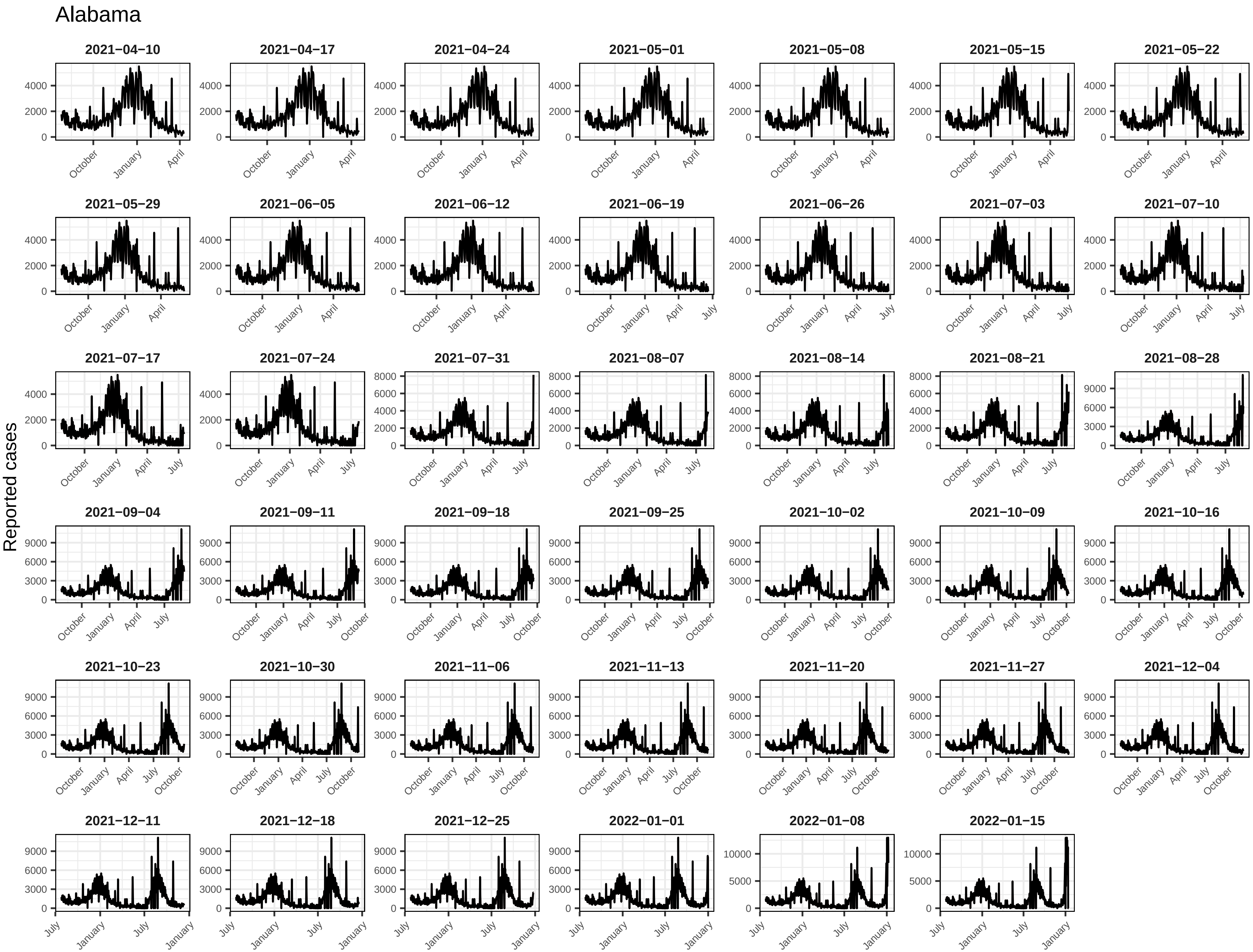

Alabama
Reported cases
2021−04−10
2021−04−17
2021−04−24
2021−05−01
2021−05−08
2021−05−15
2021−05−22
2021−05−29
2021−06−05
2021−06−12
2021−06−19
2021−06−26
2021−07−03
2021−07−10
2021−07−17
2021−07−24
2021−07−31
2021−08−07
2021−08−14
2021−08−21
2021−08−28
2021−09−04
2021−09−11
2021−09−18
2021−09−25
2021−10−02
2021−10−09
2021−10−16
2021−10−23
2021−10−30
2021−11−06
2021−11−13
2021−11−20
2021−11−27
2021−12−04
2021−12−11
2021−12−18
2021−12−25
2022−01−01
2022−01−08
2022−01−15
October
January
April
July

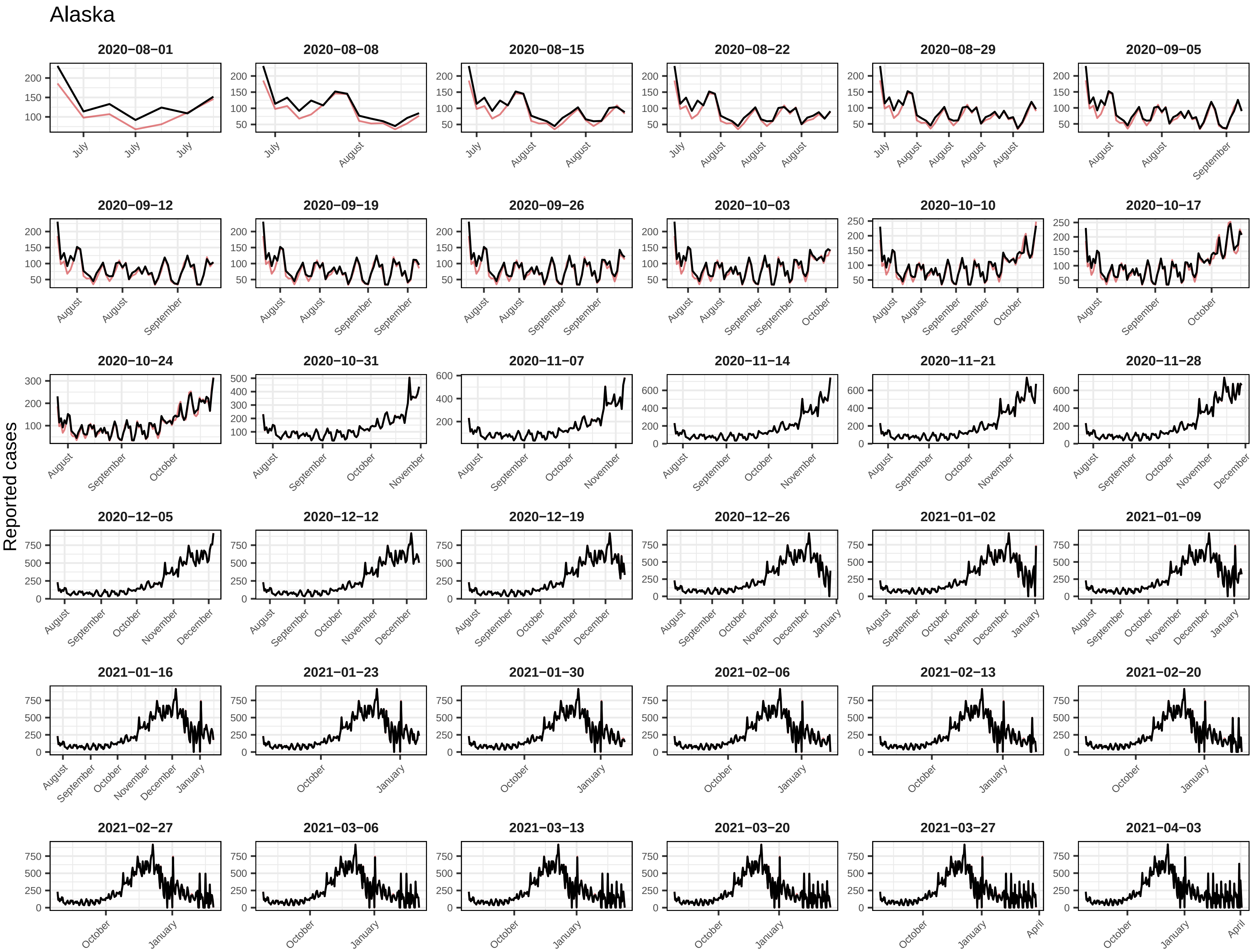
Alaska
Reported cases
2020−08−01
2020−08−08
2020−08−15
2020−08−22
2020−08−29
2020−09−05
2020−09−12
2020−09−19
2020−09−26
2020−10−03
2020−10−10
2020−10−17
2020−10−24
2020−10−31
2020−11−07
2020−11−14
2020−11−21
2020−11−28
2020−12−05
2020−12−12
2020−12−19
2020−12−26
2021−01−02
2021−01−09
2021−01−16
2021−01−23
2021−01−30
2021−02−06
2021−02−13
2021−02−20
2021−02−27
2021−03−06
2021−03−13
2021−03−20
2021−03−27
2021−04−03

Alaska

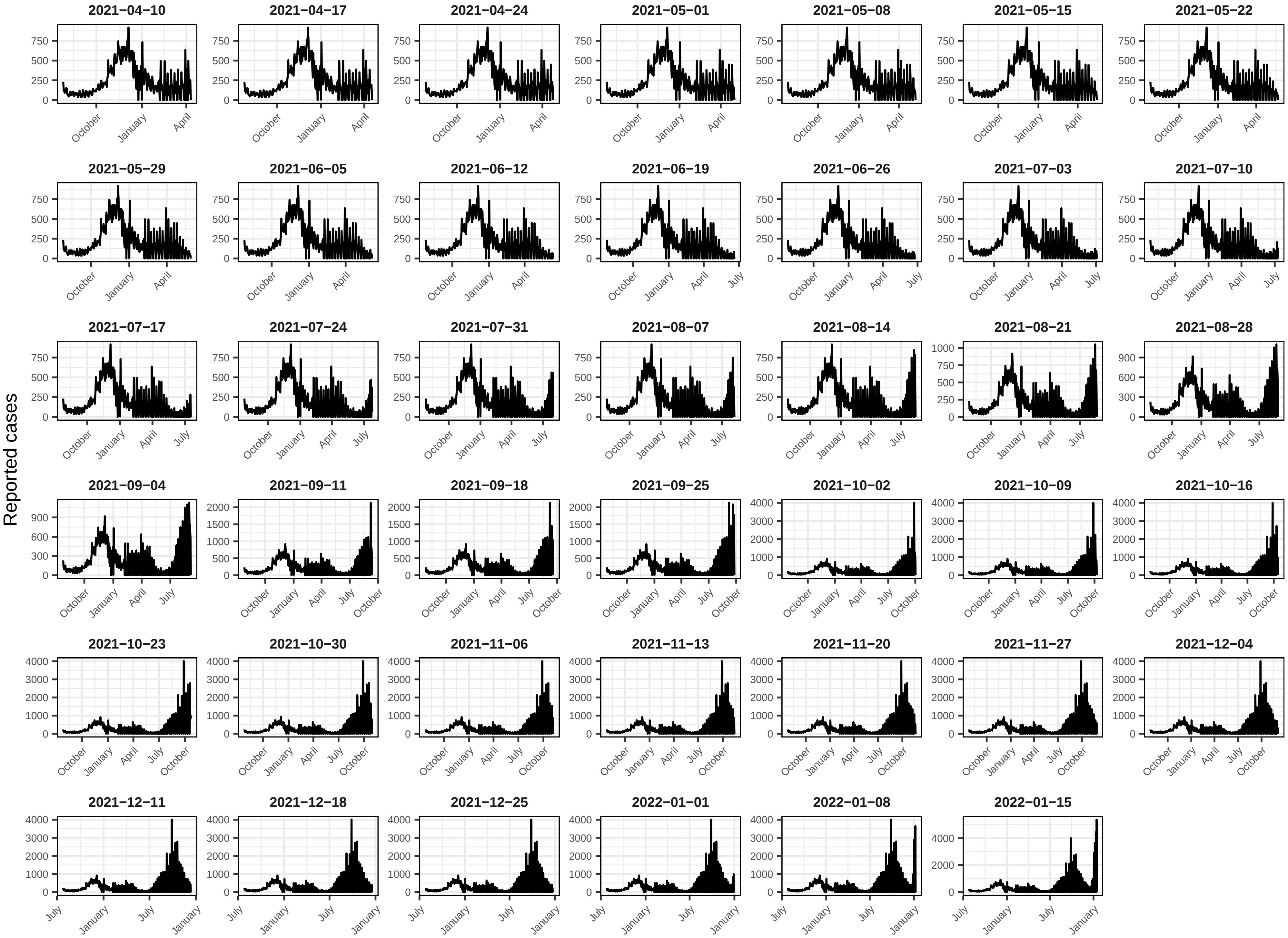
Reported cases
2021−04−10
2021−04−17
2021−04−24
2021−05−01
2021−05−08
2021−05−15
2021−05−22
2021−05−29
2021−06−05
2021−06−12
2021−06−19
2021−06−26
2021−07−03
2021−07−10
2021−07−17
2021−07−24
2021−07−31
2021−08−07
2021−08−14
2021−08−21
2021−08−28
2021−09−04
2021−09−11
2021−09−18
2021−09−25
2021−10−02
2021−10−09
2021−10−16
2021−10−23
2021−10−30
2021−11−06
2021−11−13
2021−11−20
2021−11−27
2021−12−04
2021−12−11
2021−12−18
2021−12−25
2022−01−01
2022−01−08
2022−01−15

Arizona

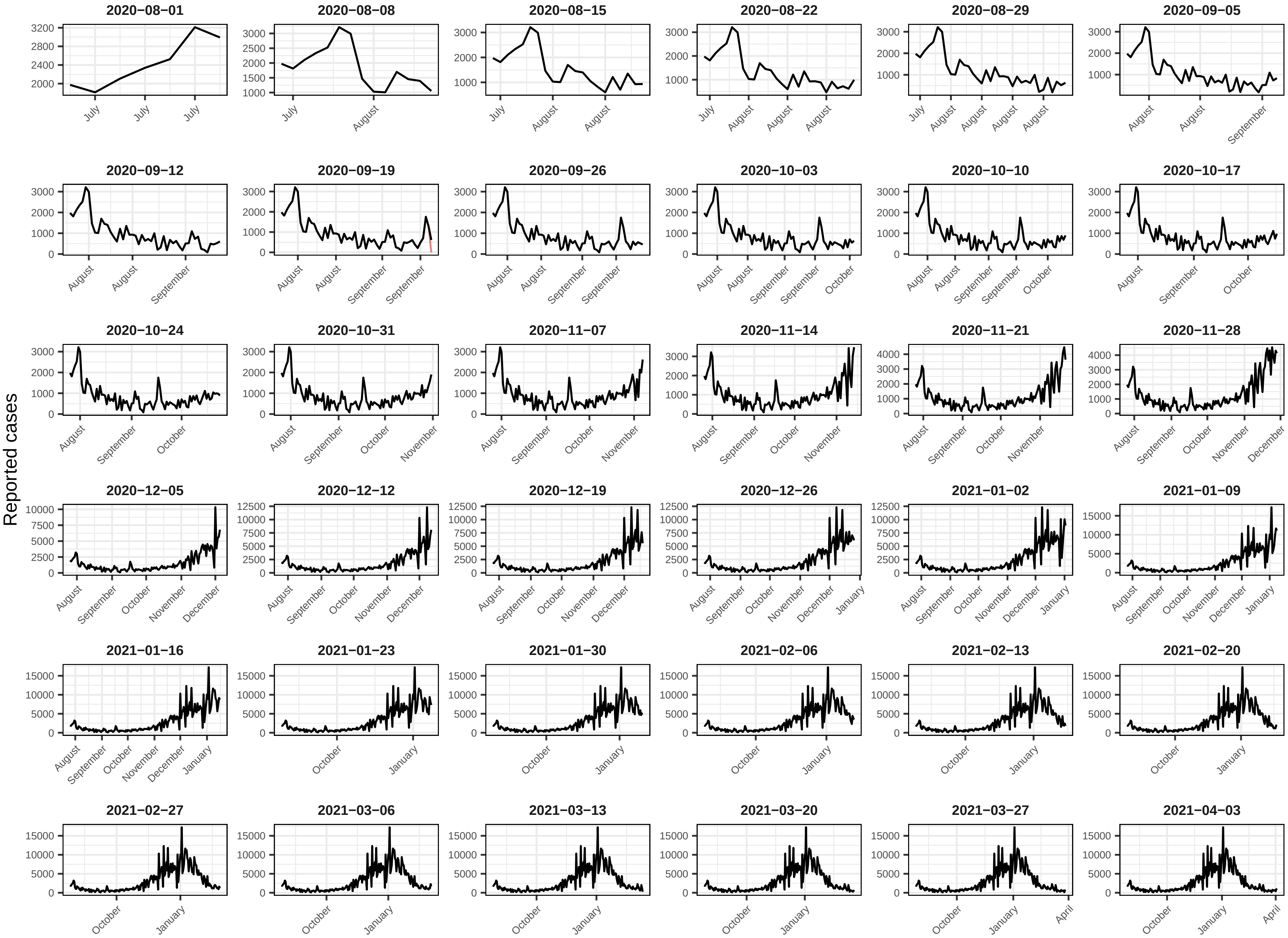

Reported cases
2020−08−01
2020−08−08
2020−08−15
2020−08−22
2020−08−29
2020−09−05
2020−09−12
2020−09−19
2020−09−26
2020−10−03
2020−10−10
2020−10−17
2020−10−24
2020−10−31
2020−11−07
2020−11−14
2020−11−21
2020−11−28
2020−12−05
2020−12−12
2020−12−19
2020−12−26
2021−01−02
2021−01−09
2021−01−16
2021−01−23
2021−01−30
2021−02−06
2021−02−13
2021−02−20
2021−02−27
2021−03−06
2021−03−13
2021−03−20
2021−03−27
2021−04−03

Arizona

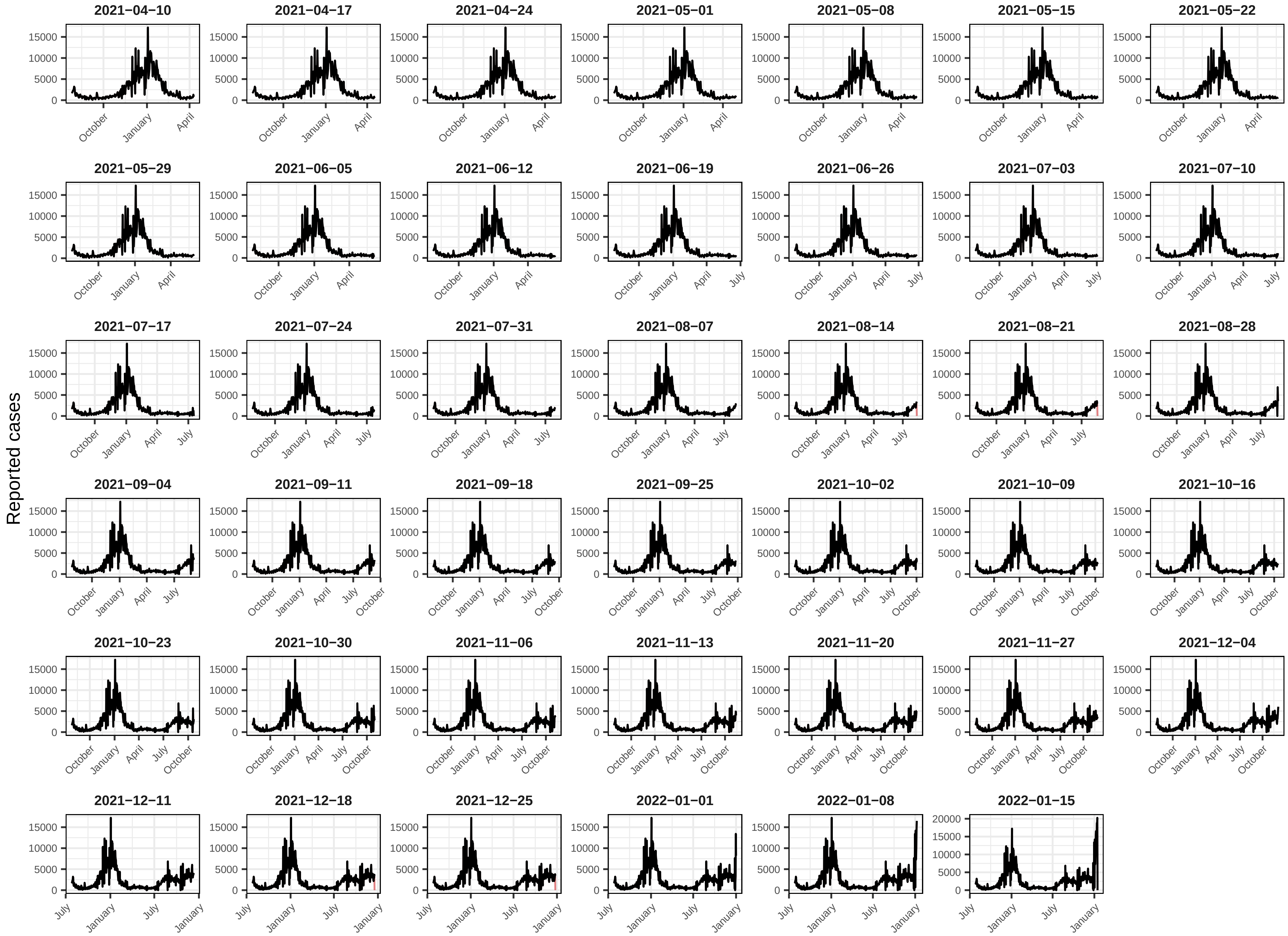
Reported cases
2021−04−10
2021−04−17
2021−04−24
2021−05−01
2021−05−08
2021−05−15
2021−05−22
2021−05−29
2021−06−05
2021−06−12
2021−06−19
2021−06−26
2021−07−03
2021−07−10
2021−07−17
2021−07−24
2021−07−31
2021−08−07
2021−08−14
2021−08−21
2021−08−28
2021−09−04
2021−09−11
2021−09−18
2021−09−25
2021−10−02
2021−10−09
2021−10−16
2021−10−23
2021−10−30
2021−11−06
2021−11−13
2021−11−20
2021−11−27
2021−12−04
2021−12−11
2021−12−18
2021−12−25
2022−01−01
2022−01−08
2022−01−15
0
5000
10000
15000
20000
July
October
January
April

Arkansas

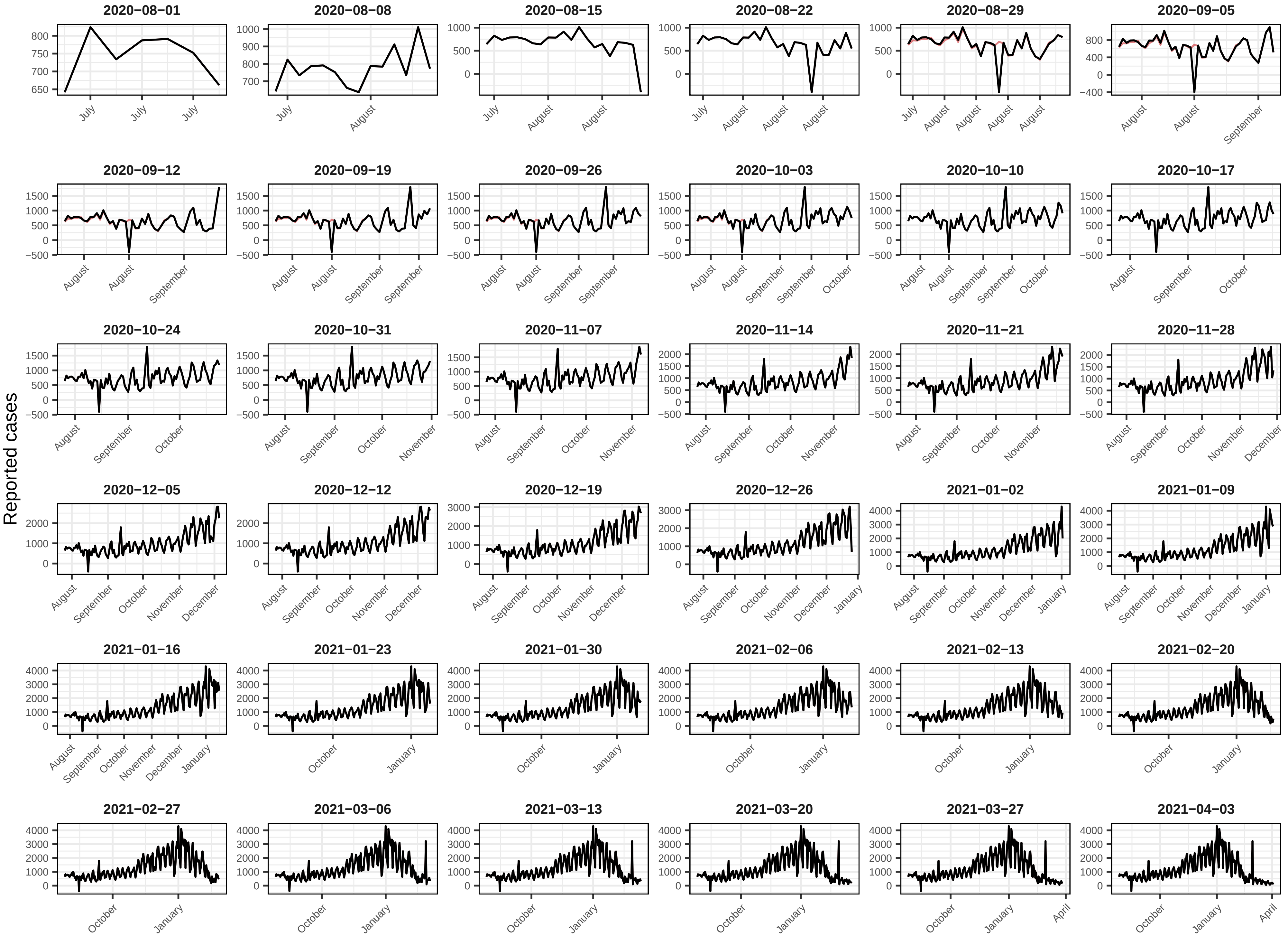
Reported cases
2020−08−01
2020−08−08
2020−08−15
2020−08−22
2020−08−29
2020−09−05
2020−09−12
2020−09−19
2020−09−26
2020−10−03
2020−10−10
2020−10−17
2020−10−24
2020−10−31
2020−11−07
2020−11−14
2020−11−21
2020−11−28
2020−12−05
2020−12−12
2020−12−19
2020−12−26
2021−01−02
2021−01−09
2021−01−16
2021−01−23
2021−01−30
2021−02−06
2021−02−13
2021−02−20
2021−02−27
2021−03−06
2021−03−13
2021−03−20
2021−03−27
2021−04−03

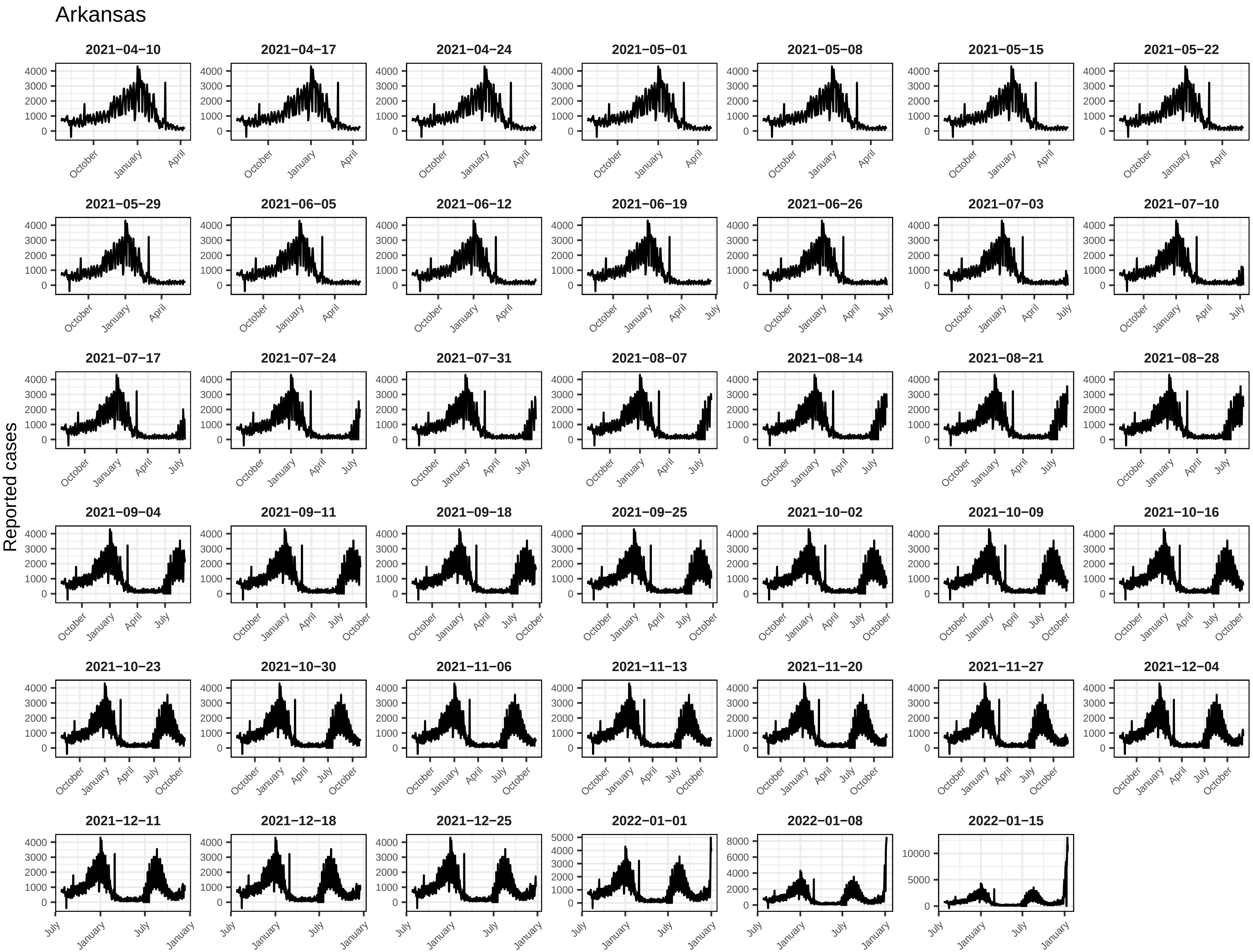
Arkansas
Reported cases
2021−04−10
2021−04−17
2021−04−24
2021−05−01
2021−05−08
2021−05−15
2021−05−22
2021−05−29
2021−06−05
2021−06−12
2021−06−19
2021−06−26
2021−07−03
2021−07−10
2021−07−17
2021−07−24
2021−07−31
2021−08−07
2021−08−14
2021−08−21
2021−08−28
2021−09−04
2021−09−11
2021−09−18
2021−09−25
2021−10−02
2021−10−09
2021−10−16
2021−10−23
2021−10−30
2021−11−06
2021−11−13
2021−11−20
2021−11−27
2021−12−04
2021−12−11
2021−12−18
2021−12−25
2022−01−01
2022−01−08
2022−01−15

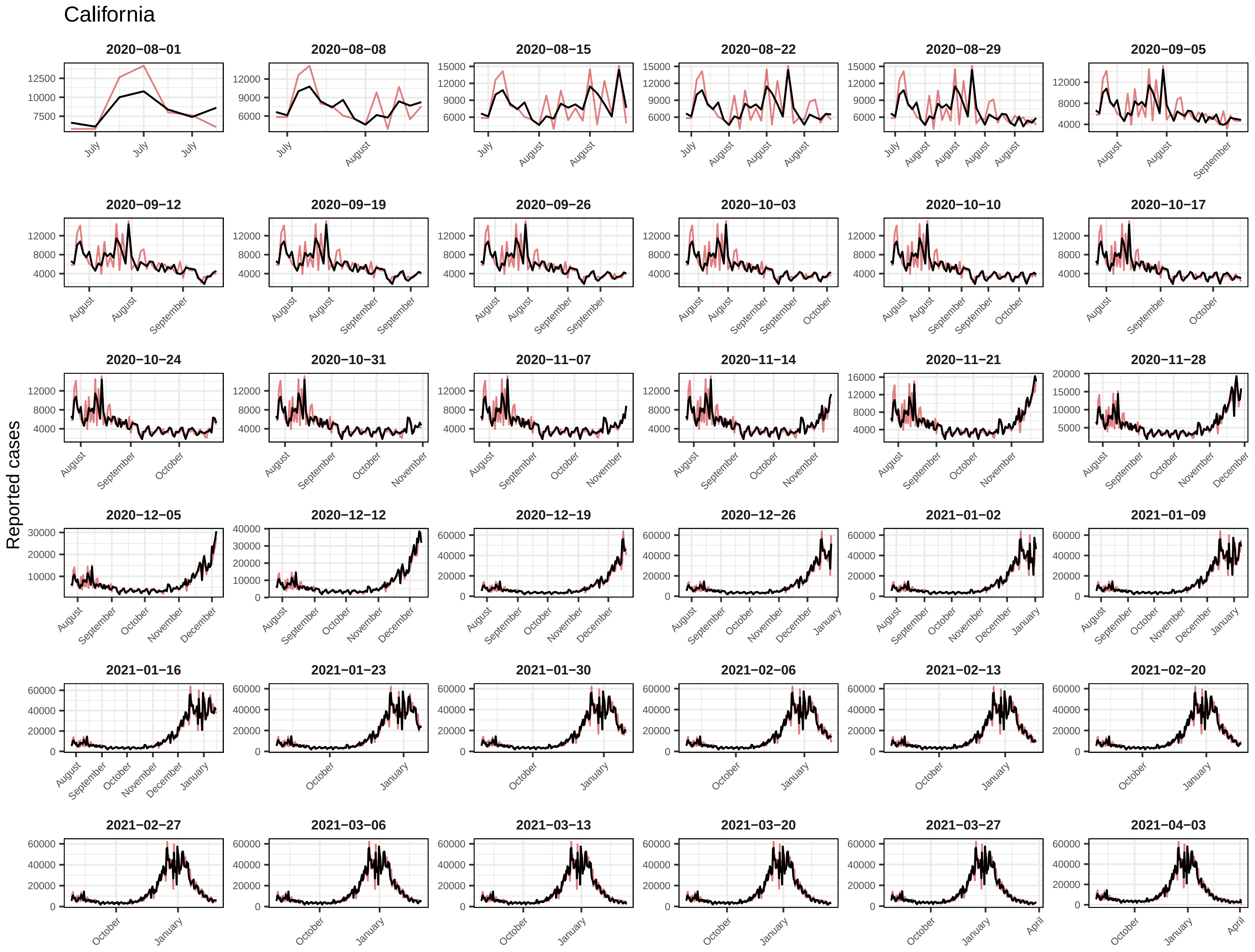
California
Reported cases
2020−08−01
2020−08−08
2020−08−15
2020−08−22
2020−08−29
2020−09−05
2020−09−12
2020−09−19
2020−09−26
2020−10−03
2020−10−10
2020−10−17
2020−10−24
2020−10−31
2020−11−07
2020−11−14
2020−11−21
2020−11−28
2020−12−05
2020−12−12
2020−12−19
2020−12−26
2021−01−02
2021−01−09
2021−01−16
2021−01−23
2021−01−30
2021−02−06
2021−02−13
2021−02−20
2021−02−27
2021−03−06
2021−03−13
2021−03−20
2021−03−27
2021−04−03

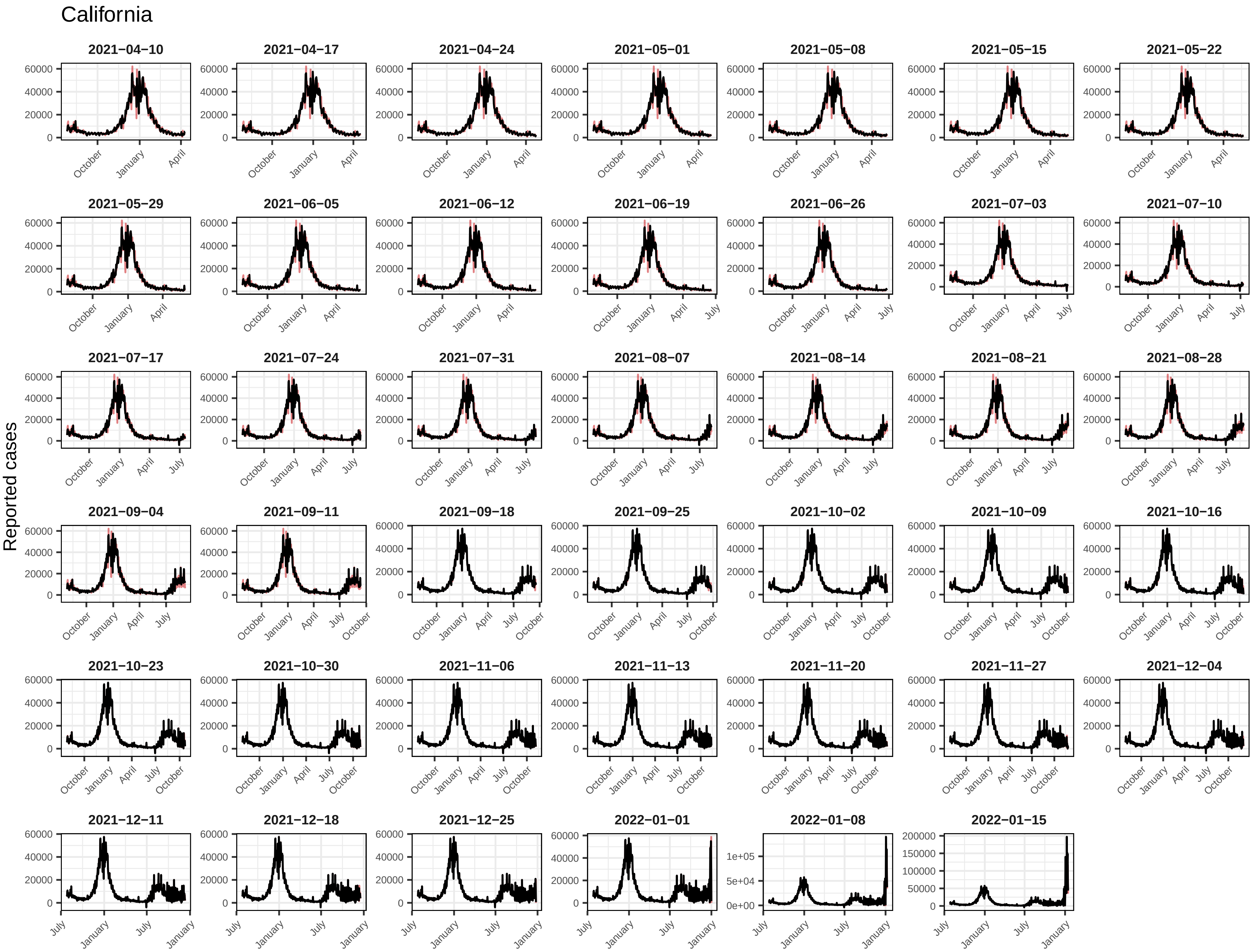
California
Reported cases
2021−04−10
2021−04−17
2021−04−24
2021−05−01
2021−05−08
2021−05−15
2021−05−22
2021−05−29
2021−06−05
2021−06−12
2021−06−19
2021−06−26
2021−07−03
2021−07−10
2021−07−17
2021−07−24
2021−07−31
2021−08−07
2021−08−14
2021−08−21
2021−08−28
2021−09−04
2021−09−11
2021−09−18
2021−09−25
2021−10−02
2021−10−09
2021−10−16
2021−10−23
2021−10−30
2021−11−06
2021−11−13
2021−11−20
2021−11−27
2021−12−04
2021−12−11
2021−12−18
2021−12−25
2022−01−01
2022−01−08
2022−01−15

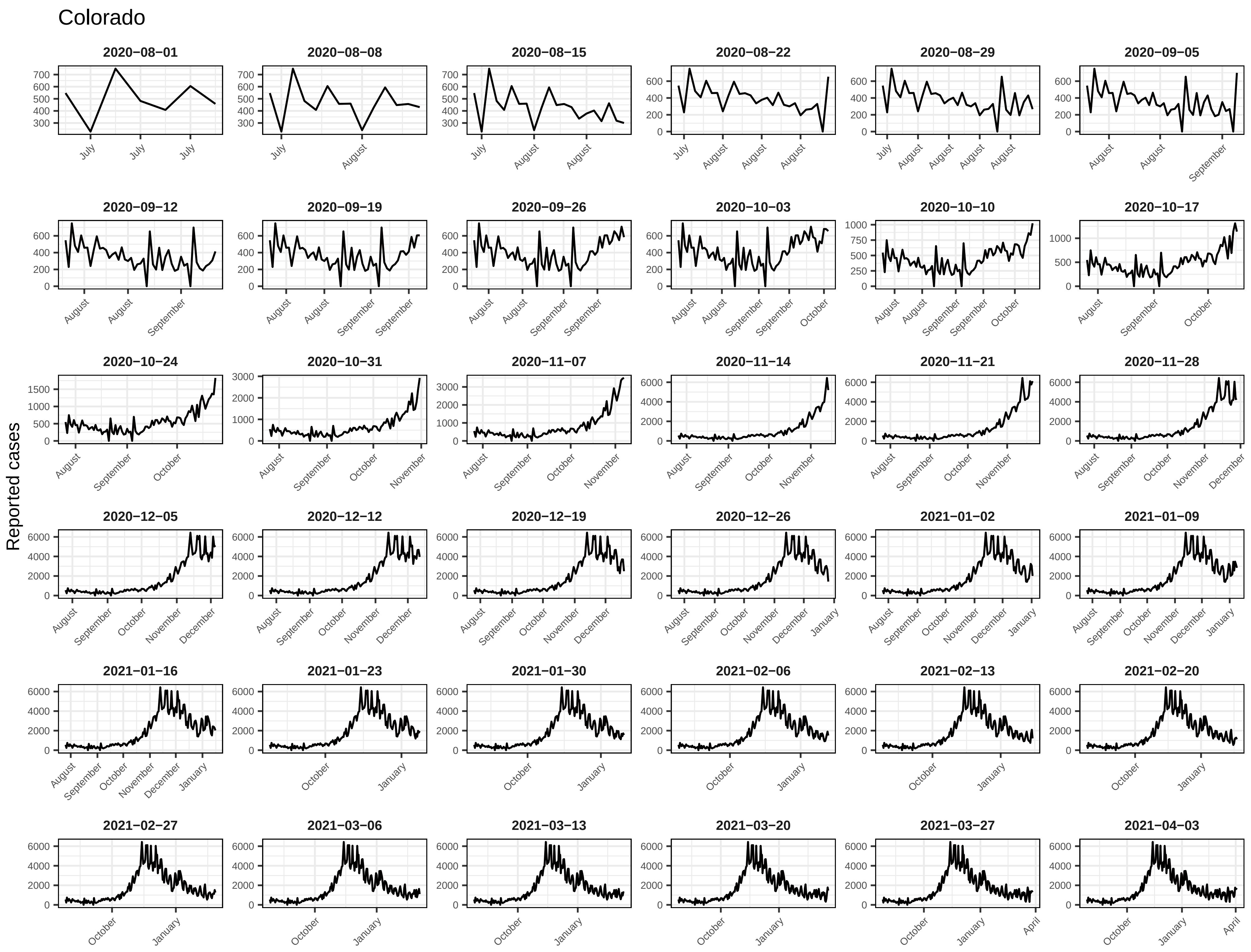
Colorado
Reported cases
2020−08−01
2020−08−08
2020−08−15
2020−08−22
2020−08−29
2020−09−05
2020−09−12
2020−09−19
2020−09−26
2020−10−03
2020−10−10
2020−10−17
2020−10−24
2020−10−31
2020−11−07
2020−11−14
2020−11−21
2020−11−28
2020−12−05
2020−12−12
2020−12−19
2020−12−26
2021−01−02
2021−01−09
2021−01−16
2021−01−23
2021−01−30
2021−02−06
2021−02−13
2021−02−20
2021−02−27
2021−03−06
2021−03−13
2021−03−20
2021−03−27
2021−04−03

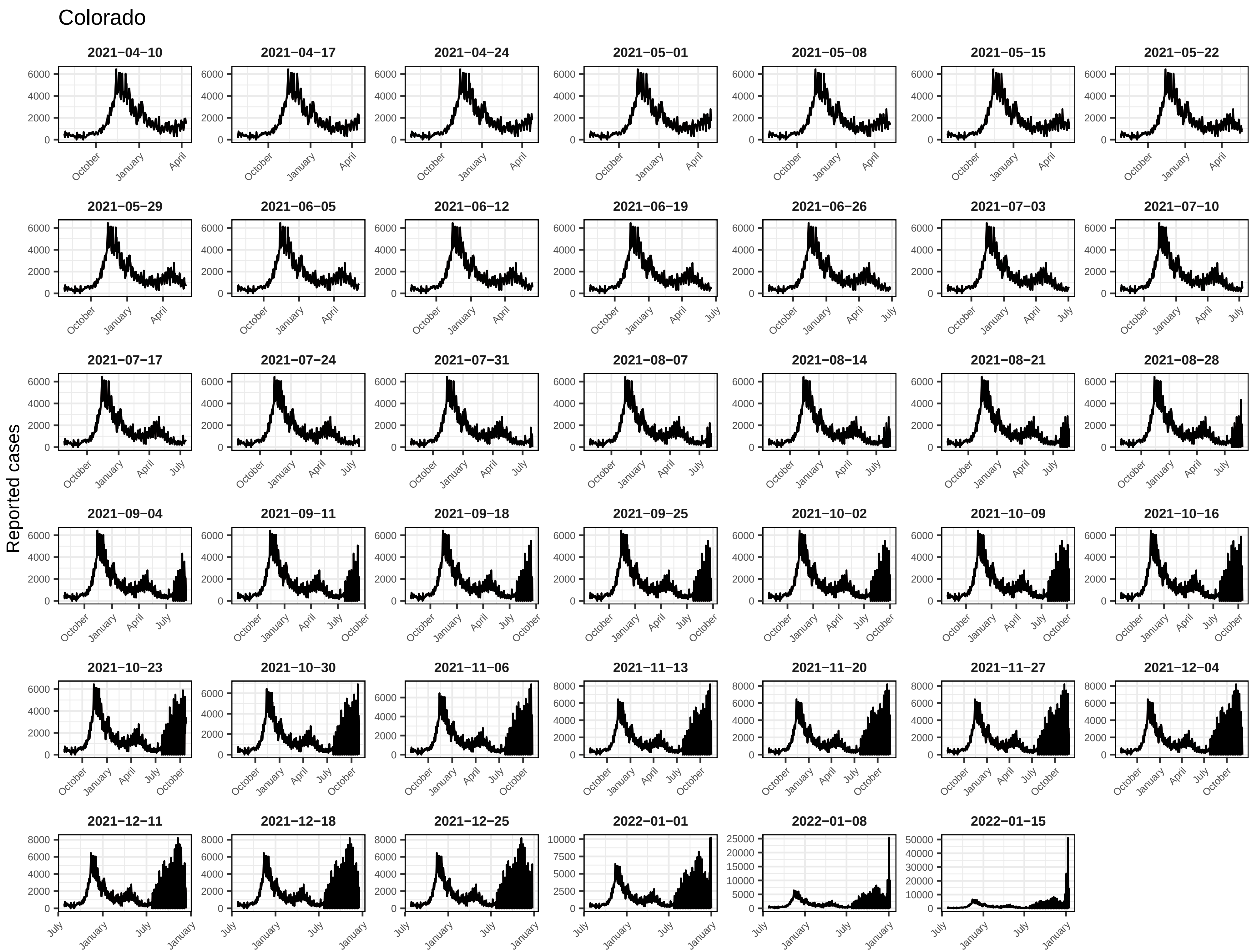
Colorado
Reported cases
2021−04−10
2021−04−17
2021−04−24
2021−05−01
2021−05−08
2021−05−15
2021−05−22
2021−05−29
2021−06−05
2021−06−12
2021−06−19
2021−06−26
2021−07−03
2021−07−10
2021−07−17
2021−07−24
2021−07−31
2021−08−07
2021−08−14
2021−08−21
2021−08−28
2021−09−04
2021−09−11
2021−09−18
2021−09−25
2021−10−02
2021−10−09
2021−10−16
2021−10−23
2021−10−30
2021−11−06
2021−11−13
2021−11−20
2021−11−27
2021−12−04
2021−12−11
2021−12−18
2021−12−25
2022−01−01
2022−01−08
2022−01−15

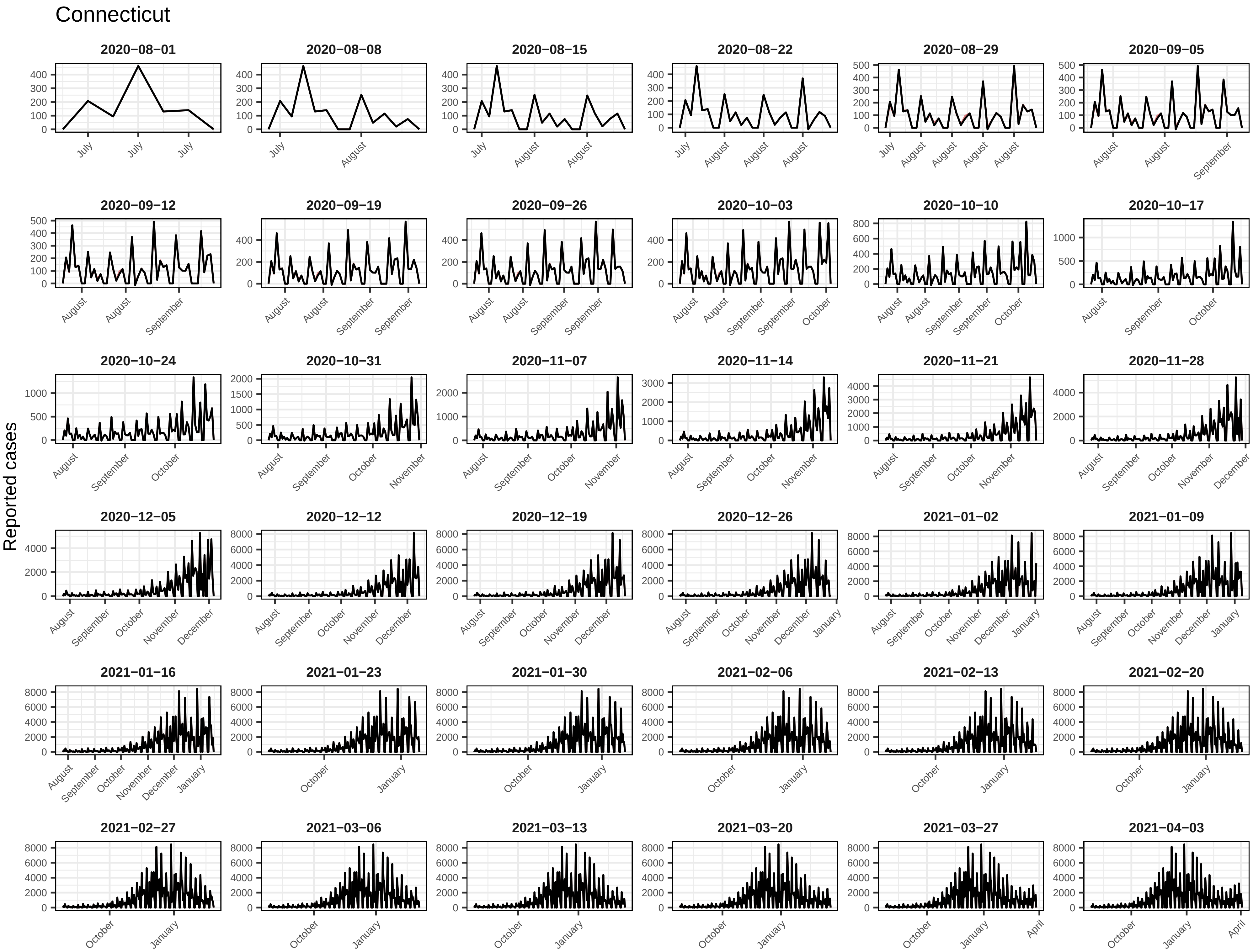
Connecticut
Reported cases
2020−08−01
2020−08−08
2020−08−15
2020−08−22
2020−08−29
2020−09−05
2020−09−12
2020−09−19
2020−09−26
2020−10−03
2020−10−10
2020−10−17
2020−10−24
2020−10−31
2020−11−07
2020−11−14
2020−11−21
2020−11−28
2020−12−05
2020−12−12
2020−12−19
2020−12−26
2021−01−02
2021−01−09
2021−01−16
2021−01−23
2021−01−30
2021−02−06
2021−02−13
2021−02−20
2021−02−27
2021−03−06
2021−03−13
2021−03−20
2021−03−27
2021−04−03

Connecticut

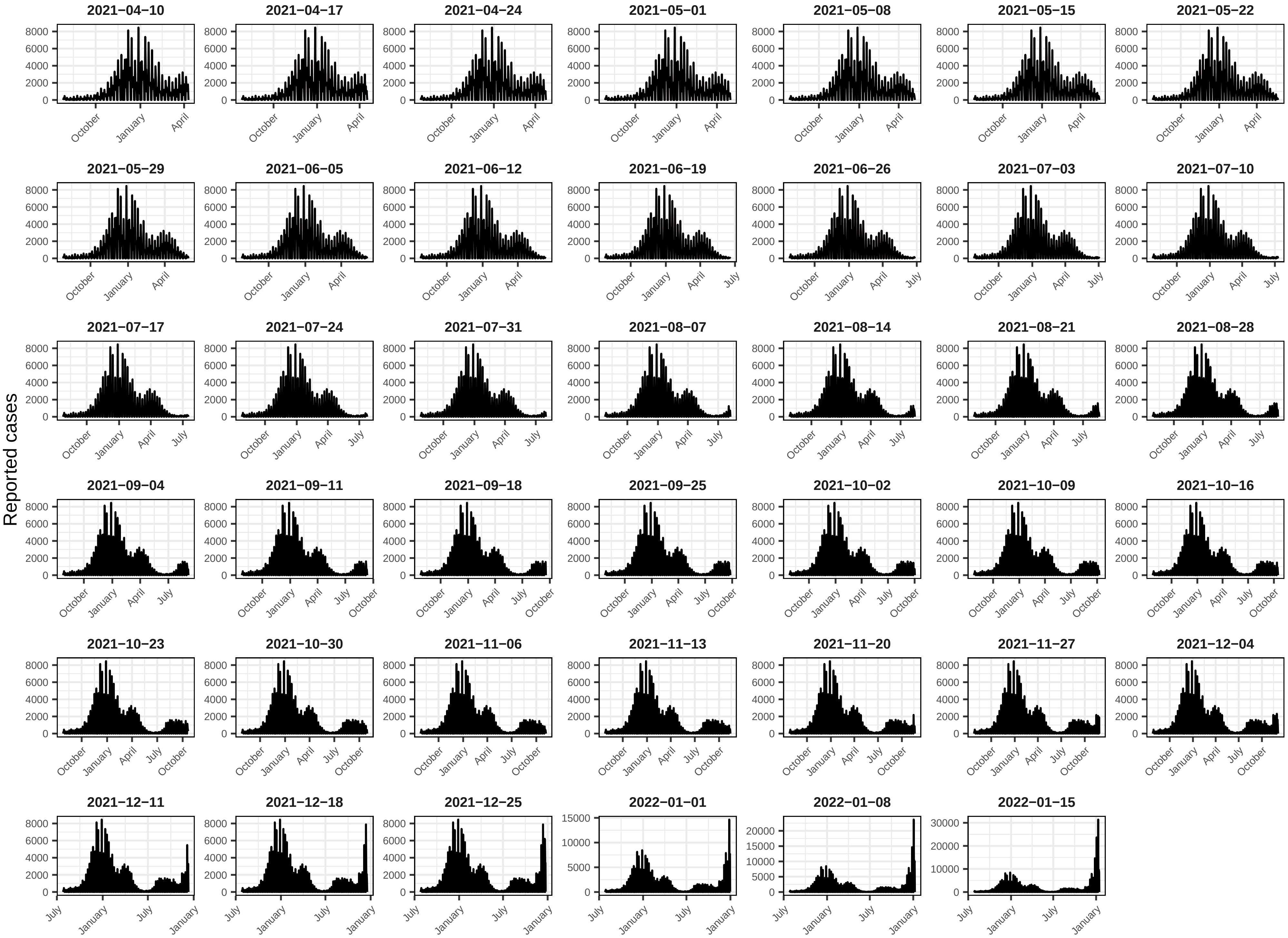

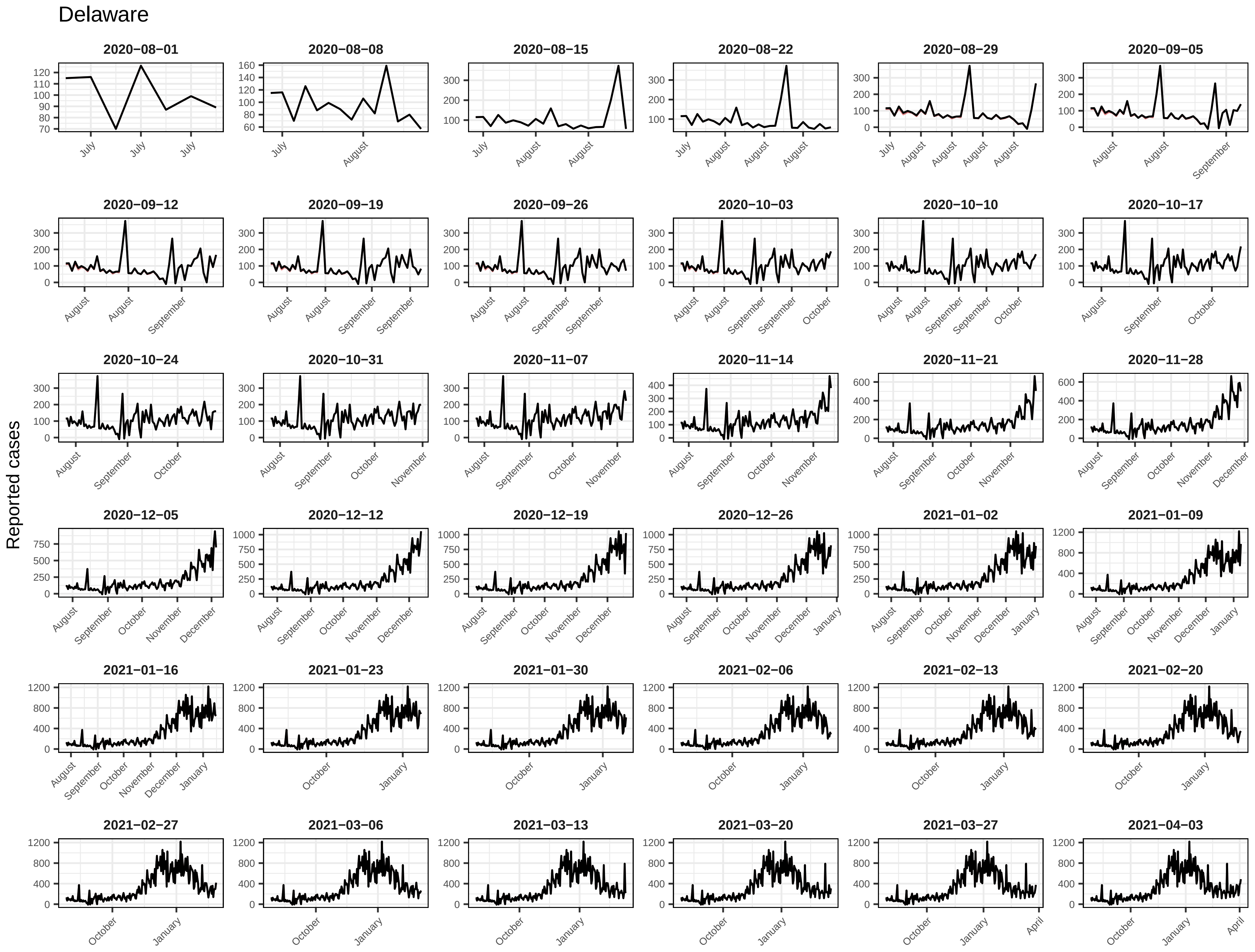

Delaware
Reported cases
2020−08−01
2020−08−08
2020−08−15
2020−08−22
2020−08−29
2020−09−05
2020−09−12
2020−09−19
2020−09−26
2020−10−03
2020−10−10
2020−10−17
2020−10−24
2020−10−31
2020−11−07
2020−11−14
2020−11−21
2020−11−28
2020−12−05
2020−12−12
2020−12−19
2020−12−26
2021−01−02
2021−01−09
2021−01−16
2021−01−23
2021−01−30
2021−02−06
2021−02−13
2021−02−20
2021−02−27
2021−03−06
2021−03−13
2021−03−20
2021−03−27
2021−04−03

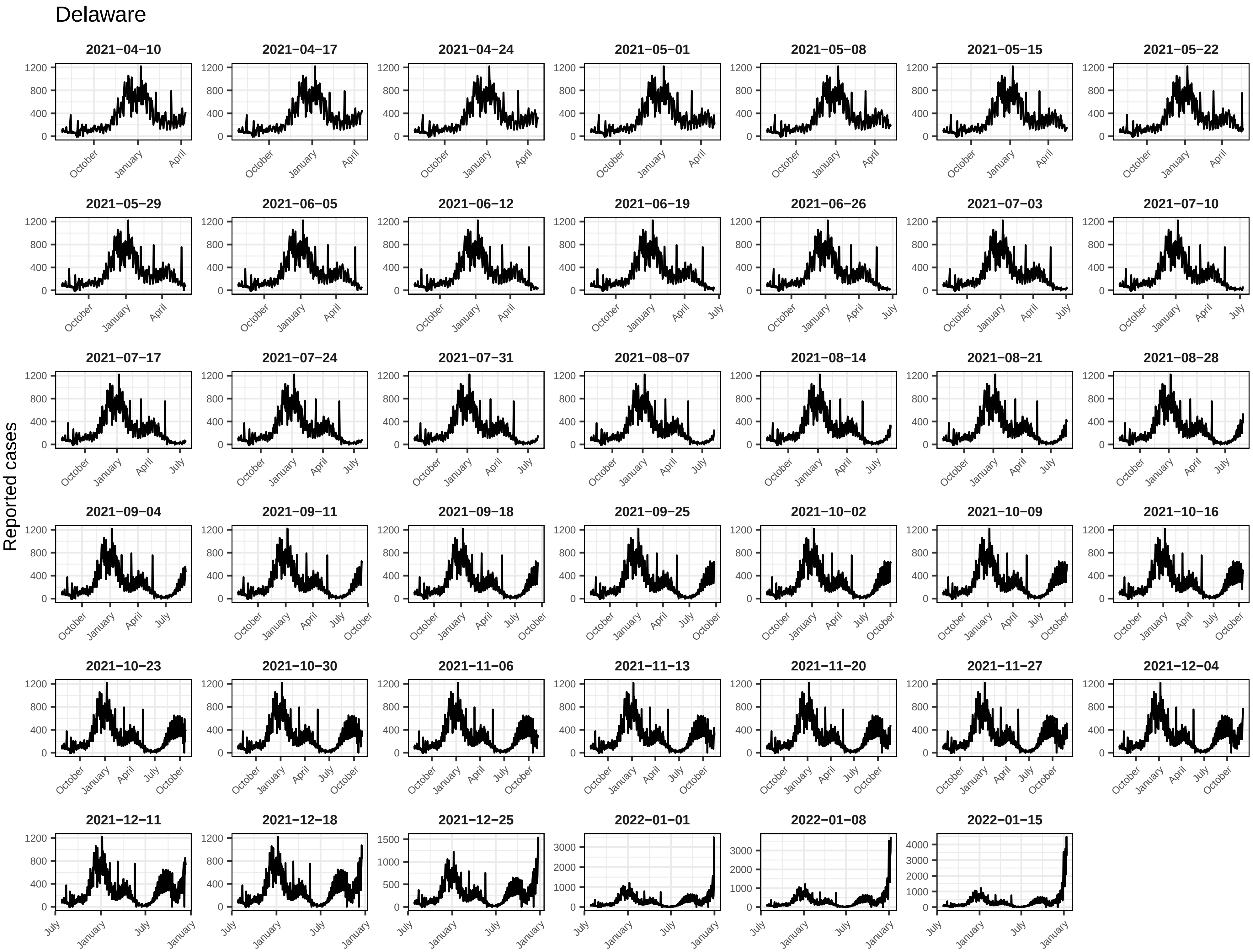
Delaware
Reported cases
2021−04−10
2021−04−17
2021−04−24
2021−05−01
2021−05−08
2021−05−15
2021−05−22
2021−05−29
2021−06−05
2021−06−12
2021−06−19
2021−06−26
2021−07−03
2021−07−10
2021−07−17
2021−07−24
2021−07−31
2021−08−07
2021−08−14
2021−08−21
2021−08−28
2021−09−04
2021−09−11
2021−09−18
2021−09−25
2021−10−02
2021−10−09
2021−10−16
2021−10−23
2021−10−30
2021−11−06
2021−11−13
2021−11−20
2021−11−27
2021−12−04
2021−12−11
2021−12−18
2021−12−25
2022−01−01
2022−01−08
2022−01−15
October
January
April
July

District of Columbia

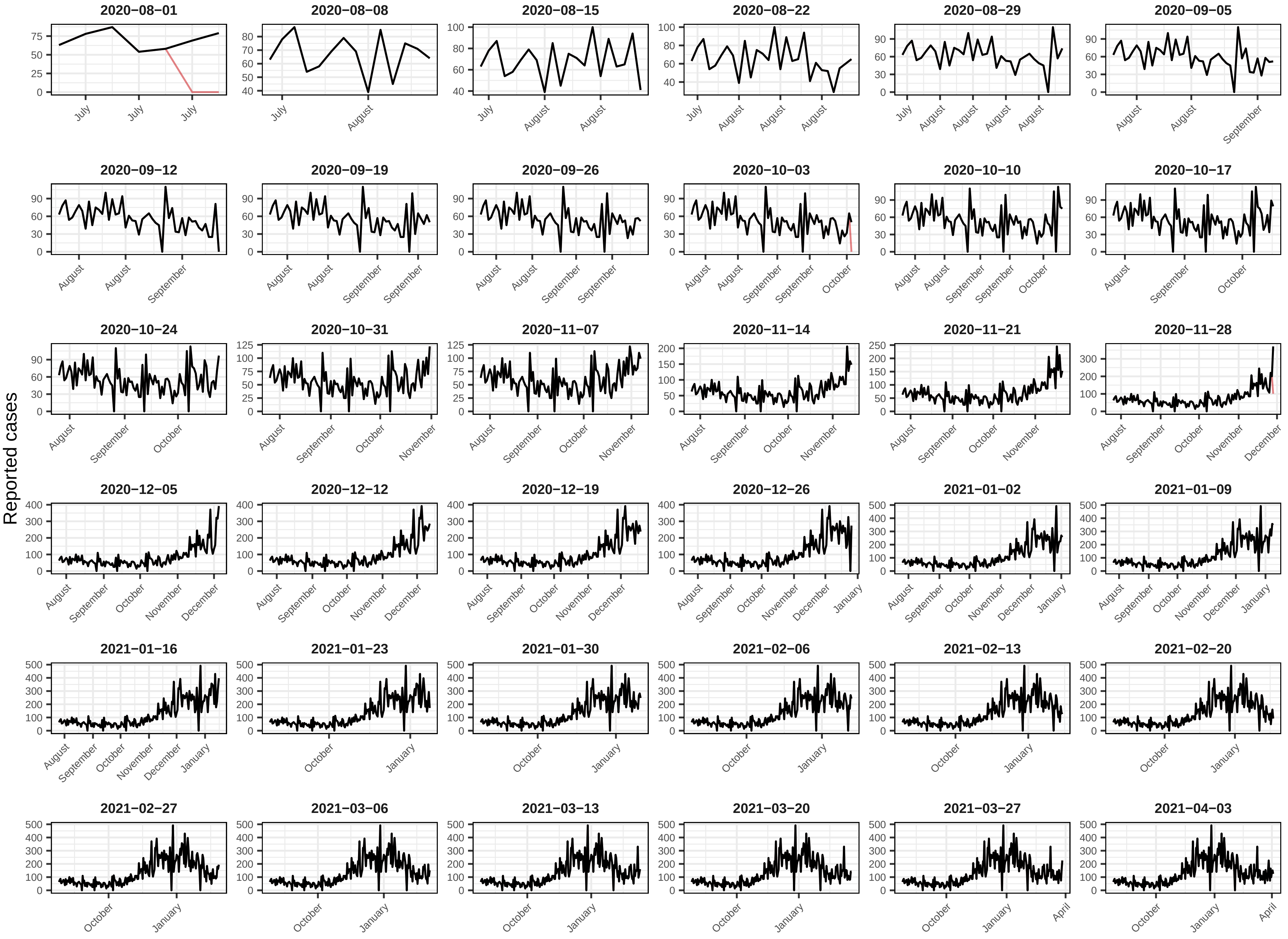

District of Columbia

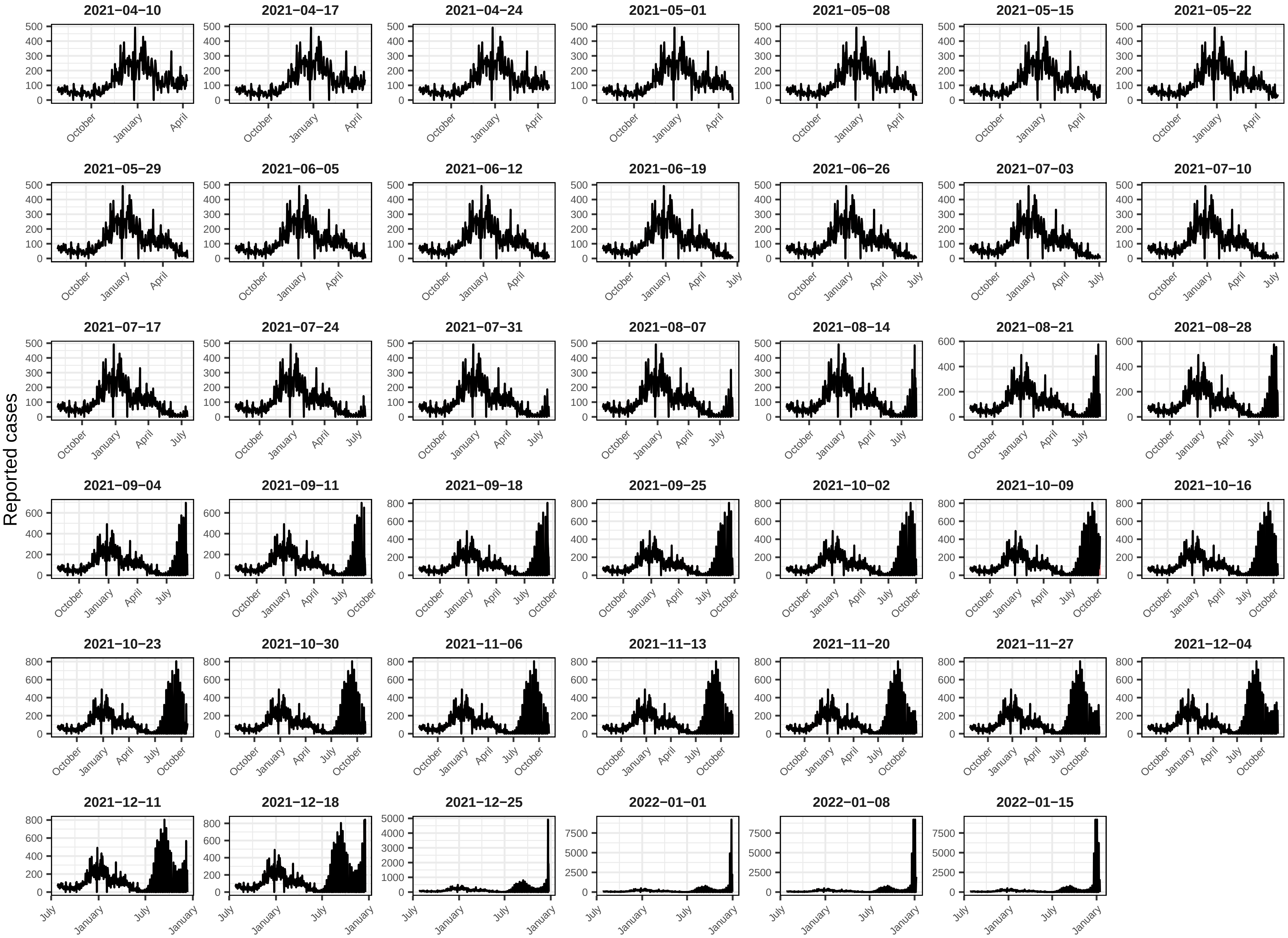

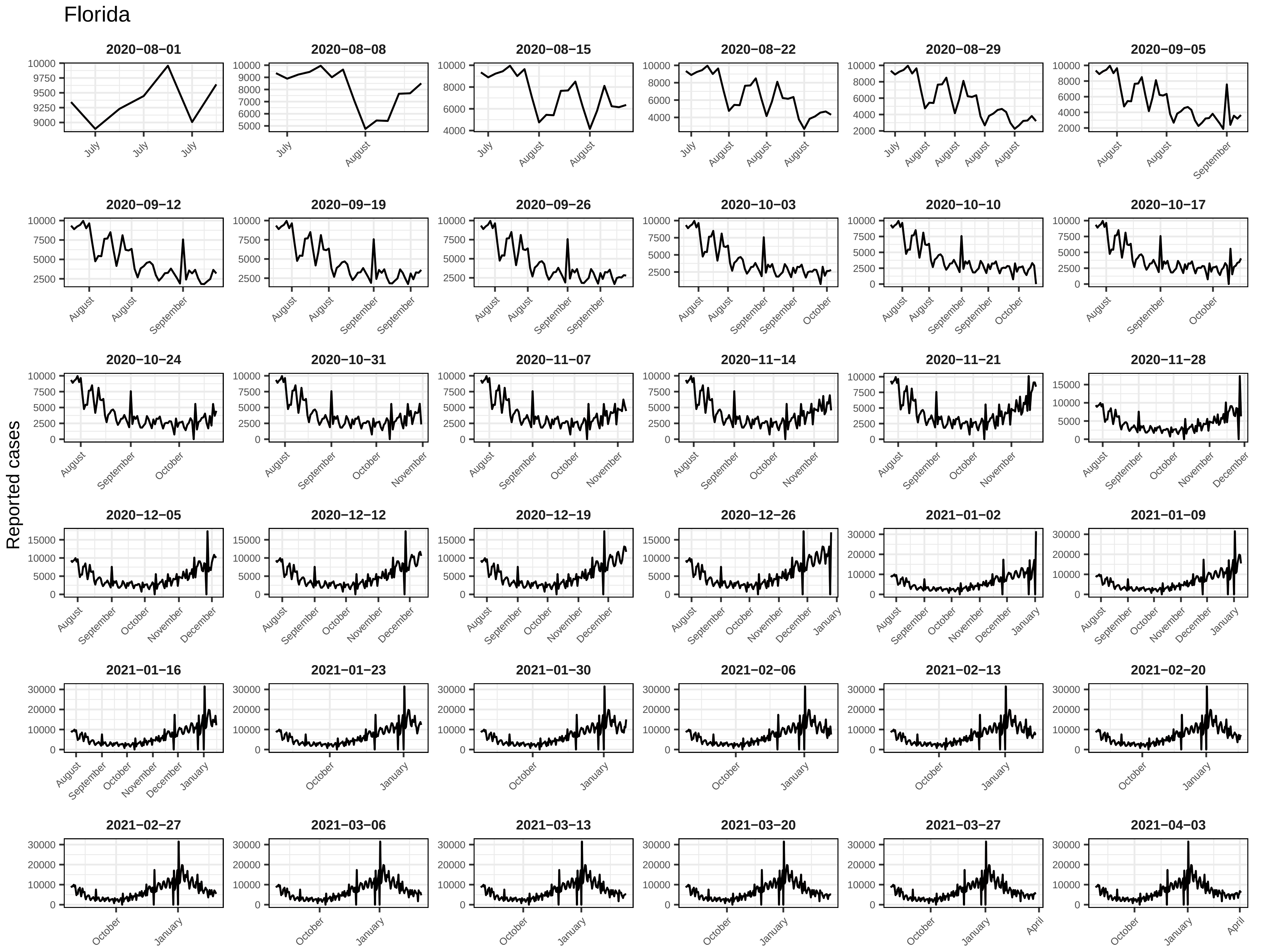
Florida
Reported cases
2020−08−01
2020−08−08
2020−08−15
2020−08−22
2020−08−29
2020−09−05
2020−09−12
2020−09−19
2020−09−26
2020−10−03
2020−10−10
2020−10−17
2020−10−24
2020−10−31
2020−11−07
2020−11−14
2020−11−21
2020−11−28
2020−12−05
2020−12−12
2020−12−19
2020−12−26
2021−01−02
2021−01−09
2021−01−16
2021−01−23
2021−01−30
2021−02−06
2021−02−13
2021−02−20
2021−02−27
2021−03−06
2021−03−13
2021−03−20
2021−03−27
2021−04−03

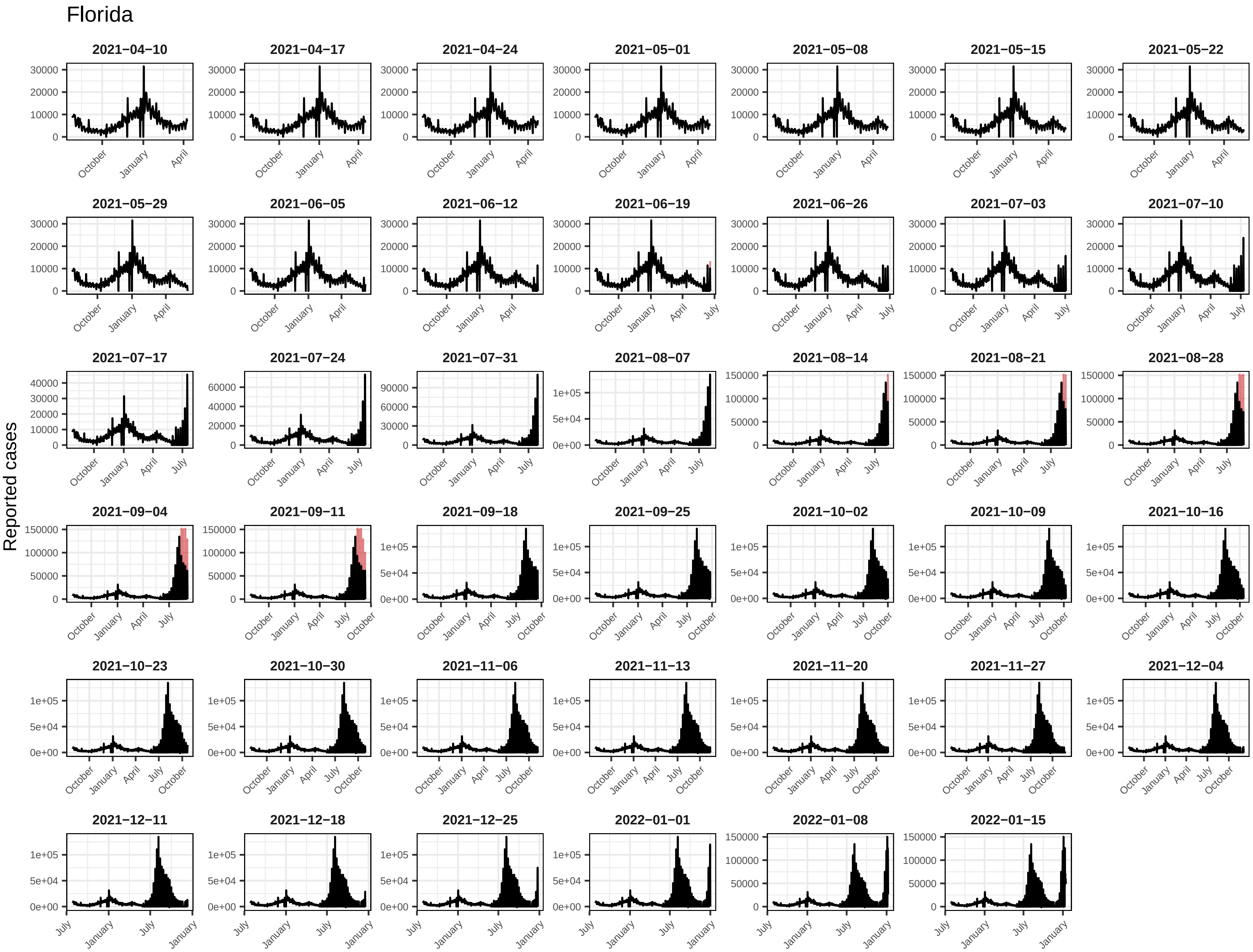
Florida
Reported cases
2021−04−10
2021−04−17
2021−04−24
2021−05−01
2021−05−08
2021−05−15
2021−05−22
2021−05−29
2021−06−05
2021−06−12
2021−06−19
2021−06−26
2021−07−03
2021−07−10
2021−07−17
2021−07−24
2021−07−31
2021−08−07
2021−08−14
2021−08−21
2021−08−28
2021−09−04
2021−09−11
2021−09−18
2021−09−25
2021−10−02
2021−10−09
2021−10−16
2021−10−23
2021−10−30
2021−11−06
2021−11−13
2021−11−20
2021−11−27
2021−12−04
2021−12−11
2021−12−18
2021−12−25
2022−01−01
2022−01−08
2022−01−15

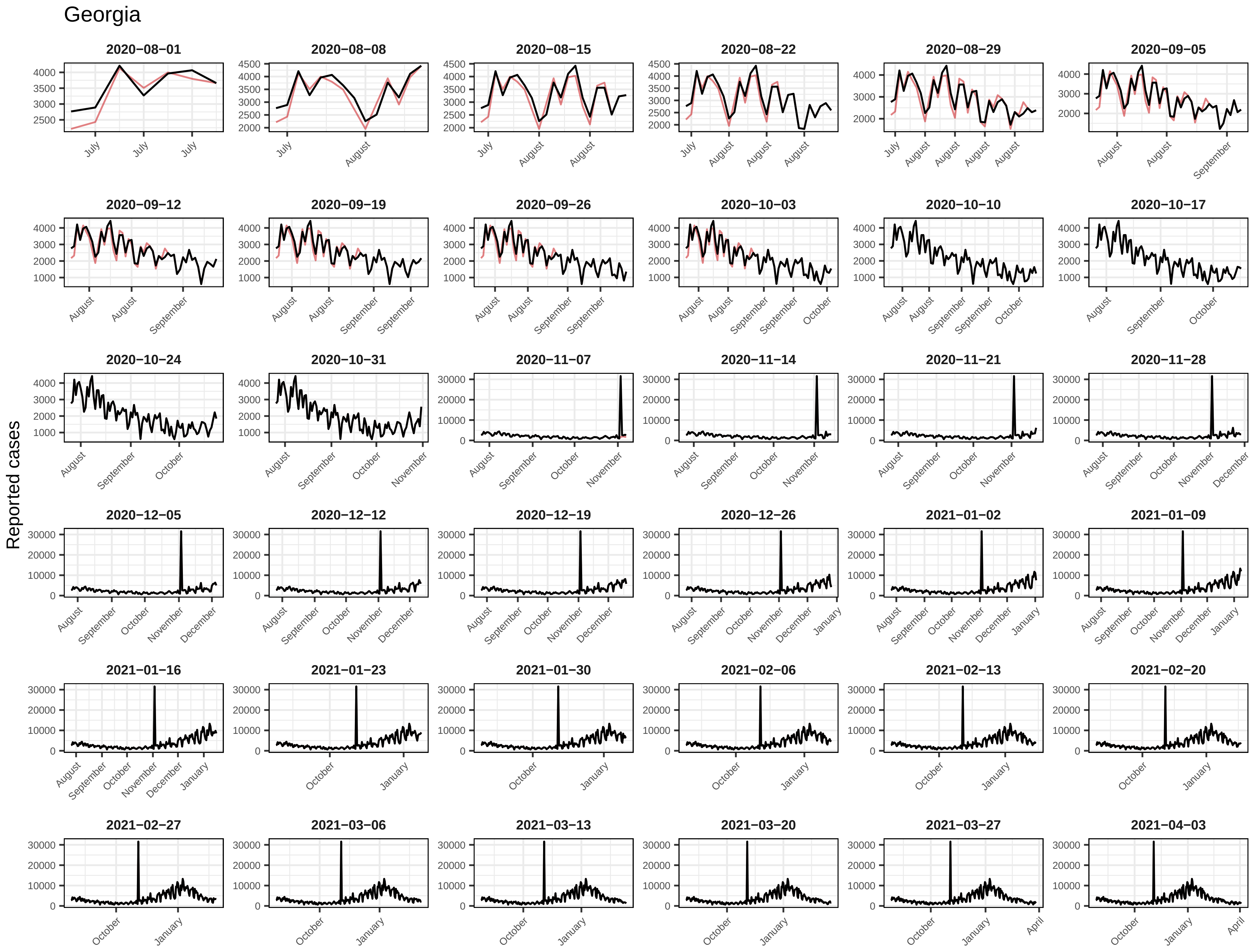
Georgia
Reported cases
2020−08−01
2020−08−08
2020−08−15
2020−08−22
2020−08−29
2020−09−05
2020−09−12
2020−09−19
2020−09−26
2020−10−03
2020−10−10
2020−10−17
2020−10−24
2020−10−31
2020−11−07
2020−11−14
2020−11−21
2020−11−28
2020−12−05
2020−12−12
2020−12−19
2020−12−26
2021−01−02
2021−01−09
2021−01−16
2021−01−23
2021−01−30
2021−02−06
2021−02−13
2021−02−20
2021−02−27
2021−03−06
2021−03−13
2021−03−20
2021−03−27
2021−04−03

Georgia

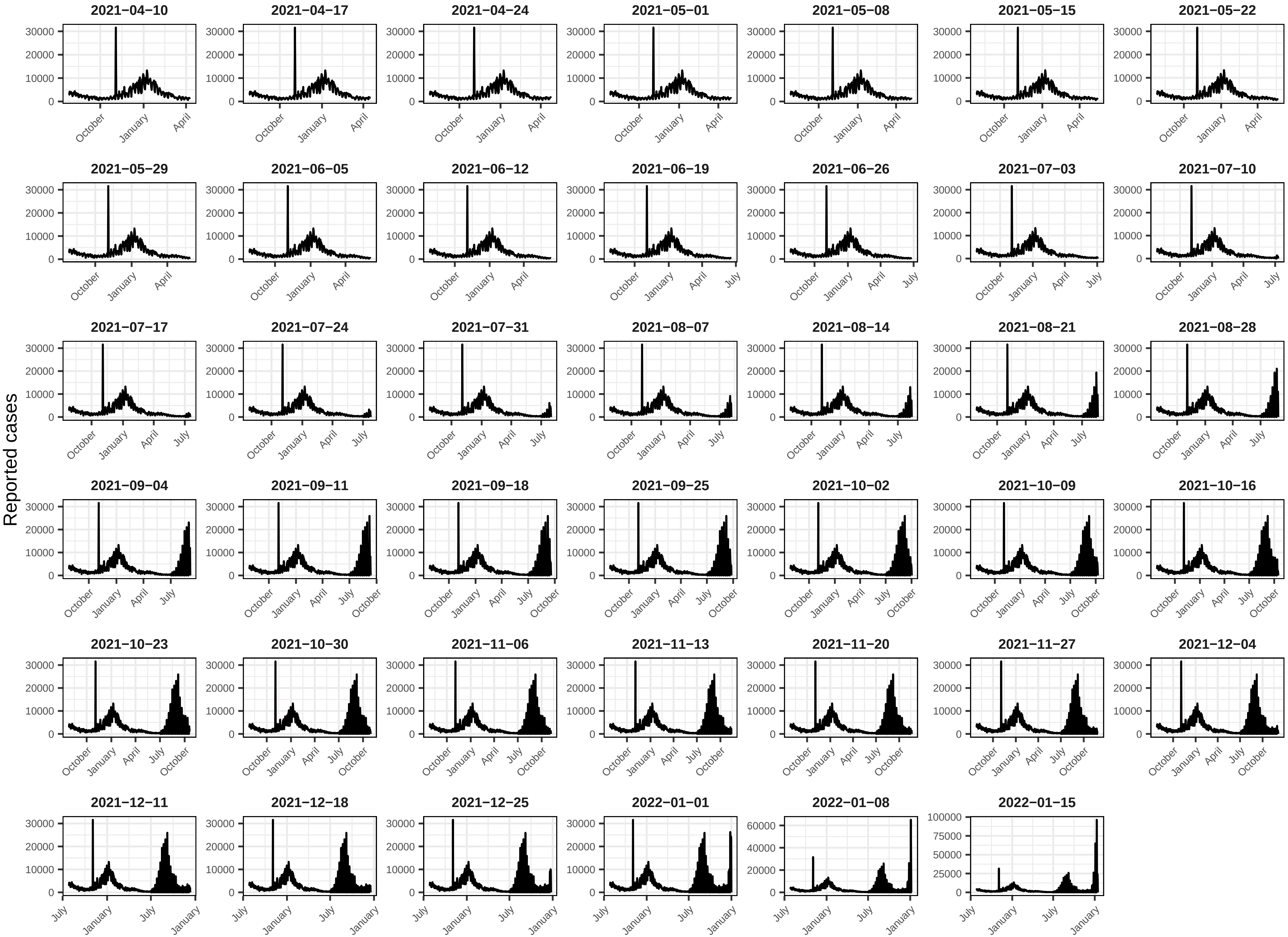

Reported cases
2021−04−10
2021−04−17
2021−04−24
2021−05−01
2021−05−08
2021−05−15
2021−05−22
2021−05−29
2021−06−05
2021−06−12
2021−06−19
2021−06−26
2021−07−03
2021−07−10
2021−07−17
2021−07−24
2021−07−31
2021−08−07
2021−08−14
2021−08−21
2021−08−28
2021−09−04
2021−09−11
2021−09−18
2021−09−25
2021−10−02
2021−10−09
2021−10−16
2021−10−23
2021−10−30
2021−11−06
2021−11−13
2021−11−20
2021−11−27
2021−12−04
2021−12−11
2021−12−18
2021−12−25
2022−01−01
2022−01−08
2022−01−15

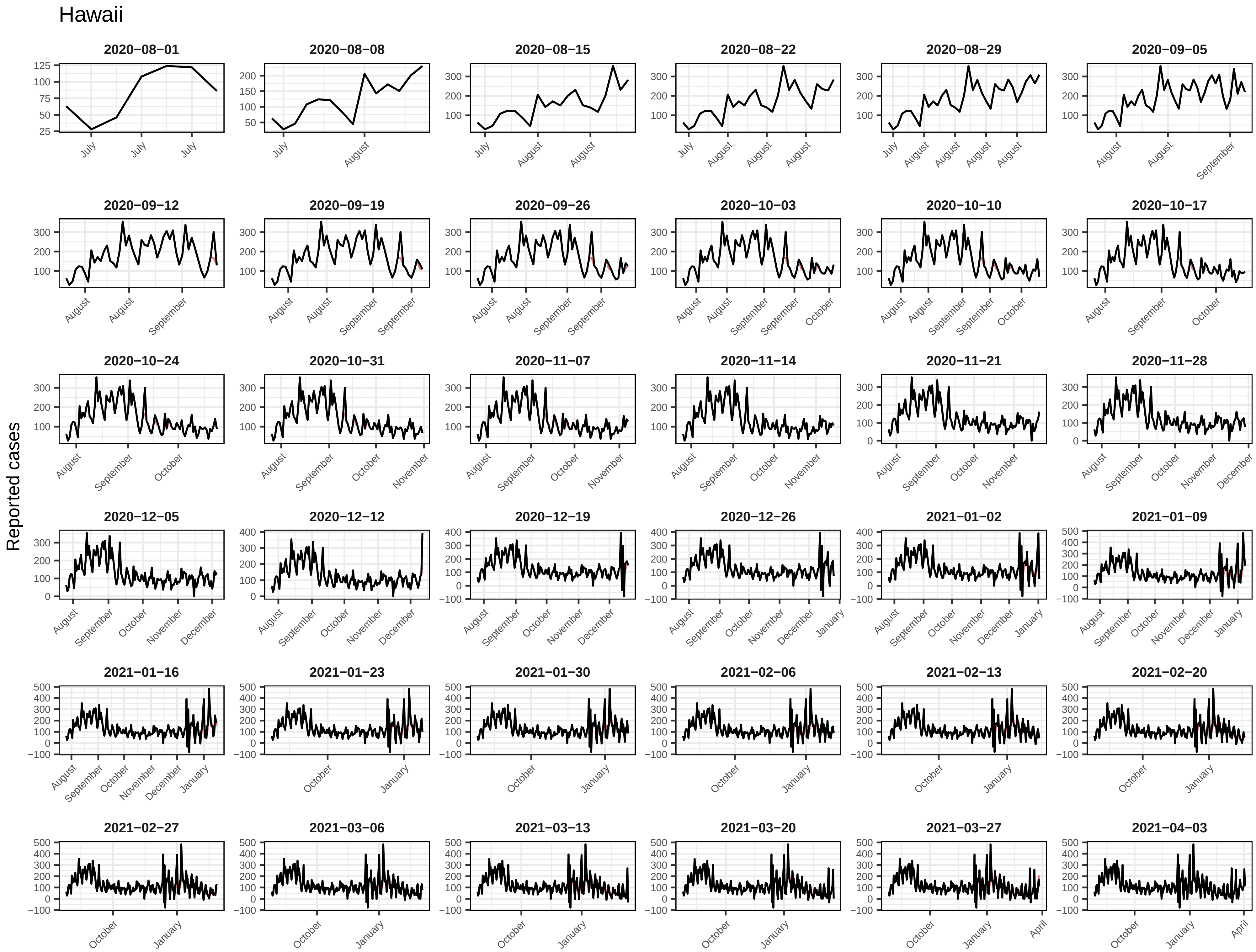
Hawaii
Reported cases
2020−08−01
2020−08−08
2020−08−15
2020−08−22
2020−08−29
2020−09−05
2020−09−12
2020−09−19
2020−09−26
2020−10−03
2020−10−10
2020−10−17
2020−10−24
2020−10−31
2020−11−07
2020−11−14
2020−11−21
2020−11−28
2020−12−05
2020−12−12
2020−12−19
2020−12−26
2021−01−02
2021−01−09
2021−01−16
2021−01−23
2021−01−30
2021−02−06
2021−02−13
2021−02−20
2021−02−27
2021−03−06
2021−03−13
2021−03−20
2021−03−27
2021−04−03

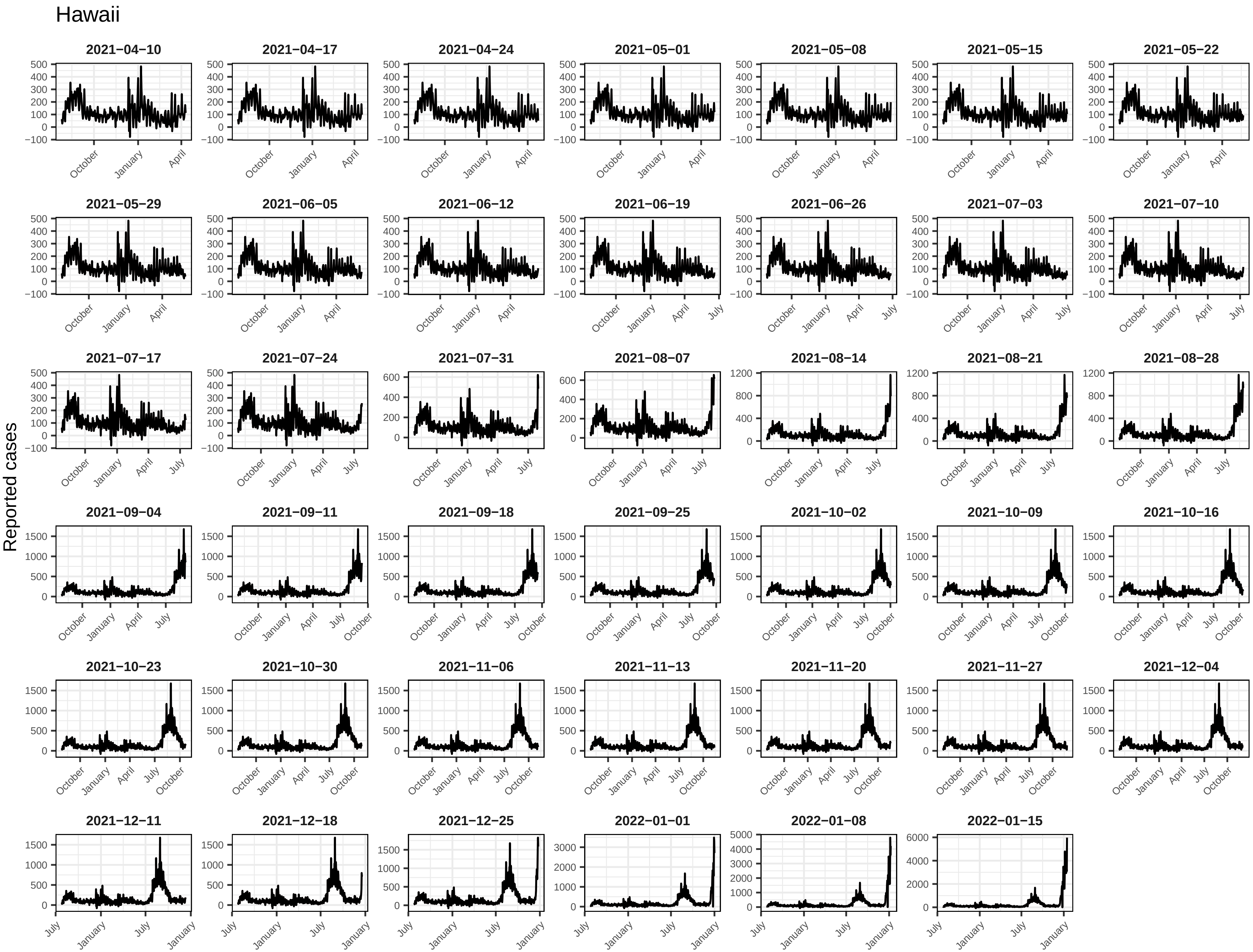
Hawaii
Reported cases
2021−04−10
2021−04−17
2021−04−24
2021−05−01
2021−05−08
2021−05−15
2021−05−22
2021−05−29
2021−06−05
2021−06−12
2021−06−19
2021−06−26
2021−07−03
2021−07−10
2021−07−17
2021−07−24
2021−07−31
2021−08−07
2021−08−14
2021−08−21
2021−08−28
2021−09−04
2021−09−11
2021−09−18
2021−09−25
2021−10−02
2021−10−09
2021−10−16
2021−10−23
2021−10−30
2021−11−06
2021−11−13
2021−11−20
2021−11−27
2021−12−04
2021−12−11
2021−12−18
2021−12−25
2022−01−01
2022−01−08
2022−01−15

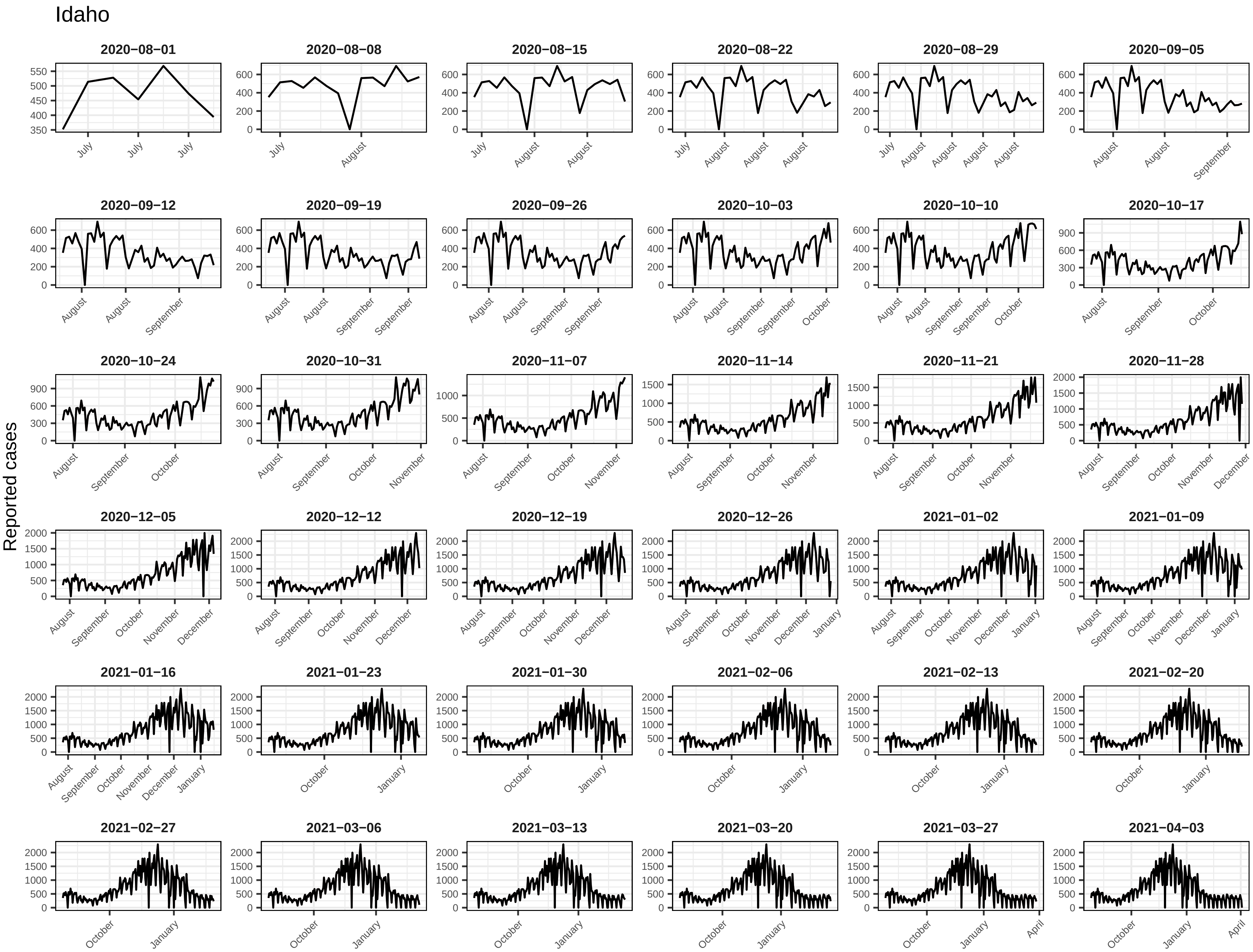
Idaho
Reported cases
2020−08−01
2020−08−08
2020−08−15
2020−08−22
2020−08−29
2020−09−05
2020−09−12
2020−09−19
2020−09−26
2020−10−03
2020−10−10
2020−10−17
2020−10−24
2020−10−31
2020−11−07
2020−11−14
2020−11−21
2020−11−28
2020−12−05
2020−12−12
2020−12−19
2020−12−26
2021−01−02
2021−01−09
2021−01−16
2021−01−23
2021−01−30
2021−02−06
2021−02−13
2021−02−20
2021−02−27
2021−03−06
2021−03−13
2021−03−20
2021−03−27
2021−04−03

Idaho

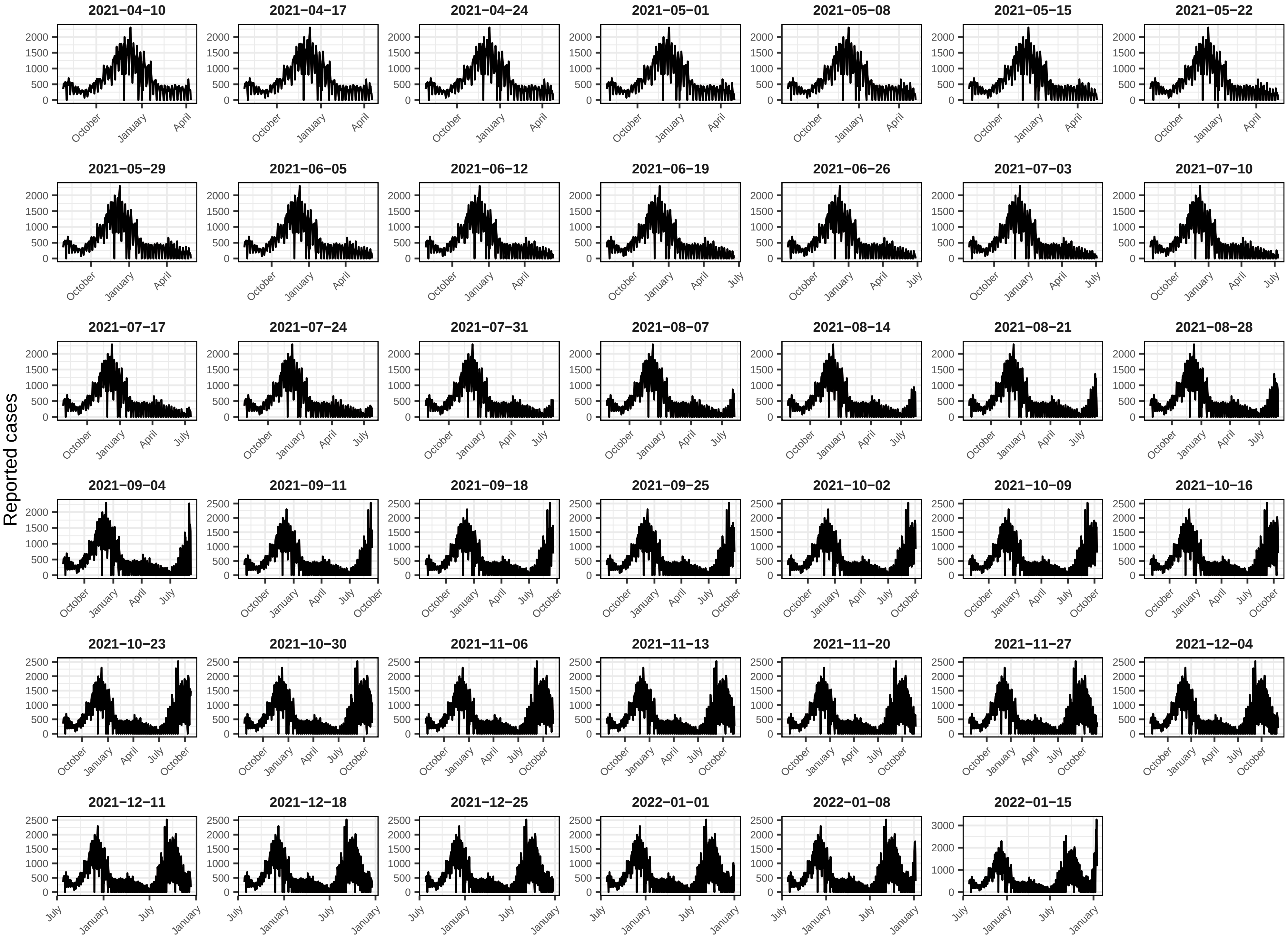

Reported cases
2021−04−10
2021−04−17
2021−04−24
2021−05−01
2021−05−08
2021−05−15
2021−05−22
2021−05−29
2021−06−05
2021−06−12
2021−06−19
2021−06−26
2021−07−03
2021−07−10
2021−07−17
2021−07−24
2021−07−31
2021−08−07
2021−08−14
2021−08−21
2021−08−28
2021−09−04
2021−09−11
2021−09−18
2021−09−25
2021−10−02
2021−10−09
2021−10−16
2021−10−23
2021−10−30
2021−11−06
2021−11−13
2021−11−20
2021−11−27
2021−12−04
2021−12−11
2021−12−18
2021−12−25
2022−01−01
2022−01−08
2022−01−15
0
500
1000
1500
2000
2500
3000
October
January
April
July

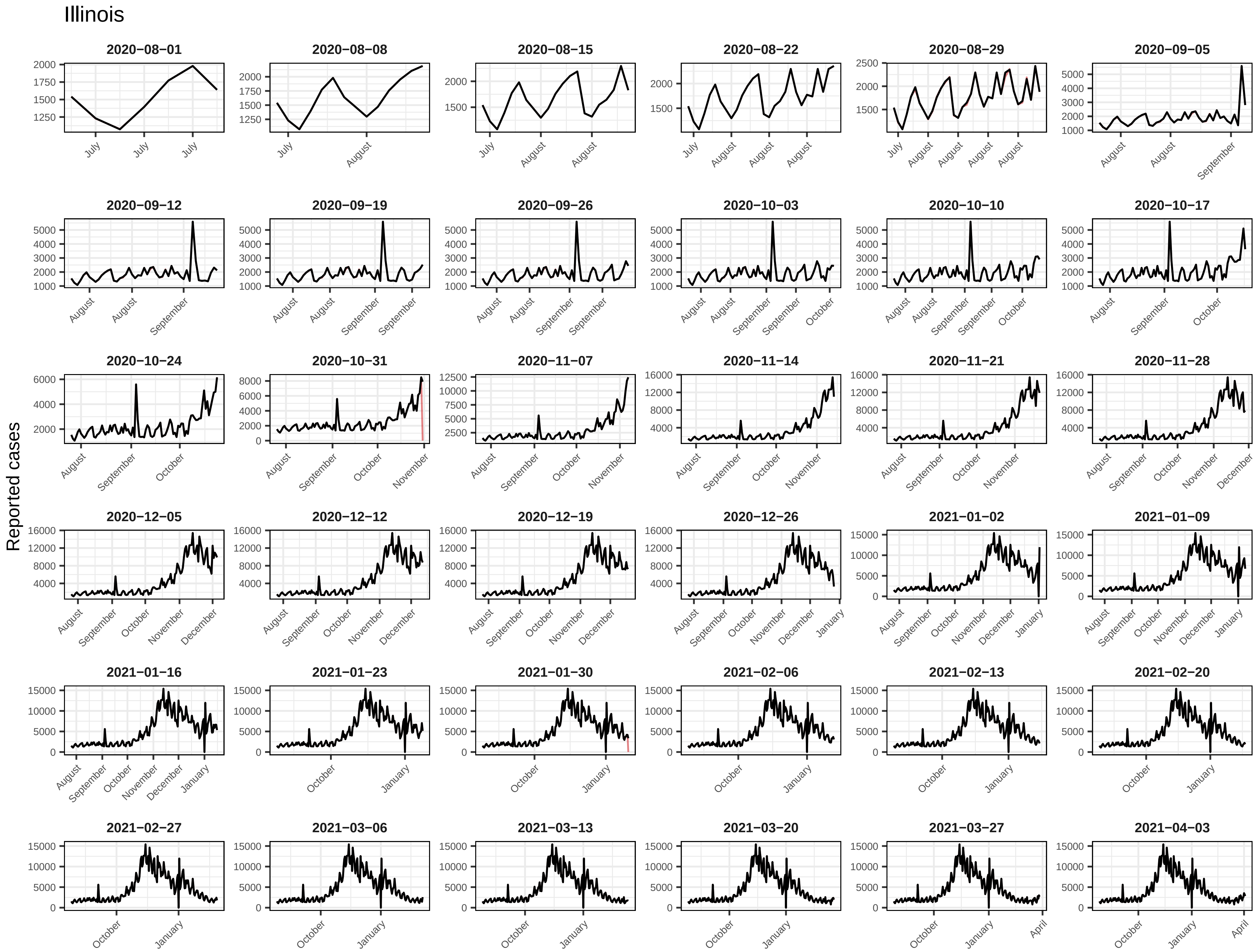
Illinois
Reported cases
2020−08−01
2020−08−08
2020−08−15
2020−08−22
2020−08−29
2020−09−05
2020−09−12
2020−09−19
2020−09−26
2020−10−03
2020−10−10
2020−10−17
2020−10−24
2020−10−31
2020−11−07
2020−11−14
2020−11−21
2020−11−28
2020−12−05
2020−12−12
2020−12−19
2020−12−26
2021−01−02
2021−01−09
2021−01−16
2021−01−23
2021−01−30
2021−02−06
2021−02−13
2021−02−20
2021−02−27
2021−03−06
2021−03−13
2021−03−20
2021−03−27
2021−04−03

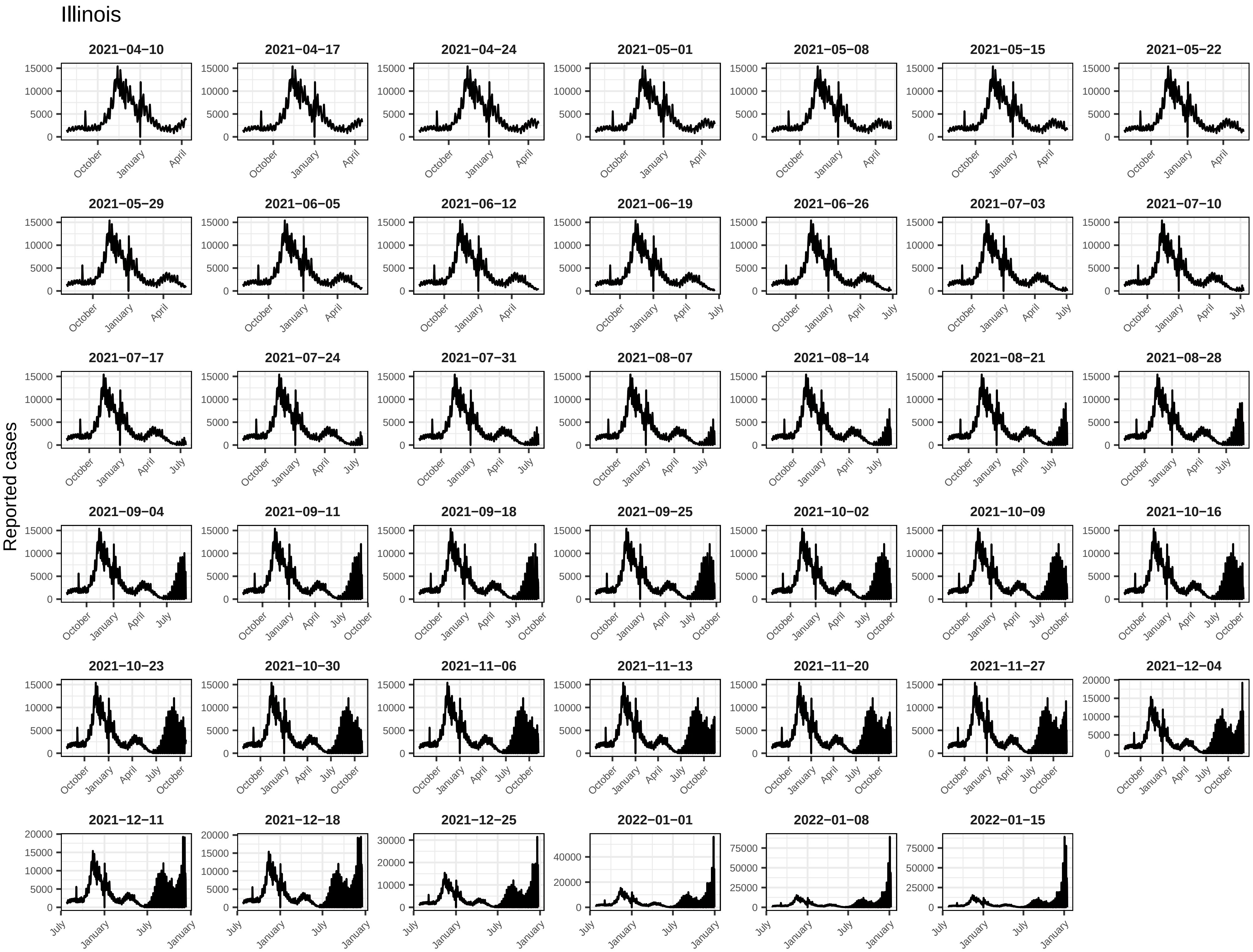
Illinois
Reported cases
2021−04−10
2021−04−17
2021−04−24
2021−05−01
2021−05−08
2021−05−15
2021−05−22
2021−05−29
2021−06−05
2021−06−12
2021−06−19
2021−06−26
2021−07−03
2021−07−10
2021−07−17
2021−07−24
2021−07−31
2021−08−07
2021−08−14
2021−08−21
2021−08−28
2021−09−04
2021−09−11
2021−09−18
2021−09−25
2021−10−02
2021−10−09
2021−10−16
2021−10−23
2021−10−30
2021−11−06
2021−11−13
2021−11−20
2021−11−27
2021−12−04
2021−12−11
2021−12−18
2021−12−25
2022−01−01
2022−01−08
2022−01−15
October
January
April
July

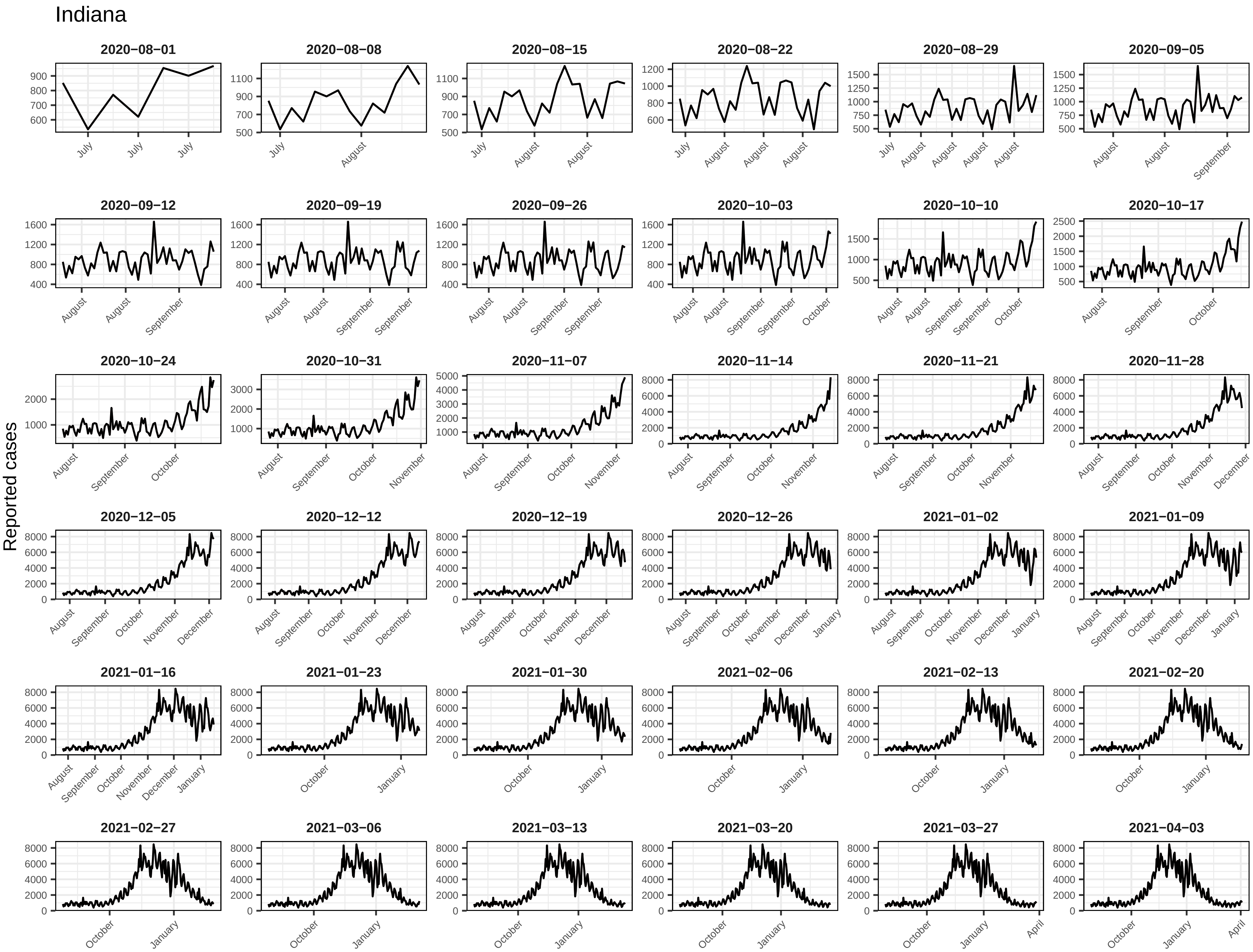
Indiana
Reported cases
2020−08−01
2020−08−08
2020−08−15
2020−08−22
2020−08−29
2020−09−05
2020−09−12
2020−09−19
2020−09−26
2020−10−03
2020−10−10
2020−10−17
2020−10−24
2020−10−31
2020−11−07
2020−11−14
2020−11−21
2020−11−28
2020−12−05
2020−12−12
2020−12−19
2020−12−26
2021−01−02
2021−01−09
2021−01−16
2021−01−23
2021−01−30
2021−02−06
2021−02−13
2021−02−20
2021−02−27
2021−03−06
2021−03−13
2021−03−20
2021−03−27
2021−04−03

Indiana

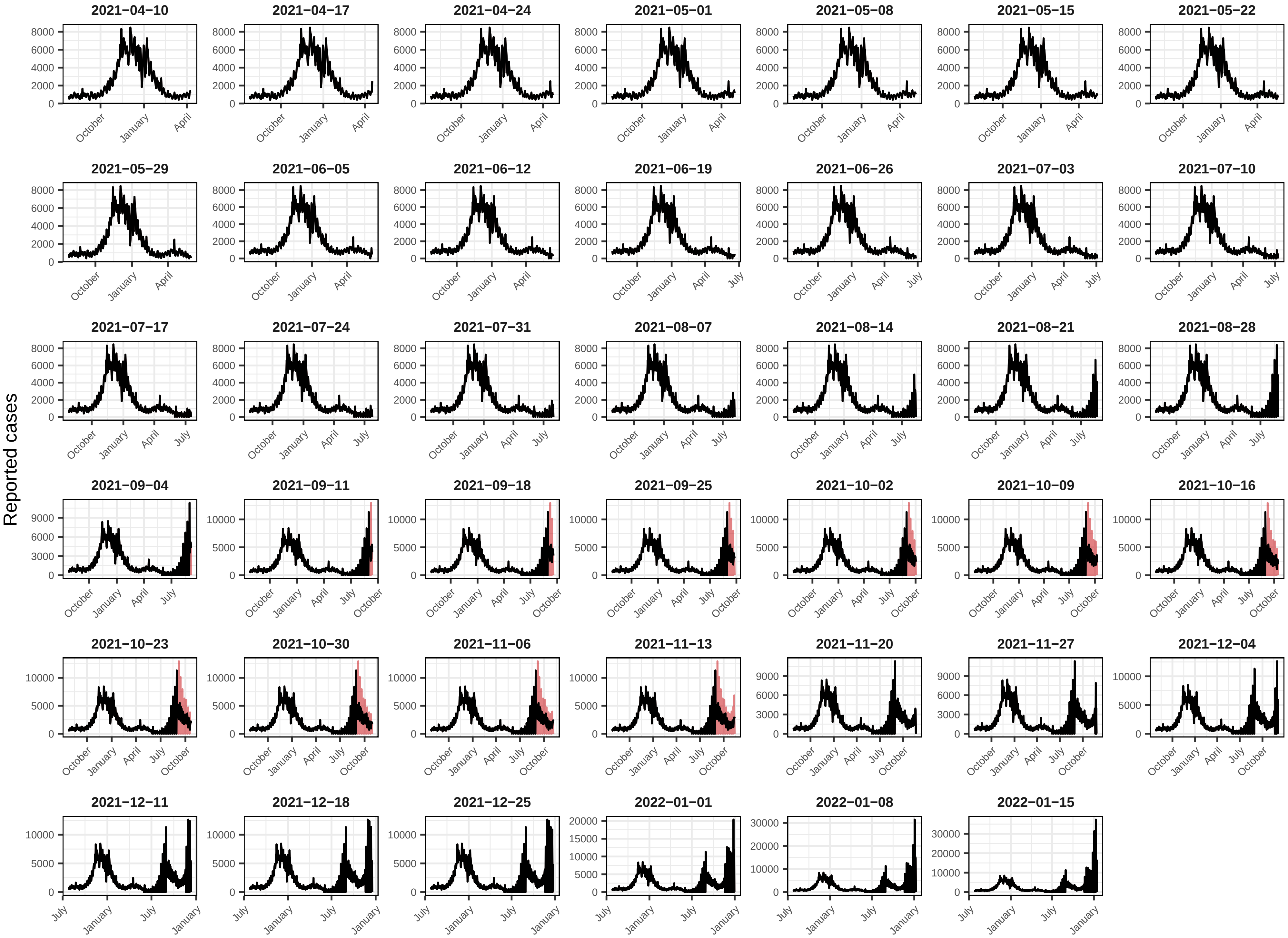

Reported cases
2021−04−10
2021−04−17
2021−04−24
2021−05−01
2021−05−08
2021−05−15
2021−05−22
2021−05−29
2021−06−05
2021−06−12
2021−06−19
2021−06−26
2021−07−03
2021−07−10
2021−07−17
2021−07−24
2021−07−31
2021−08−07
2021−08−14
2021−08−21
2021−08−28
2021−09−04
2021−09−11
2021−09−18
2021−09−25
2021−10−02
2021−10−09
2021−10−16
2021−10−23
2021−10−30
2021−11−06
2021−11−13
2021−11−20
2021−11−27
2021−12−04
2021−12−11
2021−12−18
2021−12−25
2022−01−01
2022−01−08
2022−01−15

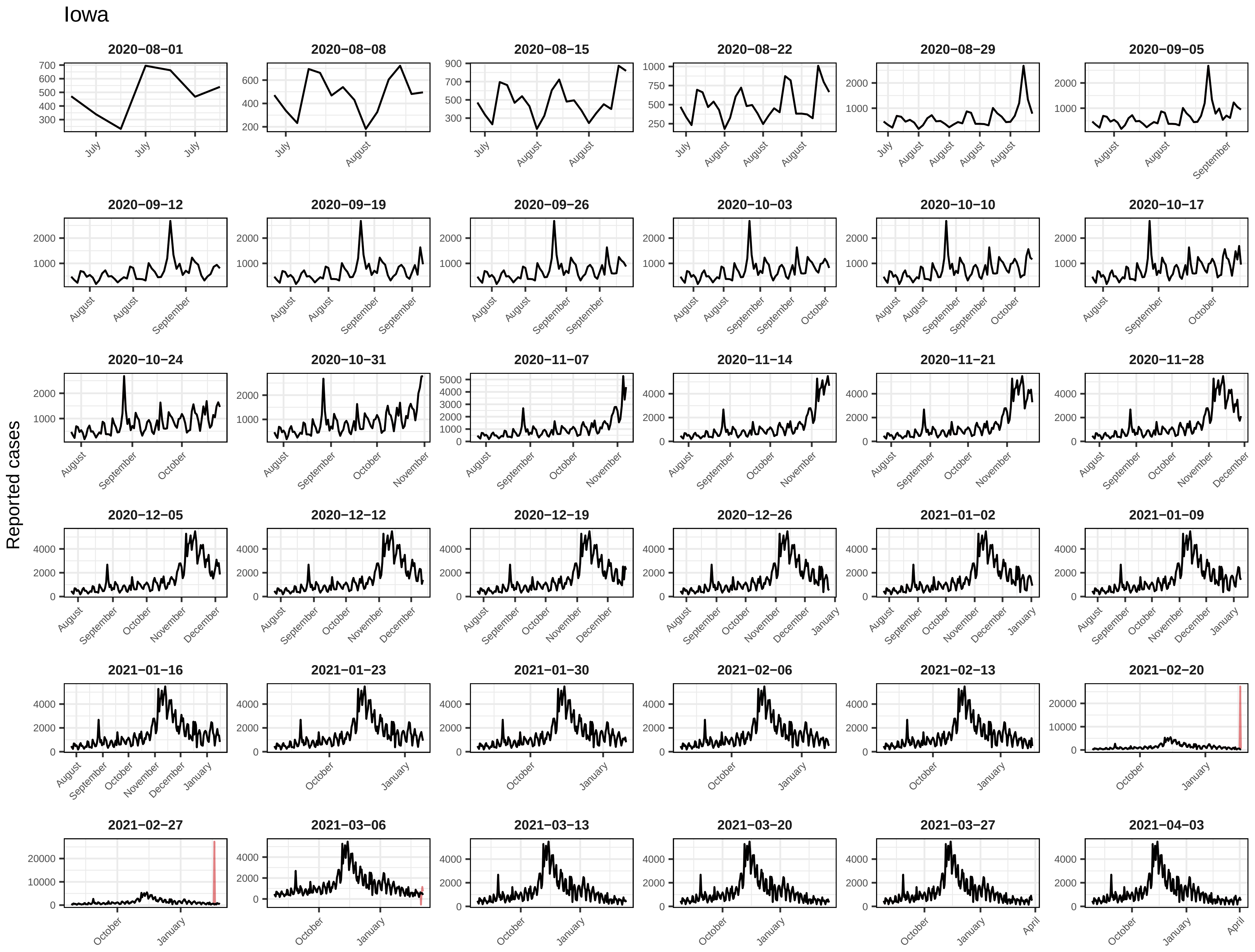
Iowa
Reported cases
2020−08−01
2020−08−08
2020−08−15
2020−08−22
2020−08−29
2020−09−05
2020−09−12
2020−09−19
2020−09−26
2020−10−03
2020−10−10
2020−10−17
2020−10−24
2020−10−31
2020−11−07
2020−11−14
2020−11−21
2020−11−28
2020−12−05
2020−12−12
2020−12−19
2020−12−26
2021−01−02
2021−01−09
2021−01−16
2021−01−23
2021−01−30
2021−02−06
2021−02−13
2021−02−20
2021−02−27
2021−03−06
2021−03−13
2021−03−20
2021−03−27
2021−04−03

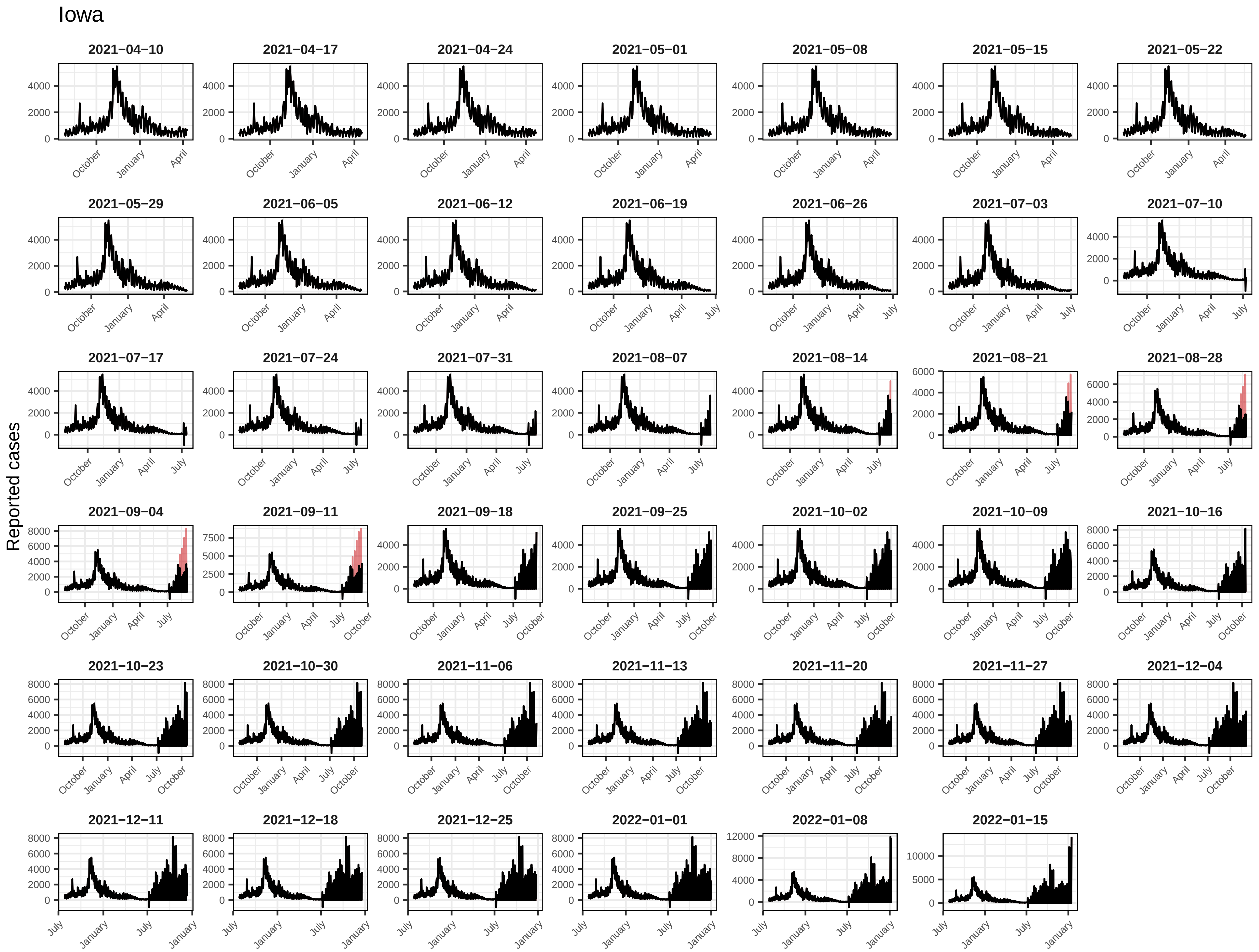
Iowa
Reported cases
2021−04−10
2021−04−17
2021−04−24
2021−05−01
2021−05−08
2021−05−15
2021−05−22
2021−05−29
2021−06−05
2021−06−12
2021−06−19
2021−06−26
2021−07−03
2021−07−10
2021−07−17
2021−07−24
2021−07−31
2021−08−07
2021−08−14
2021−08−21
2021−08−28
2021−09−04
2021−09−11
2021−09−18
2021−09−25
2021−10−02
2021−10−09
2021−10−16
2021−10−23
2021−10−30
2021−11−06
2021−11−13
2021−11−20
2021−11−27
2021−12−04
2021−12−11
2021−12−18
2021−12−25
2022−01−01
2022−01−08
2022−01−15

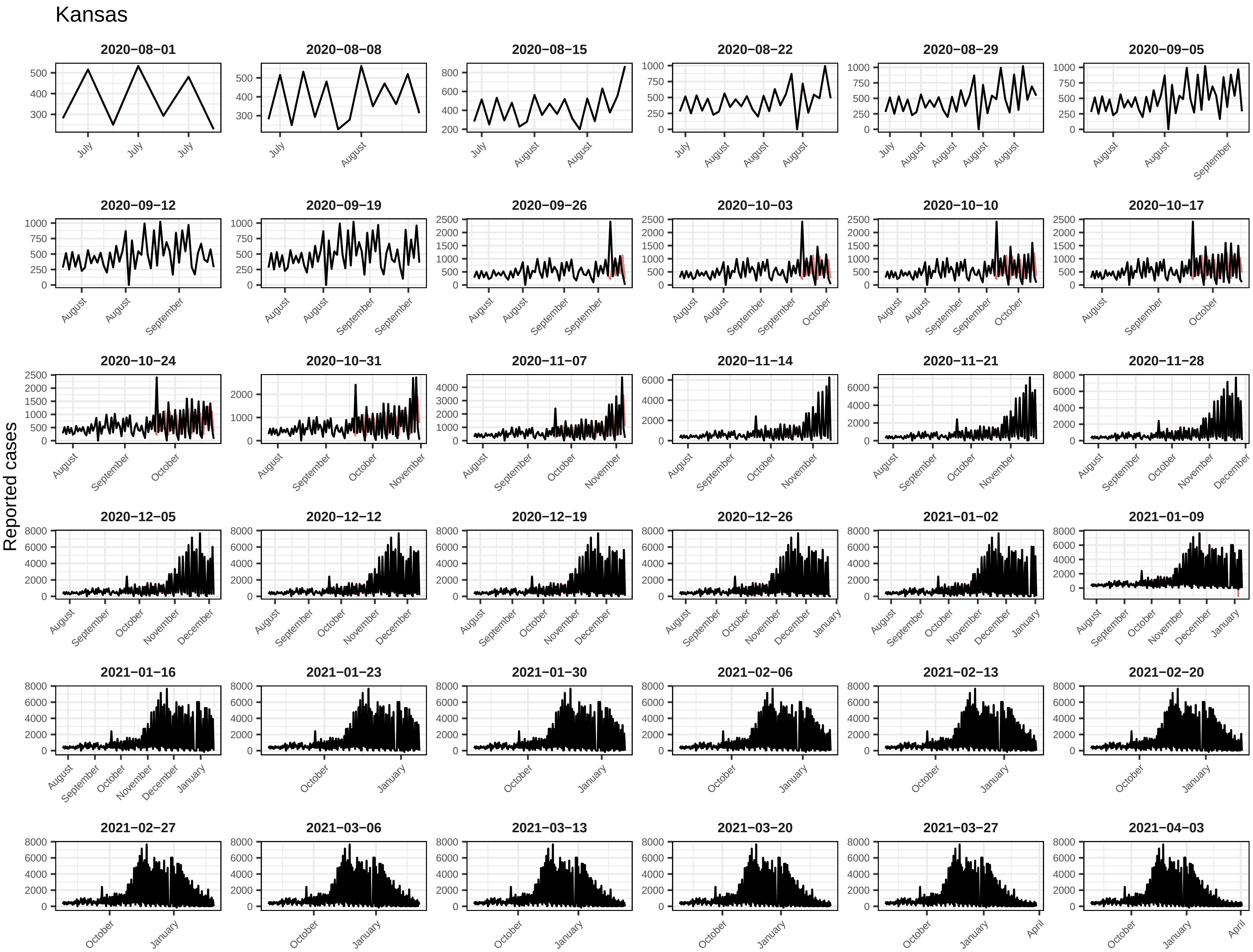

Kansas
Reported cases
2020−08−01
2020−08−08
2020−08−15
2020−08−22
2020−08−29
2020−09−05
2020−09−12
2020−09−19
2020−09−26
2020−10−03
2020−10−10
2020−10−17
2020−10−24
2020−10−31
2020−11−07
2020−11−14
2020−11−21
2020−11−28
2020−12−05
2020−12−12
2020−12−19
2020−12−26
2021−01−02
2021−01−09
2021−01−16
2021−01−23
2021−01−30
2021−02−06
2021−02−13
2021−02−20
2021−02−27
2021−03−06
2021−03−13
2021−03−20
2021−03−27
2021−04−03
July
August
September
October
November
December
January
April

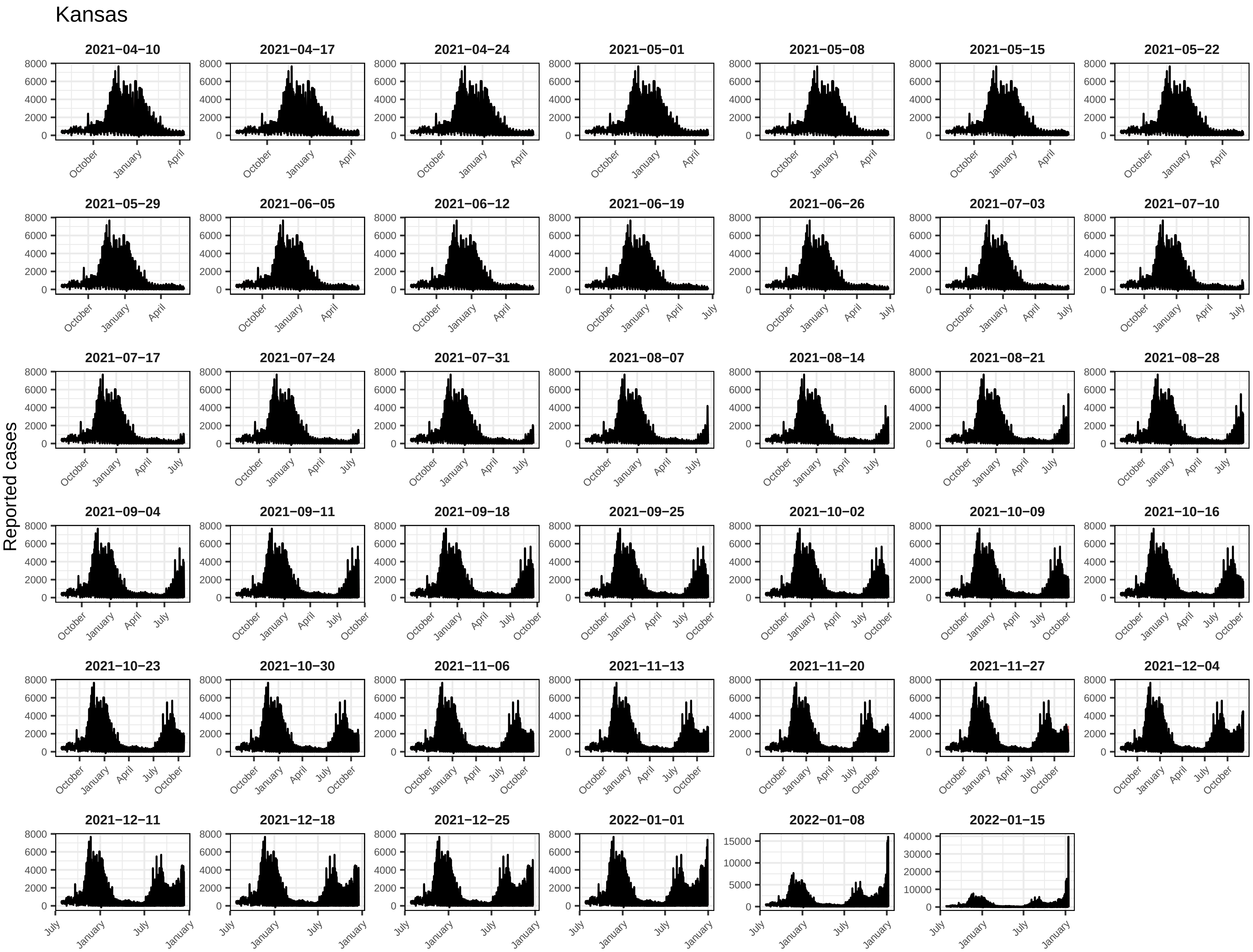
Kansas
Reported cases
2021−04−10
2021−04−17
2021−04−24
2021−05−01
2021−05−08
2021−05−15
2021−05−22
2021−05−29
2021−06−05
2021−06−12
2021−06−19
2021−06−26
2021−07−03
2021−07−10
2021−07−17
2021−07−24
2021−07−31
2021−08−07
2021−08−14
2021−08−21
2021−08−28
2021−09−04
2021−09−11
2021−09−18
2021−09−25
2021−10−02
2021−10−09
2021−10−16
2021−10−23
2021−10−30
2021−11−06
2021−11−13
2021−11−20
2021−11−27
2021−12−04
2021−12−11
2021−12−18
2021−12−25
2022−01−01
2022−01−08
2022−01−15
0
2000
4000
6000
8000
5000
10000
15000
20000
30000
40000
October
January
April
July

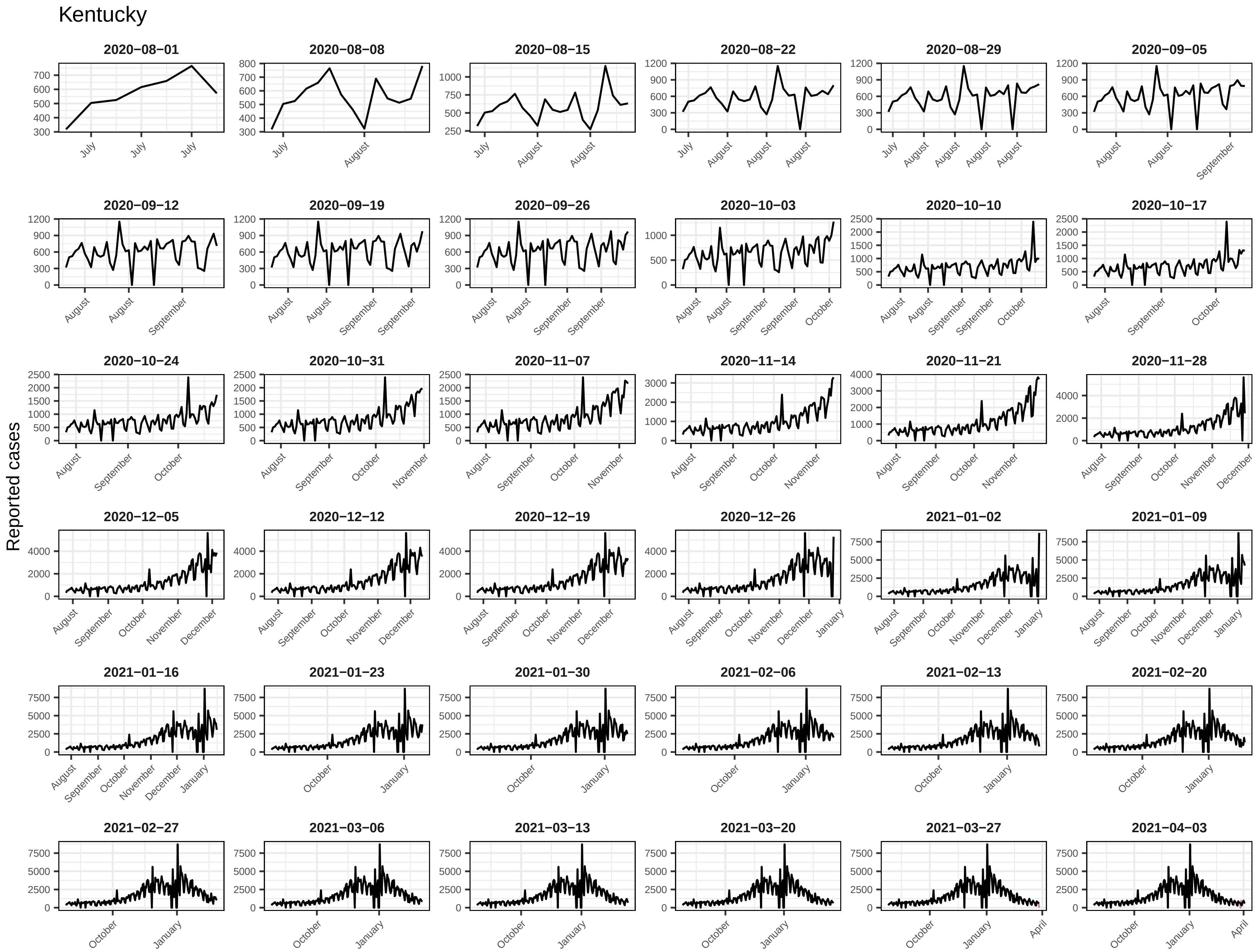
Kentucky
Reported cases
2020−08−01
2020−08−08
2020−08−15
2020−08−22
2020−08−29
2020−09−05
2020−09−12
2020−09−19
2020−09−26
2020−10−03
2020−10−10
2020−10−17
2020−10−24
2020−10−31
2020−11−07
2020−11−14
2020−11−21
2020−11−28
2020−12−05
2020−12−12
2020−12−19
2020−12−26
2021−01−02
2021−01−09
2021−01−16
2021−01−23
2021−01−30
2021−02−06
2021−02−13
2021−02−20
2021−02−27
2021−03−06
2021−03−13
2021−03−20
2021−03−27
2021−04−03

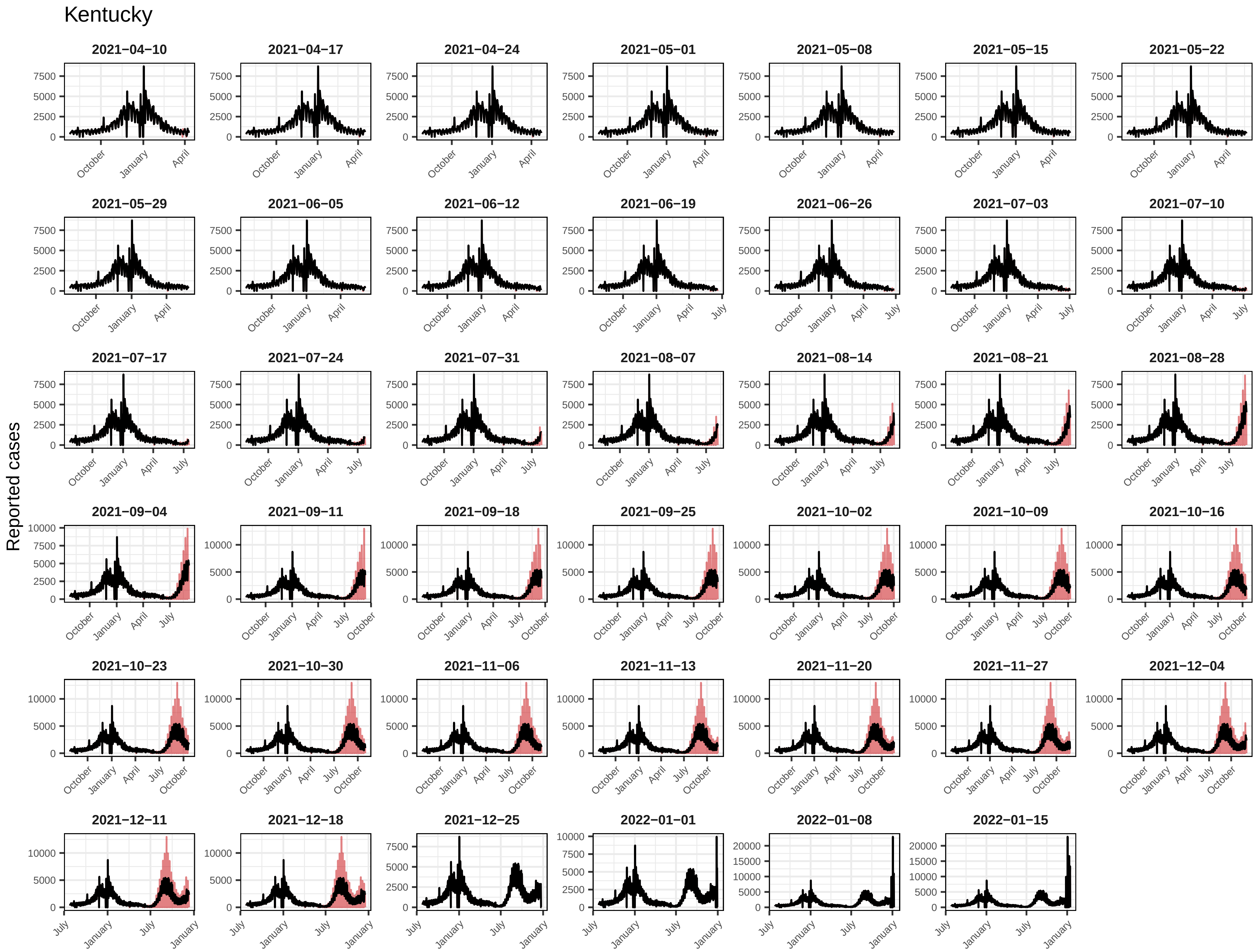
Kentucky
Reported cases
2021−04−10
2021−04−17
2021−04−24
2021−05−01
2021−05−08
2021−05−15
2021−05−22
2021−05−29
2021−06−05
2021−06−12
2021−06−19
2021−06−26
2021−07−03
2021−07−10
2021−07−17
2021−07−24
2021−07−31
2021−08−07
2021−08−14
2021−08−21
2021−08−28
2021−09−04
2021−09−11
2021−09−18
2021−09−25
2021−10−02
2021−10−09
2021−10−16
2021−10−23
2021−10−30
2021−11−06
2021−11−13
2021−11−20
2021−11−27
2021−12−04
2021−12−11
2021−12−18
2021−12−25
2022−01−01
2022−01−08
2022−01−15
0
2500
5000
7500
10000
15000
20000
July
October
January
April

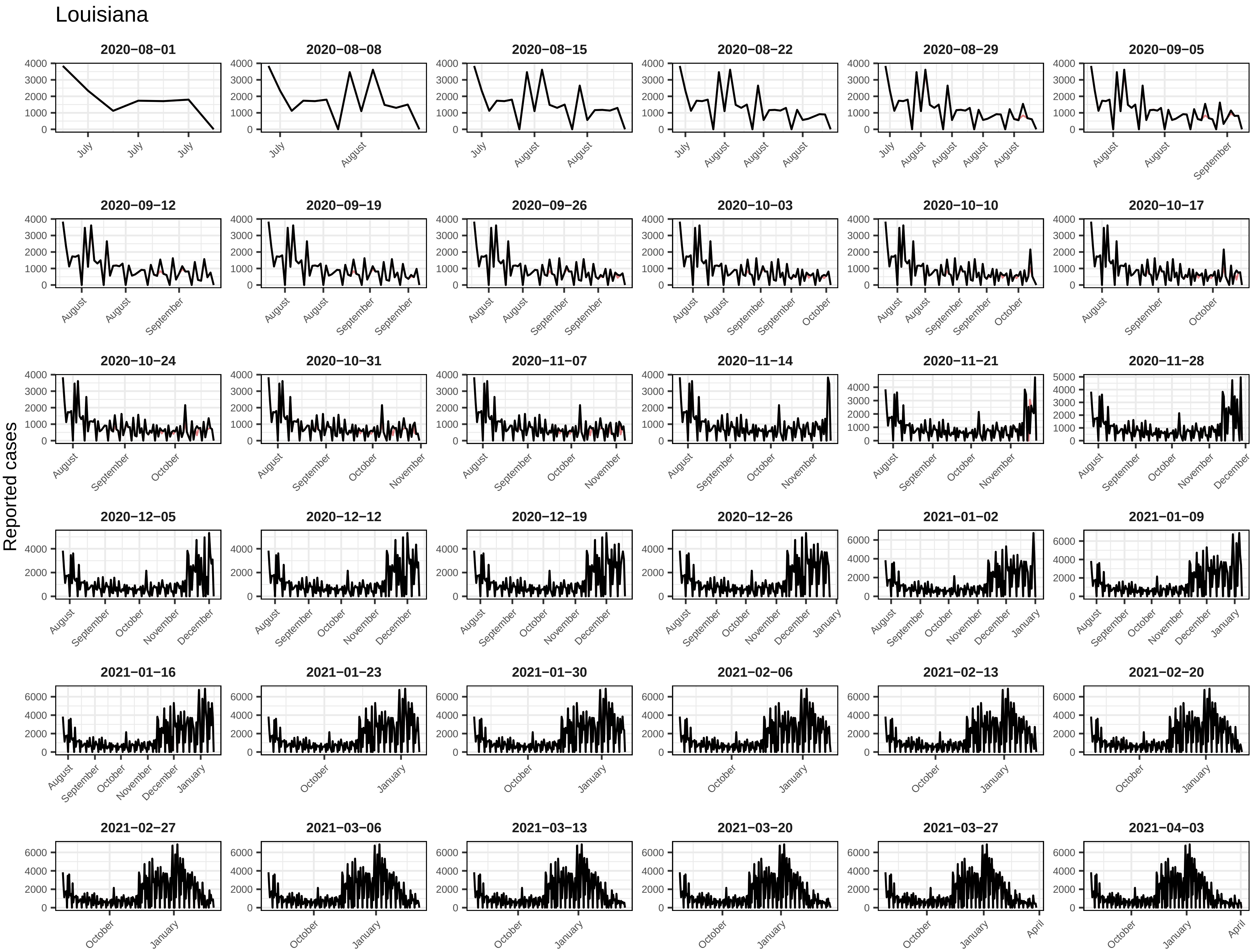
Louisiana
Reported cases
2020−08−01
2020−08−08
2020−08−15
2020−08−22
2020−08−29
2020−09−05
2020−09−12
2020−09−19
2020−09−26
2020−10−03
2020−10−10
2020−10−17
2020−10−24
2020−10−31
2020−11−07
2020−11−14
2020−11−21
2020−11−28
2020−12−05
2020−12−12
2020−12−19
2020−12−26
2021−01−02
2021−01−09
2021−01−16
2021−01−23
2021−01−30
2021−02−06
2021−02−13
2021−02−20
2021−02−27
2021−03−06
2021−03−13
2021−03−20
2021−03−27
2021−04−03

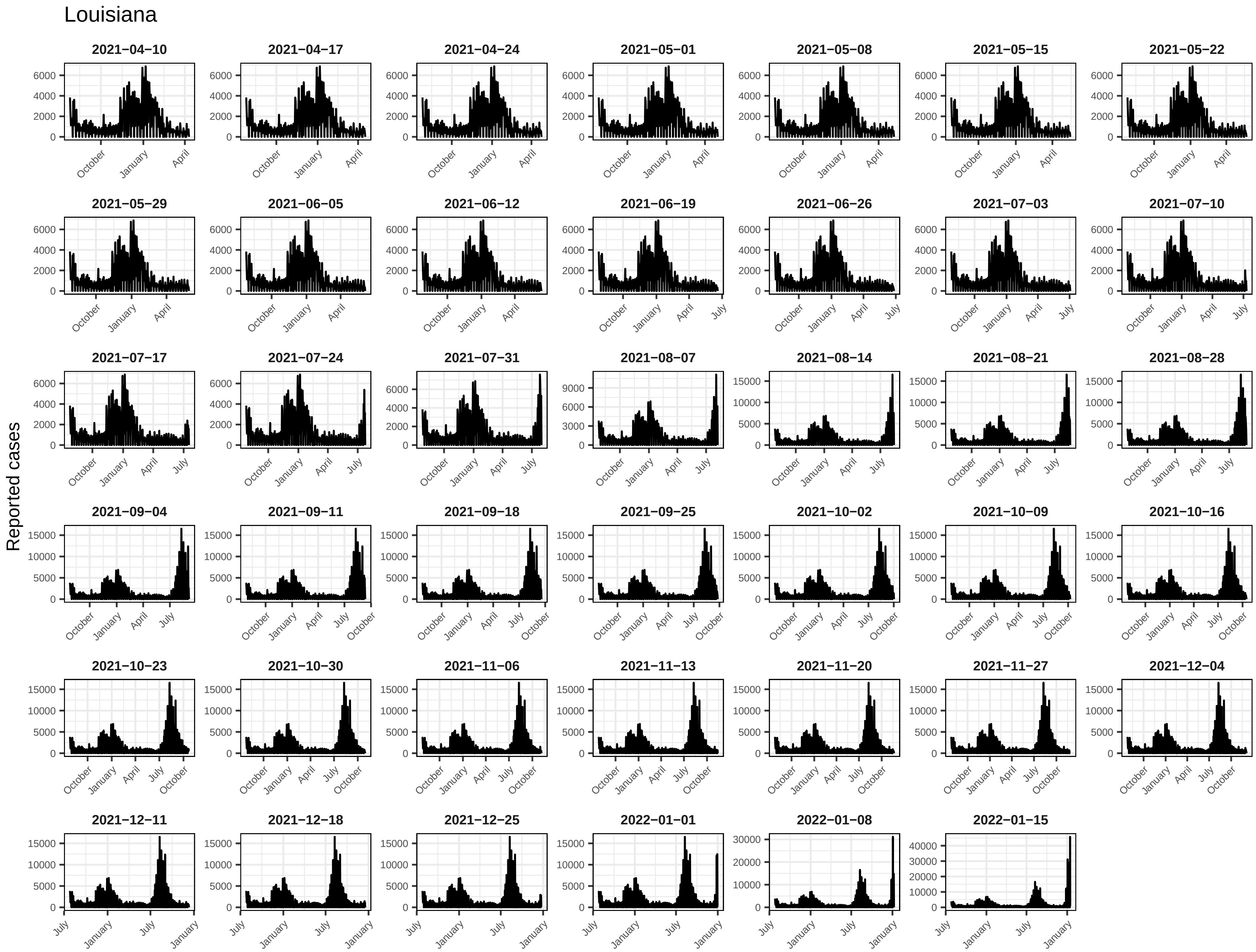
Louisiana
Reported cases
2021−04−10
2021−04−17
2021−04−24
2021−05−01
2021−05−08
2021−05−15
2021−05−22
2021−05−29
2021−06−05
2021−06−12
2021−06−19
2021−06−26
2021−07−03
2021−07−10
2021−07−17
2021−07−24
2021−07−31
2021−08−07
2021−08−14
2021−08−21
2021−08−28
2021−09−04
2021−09−11
2021−09−18
2021−09−25
2021−10−02
2021−10−09
2021−10−16
2021−10−23
2021−10−30
2021−11−06
2021−11−13
2021−11−20
2021−11−27
2021−12−04
2021−12−11
2021−12−18
2021−12−25
2022−01−01
2022−01−08
2022−01−15
October
January
April
July

Maine

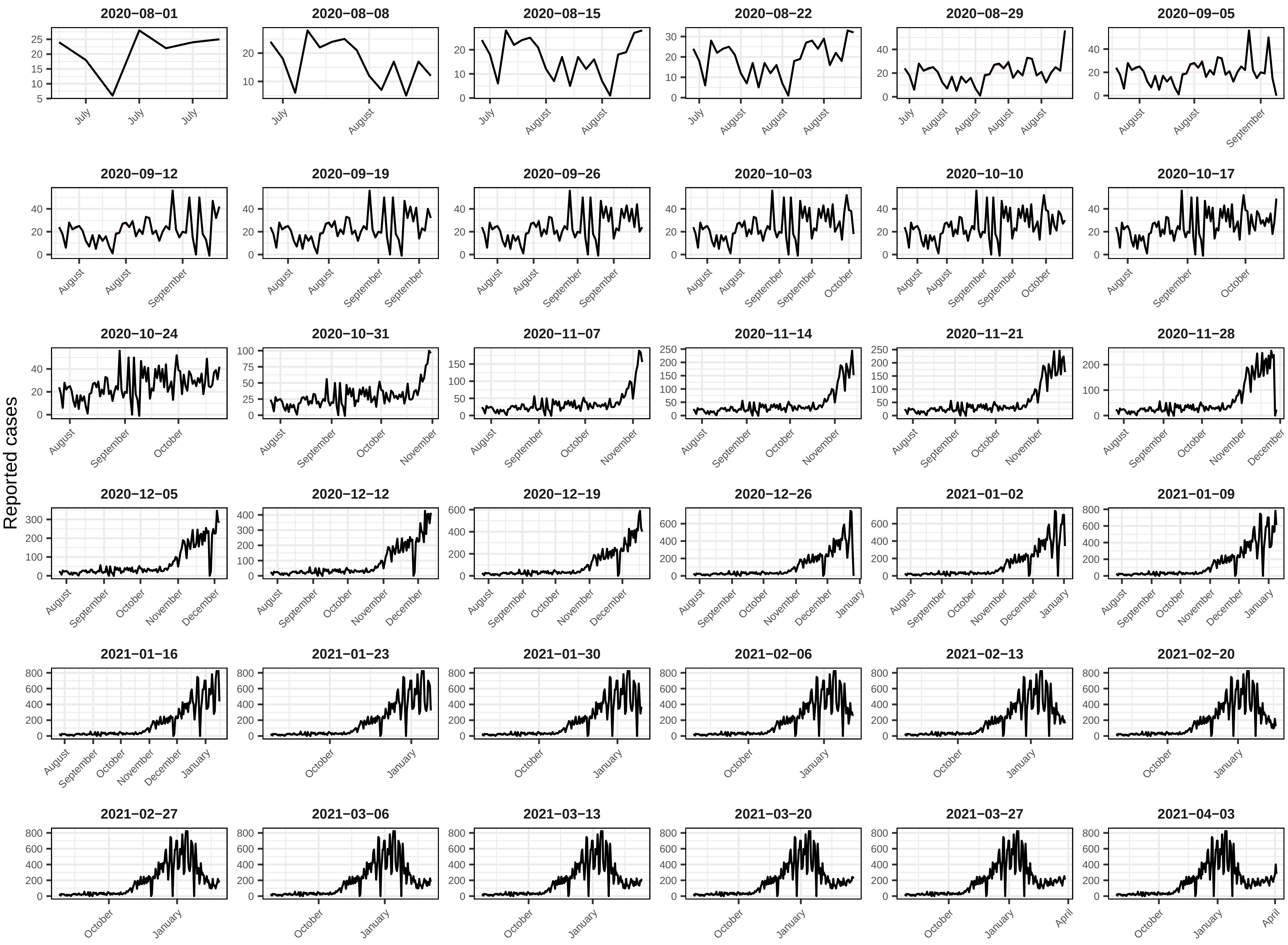

Reported cases
2020−08−01
2020−08−08
2020−08−15
2020−08−22
2020−08−29
2020−09−05
2020−09−12
2020−09−19
2020−09−26
2020−10−03
2020−10−10
2020−10−17
2020−10−24
2020−10−31
2020−11−07
2020−11−14
2020−11−21
2020−11−28
2020−12−05
2020−12−12
2020−12−19
2020−12−26
2021−01−02
2021−01−09
2021−01−16
2021−01−23
2021−01−30
2021−02−06
2021−02−13
2021−02−20
2021−02−27
2021−03−06
2021−03−13
2021−03−20
2021−03−27
2021−04−03

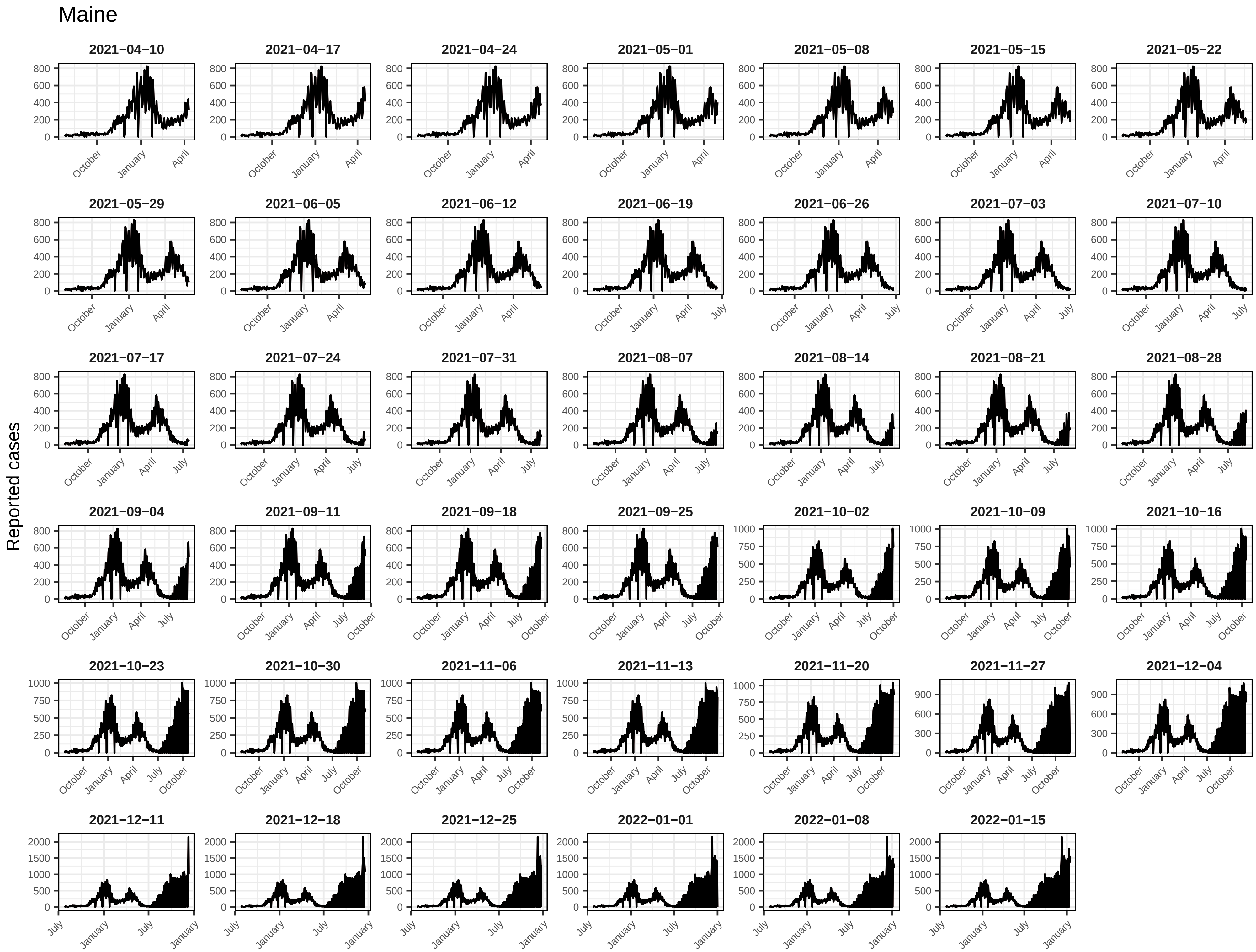
Maine
Reported cases
2021−04−10
2021−04−17
2021−04−24
2021−05−01
2021−05−08
2021−05−15
2021−05−22
2021−05−29
2021−06−05
2021−06−12
2021−06−19
2021−06−26
2021−07−03
2021−07−10
2021−07−17
2021−07−24
2021−07−31
2021−08−07
2021−08−14
2021−08−21
2021−08−28
2021−09−04
2021−09−11
2021−09−18
2021−09−25
2021−10−02
2021−10−09
2021−10−16
2021−10−23
2021−10−30
2021−11−06
2021−11−13
2021−11−20
2021−11−27
2021−12−04
2021−12−11
2021−12−18
2021−12−25
2022−01−01
2022−01−08
2022−01−15

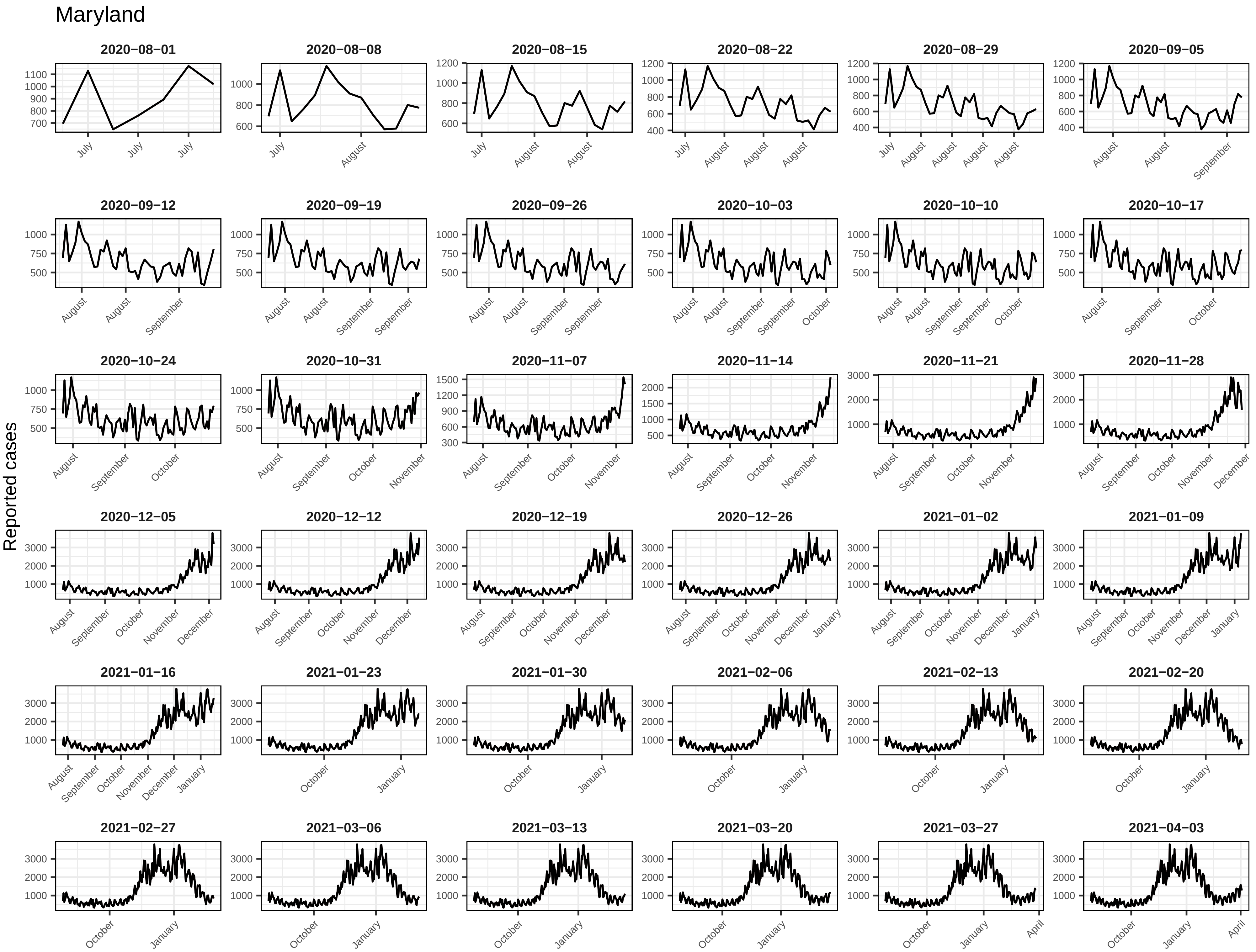
Maryland
Reported cases
2020−08−01
2020−08−08
2020−08−15
2020−08−22
2020−08−29
2020−09−05
2020−09−12
2020−09−19
2020−09−26
2020−10−03
2020−10−10
2020−10−17
2020−10−24
2020−10−31
2020−11−07
2020−11−14
2020−11−21
2020−11−28
2020−12−05
2020−12−12
2020−12−19
2020−12−26
2021−01−02
2021−01−09
2021−01−16
2021−01−23
2021−01−30
2021−02−06
2021−02−13
2021−02−20
2021−02−27
2021−03−06
2021−03−13
2021−03−20
2021−03−27
2021−04−03

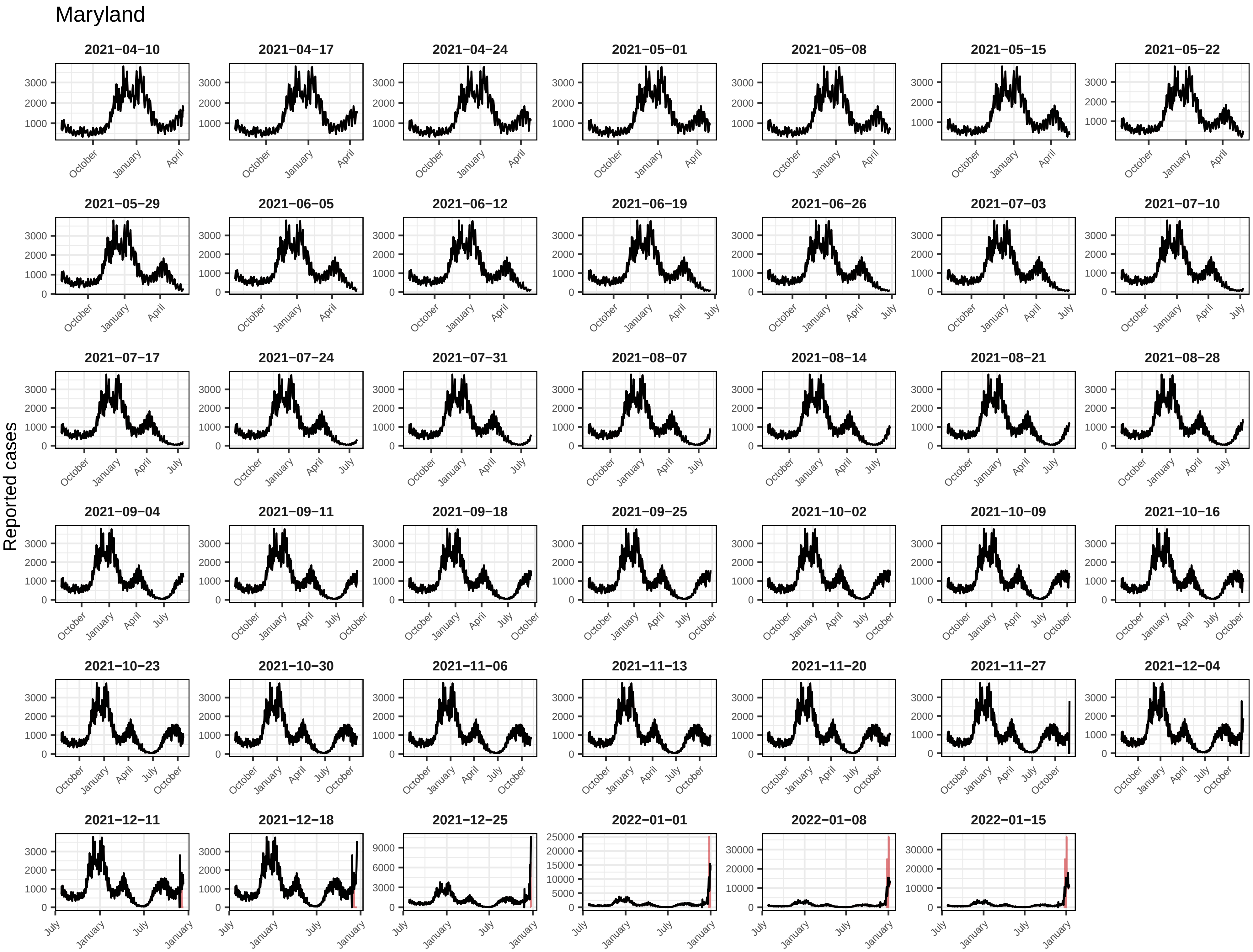
Maryland
Reported cases
2021−04−10
2021−04−17
2021−04−24
2021−05−01
2021−05−08
2021−05−15
2021−05−22
2021−05−29
2021−06−05
2021−06−12
2021−06−19
2021−06−26
2021−07−03
2021−07−10
2021−07−17
2021−07−24
2021−07−31
2021−08−07
2021−08−14
2021−08−21
2021−08−28
2021−09−04
2021−09−11
2021−09−18
2021−09−25
2021−10−02
2021−10−09
2021−10−16
2021−10−23
2021−10−30
2021−11−06
2021−11−13
2021−11−20
2021−11−27
2021−12−04
2021−12−11
2021−12−18
2021−12−25
2022−01−01
2022−01−08
2022−01−15

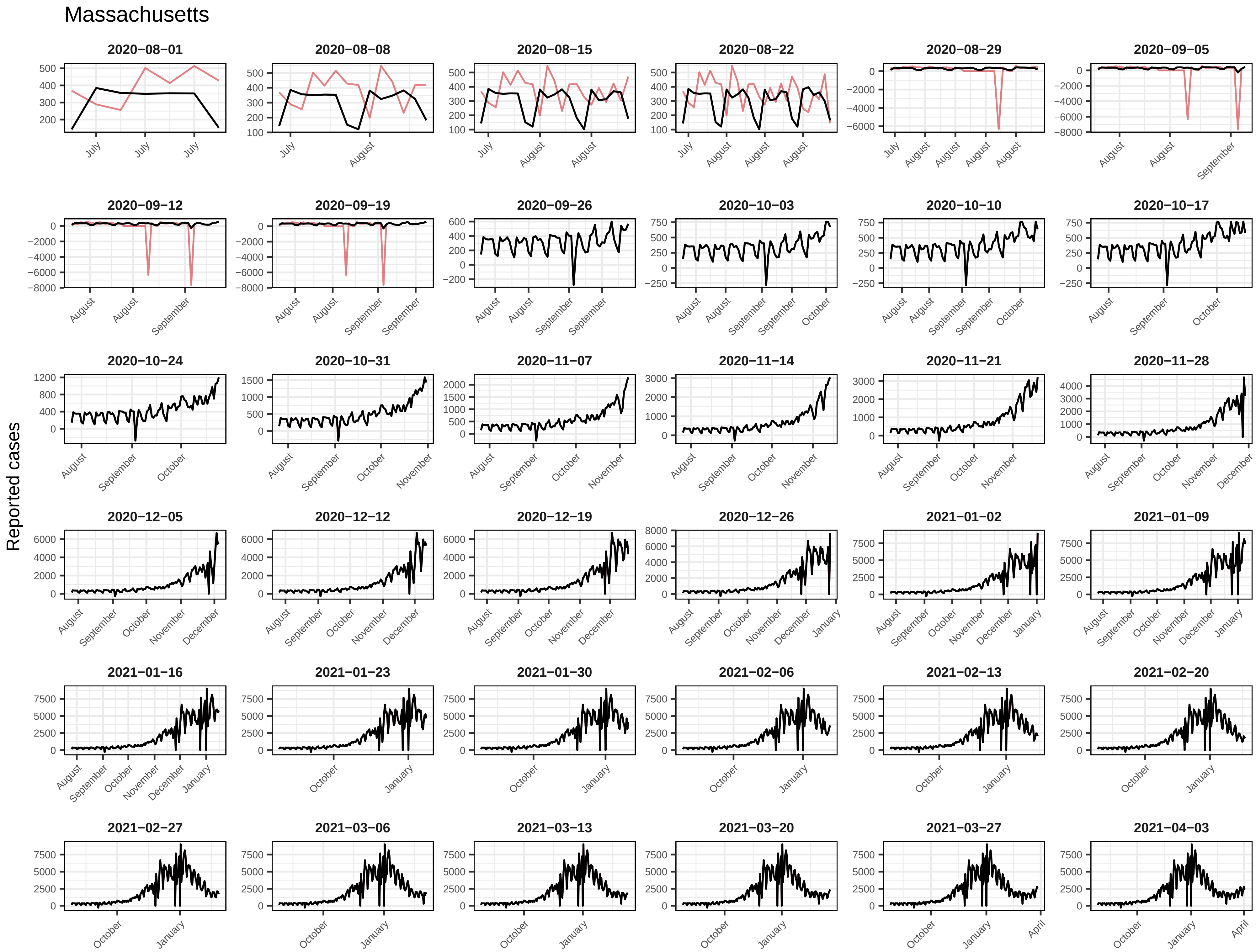
Massachusetts
Reported cases
2020−08−01
2020−08−08
2020−08−15
2020−08−22
2020−08−29
2020−09−05
2020−09−12
2020−09−19
2020−09−26
2020−10−03
2020−10−10
2020−10−17
2020−10−24
2020−10−31
2020−11−07
2020−11−14
2020−11−21
2020−11−28
2020−12−05
2020−12−12
2020−12−19
2020−12−26
2021−01−02
2021−01−09
2021−01−16
2021−01−23
2021−01−30
2021−02−06
2021−02−13
2021−02−20
2021−02−27
2021−03−06
2021−03−13
2021−03−20
2021−03−27
2021−04−03

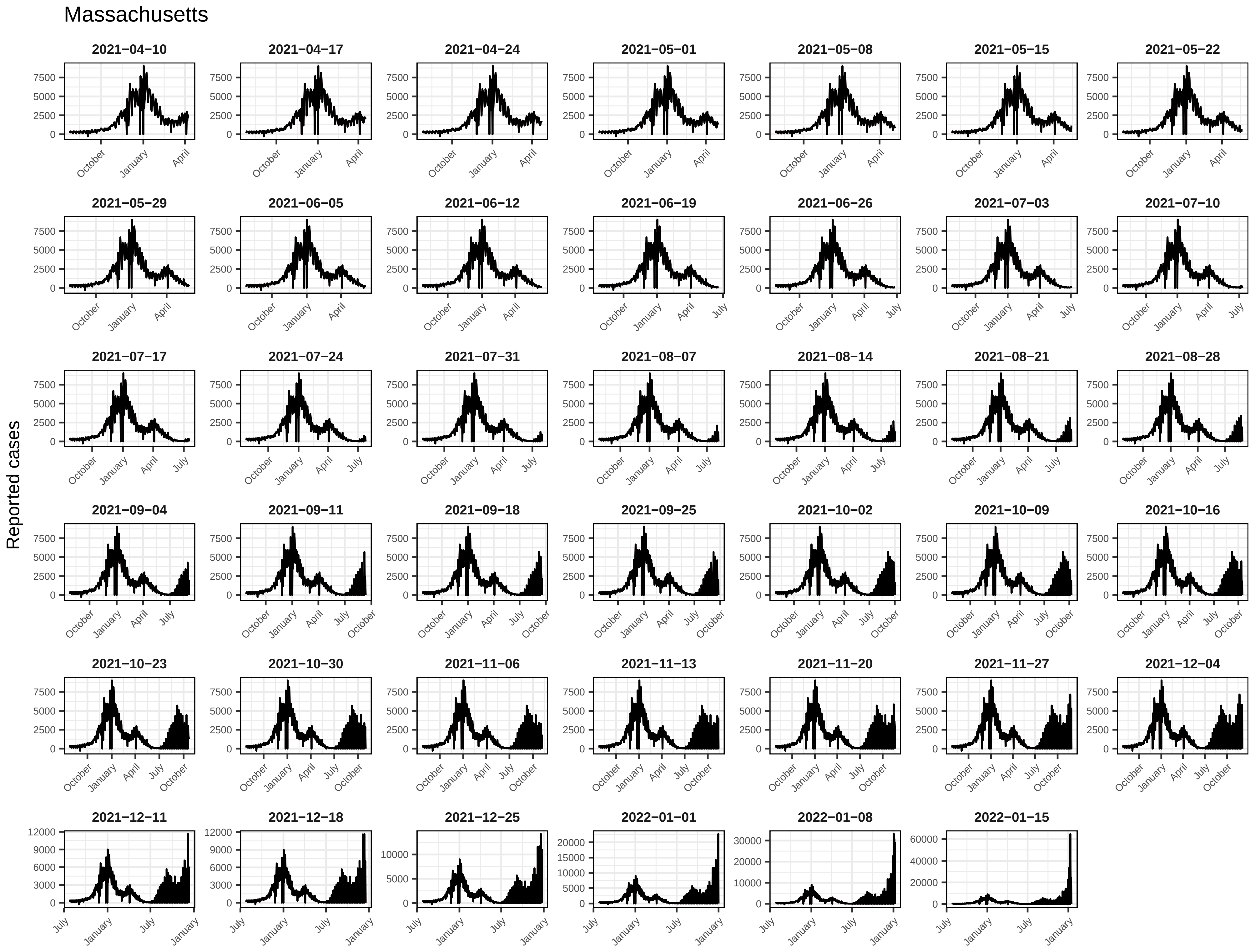
Massachusetts
Reported cases
2021−04−10
2021−04−17
2021−04−24
2021−05−01
2021−05−08
2021−05−15
2021−05−22
2021−05−29
2021−06−05
2021−06−12
2021−06−19
2021−06−26
2021−07−03
2021−07−10
2021−07−17
2021−07−24
2021−07−31
2021−08−07
2021−08−14
2021−08−21
2021−08−28
2021−09−04
2021−09−11
2021−09−18
2021−09−25
2021−10−02
2021−10−09
2021−10−16
2021−10−23
2021−10−30
2021−11−06
2021−11−13
2021−11−20
2021−11−27
2021−12−04
2021−12−11
2021−12−18
2021−12−25
2022−01−01
2022−01−08
2022−01−15

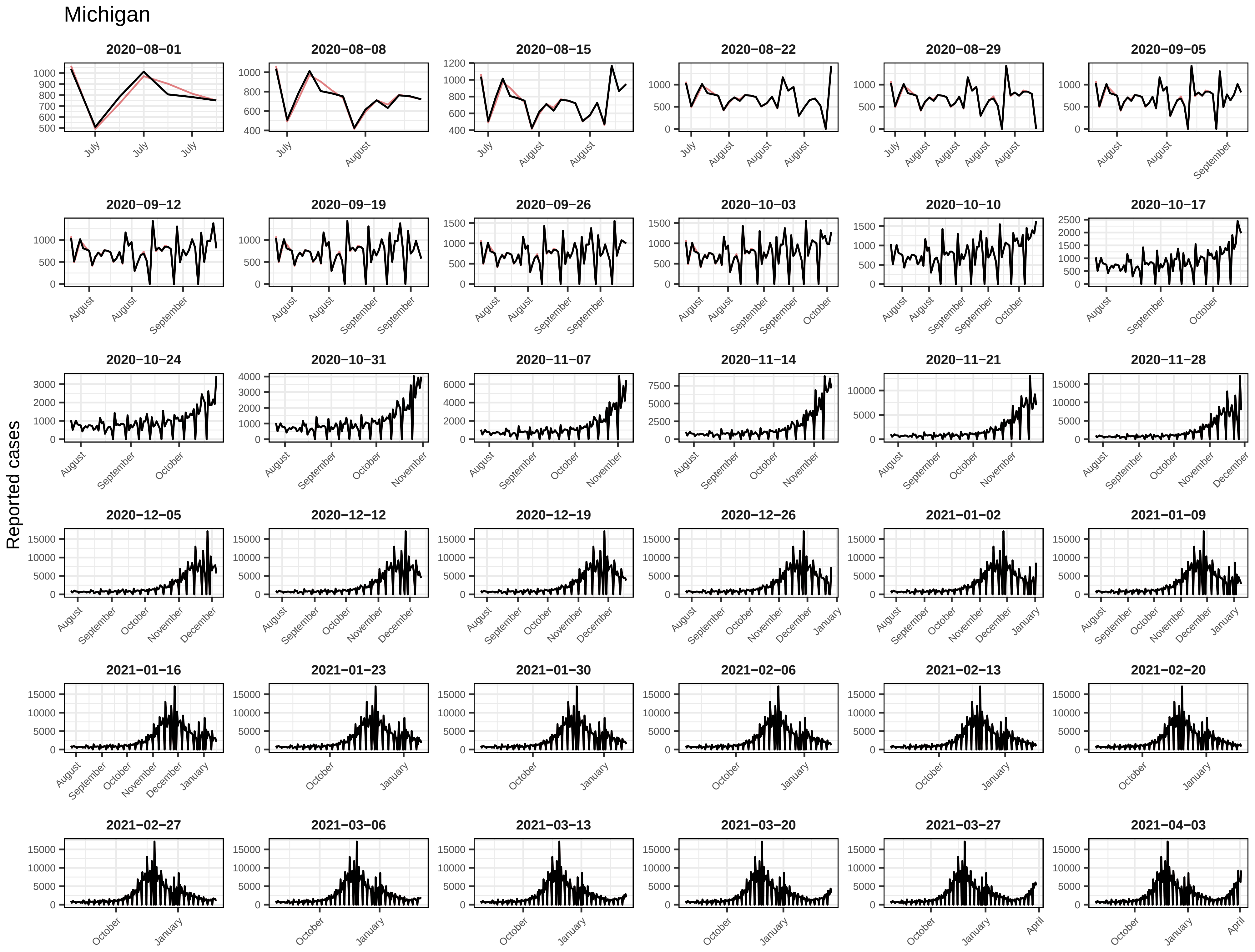
Michigan
Reported cases
2020−08−01
2020−08−08
2020−08−15
2020−08−22
2020−08−29
2020−09−05
2020−09−12
2020−09−19
2020−09−26
2020−10−03
2020−10−10
2020−10−17
2020−10−24
2020−10−31
2020−11−07
2020−11−14
2020−11−21
2020−11−28
2020−12−05
2020−12−12
2020−12−19
2020−12−26
2021−01−02
2021−01−09
2021−01−16
2021−01−23
2021−01−30
2021−02−06
2021−02−13
2021−02−20
2021−02−27
2021−03−06
2021−03−13
2021−03−20
2021−03−27
2021−04−03

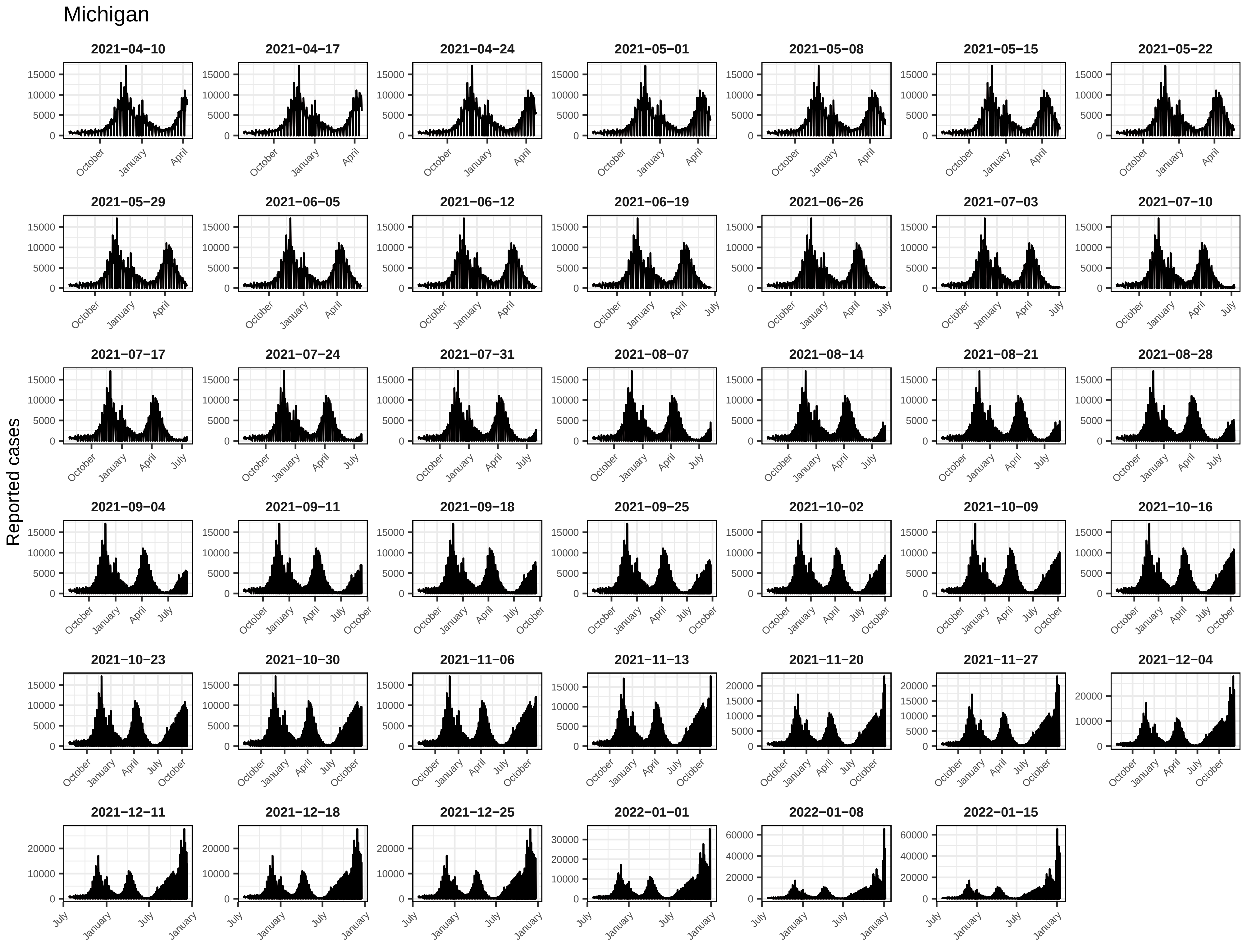
Michigan
Reported cases
2021−04−10
2021−04−17
2021−04−24
2021−05−01
2021−05−08
2021−05−15
2021−05−22
2021−05−29
2021−06−05
2021−06−12
2021−06−19
2021−06−26
2021−07−03
2021−07−10
2021−07−17
2021−07−24
2021−07−31
2021−08−07
2021−08−14
2021−08−21
2021−08−28
2021−09−04
2021−09−11
2021−09−18
2021−09−25
2021−10−02
2021−10−09
2021−10−16
2021−10−23
2021−10−30
2021−11−06
2021−11−13
2021−11−20
2021−11−27
2021−12−04
2021−12−11
2021−12−18
2021−12−25
2022−01−01
2022−01−08
2022−01−15
0
5000
10000
15000
20000
30000
40000
60000
July
October
January
April

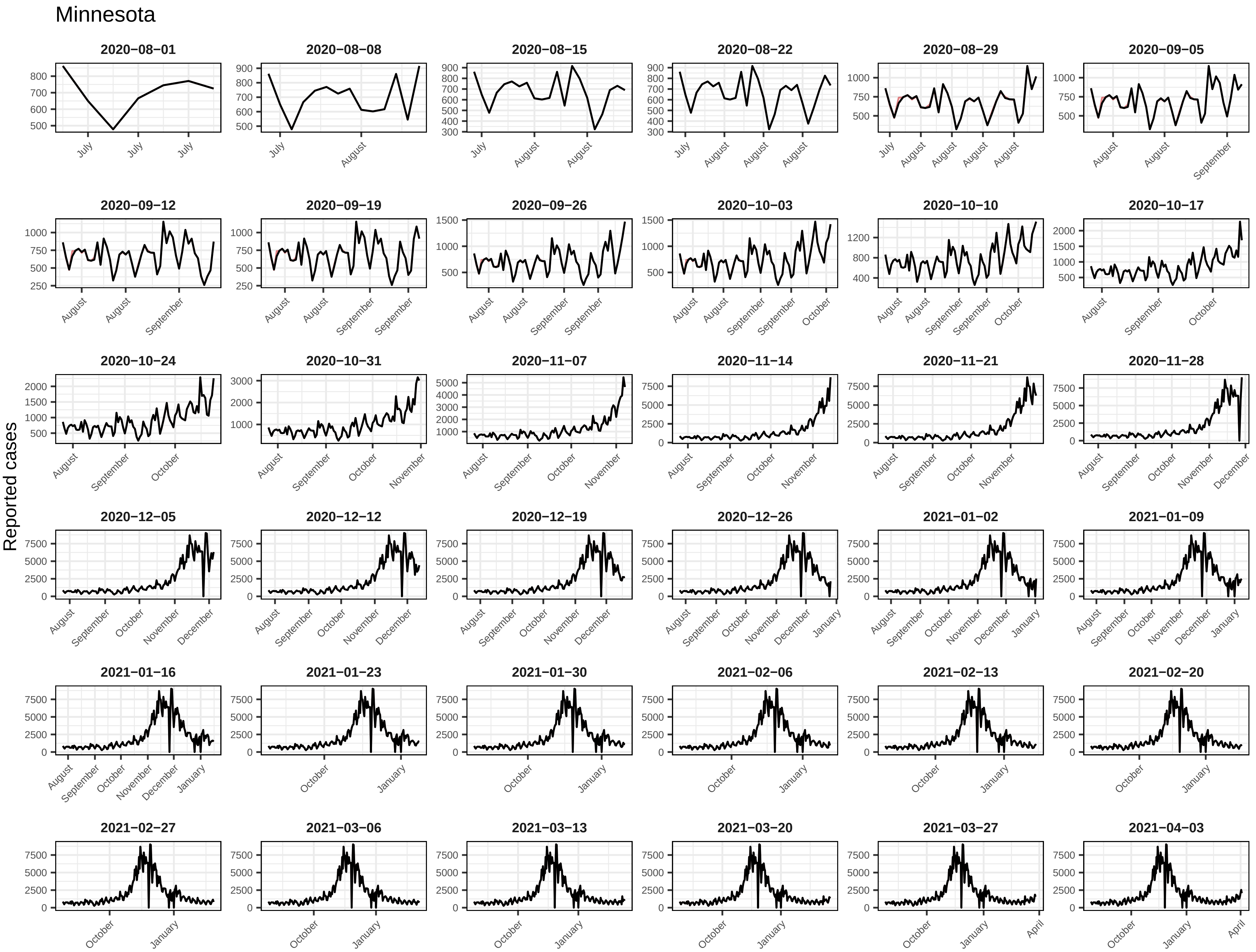
Minnesota
Reported cases
2020−08−01
2020−08−08
2020−08−15
2020−08−22
2020−08−29
2020−09−05
2020−09−12
2020−09−19
2020−09−26
2020−10−03
2020−10−10
2020−10−17
2020−10−24
2020−10−31
2020−11−07
2020−11−14
2020−11−21
2020−11−28
2020−12−05
2020−12−12
2020−12−19
2020−12−26
2021−01−02
2021−01−09
2021−01−16
2021−01−23
2021−01−30
2021−02−06
2021−02−13
2021−02−20
2021−02−27
2021−03−06
2021−03−13
2021−03−20
2021−03−27
2021−04−03

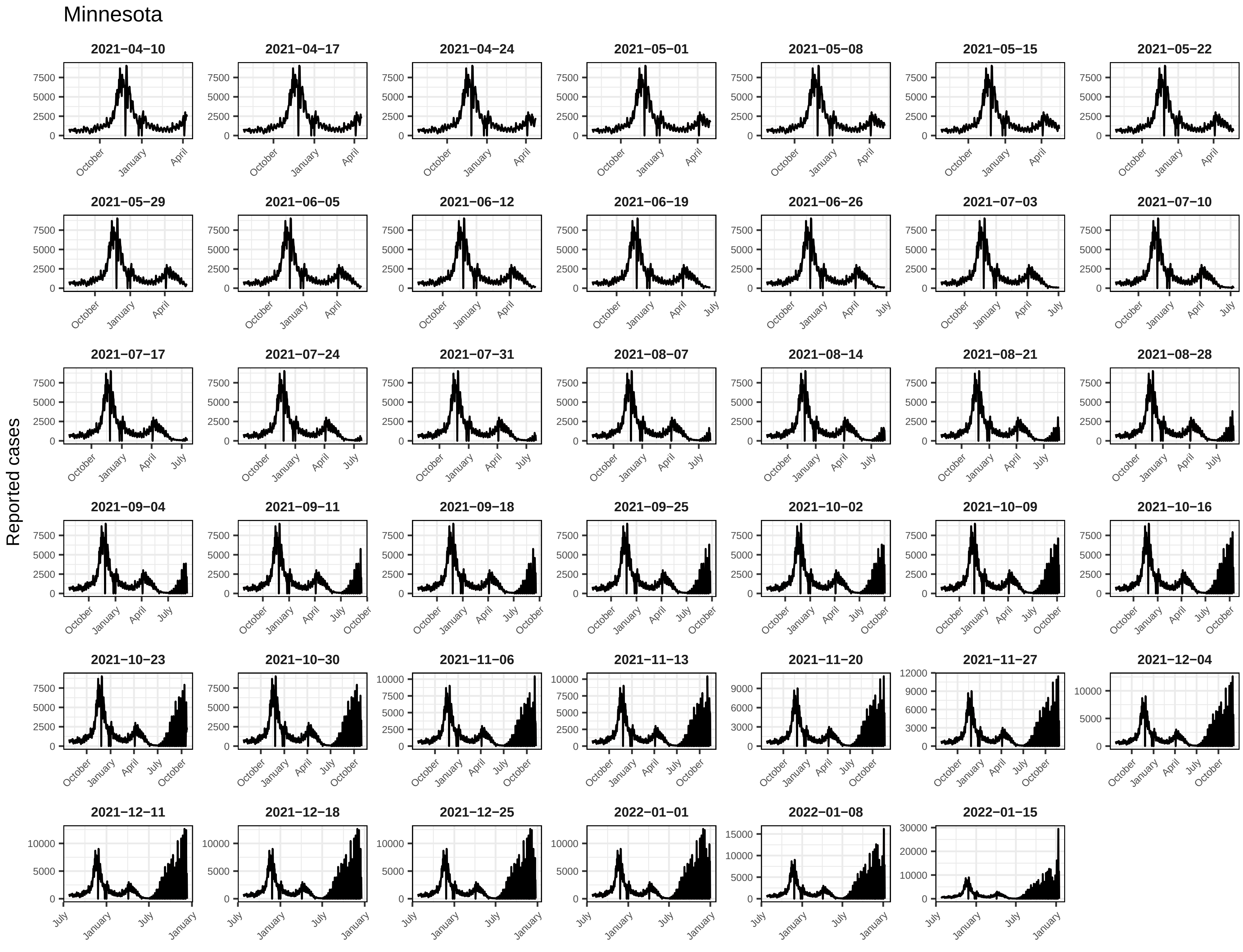
Minnesota
Reported cases
2021−04−10
2021−04−17
2021−04−24
2021−05−01
2021−05−08
2021−05−15
2021−05−22
2021−05−29
2021−06−05
2021−06−12
2021−06−19
2021−06−26
2021−07−03
2021−07−10
2021−07−17
2021−07−24
2021−07−31
2021−08−07
2021−08−14
2021−08−21
2021−08−28
2021−09−04
2021−09−11
2021−09−18
2021−09−25
2021−10−02
2021−10−09
2021−10−16
2021−10−23
2021−10−30
2021−11−06
2021−11−13
2021−11−20
2021−11−27
2021−12−04
2021−12−11
2021−12−18
2021−12−25
2022−01−01
2022−01−08
2022−01−15
October
January
April
July

Mississippi

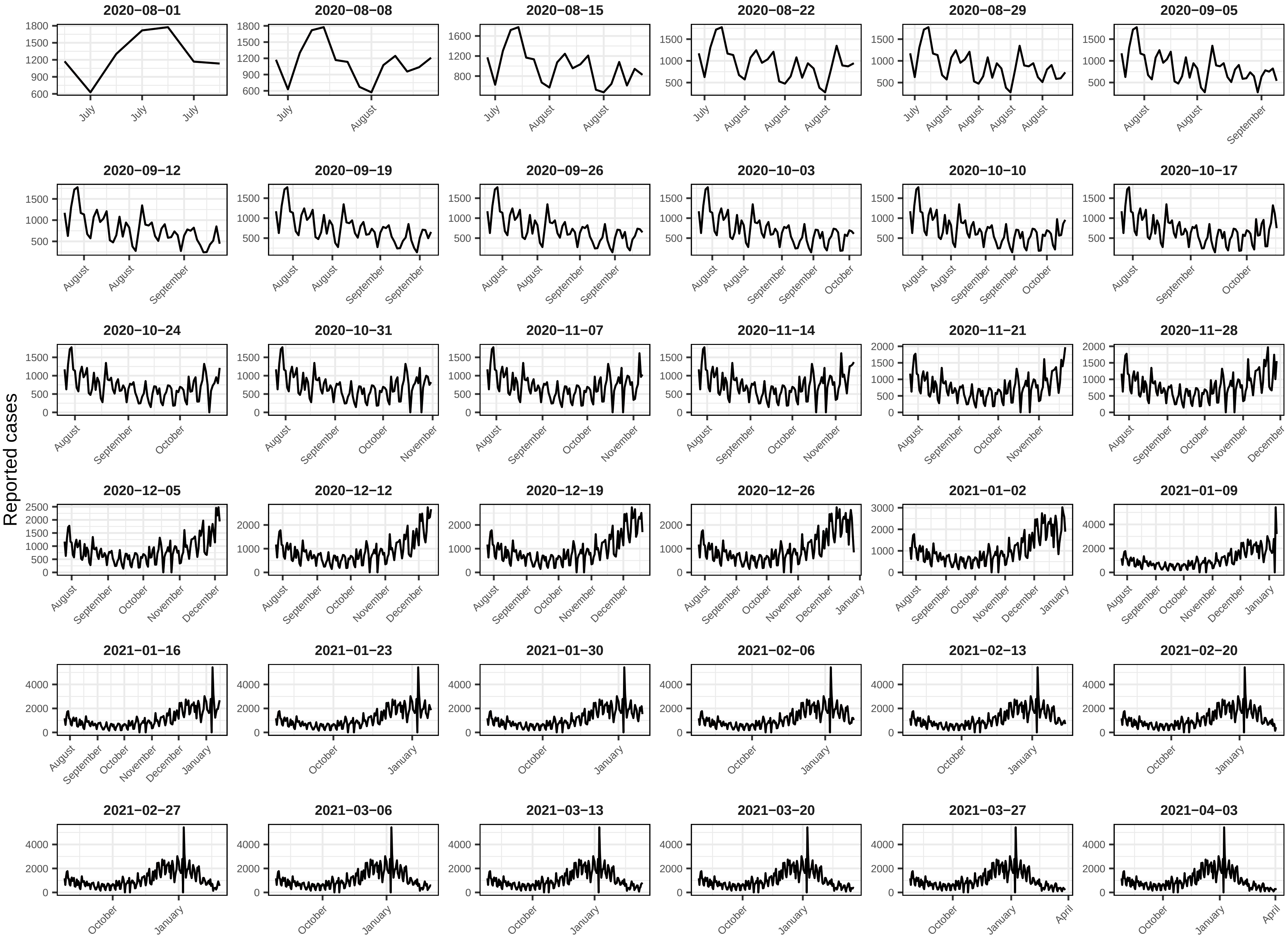

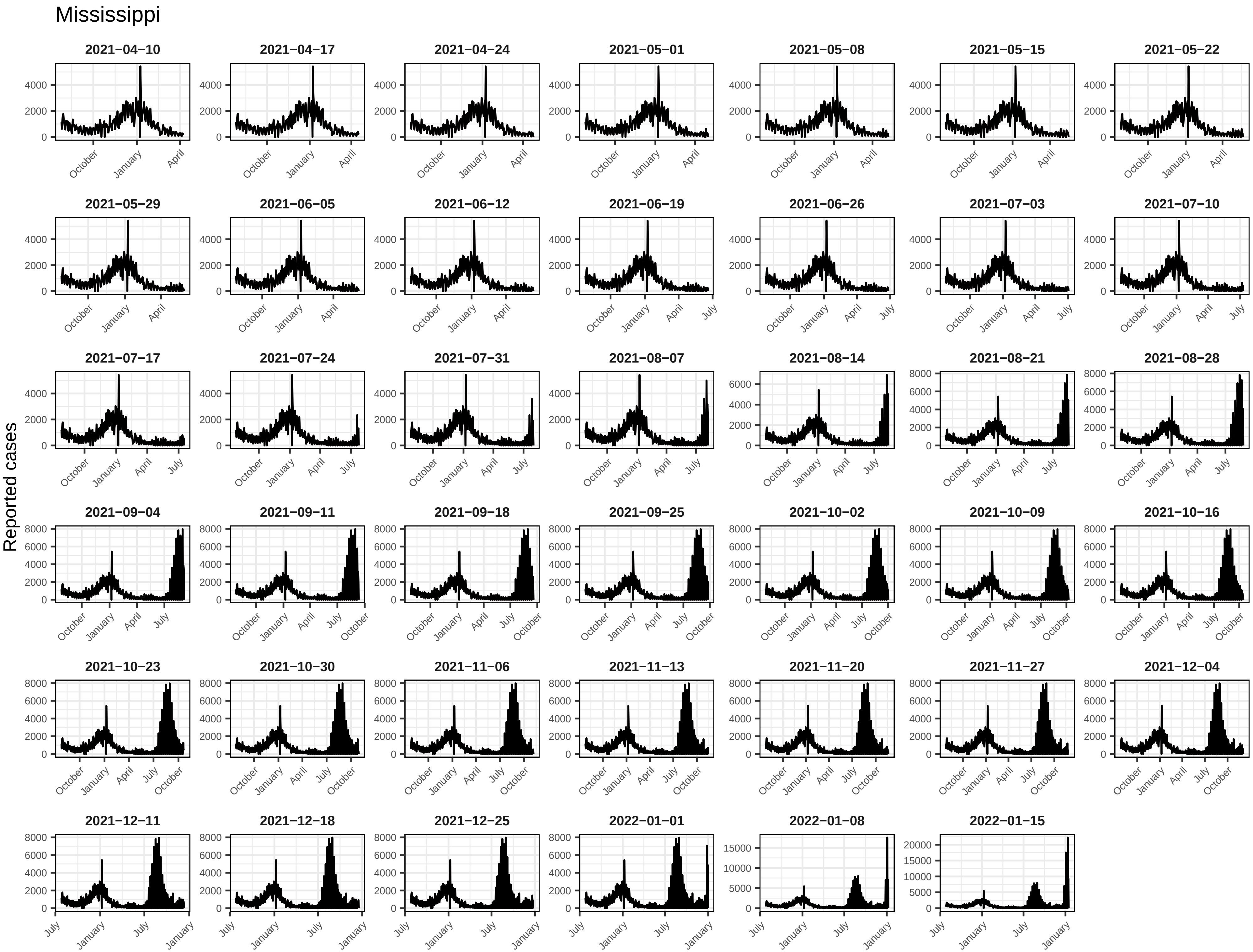
Mississippi
Reported cases
2021−04−10
2021−04−17
2021−04−24
2021−05−01
2021−05−08
2021−05−15
2021−05−22
2021−05−29
2021−06−05
2021−06−12
2021−06−19
2021−06−26
2021−07−03
2021−07−10
2021−07−17
2021−07−24
2021−07−31
2021−08−07
2021−08−14
2021−08−21
2021−08−28
2021−09−04
2021−09−11
2021−09−18
2021−09−25
2021−10−02
2021−10−09
2021−10−16
2021−10−23
2021−10−30
2021−11−06
2021−11−13
2021−11−20
2021−11−27
2021−12−04
2021−12−11
2021−12−18
2021−12−25
2022−01−01
2022−01−08
2022−01−15
0
2000
4000
6000
8000
5000
10000
15000
20000
July
October
January
April

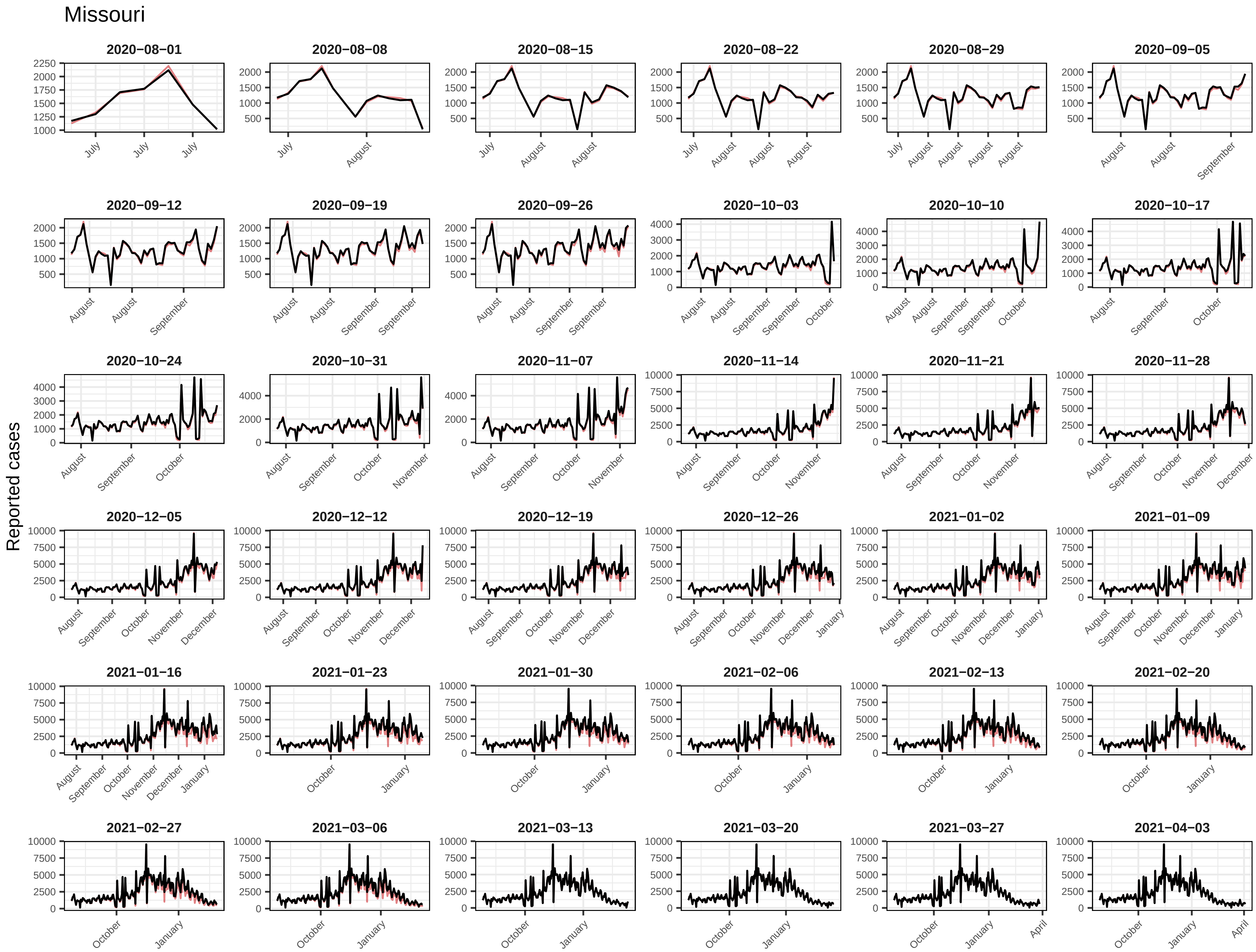
Missouri
Reported cases
2020−08−01
2020−08−08
2020−08−15
2020−08−22
2020−08−29
2020−09−05
2020−09−12
2020−09−19
2020−09−26
2020−10−03
2020−10−10
2020−10−17
2020−10−24
2020−10−31
2020−11−07
2020−11−14
2020−11−21
2020−11−28
2020−12−05
2020−12−12
2020−12−19
2020−12−26
2021−01−02
2021−01−09
2021−01−16
2021−01−23
2021−01−30
2021−02−06
2021−02−13
2021−02−20
2021−02−27
2021−03−06
2021−03−13
2021−03−20
2021−03−27
2021−04−03

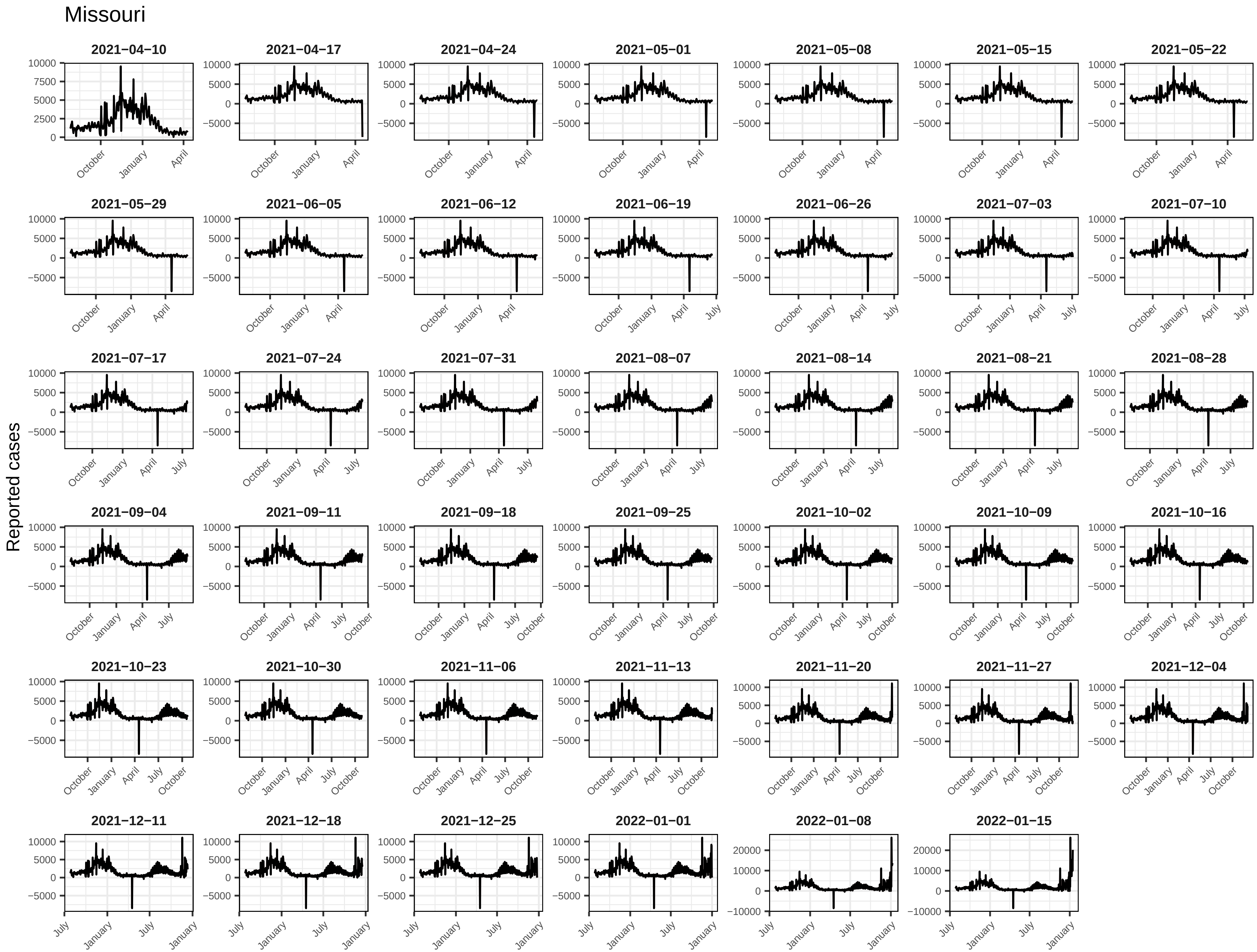

Missouri
2021−04−10
2021−04−17
2021−04−24
2021−05−01
2021−05−08
2021−05−15
2021−05−22
2021−05−29
2021−06−05
2021−06−12
2021−06−19
2021−06−26
2021−07−03
2021−07−10
2021−07−17
2021−07−24
2021−07−31
2021−08−07
2021−08−14
2021−08−21
2021−08−28
2021−09−04
2021−09−11
2021−09−18
2021−09−25
2021−10−02
2021−10−09
2021−10−16
2021−10−23
2021−10−30
2021−11−06
2021−11−13
2021−11−20
2021−11−27
2021−12−04
2021−12−11
2021−12−18
2021−12−25
2022−01−01
2022−01−08
2022−01−15
Reported cases
10000
7500
5000
2500
0
−5000
20000
−10000
October
January
April
July

Montana

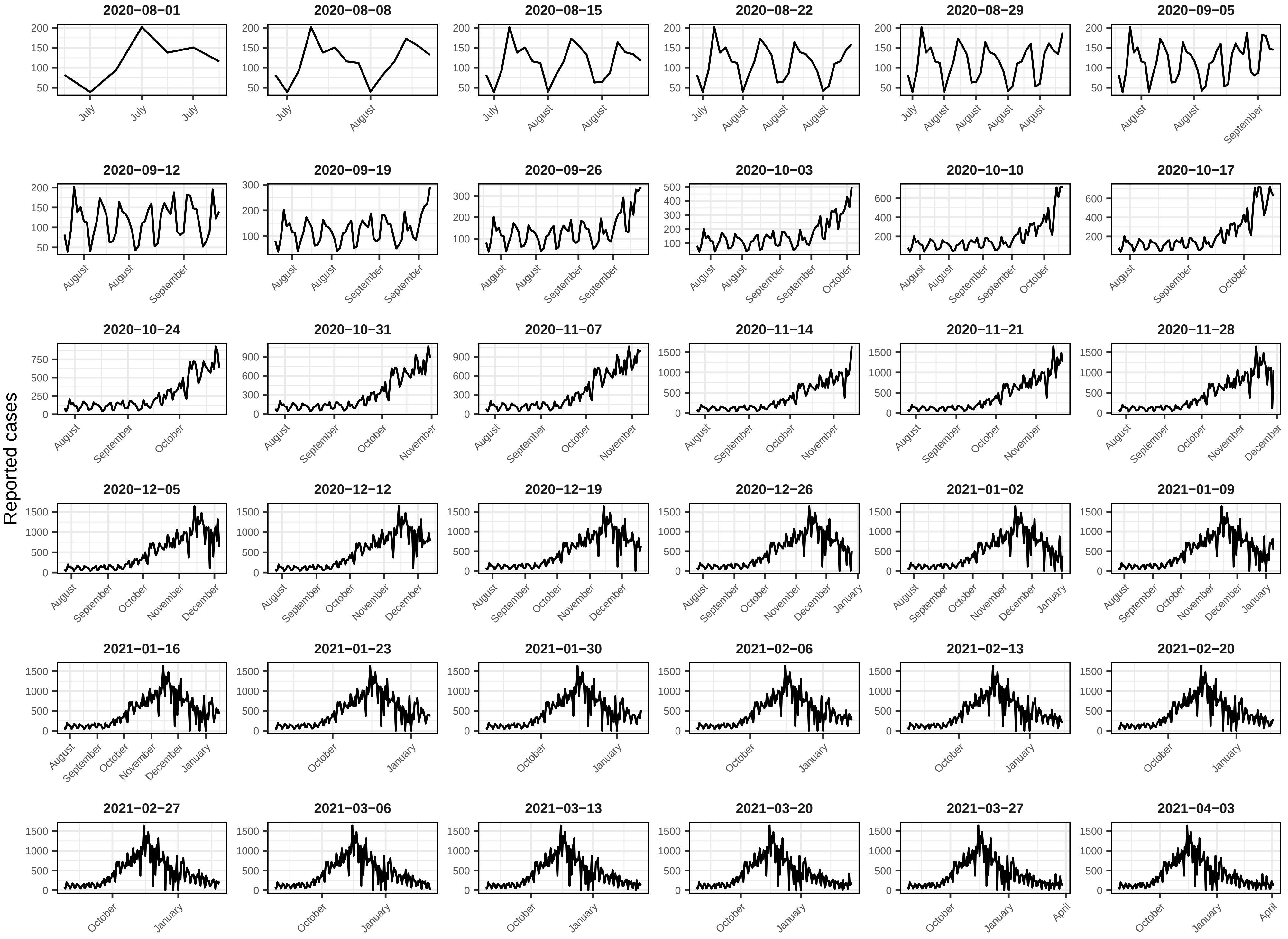

Reported cases
2020−08−01
2020−08−08
2020−08−15
2020−08−22
2020−08−29
2020−09−05
2020−09−12
2020−09−19
2020−09−26
2020−10−03
2020−10−10
2020−10−17
2020−10−24
2020−10−31
2020−11−07
2020−11−14
2020−11−21
2020−11−28
2020−12−05
2020−12−12
2020−12−19
2020−12−26
2021−01−02
2021−01−09
2021−01−16
2021−01−23
2021−01−30
2021−02−06
2021−02−13
2021−02−20
2021−02−27
2021−03−06
2021−03−13
2021−03−20
2021−03−27
2021−04−03

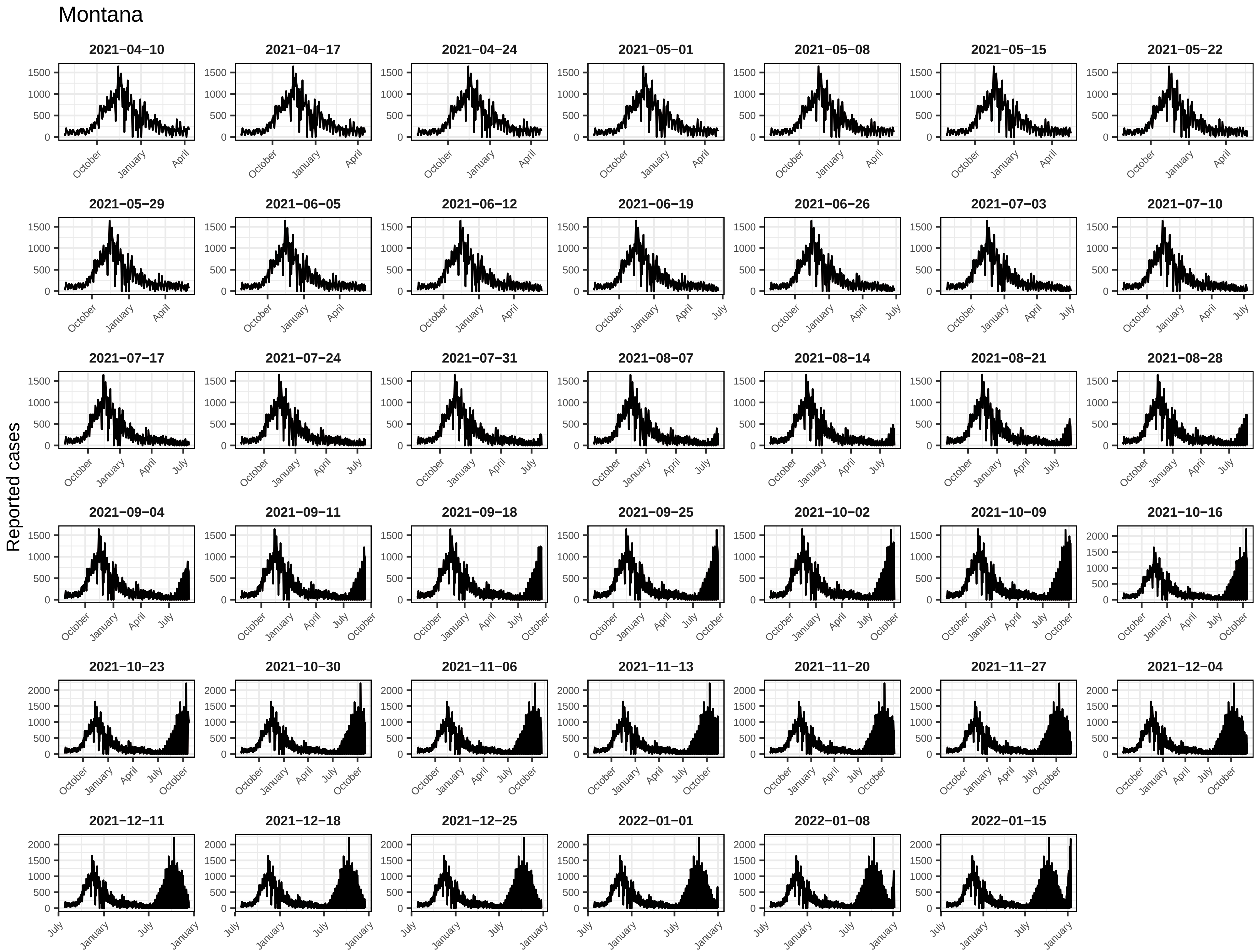
Montana
Reported cases
2021−04−10
2021−04−17
2021−04−24
2021−05−01
2021−05−08
2021−05−15
2021−05−22
2021−05−29
2021−06−05
2021−06−12
2021−06−19
2021−06−26
2021−07−03
2021−07−10
2021−07−17
2021−07−24
2021−07−31
2021−08−07
2021−08−14
2021−08−21
2021−08−28
2021−09−04
2021−09−11
2021−09−18
2021−09−25
2021−10−02
2021−10−09
2021−10−16
2021−10−23
2021−10−30
2021−11−06
2021−11−13
2021−11−20
2021−11−27
2021−12−04
2021−12−11
2021−12−18
2021−12−25
2022−01−01
2022−01−08
2022−01−15
October
January
April
July

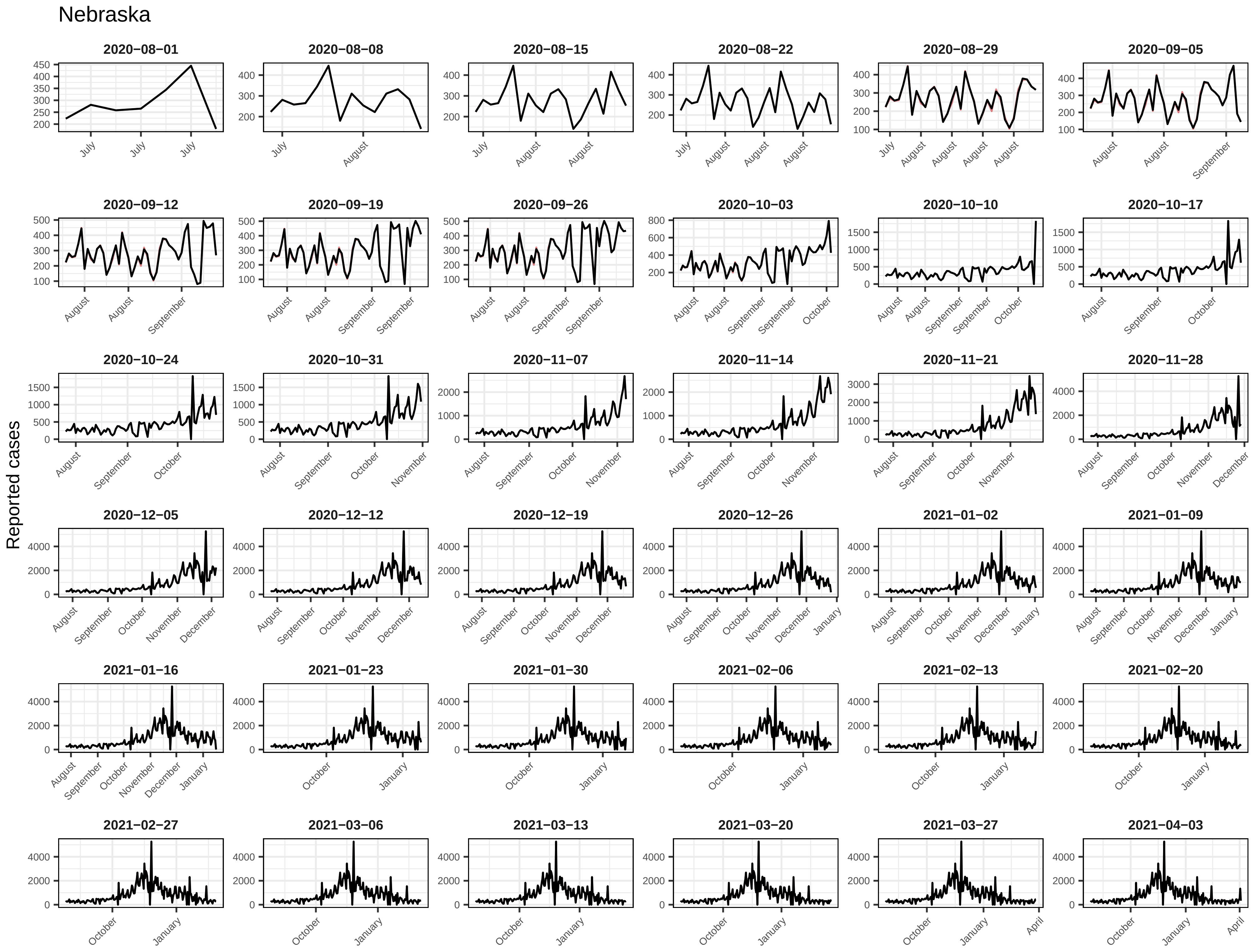

Nebraska
Reported cases
2020−08−01
2020−08−08
2020−08−15
2020−08−22
2020−08−29
2020−09−05
2020−09−12
2020−09−19
2020−09−26
2020−10−03
2020−10−10
2020−10−17
2020−10−24
2020−10−31
2020−11−07
2020−11−14
2020−11−21
2020−11−28
2020−12−05
2020−12−12
2020−12−19
2020−12−26
2021−01−02
2021−01−09
2021−01−16
2021−01−23
2021−01−30
2021−02−06
2021−02−13
2021−02−20
2021−02−27
2021−03−06
2021−03−13
2021−03−20
2021−03−27
2021−04−03

Nebraska

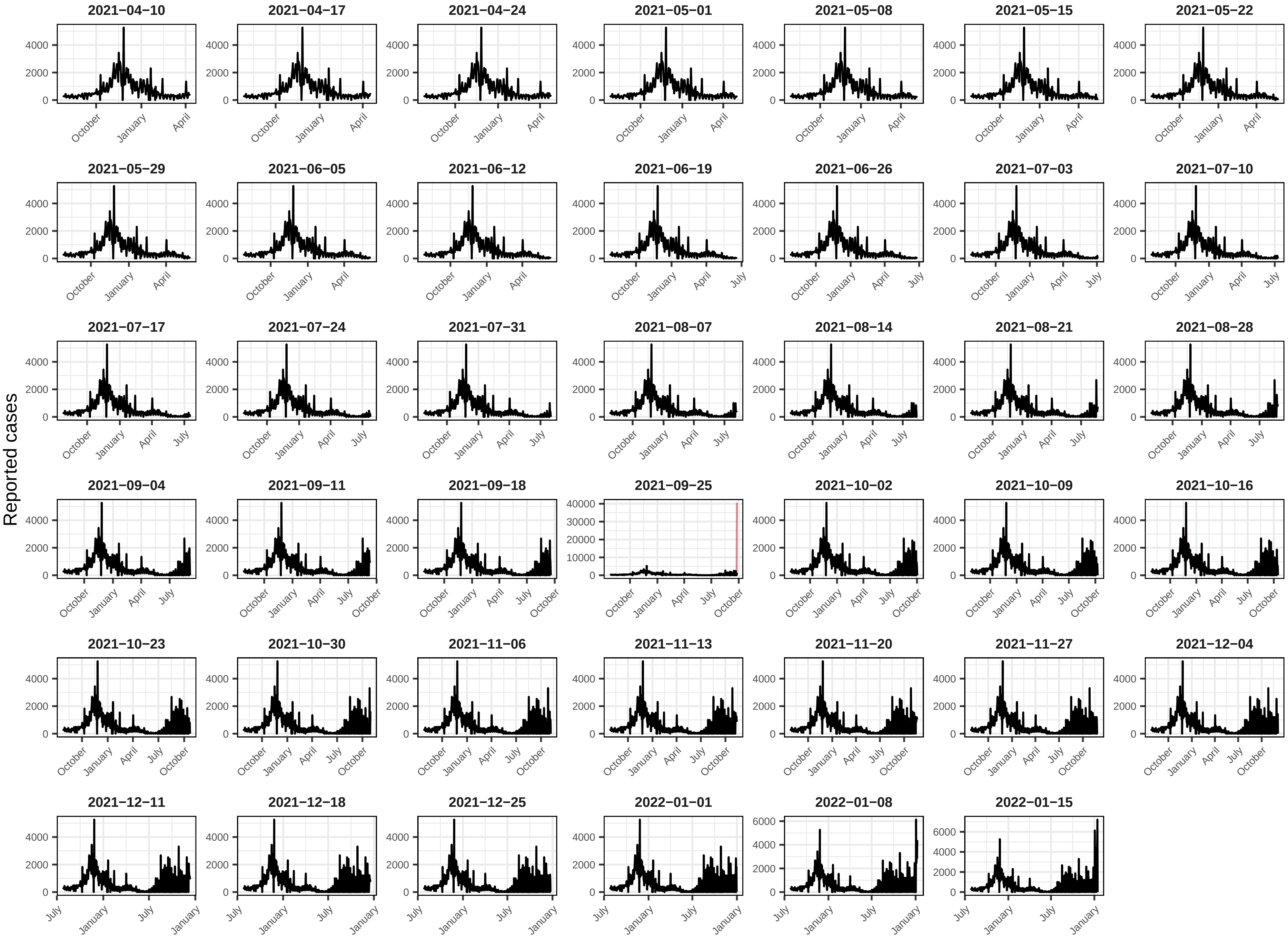
2021−04−10
2021−04−17
2021−04−24
2021−05−01
2021−05−08
2021−05−15
2021−05−22
2021−05−29
2021−06−05
2021−06−12
2021−06−19
2021−06−26
2021−07−03
2021−07−10
2021−07−17
2021−07−24
2021−07−31
2021−08−07
2021−08−14
2021−08−21
2021−08−28
2021−09−04
2021−09−11
2021−09−18
2021−09−25
2021−10−02
2021−10−09
2021−10−16
2021−10−23
2021−10−30
2021−11−06
2021−11−13
2021−11−20
2021−11−27
2021−12−04
2021−12−11
2021−12−18
2021−12−25
2022−01−01
2022−01−08
2022−01−15
Reported cases
0
2000
4000
6000
10000
20000
30000
40000
October
January
April
July

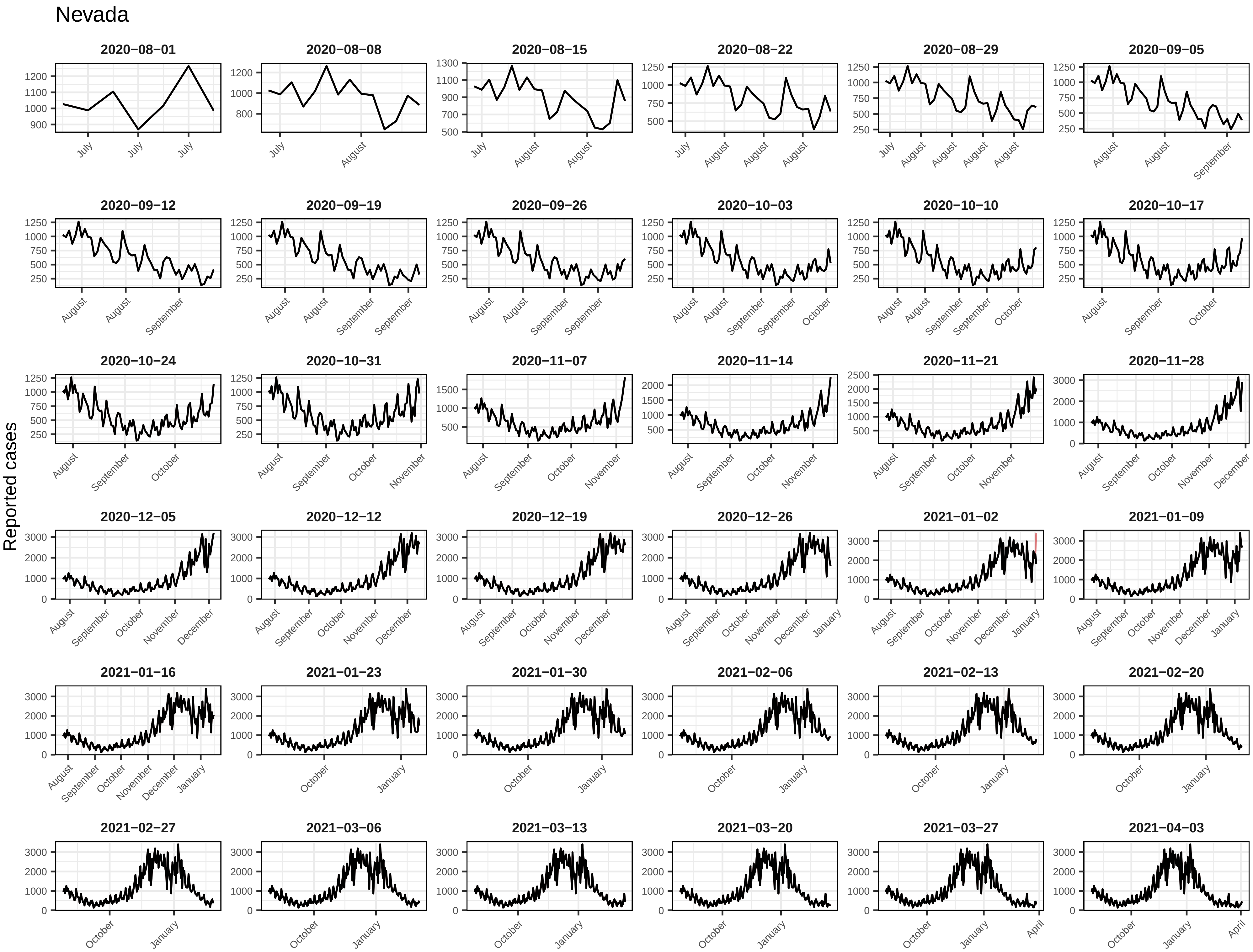

Nevada
Reported cases
2020−08−01
2020−08−08
2020−08−15
2020−08−22
2020−08−29
2020−09−05
2020−09−12
2020−09−19
2020−09−26
2020−10−03
2020−10−10
2020−10−17
2020−10−24
2020−10−31
2020−11−07
2020−11−14
2020−11−21
2020−11−28
2020−12−05
2020−12−12
2020−12−19
2020−12−26
2021−01−02
2021−01−09
2021−01−16
2021−01−23
2021−01−30
2021−02−06
2021−02−13
2021−02−20
2021−02−27
2021−03−06
2021−03−13
2021−03−20
2021−03−27
2021−04−03

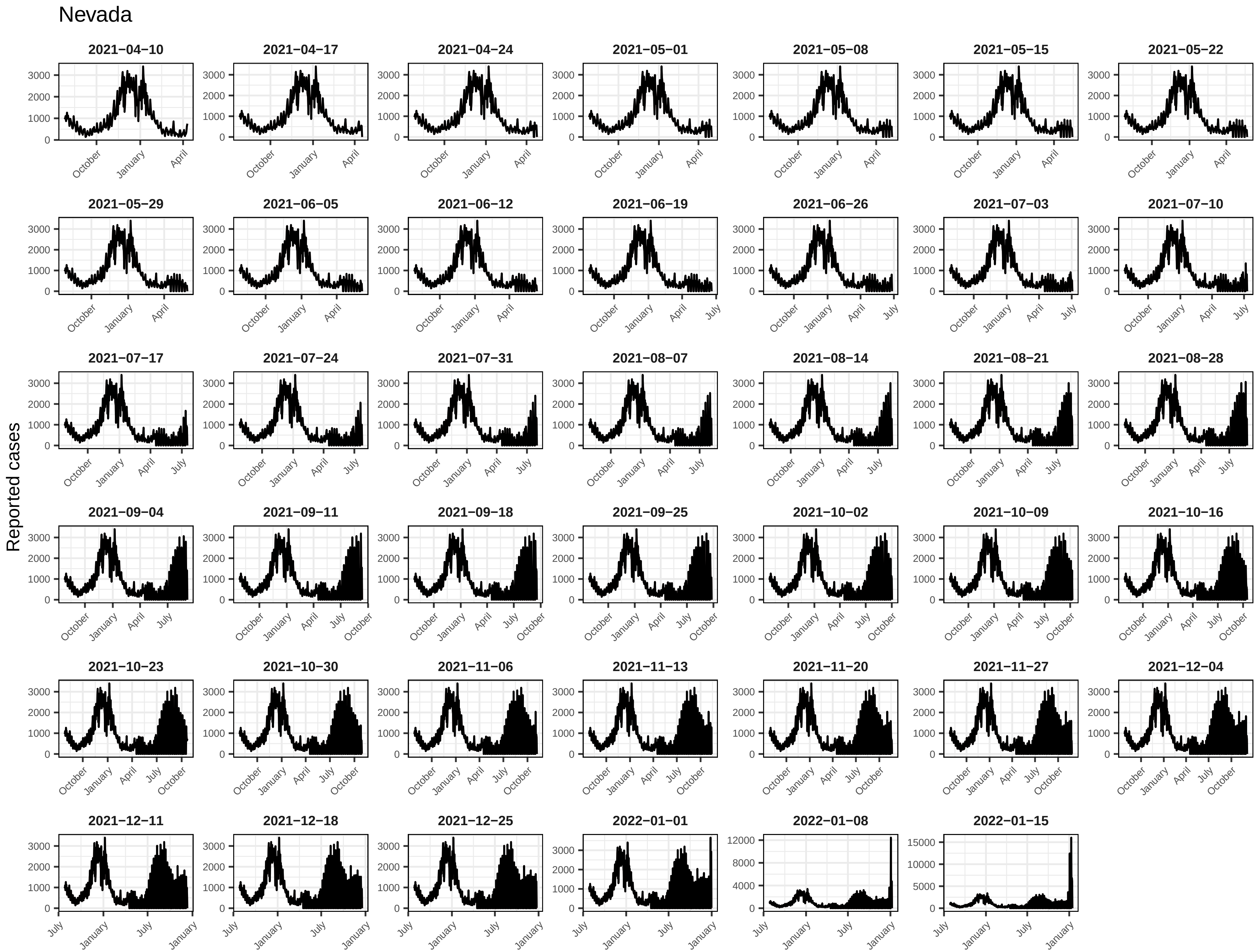
Nevada
Reported cases
2021−04−10
2021−04−17
2021−04−24
2021−05−01
2021−05−08
2021−05−15
2021−05−22
2021−05−29
2021−06−05
2021−06−12
2021−06−19
2021−06−26
2021−07−03
2021−07−10
2021−07−17
2021−07−24
2021−07−31
2021−08−07
2021−08−14
2021−08−21
2021−08−28
2021−09−04
2021−09−11
2021−09−18
2021−09−25
2021−10−02
2021−10−09
2021−10−16
2021−10−23
2021−10−30
2021−11−06
2021−11−13
2021−11−20
2021−11−27
2021−12−04
2021−12−11
2021−12−18
2021−12−25
2022−01−01
2022−01−08
2022−01−15

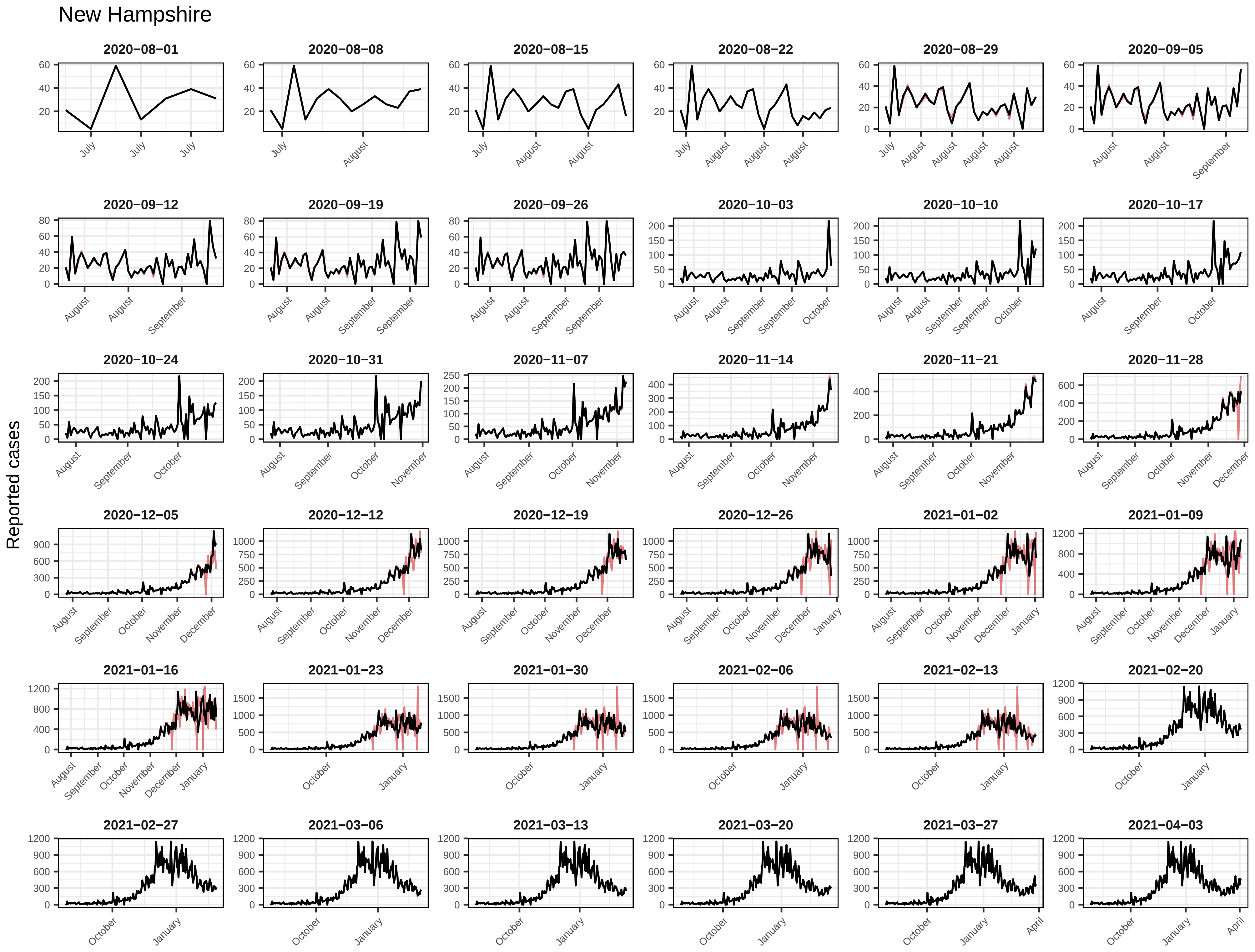
New Hampshire
Reported cases
2020−08−01
2020−08−08
2020−08−15
2020−08−22
2020−08−29
2020−09−05
2020−09−12
2020−09−19
2020−09−26
2020−10−03
2020−10−10
2020−10−17
2020−10−24
2020−10−31
2020−11−07
2020−11−14
2020−11−21
2020−11−28
2020−12−05
2020−12−12
2020−12−19
2020−12−26
2021−01−02
2021−01−09
2021−01−16
2021−01−23
2021−01−30
2021−02−06
2021−02−13
2021−02−20
2021−02−27
2021−03−06
2021−03−13
2021−03−20
2021−03−27
2021−04−03

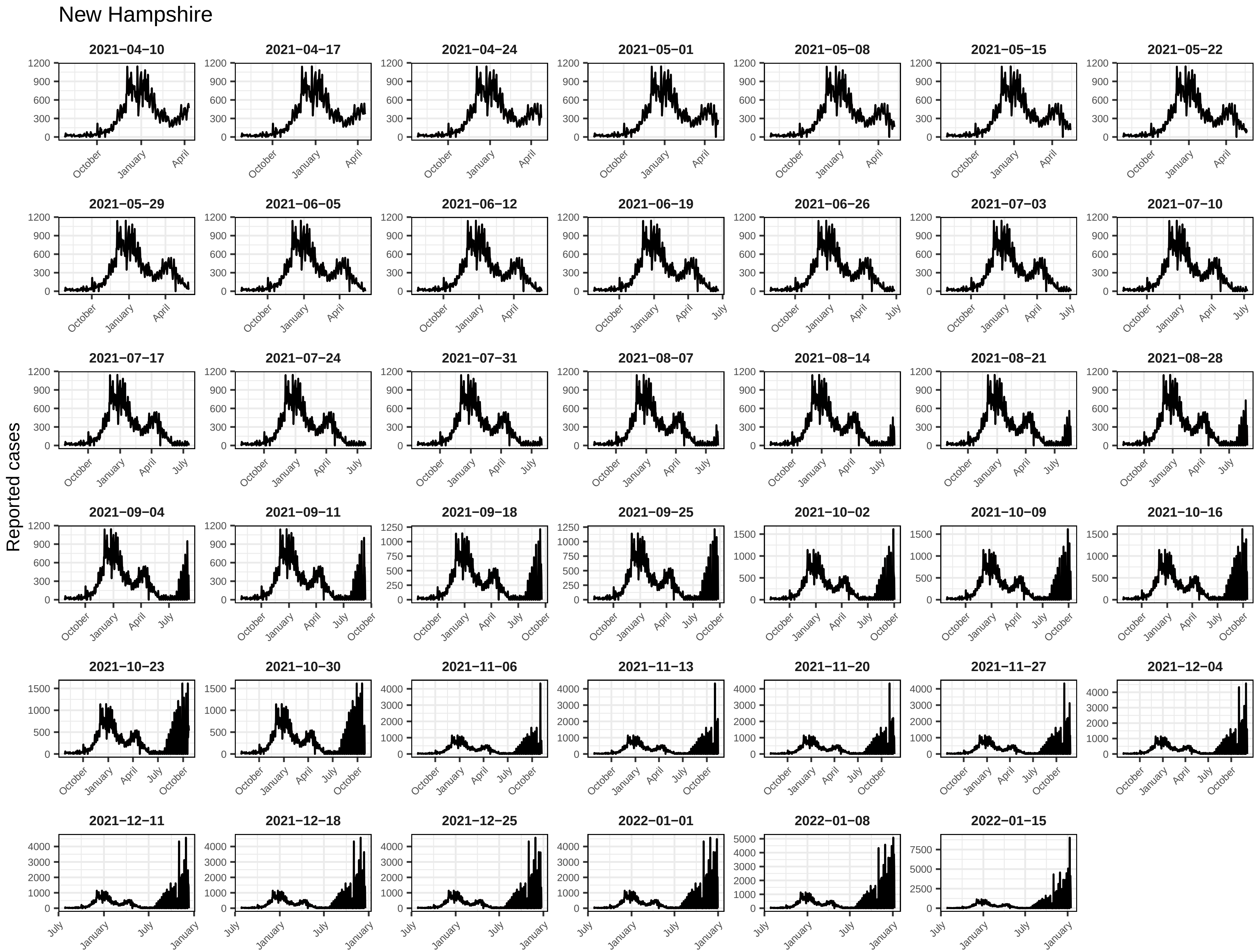
New Hampshire
Reported cases
2021−04−10
2021−04−17
2021−04−24
2021−05−01
2021−05−08
2021−05−15
2021−05−22
2021−05−29
2021−06−05
2021−06−12
2021−06−19
2021−06−26
2021−07−03
2021−07−10
2021−07−17
2021−07−24
2021−07−31
2021−08−07
2021−08−14
2021−08−21
2021−08−28
2021−09−04
2021−09−11
2021−09−18
2021−09−25
2021−10−02
2021−10−09
2021−10−16
2021−10−23
2021−10−30
2021−11−06
2021−11−13
2021−11−20
2021−11−27
2021−12−04
2021−12−11
2021−12−18
2021−12−25
2022−01−01
2022−01−08
2022−01−15
October
January
April
July

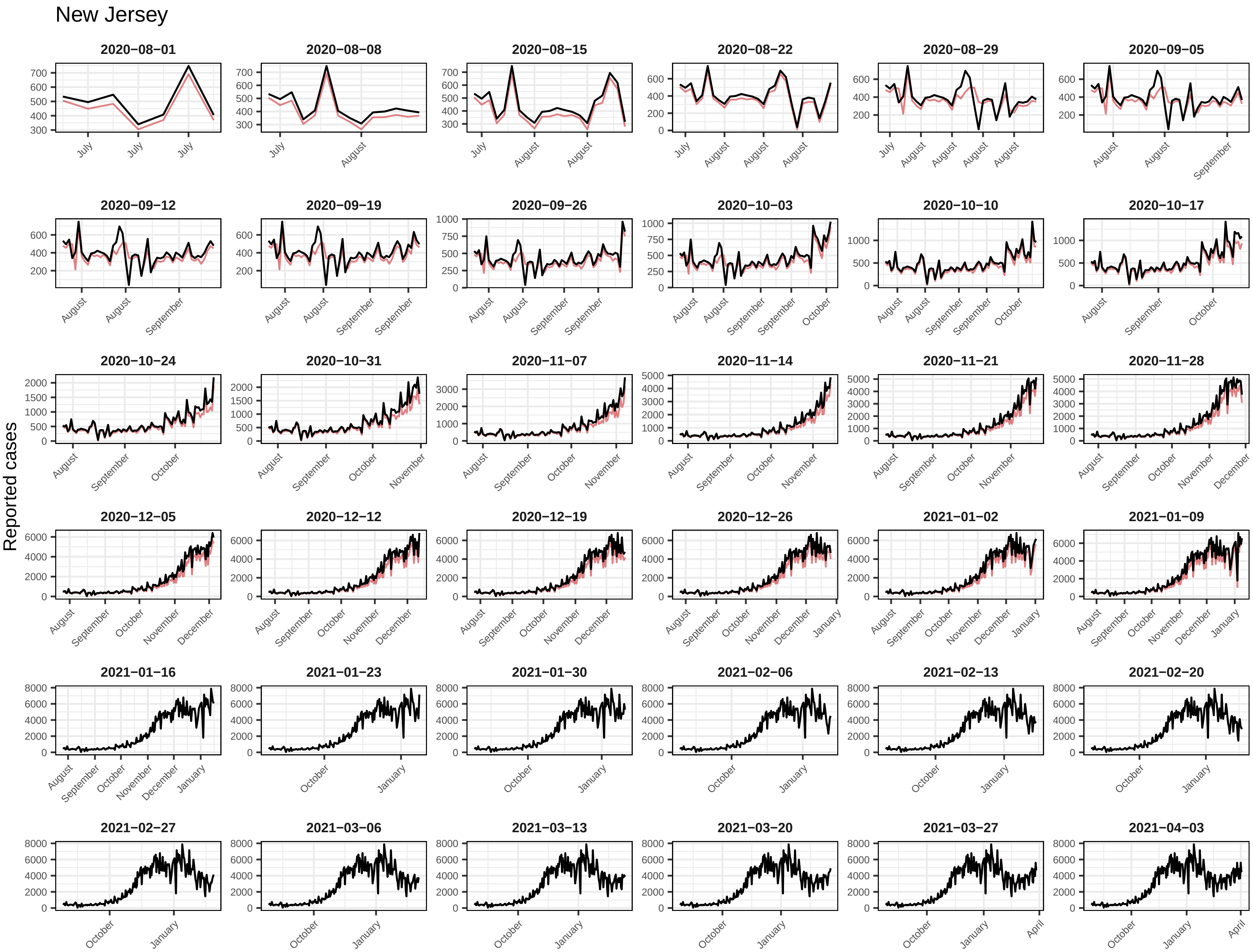

New Jersey
Reported cases
2020−08−01
2020−08−08
2020−08−15
2020−08−22
2020−08−29
2020−09−05
2020−09−12
2020−09−19
2020−09−26
2020−10−03
2020−10−10
2020−10−17
2020−10−24
2020−10−31
2020−11−07
2020−11−14
2020−11−21
2020−11−28
2020−12−05
2020−12−12
2020−12−19
2020−12−26
2021−01−02
2021−01−09
2021−01−16
2021−01−23
2021−01−30
2021−02−06
2021−02−13
2021−02−20
2021−02−27
2021−03−06
2021−03−13
2021−03−20
2021−03−27
2021−04−03

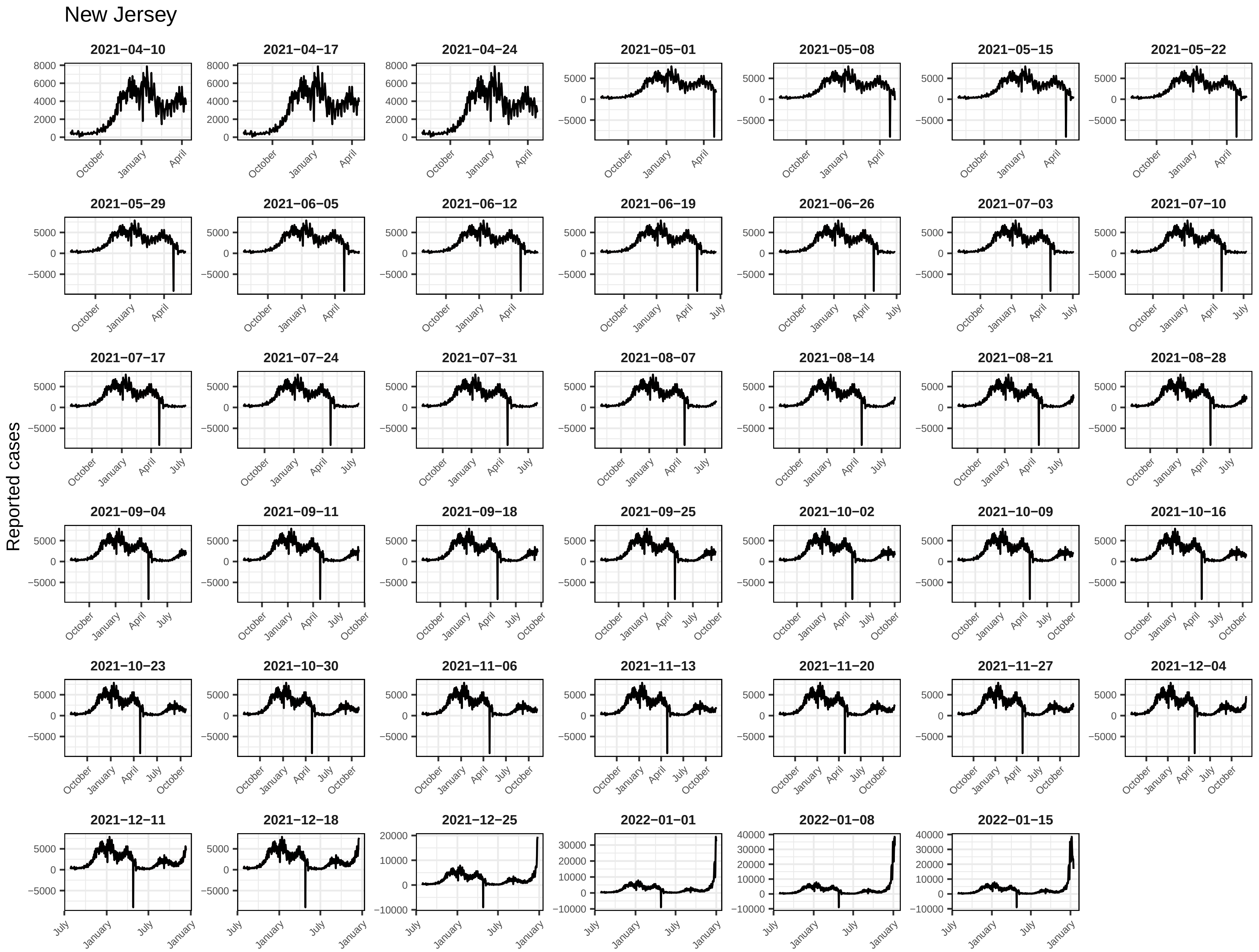
New Jersey
Reported cases
2021−04−10
2021−04−17
2021−04−24
2021−05−01
2021−05−08
2021−05−15
2021−05−22
2021−05−29
2021−06−05
2021−06−12
2021−06−19
2021−06−26
2021−07−03
2021−07−10
2021−07−17
2021−07−24
2021−07−31
2021−08−07
2021−08−14
2021−08−21
2021−08−28
2021−09−04
2021−09−11
2021−09−18
2021−09−25
2021−10−02
2021−10−09
2021−10−16
2021−10−23
2021−10−30
2021−11−06
2021−11−13
2021−11−20
2021−11−27
2021−12−04
2021−12−11
2021−12−18
2021−12−25
2022−01−01
2022−01−08
2022−01−15
0
2000
4000
6000
8000
−5000
5000
−10000
10000
20000
30000
40000
July
October
January
April

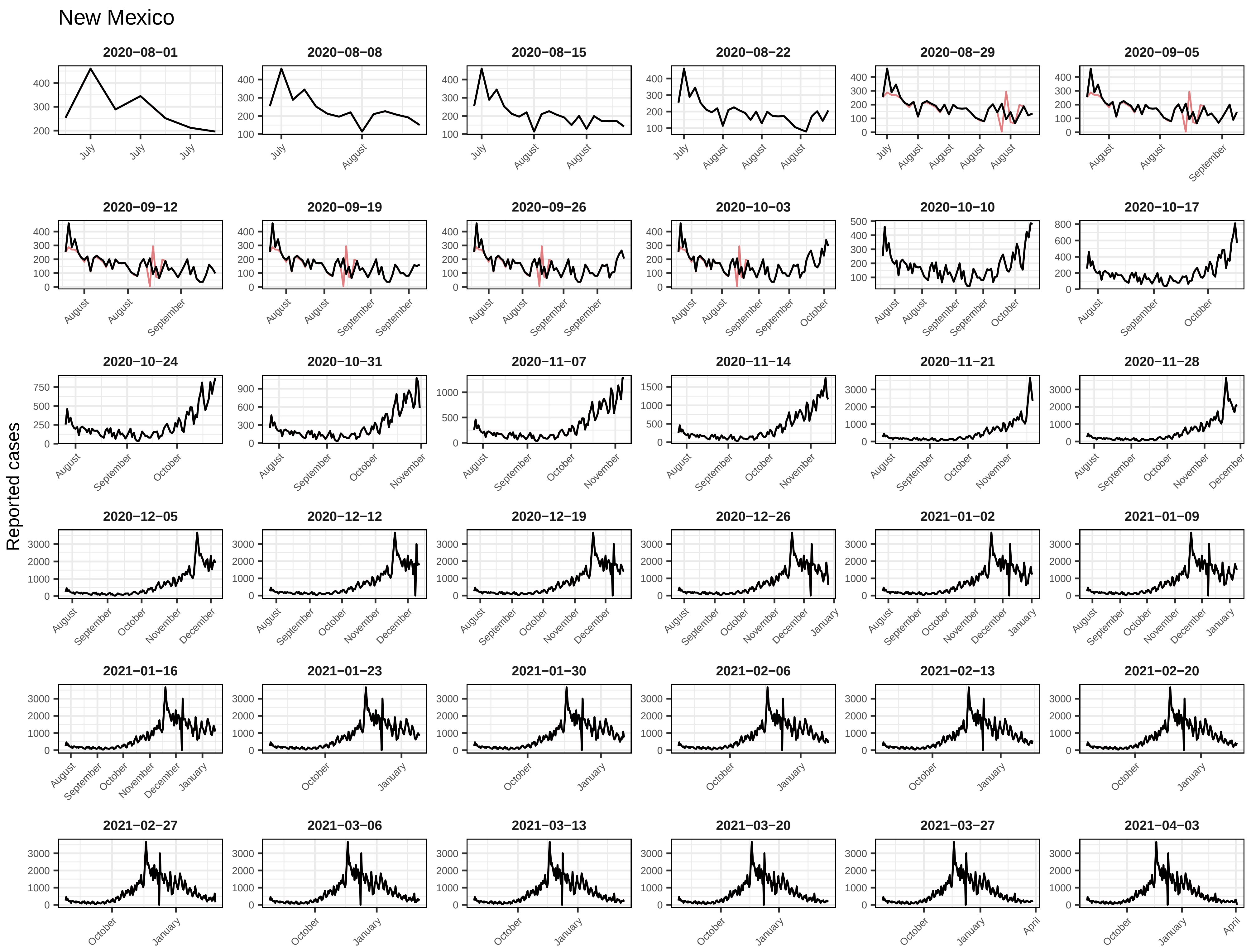
New Mexico
Reported cases
2020−08−01
2020−08−08
2020−08−15
2020−08−22
2020−08−29
2020−09−05
2020−09−12
2020−09−19
2020−09−26
2020−10−03
2020−10−10
2020−10−17
2020−10−24
2020−10−31
2020−11−07
2020−11−14
2020−11−21
2020−11−28
2020−12−05
2020−12−12
2020−12−19
2020−12−26
2021−01−02
2021−01−09
2021−01−16
2021−01−23
2021−01−30
2021−02−06
2021−02−13
2021−02−20
2021−02−27
2021−03−06
2021−03−13
2021−03−20
2021−03−27
2021−04−03

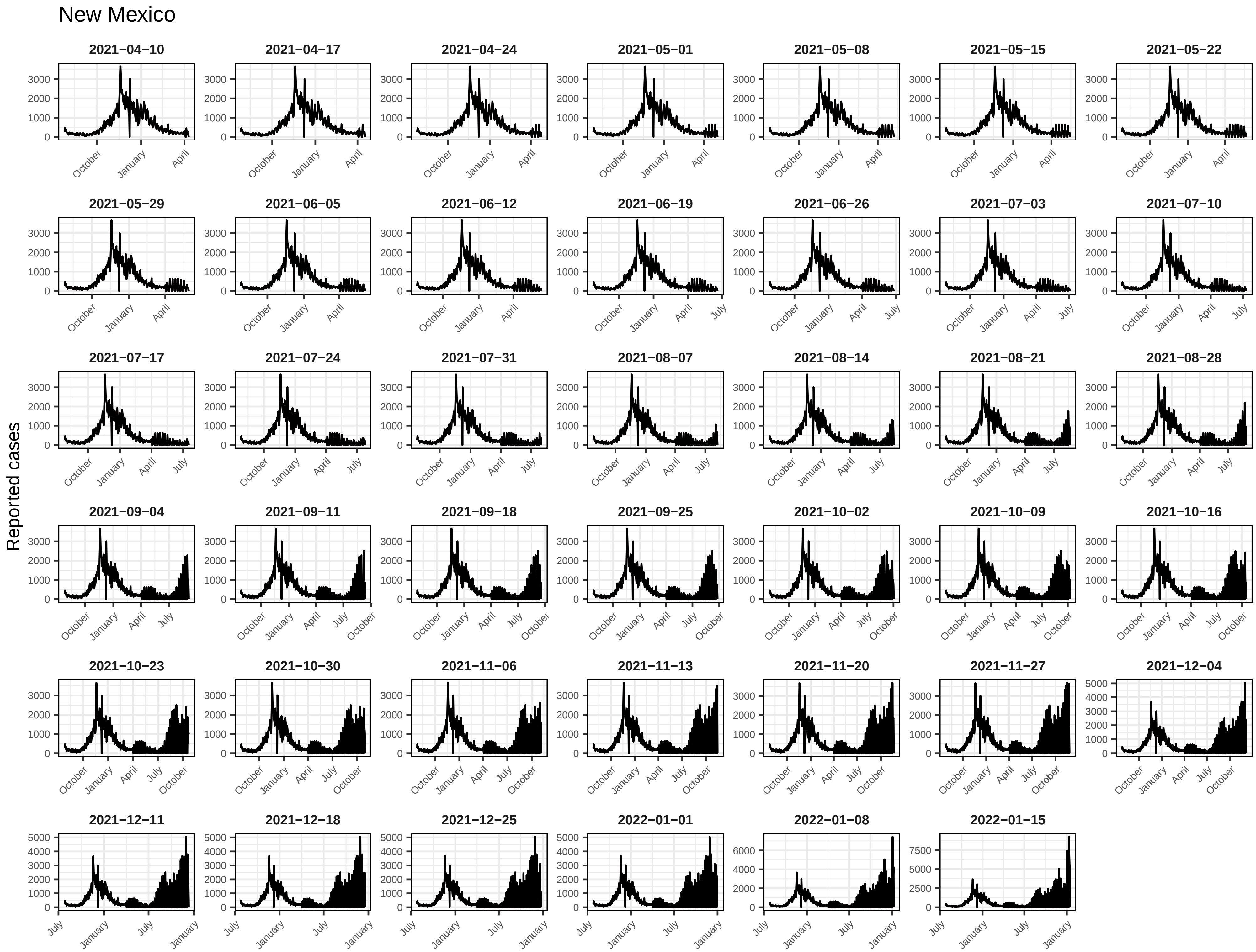
New Mexico
Reported cases
2021−04−10
2021−04−17
2021−04−24
2021−05−01
2021−05−08
2021−05−15
2021−05−22
2021−05−29
2021−06−05
2021−06−12
2021−06−19
2021−06−26
2021−07−03
2021−07−10
2021−07−17
2021−07−24
2021−07−31
2021−08−07
2021−08−14
2021−08−21
2021−08−28
2021−09−04
2021−09−11
2021−09−18
2021−09−25
2021−10−02
2021−10−09
2021−10−16
2021−10−23
2021−10−30
2021−11−06
2021−11−13
2021−11−20
2021−11−27
2021−12−04
2021−12−11
2021−12−18
2021−12−25
2022−01−01
2022−01−08
2022−01−15
0
1000
2000
3000
4000
5000
6000
2500
7500
July
October
January
April

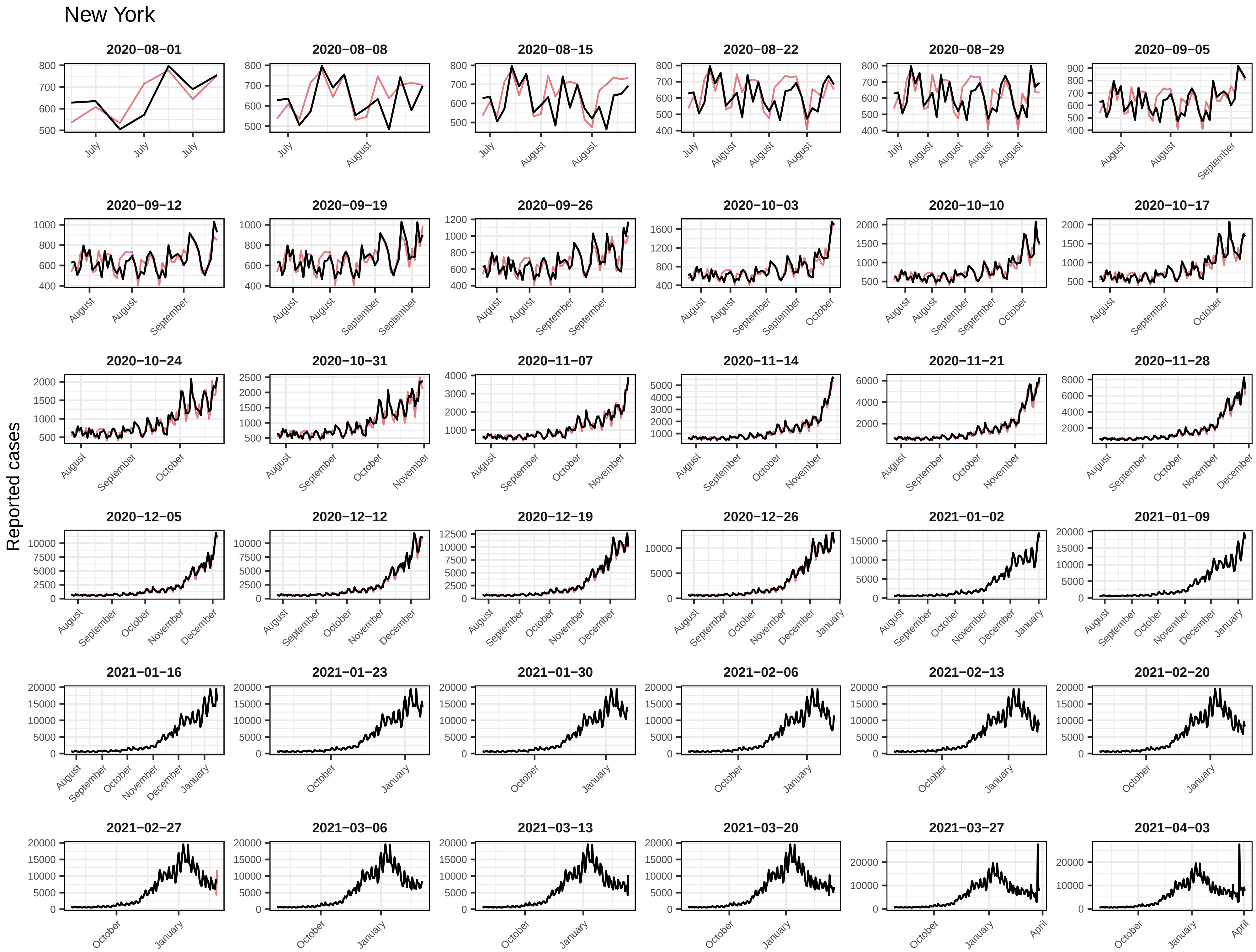
New York
Reported cases
2020−08−01
2020−08−08
2020−08−15
2020−08−22
2020−08−29
2020−09−05
2020−09−12
2020−09−19
2020−09−26
2020−10−03
2020−10−10
2020−10−17
2020−10−24
2020−10−31
2020−11−07
2020−11−14
2020−11−21
2020−11−28
2020−12−05
2020−12−12
2020−12−19
2020−12−26
2021−01−02
2021−01−09
2021−01−16
2021−01−23
2021−01−30
2021−02−06
2021−02−13
2021−02−20
2021−02−27
2021−03−06
2021−03−13
2021−03−20
2021−03−27
2021−04−03

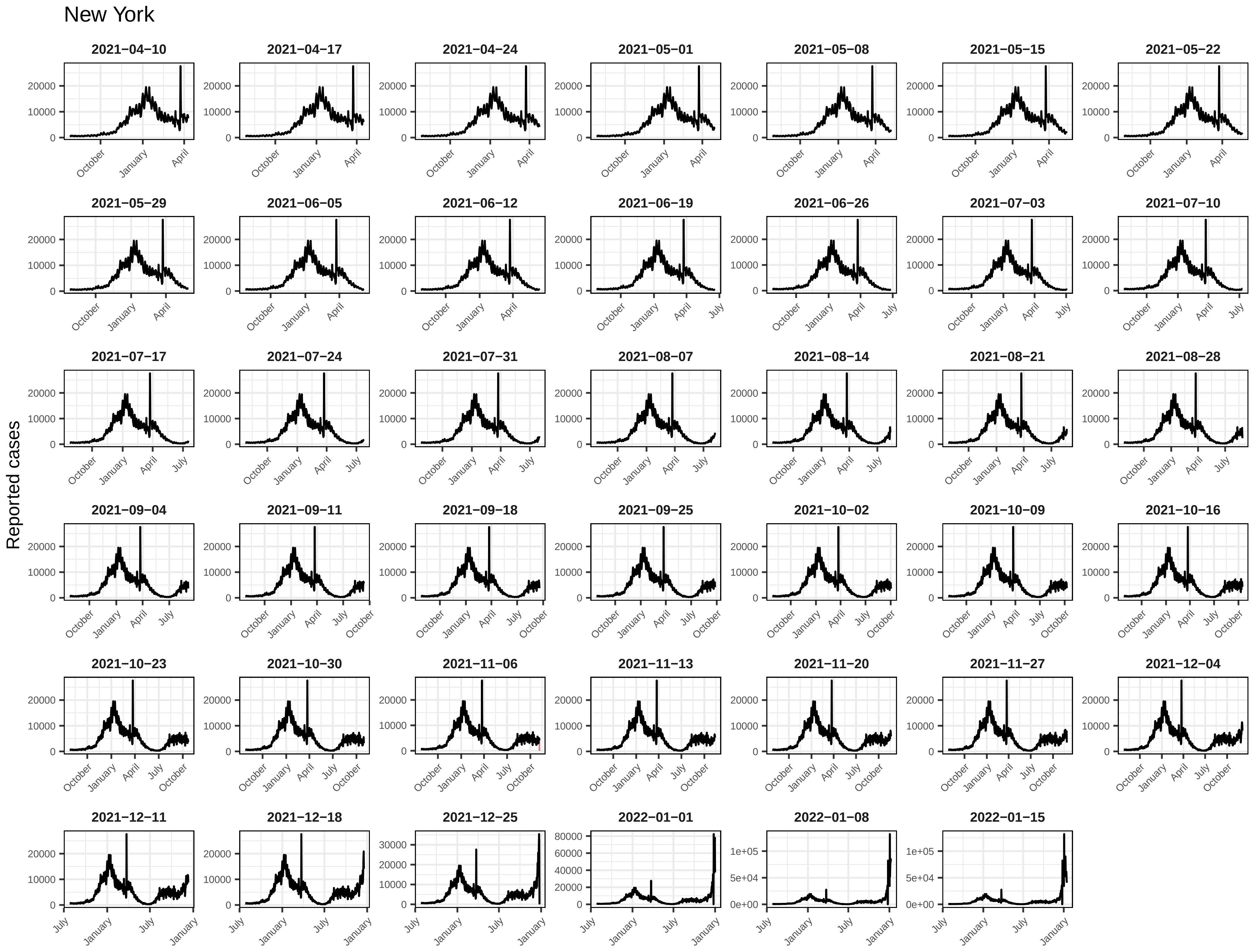
New York
Reported cases
2021−04−10
2021−04−17
2021−04−24
2021−05−01
2021−05−08
2021−05−15
2021−05−22
2021−05−29
2021−06−05
2021−06−12
2021−06−19
2021−06−26
2021−07−03
2021−07−10
2021−07−17
2021−07−24
2021−07−31
2021−08−07
2021−08−14
2021−08−21
2021−08−28
2021−09−04
2021−09−11
2021−09−18
2021−09−25
2021−10−02
2021−10−09
2021−10−16
2021−10−23
2021−10−30
2021−11−06
2021−11−13
2021−11−20
2021−11−27
2021−12−04
2021−12−11
2021−12−18
2021−12−25
2022−01−01
2022−01−08
2022−01−15

North Carolina

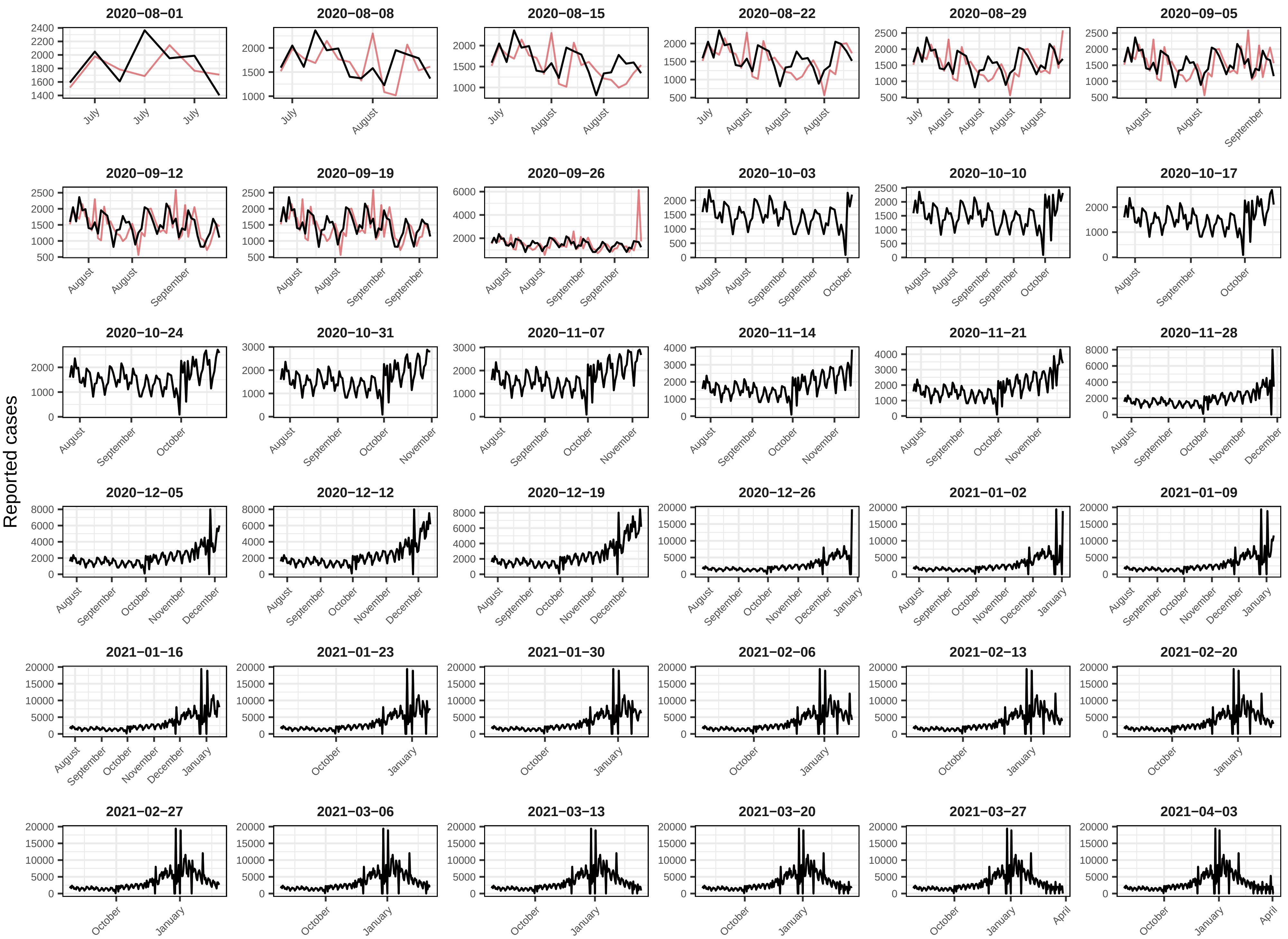

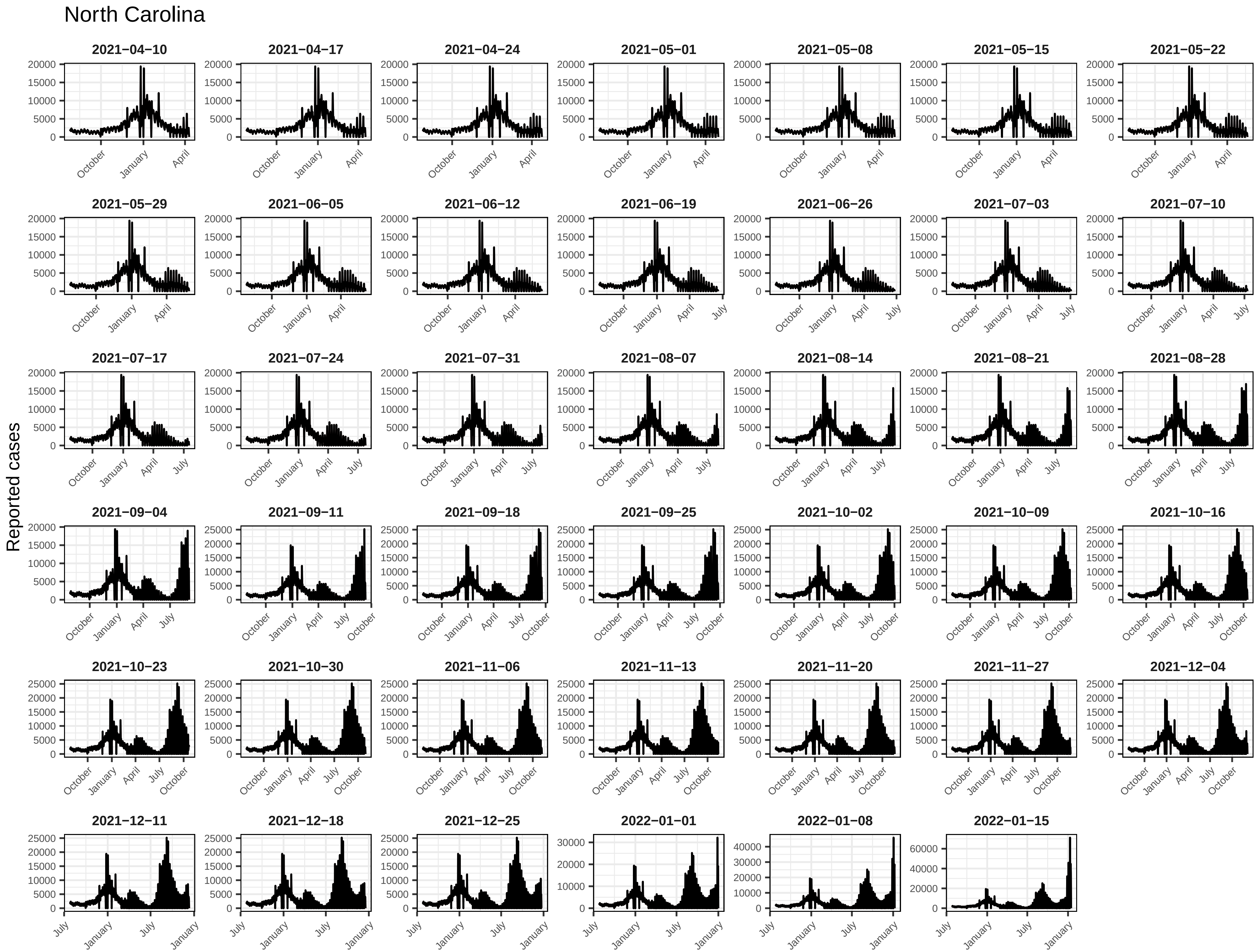
North Carolina
Reported cases
2021−04−10
2021−04−17
2021−04−24
2021−05−01
2021−05−08
2021−05−15
2021−05−22
2021−05−29
2021−06−05
2021−06−12
2021−06−19
2021−06−26
2021−07−03
2021−07−10
2021−07−17
2021−07−24
2021−07−31
2021−08−07
2021−08−14
2021−08−21
2021−08−28
2021−09−04
2021−09−11
2021−09−18
2021−09−25
2021−10−02
2021−10−09
2021−10−16
2021−10−23
2021−10−30
2021−11−06
2021−11−13
2021−11−20
2021−11−27
2021−12−04
2021−12−11
2021−12−18
2021−12−25
2022−01−01
2022−01−08
2022−01−15

North Dakota

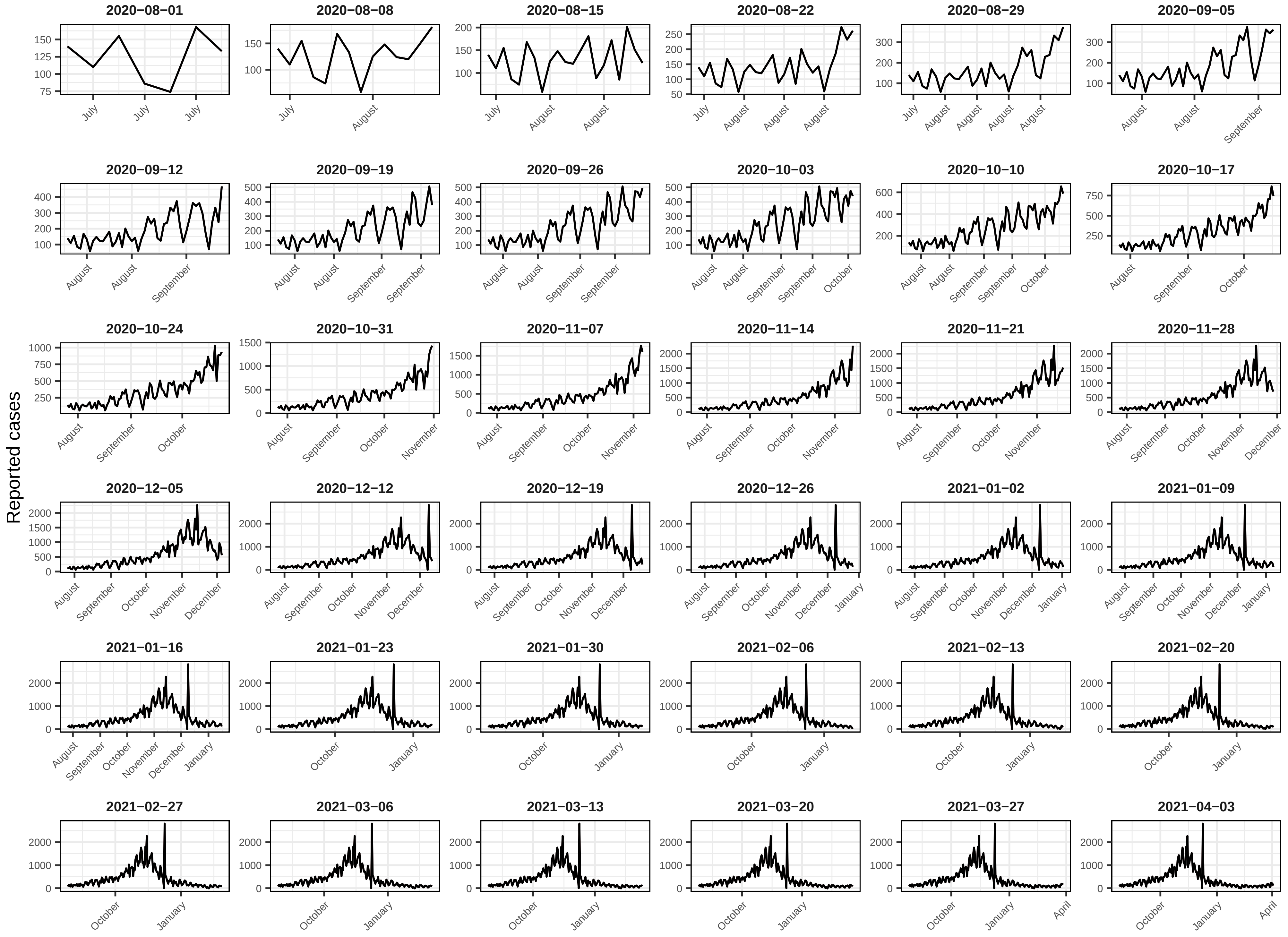

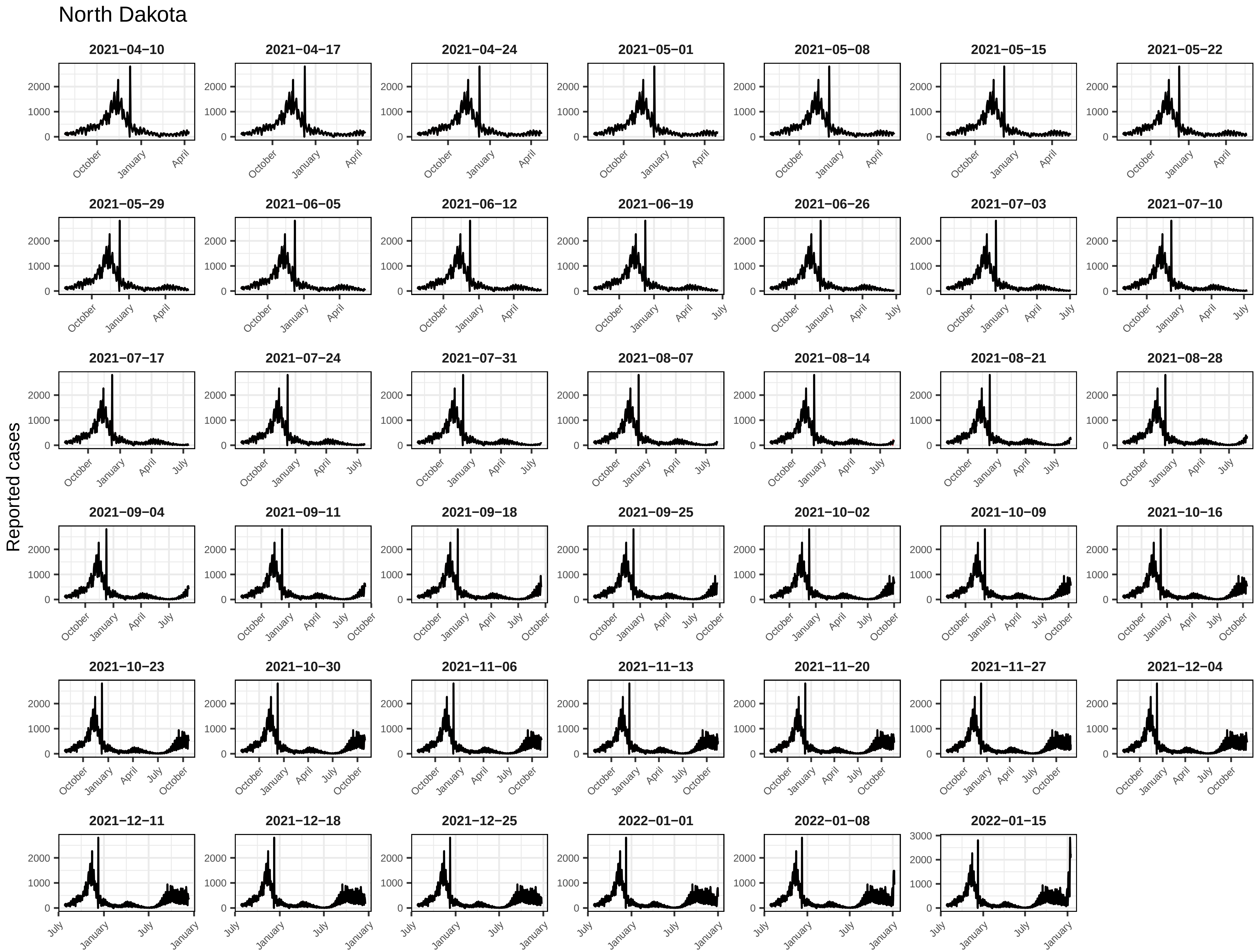
North Dakota
Reported cases
2021−04−10
2021−04−17
2021−04−24
2021−05−01
2021−05−08
2021−05−15
2021−05−22
2021−05−29
2021−06−05
2021−06−12
2021−06−19
2021−06−26
2021−07−03
2021−07−10
2021−07−17
2021−07−24
2021−07−31
2021−08−07
2021−08−14
2021−08−21
2021−08−28
2021−09−04
2021−09−11
2021−09−18
2021−09−25
2021−10−02
2021−10−09
2021−10−16
2021−10−23
2021−10−30
2021−11−06
2021−11−13
2021−11−20
2021−11−27
2021−12−04
2021−12−11
2021−12−18
2021−12−25
2022−01−01
2022−01−08
2022−01−15
0
1000
2000
3000
July
October
January
April

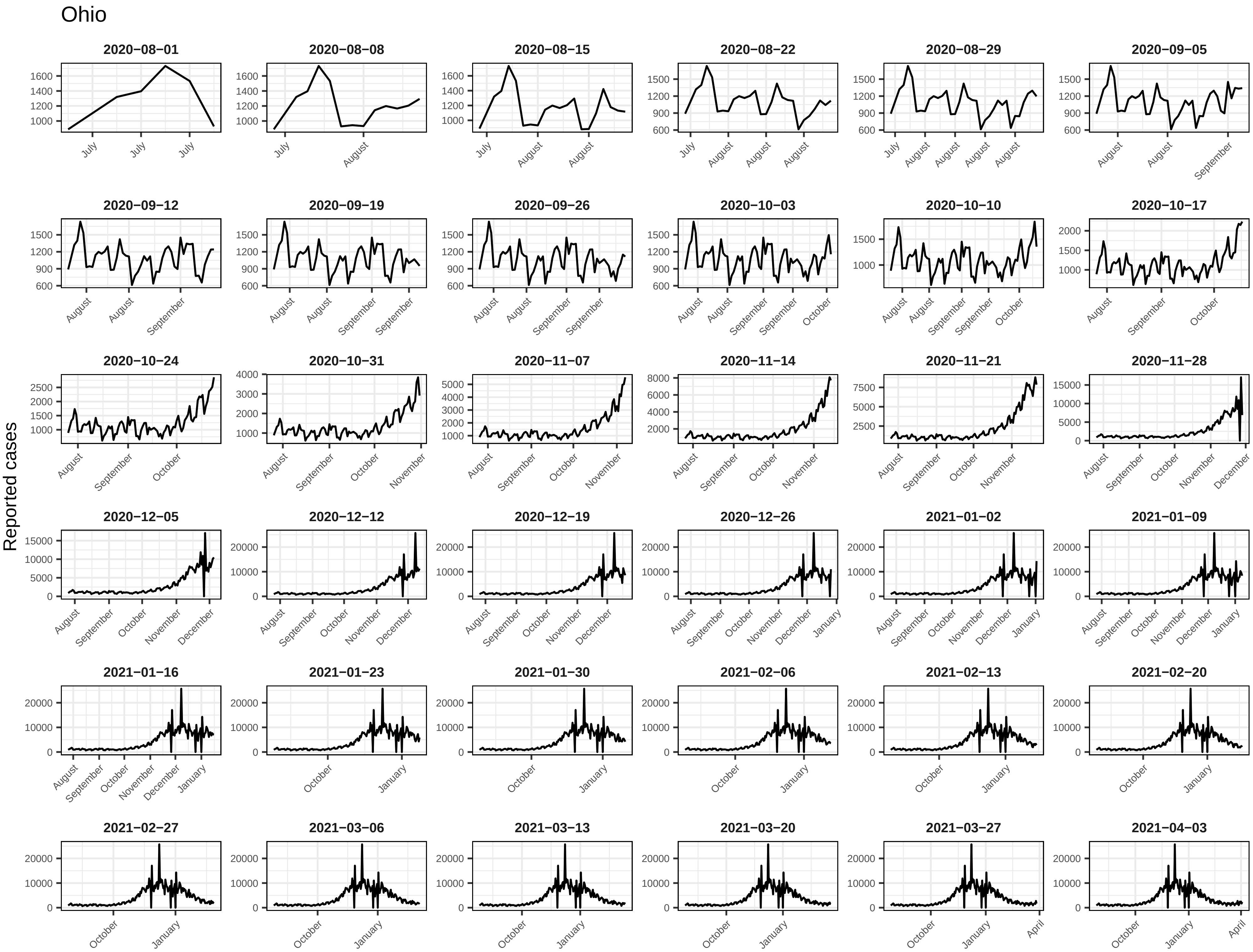
Ohio
Reported cases
2020−08−01
2020−08−08
2020−08−15
2020−08−22
2020−08−29
2020−09−05
2020−09−12
2020−09−19
2020−09−26
2020−10−03
2020−10−10
2020−10−17
2020−10−24
2020−10−31
2020−11−07
2020−11−14
2020−11−21
2020−11−28
2020−12−05
2020−12−12
2020−12−19
2020−12−26
2021−01−02
2021−01−09
2021−01−16
2021−01−23
2021−01−30
2021−02−06
2021−02−13
2021−02−20
2021−02−27
2021−03−06
2021−03−13
2021−03−20
2021−03−27
2021−04−03

Ohio

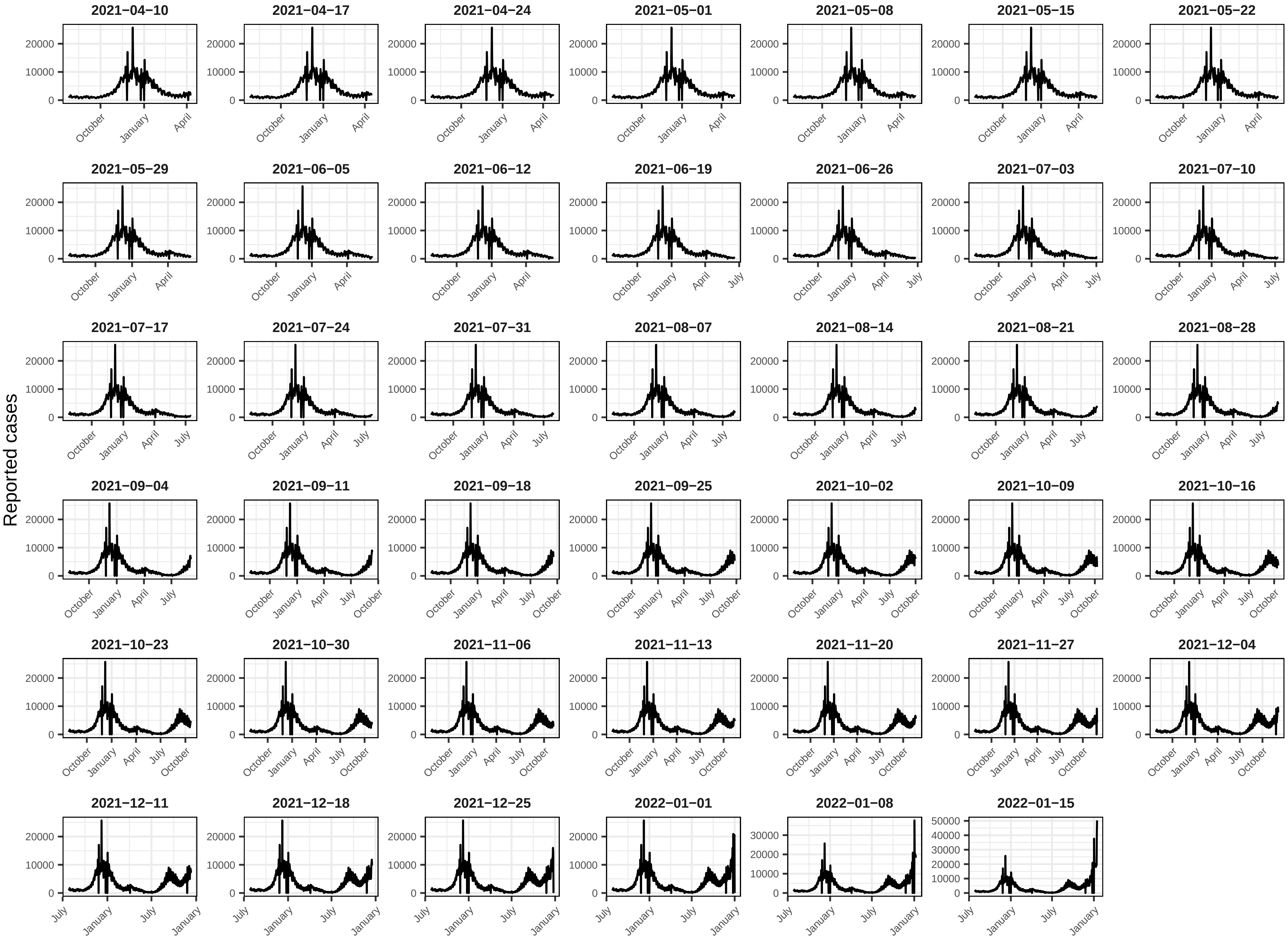

Reported cases
2021−04−10
2021−04−17
2021−04−24
2021−05−01
2021−05−08
2021−05−15
2021−05−22
2021−05−29
2021−06−05
2021−06−12
2021−06−19
2021−06−26
2021−07−03
2021−07−10
2021−07−17
2021−07−24
2021−07−31
2021−08−07
2021−08−14
2021−08−21
2021−08−28
2021−09−04
2021−09−11
2021−09−18
2021−09−25
2021−10−02
2021−10−09
2021−10−16
2021−10−23
2021−10−30
2021−11−06
2021−11−13
2021−11−20
2021−11−27
2021−12−04
2021−12−11
2021−12−18
2021−12−25
2022−01−01
2022−01−08
2022−01−15

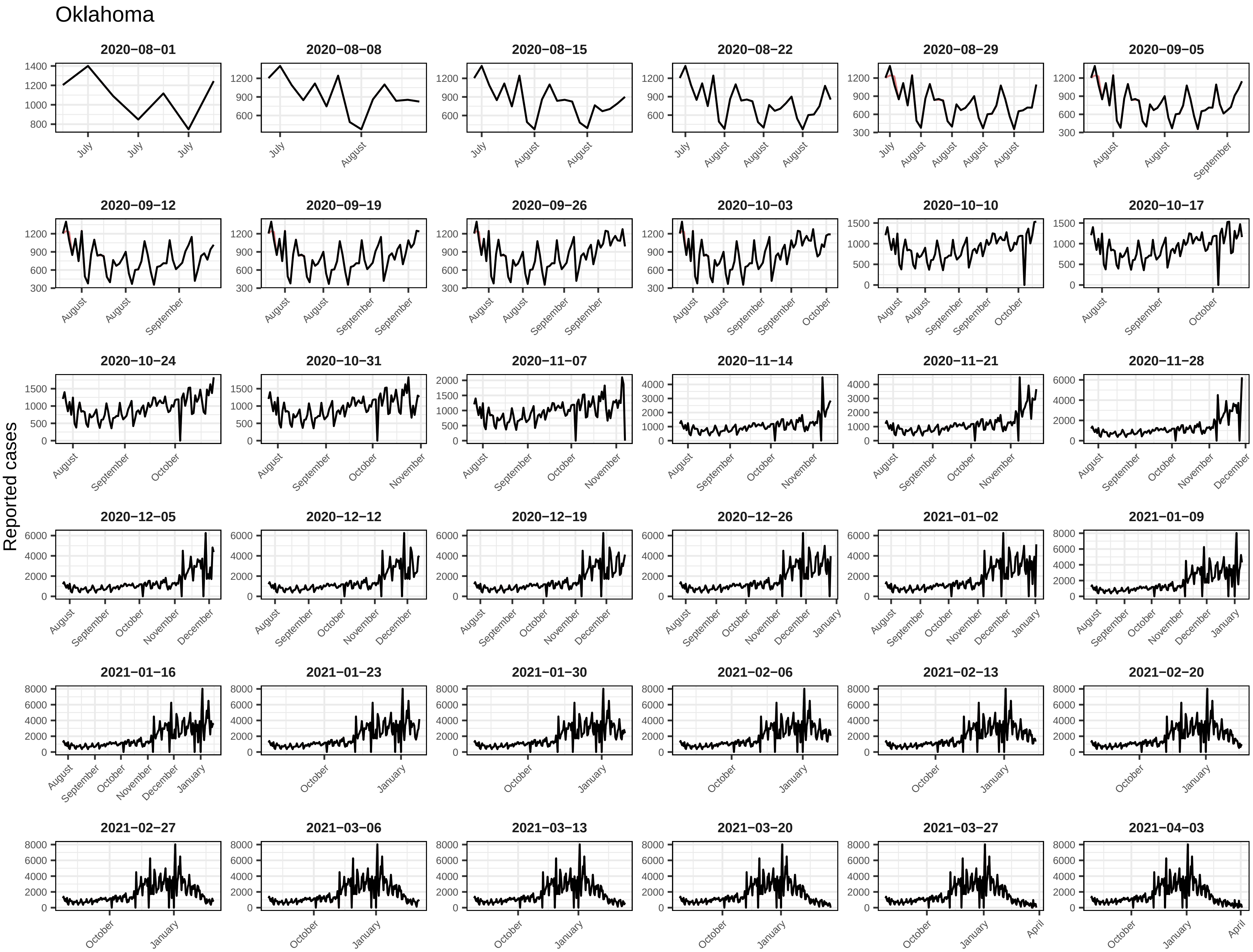

Oklahoma
Reported cases
2020−08−01
2020−08−08
2020−08−15
2020−08−22
2020−08−29
2020−09−05
2020−09−12
2020−09−19
2020−09−26
2020−10−03
2020−10−10
2020−10−17
2020−10−24
2020−10−31
2020−11−07
2020−11−14
2020−11−21
2020−11−28
2020−12−05
2020−12−12
2020−12−19
2020−12−26
2021−01−02
2021−01−09
2021−01−16
2021−01−23
2021−01−30
2021−02−06
2021−02−13
2021−02−20
2021−02−27
2021−03−06
2021−03−13
2021−03−20
2021−03−27
2021−04−03

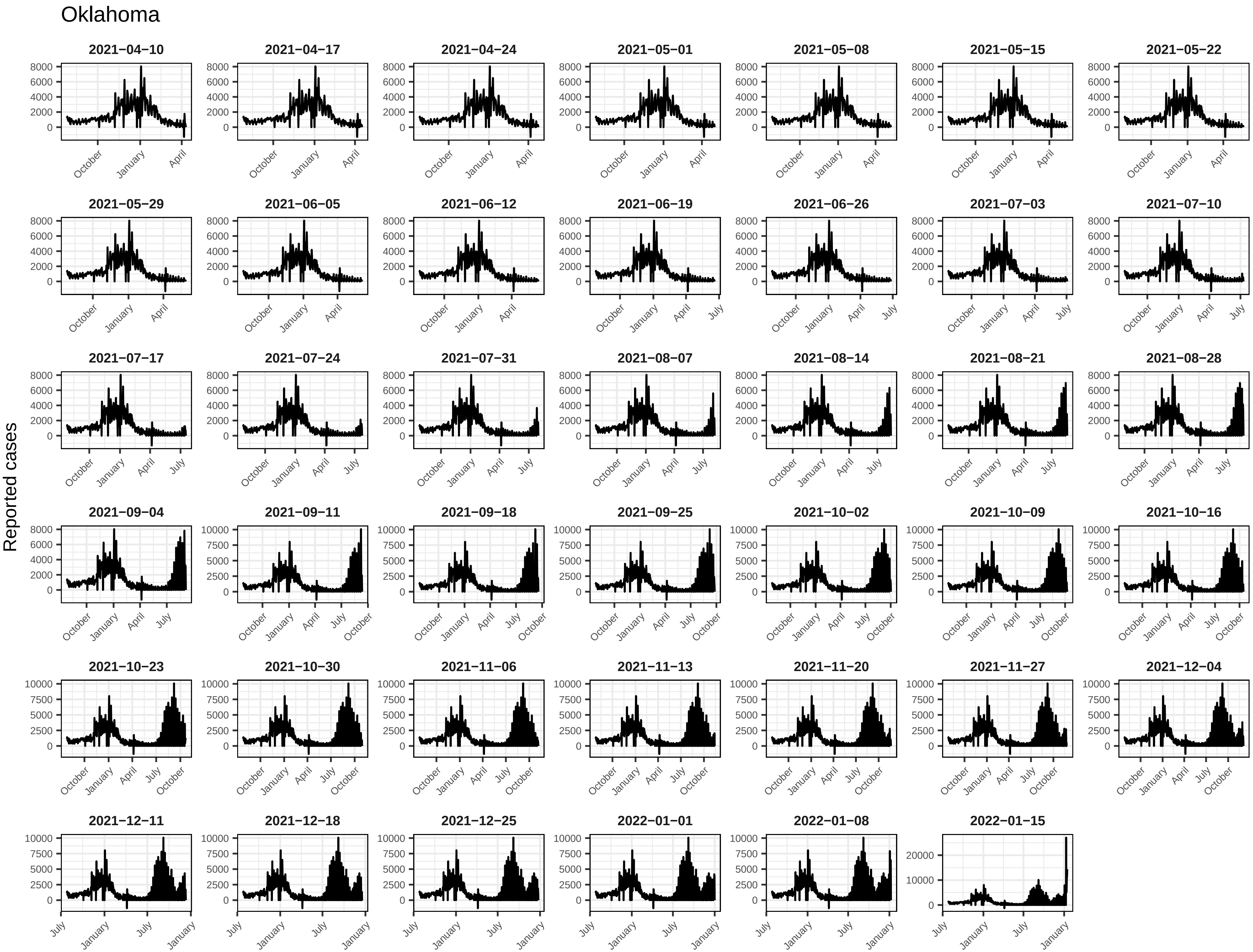
Oklahoma
Reported cases
2021−04−10
2021−04−17
2021−04−24
2021−05−01
2021−05−08
2021−05−15
2021−05−22
2021−05−29
2021−06−05
2021−06−12
2021−06−19
2021−06−26
2021−07−03
2021−07−10
2021−07−17
2021−07−24
2021−07−31
2021−08−07
2021−08−14
2021−08−21
2021−08−28
2021−09−04
2021−09−11
2021−09−18
2021−09−25
2021−10−02
2021−10−09
2021−10−16
2021−10−23
2021−10−30
2021−11−06
2021−11−13
2021−11−20
2021−11−27
2021−12−04
2021−12−11
2021−12−18
2021−12−25
2022−01−01
2022−01−08
2022−01−15

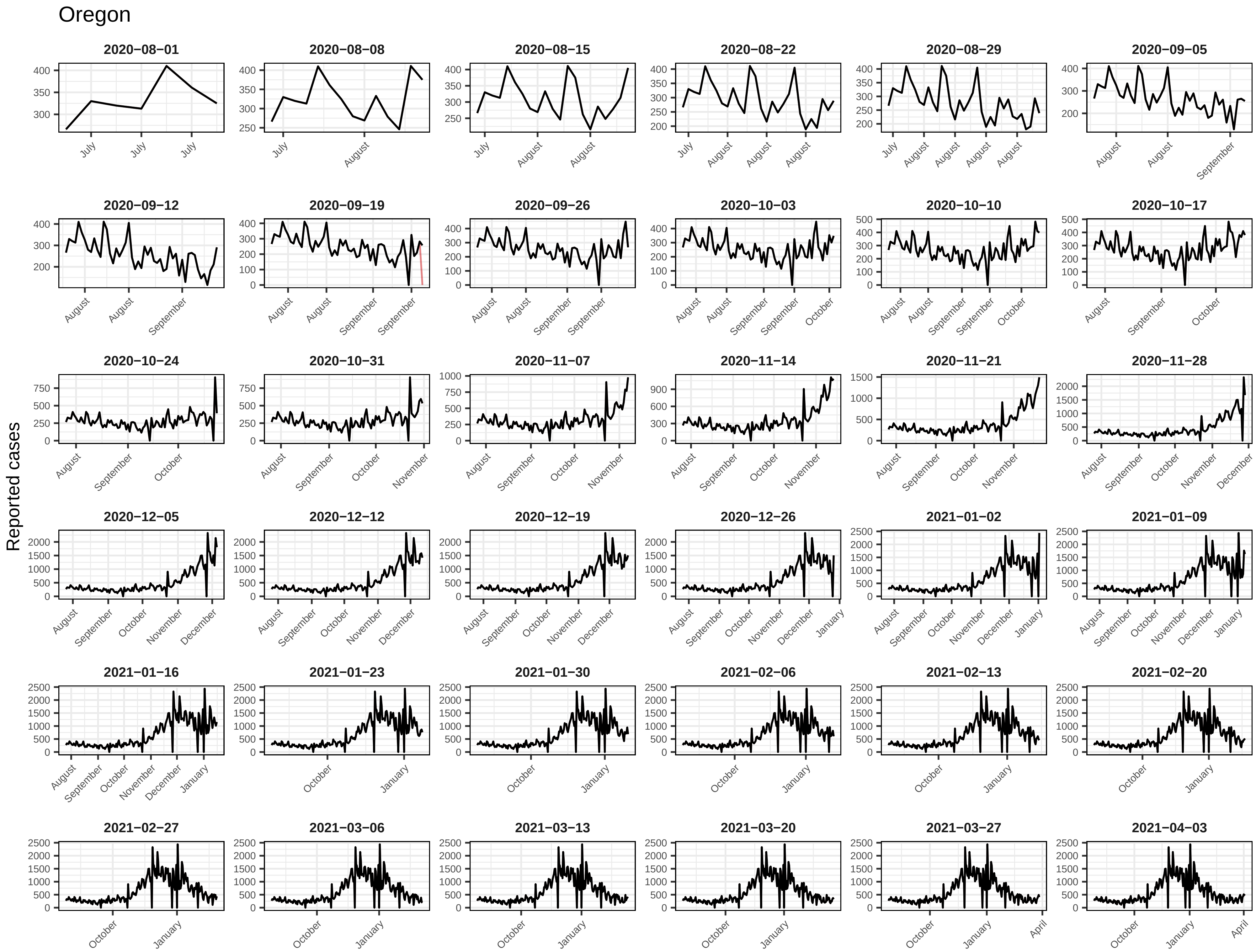
Oregon
2020−08−01
2020−08−08
2020−08−15
2020−08−22
2020−08−29
2020−09−05
2020−09−12
2020−09−19
2020−09−26
2020−10−03
2020−10−10
2020−10−17
2020−10−24
2020−10−31
2020−11−07
2020−11−14
2020−11−21
2020−11−28
2020−12−05
2020−12−12
2020−12−19
2020−12−26
2021−01−02
2021−01−09
2021−01−16
2021−01−23
2021−01−30
2021−02−06
2021−02−13
2021−02−20
2021−02−27
2021−03−06
2021−03−13
2021−03−20
2021−03−27
2021−04−03
Reported cases

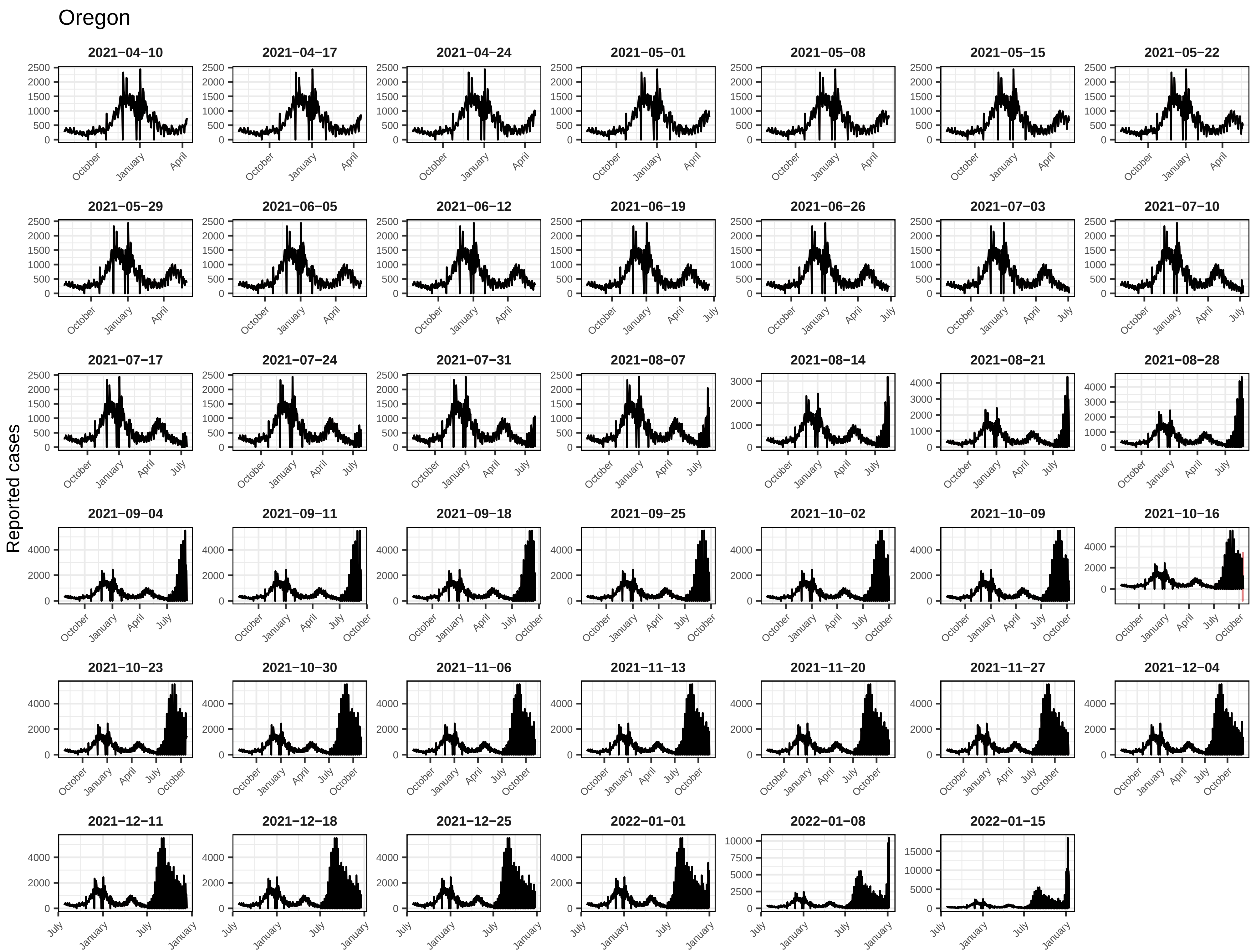
Oregon
Reported cases
2021−04−10
2021−04−17
2021−04−24
2021−05−01
2021−05−08
2021−05−15
2021−05−22
2021−05−29
2021−06−05
2021−06−12
2021−06−19
2021−06−26
2021−07−03
2021−07−10
2021−07−17
2021−07−24
2021−07−31
2021−08−07
2021−08−14
2021−08−21
2021−08−28
2021−09−04
2021−09−11
2021−09−18
2021−09−25
2021−10−02
2021−10−09
2021−10−16
2021−10−23
2021−10−30
2021−11−06
2021−11−13
2021−11−20
2021−11−27
2021−12−04
2021−12−11
2021−12−18
2021−12−25
2022−01−01
2022−01−08
2022−01−15
October
January
April
July

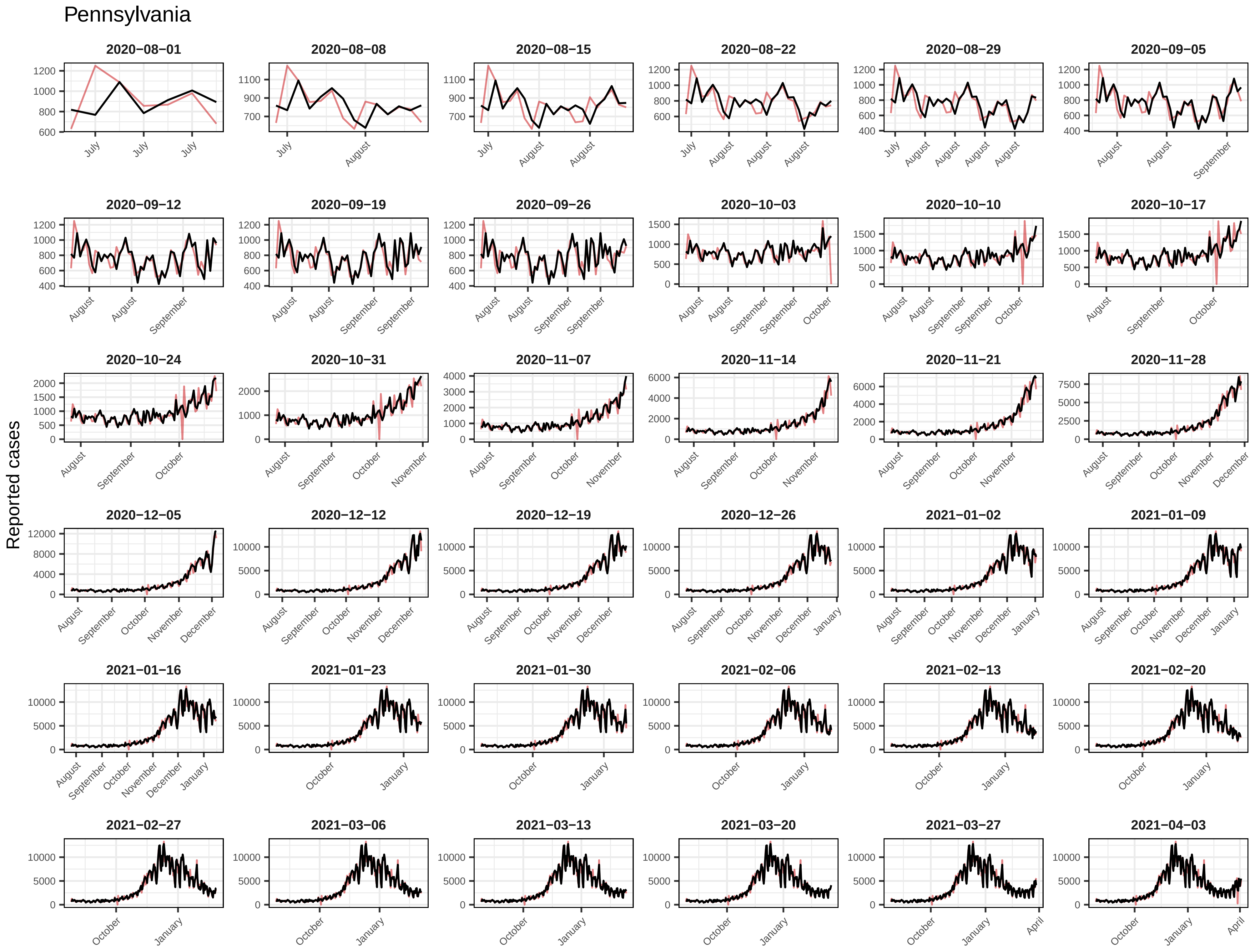

Pennsylvania
Reported cases
2020−08−01
2020−08−08
2020−08−15
2020−08−22
2020−08−29
2020−09−05
2020−09−12
2020−09−19
2020−09−26
2020−10−03
2020−10−10
2020−10−17
2020−10−24
2020−10−31
2020−11−07
2020−11−14
2020−11−21
2020−11−28
2020−12−05
2020−12−12
2020−12−19
2020−12−26
2021−01−02
2021−01−09
2021−01−16
2021−01−23
2021−01−30
2021−02−06
2021−02−13
2021−02−20
2021−02−27
2021−03−06
2021−03−13
2021−03−20
2021−03−27
2021−04−03

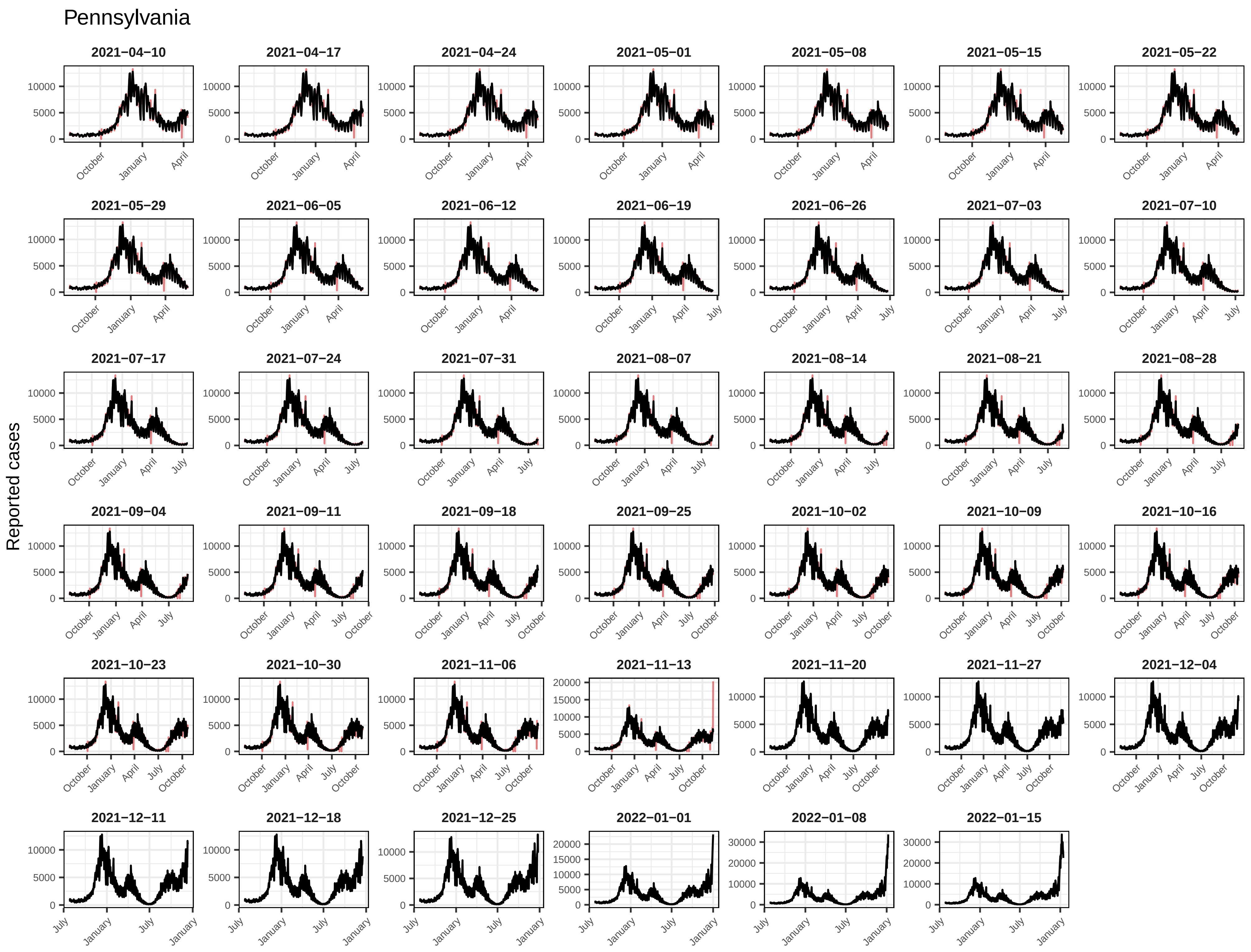

Pennsylvania
Reported cases
2021−04−10
2021−04−17
2021−04−24
2021−05−01
2021−05−08
2021−05−15
2021−05−22
2021−05−29
2021−06−05
2021−06−12
2021−06−19
2021−06−26
2021−07−03
2021−07−10
2021−07−17
2021−07−24
2021−07−31
2021−08−07
2021−08−14
2021−08−21
2021−08−28
2021−09−04
2021−09−11
2021−09−18
2021−09−25
2021−10−02
2021−10−09
2021−10−16
2021−10−23
2021−10−30
2021−11−06
2021−11−13
2021−11−20
2021−11−27
2021−12−04
2021−12−11
2021−12−18
2021−12−25
2022−01−01
2022−01−08
2022−01−15

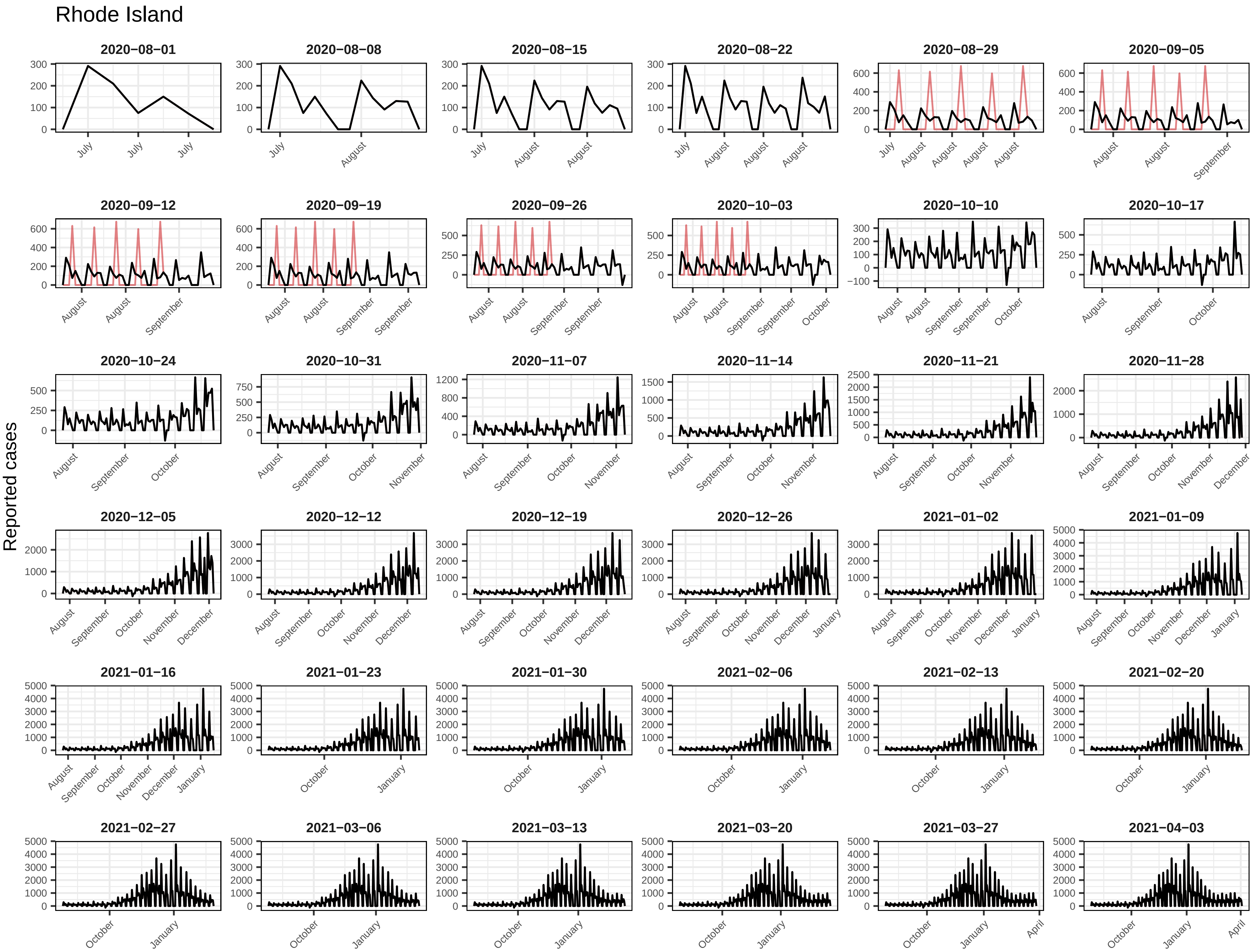

Rhode Island
Reported cases
2020−08−01
2020−08−08
2020−08−15
2020−08−22
2020−08−29
2020−09−05
2020−09−12
2020−09−19
2020−09−26
2020−10−03
2020−10−10
2020−10−17
2020−10−24
2020−10−31
2020−11−07
2020−11−14
2020−11−21
2020−11−28
2020−12−05
2020−12−12
2020−12−19
2020−12−26
2021−01−02
2021−01−09
2021−01−16
2021−01−23
2021−01−30
2021−02−06
2021−02−13
2021−02−20
2021−02−27
2021−03−06
2021−03−13
2021−03−20
2021−03−27
2021−04−03

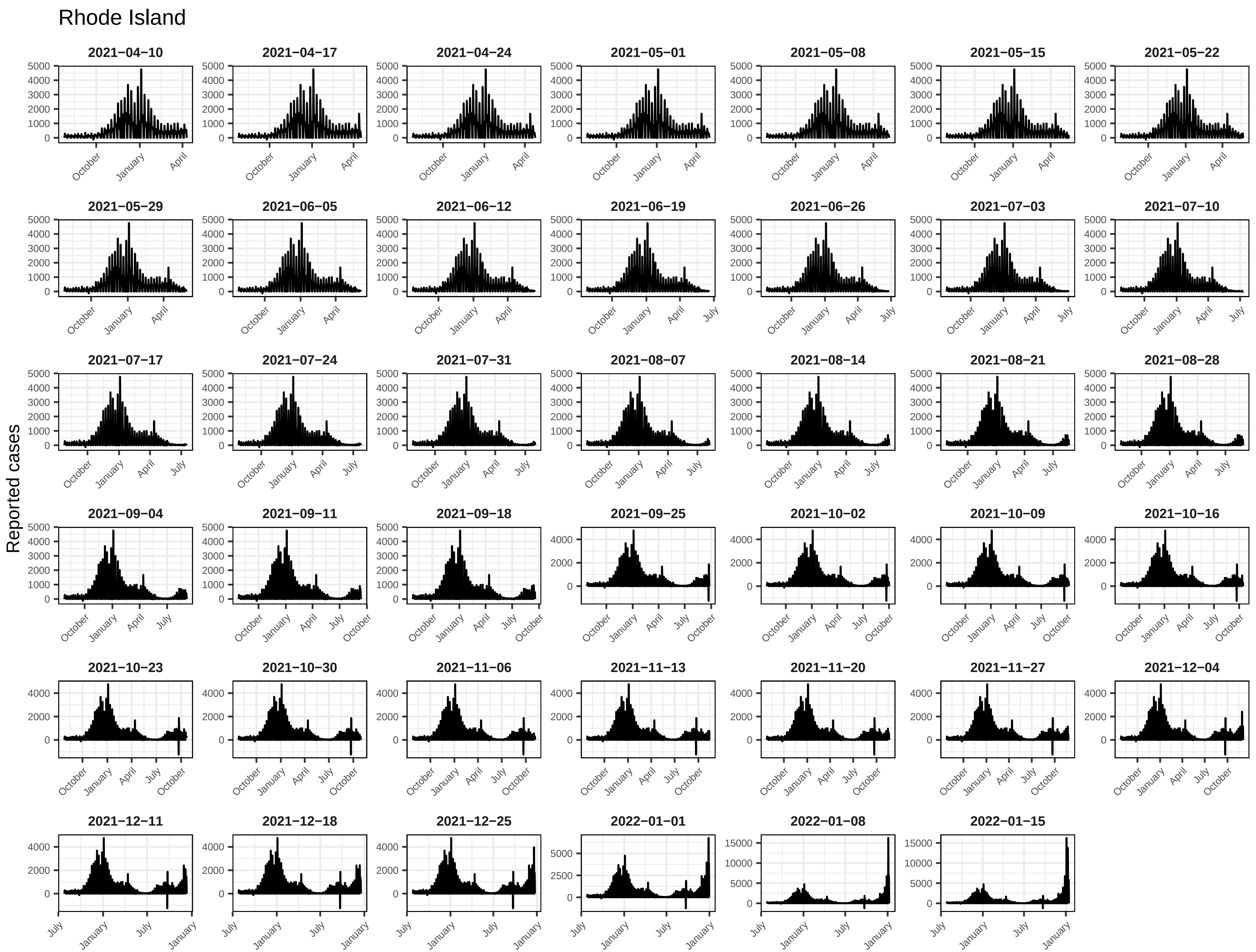

Rhode Island
Reported cases
2021−04−10
2021−04−17
2021−04−24
2021−05−01
2021−05−08
2021−05−15
2021−05−22
2021−05−29
2021−06−05
2021−06−12
2021−06−19
2021−06−26
2021−07−03
2021−07−10
2021−07−17
2021−07−24
2021−07−31
2021−08−07
2021−08−14
2021−08−21
2021−08−28
2021−09−04
2021−09−11
2021−09−18
2021−09−25
2021−10−02
2021−10−09
2021−10−16
2021−10−23
2021−10−30
2021−11−06
2021−11−13
2021−11−20
2021−11−27
2021−12−04
2021−12−11
2021−12−18
2021−12−25
2022−01−01
2022−01−08
2022−01−15
October
January
April
July

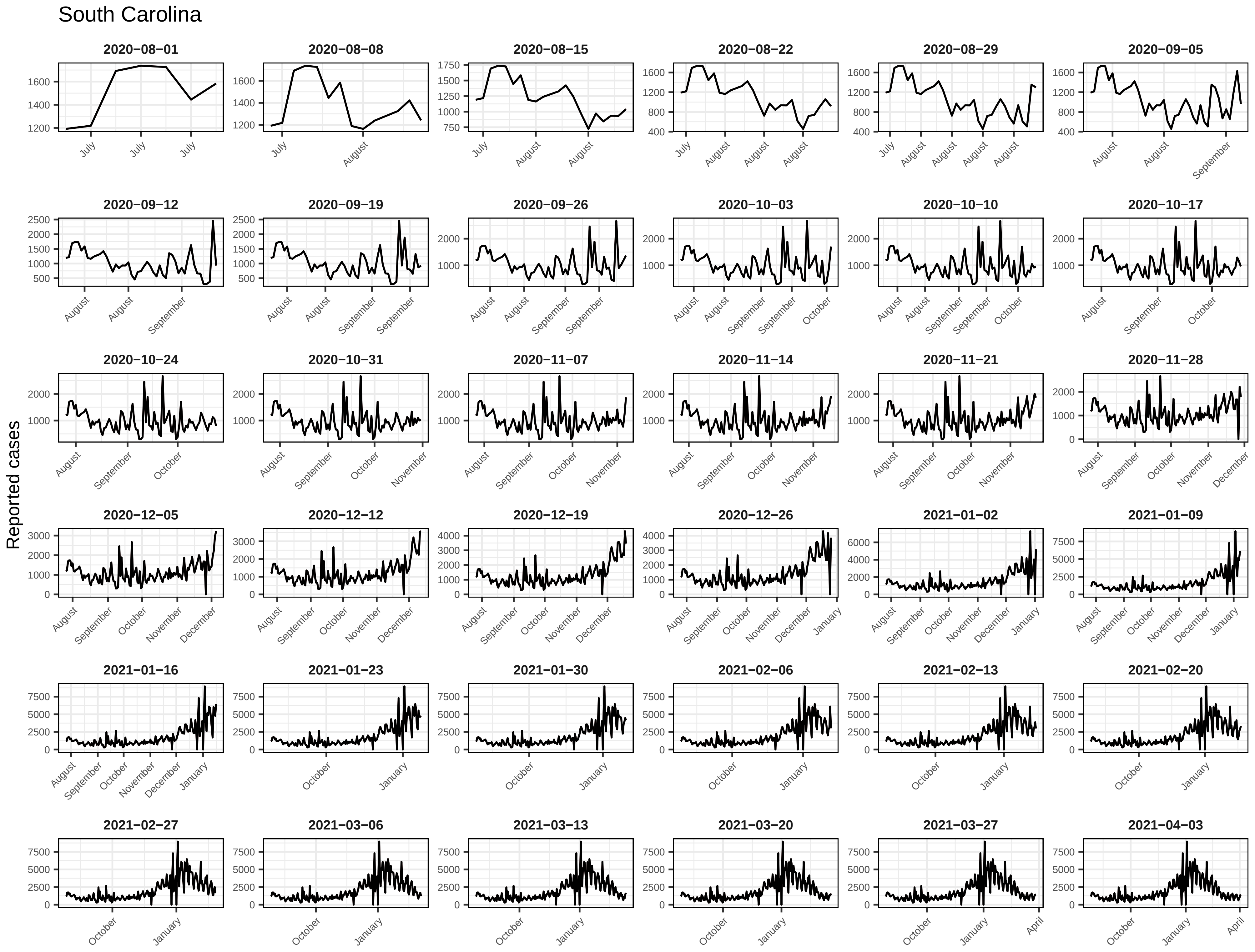
South Carolina
Reported cases
2020−08−01
2020−08−08
2020−08−15
2020−08−22
2020−08−29
2020−09−05
2020−09−12
2020−09−19
2020−09−26
2020−10−03
2020−10−10
2020−10−17
2020−10−24
2020−10−31
2020−11−07
2020−11−14
2020−11−21
2020−11−28
2020−12−05
2020−12−12
2020−12−19
2020−12−26
2021−01−02
2021−01−09
2021−01−16
2021−01−23
2021−01−30
2021−02−06
2021−02−13
2021−02−20
2021−02−27
2021−03−06
2021−03−13
2021−03−20
2021−03−27
2021−04−03

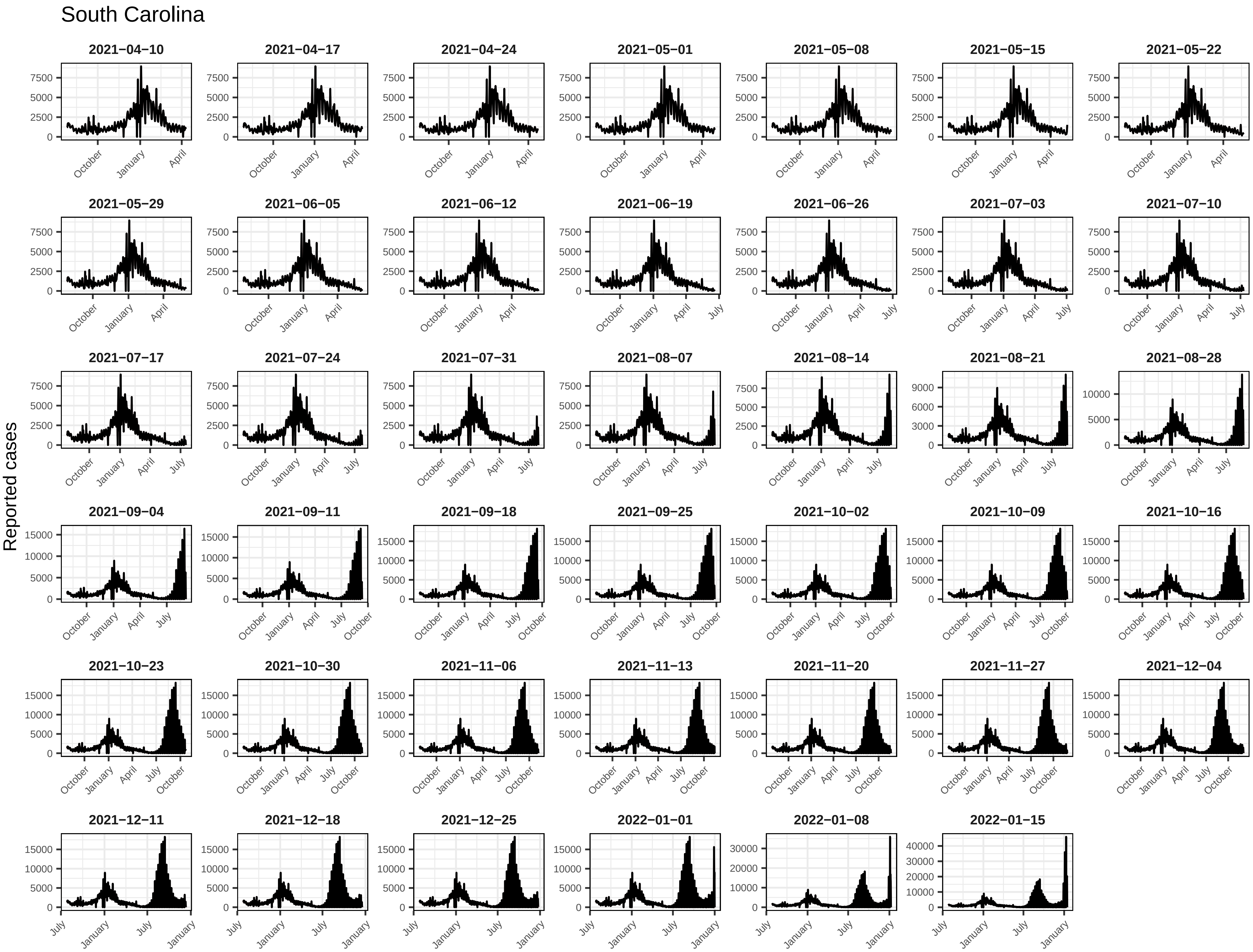
South Carolina
Reported cases
2021−04−10
2021−04−17
2021−04−24
2021−05−01
2021−05−08
2021−05−15
2021−05−22
2021−05−29
2021−06−05
2021−06−12
2021−06−19
2021−06−26
2021−07−03
2021−07−10
2021−07−17
2021−07−24
2021−07−31
2021−08−07
2021−08−14
2021−08−21
2021−08−28
2021−09−04
2021−09−11
2021−09−18
2021−09−25
2021−10−02
2021−10−09
2021−10−16
2021−10−23
2021−10−30
2021−11−06
2021−11−13
2021−11−20
2021−11−27
2021−12−04
2021−12−11
2021−12−18
2021−12−25
2022−01−01
2022−01−08
2022−01−15
October
January
April
July

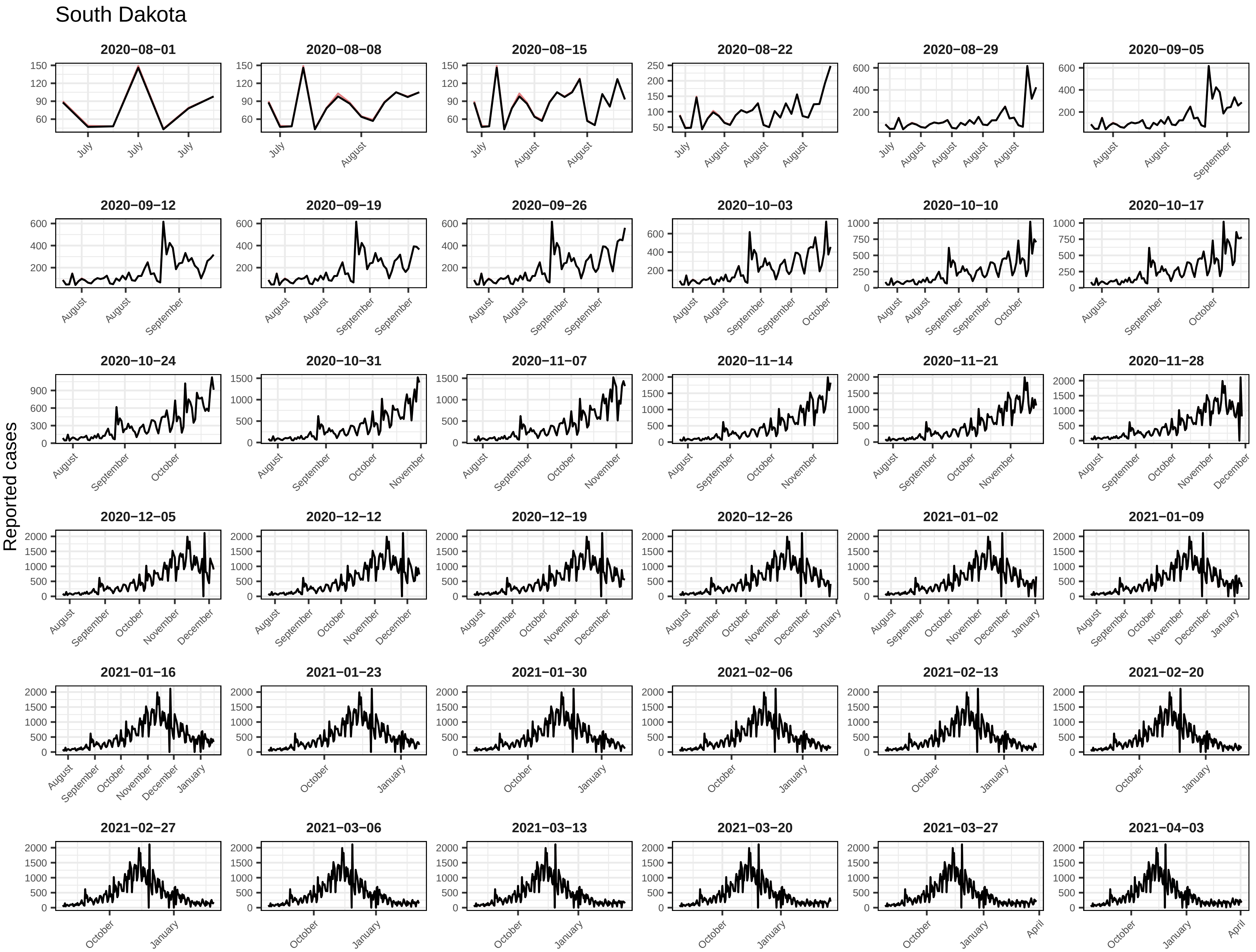
South Dakota
Reported cases
2020−08−01
2020−08−08
2020−08−15
2020−08−22
2020−08−29
2020−09−05
2020−09−12
2020−09−19
2020−09−26
2020−10−03
2020−10−10
2020−10−17
2020−10−24
2020−10−31
2020−11−07
2020−11−14
2020−11−21
2020−11−28
2020−12−05
2020−12−12
2020−12−19
2020−12−26
2021−01−02
2021−01−09
2021−01−16
2021−01−23
2021−01−30
2021−02−06
2021−02−13
2021−02−20
2021−02−27
2021−03−06
2021−03−13
2021−03−20
2021−03−27
2021−04−03

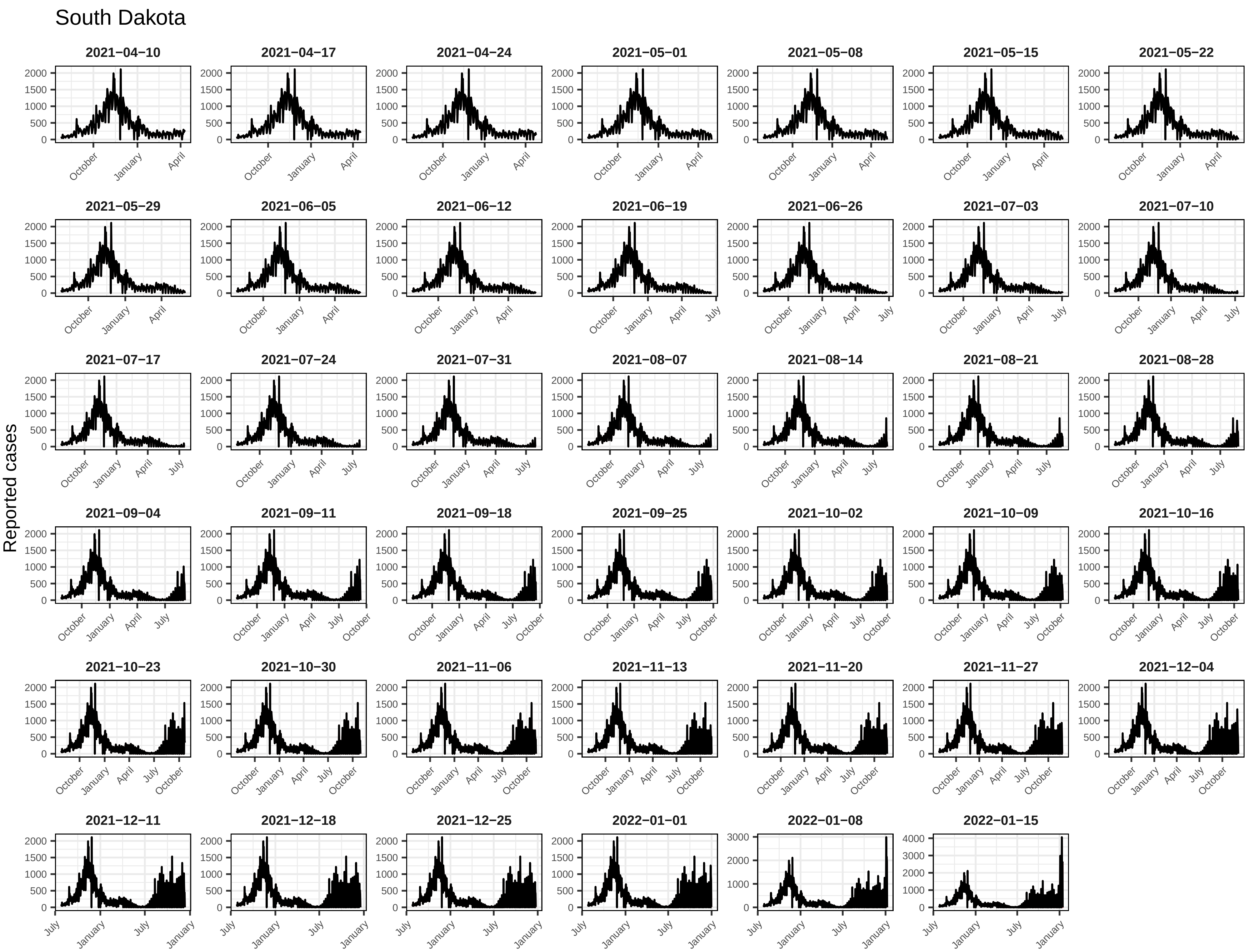
South Dakota
Reported cases
2021−04−10
2021−04−17
2021−04−24
2021−05−01
2021−05−08
2021−05−15
2021−05−22
2021−05−29
2021−06−05
2021−06−12
2021−06−19
2021−06−26
2021−07−03
2021−07−10
2021−07−17
2021−07−24
2021−07−31
2021−08−07
2021−08−14
2021−08−21
2021−08−28
2021−09−04
2021−09−11
2021−09−18
2021−09−25
2021−10−02
2021−10−09
2021−10−16
2021−10−23
2021−10−30
2021−11−06
2021−11−13
2021−11−20
2021−11−27
2021−12−04
2021−12−11
2021−12−18
2021−12−25
2022−01−01
2022−01−08
2022−01−15

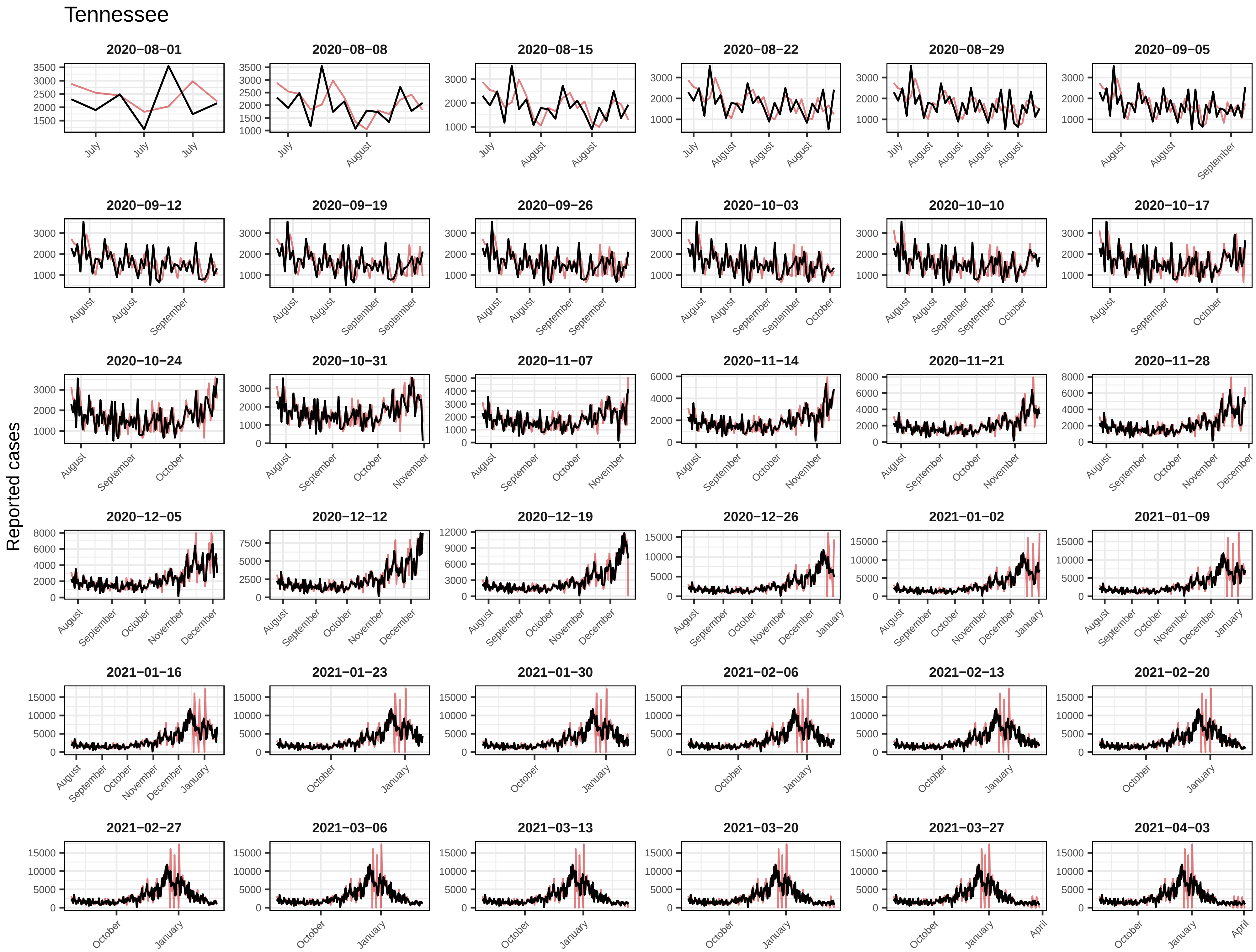

Tennessee
Reported cases
2020−08−01
2020−08−08
2020−08−15
2020−08−22
2020−08−29
2020−09−05
2020−09−12
2020−09−19
2020−09−26
2020−10−03
2020−10−10
2020−10−17
2020−10−24
2020−10−31
2020−11−07
2020−11−14
2020−11−21
2020−11−28
2020−12−05
2020−12−12
2020−12−19
2020−12−26
2021−01−02
2021−01−09
2021−01−16
2021−01−23
2021−01−30
2021−02−06
2021−02−13
2021−02−20
2021−02−27
2021−03−06
2021−03−13
2021−03−20
2021−03−27
2021−04−03

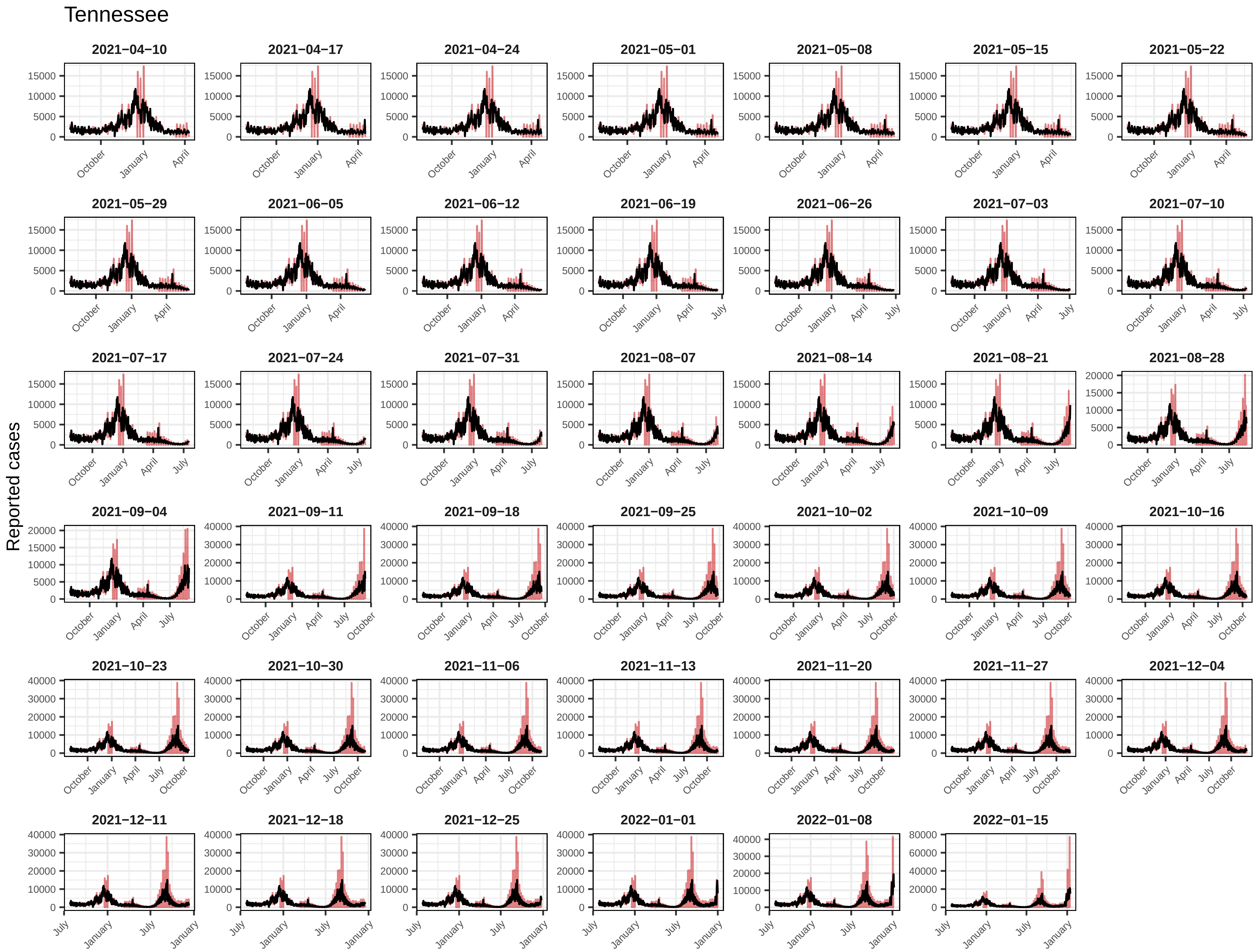

Tennessee
Reported cases
2021−04−10
2021−04−17
2021−04−24
2021−05−01
2021−05−08
2021−05−15
2021−05−22
2021−05−29
2021−06−05
2021−06−12
2021−06−19
2021−06−26
2021−07−03
2021−07−10
2021−07−17
2021−07−24
2021−07−31
2021−08−07
2021−08−14
2021−08−21
2021−08−28
2021−09−04
2021−09−11
2021−09−18
2021−09−25
2021−10−02
2021−10−09
2021−10−16
2021−10−23
2021−10−30
2021−11−06
2021−11−13
2021−11−20
2021−11−27
2021−12−04
2021−12−11
2021−12−18
2021−12−25
2022−01−01
2022−01−08
2022−01−15

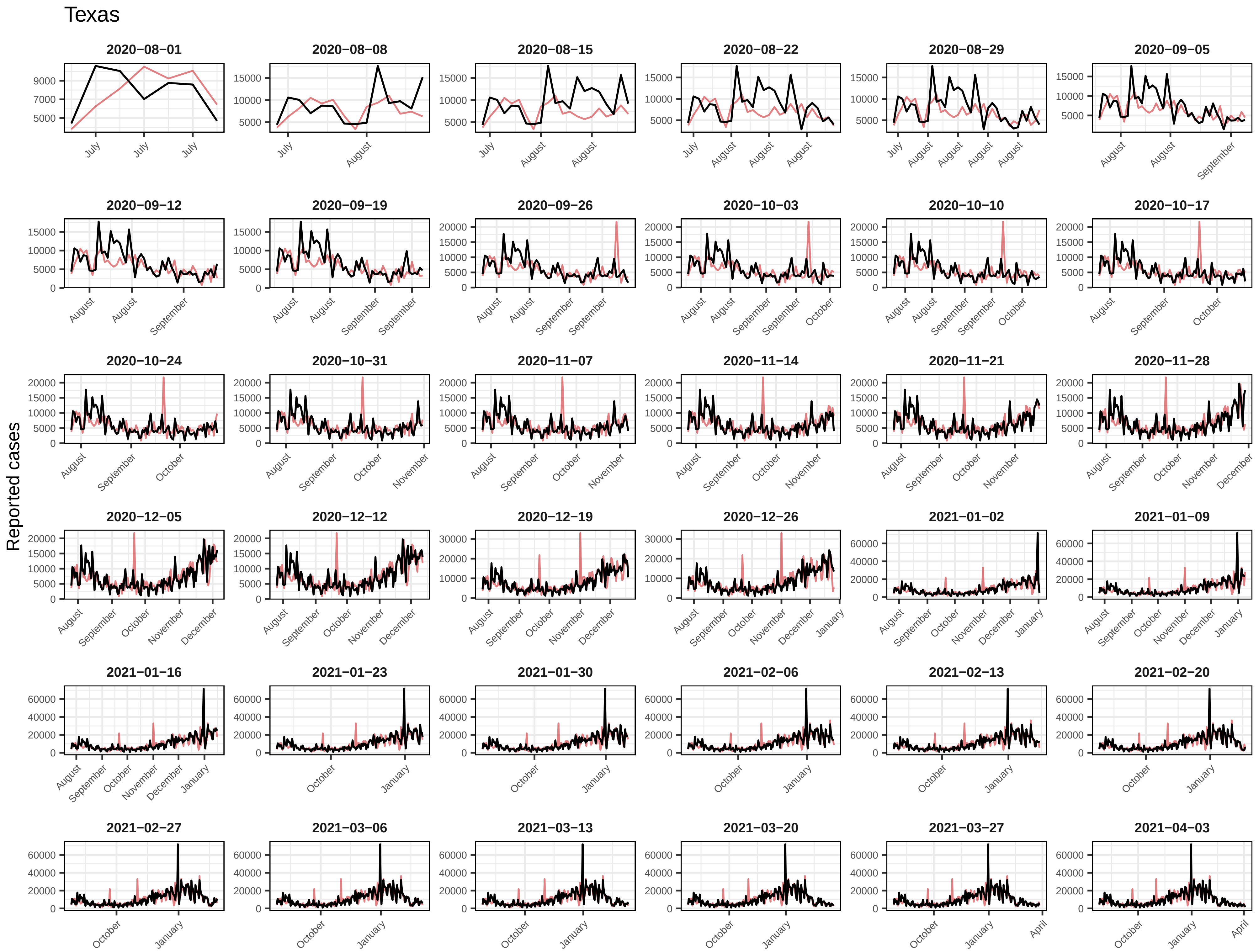

Texas
Reported cases
2020−08−01
2020−08−08
2020−08−15
2020−08−22
2020−08−29
2020−09−05
2020−09−12
2020−09−19
2020−09−26
2020−10−03
2020−10−10
2020−10−17
2020−10−24
2020−10−31
2020−11−07
2020−11−14
2020−11−21
2020−11−28
2020−12−05
2020−12−12
2020−12−19
2020−12−26
2021−01−02
2021−01−09
2021−01−16
2021−01−23
2021−01−30
2021−02−06
2021−02−13
2021−02−20
2021−02−27
2021−03−06
2021−03−13
2021−03−20
2021−03−27
2021−04−03

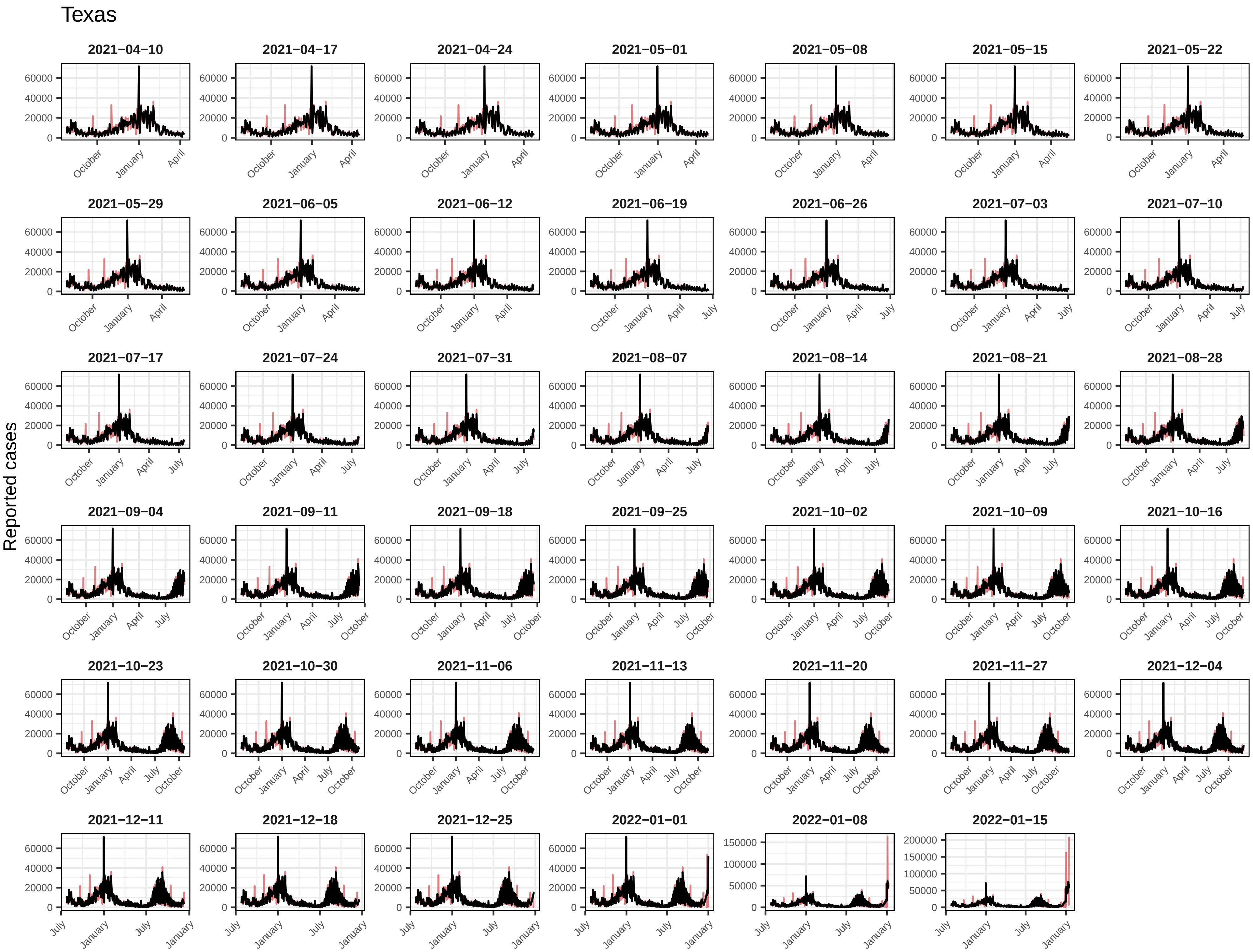
Texas
Reported cases
2021−04−10
2021−04−17
2021−04−24
2021−05−01
2021−05−08
2021−05−15
2021−05−22
2021−05−29
2021−06−05
2021−06−12
2021−06−19
2021−06−26
2021−07−03
2021−07−10
2021−07−17
2021−07−24
2021−07−31
2021−08−07
2021−08−14
2021−08−21
2021−08−28
2021−09−04
2021−09−11
2021−09−18
2021−09−25
2021−10−02
2021−10−09
2021−10−16
2021−10−23
2021−10−30
2021−11−06
2021−11−13
2021−11−20
2021−11−27
2021−12−04
2021−12−11
2021−12−18
2021−12−25
2022−01−01
2022−01−08
2022−01−15

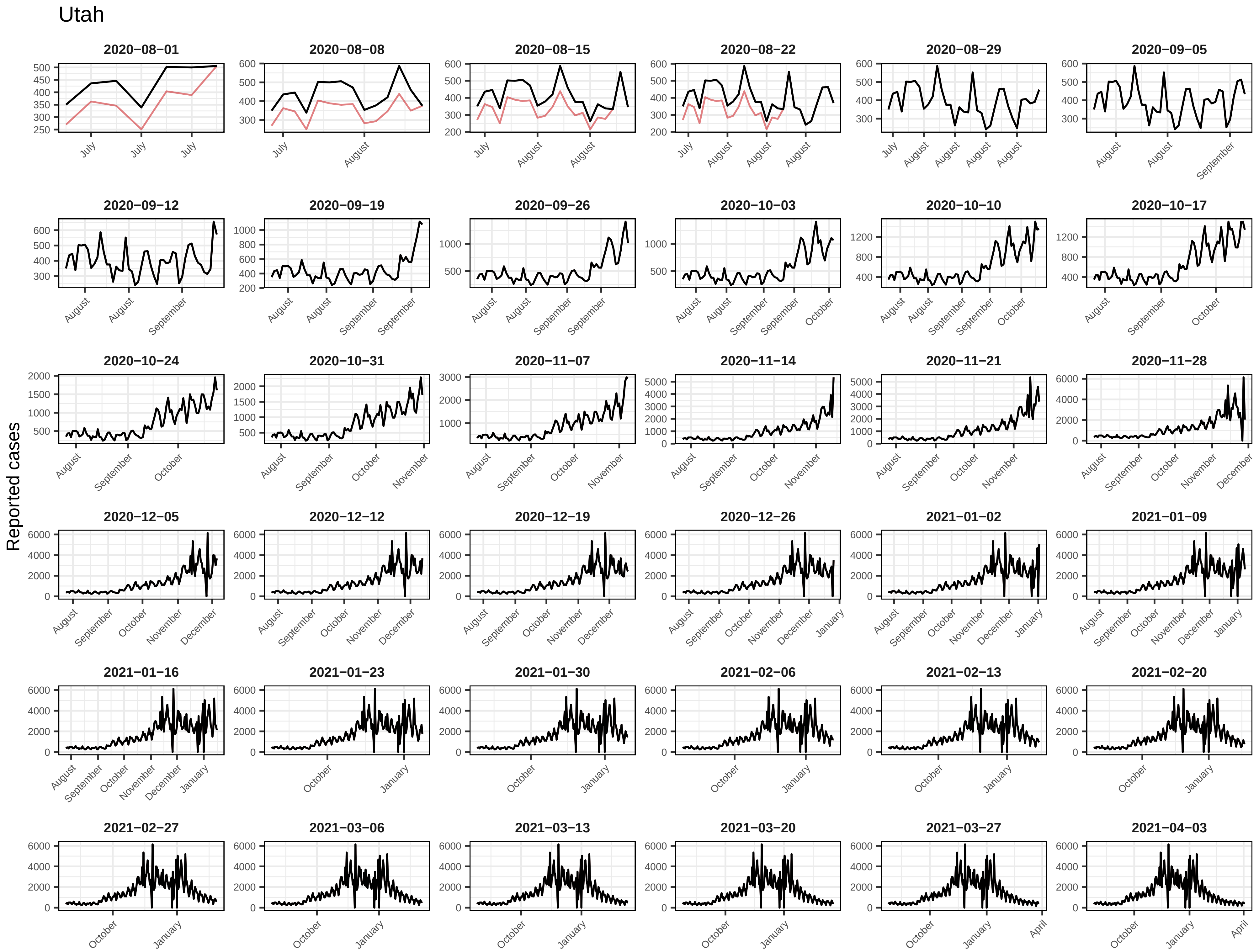
Utah
Reported cases
2020−08−01
2020−08−08
2020−08−15
2020−08−22
2020−08−29
2020−09−05
2020−09−12
2020−09−19
2020−09−26
2020−10−03
2020−10−10
2020−10−17
2020−10−24
2020−10−31
2020−11−07
2020−11−14
2020−11−21
2020−11−28
2020−12−05
2020−12−12
2020−12−19
2020−12−26
2021−01−02
2021−01−09
2021−01−16
2021−01−23
2021−01−30
2021−02−06
2021−02−13
2021−02−20
2021−02−27
2021−03−06
2021−03−13
2021−03−20
2021−03−27
2021−04−03

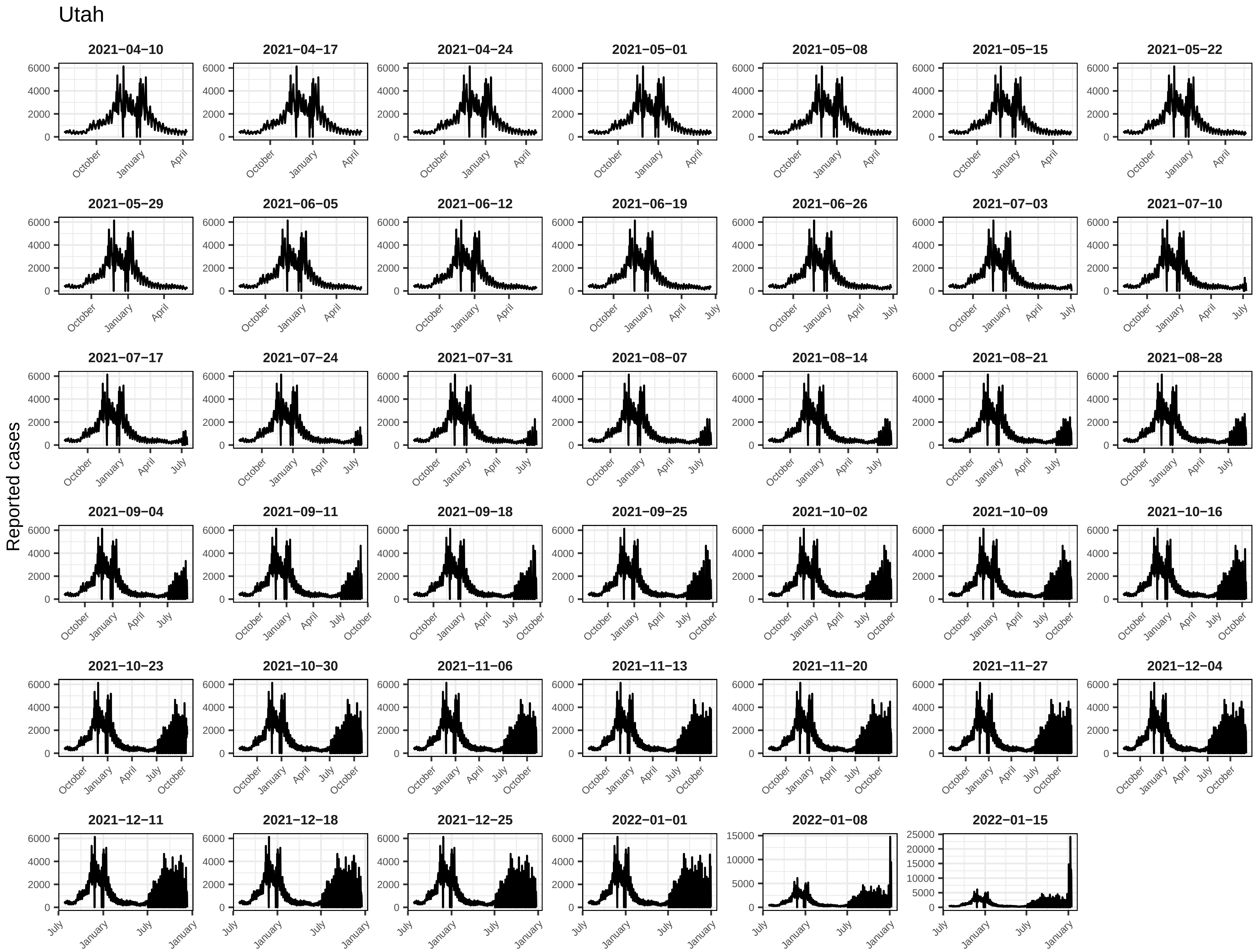
Utah
Reported cases
2021−04−10
2021−04−17
2021−04−24
2021−05−01
2021−05−08
2021−05−15
2021−05−22
2021−05−29
2021−06−05
2021−06−12
2021−06−19
2021−06−26
2021−07−03
2021−07−10
2021−07−17
2021−07−24
2021−07−31
2021−08−07
2021−08−14
2021−08−21
2021−08−28
2021−09−04
2021−09−11
2021−09−18
2021−09−25
2021−10−02
2021−10−09
2021−10−16
2021−10−23
2021−10−30
2021−11−06
2021−11−13
2021−11−20
2021−11−27
2021−12−04
2021−12−11
2021−12−18
2021−12−25
2022−01−01
2022−01−08
2022−01−15

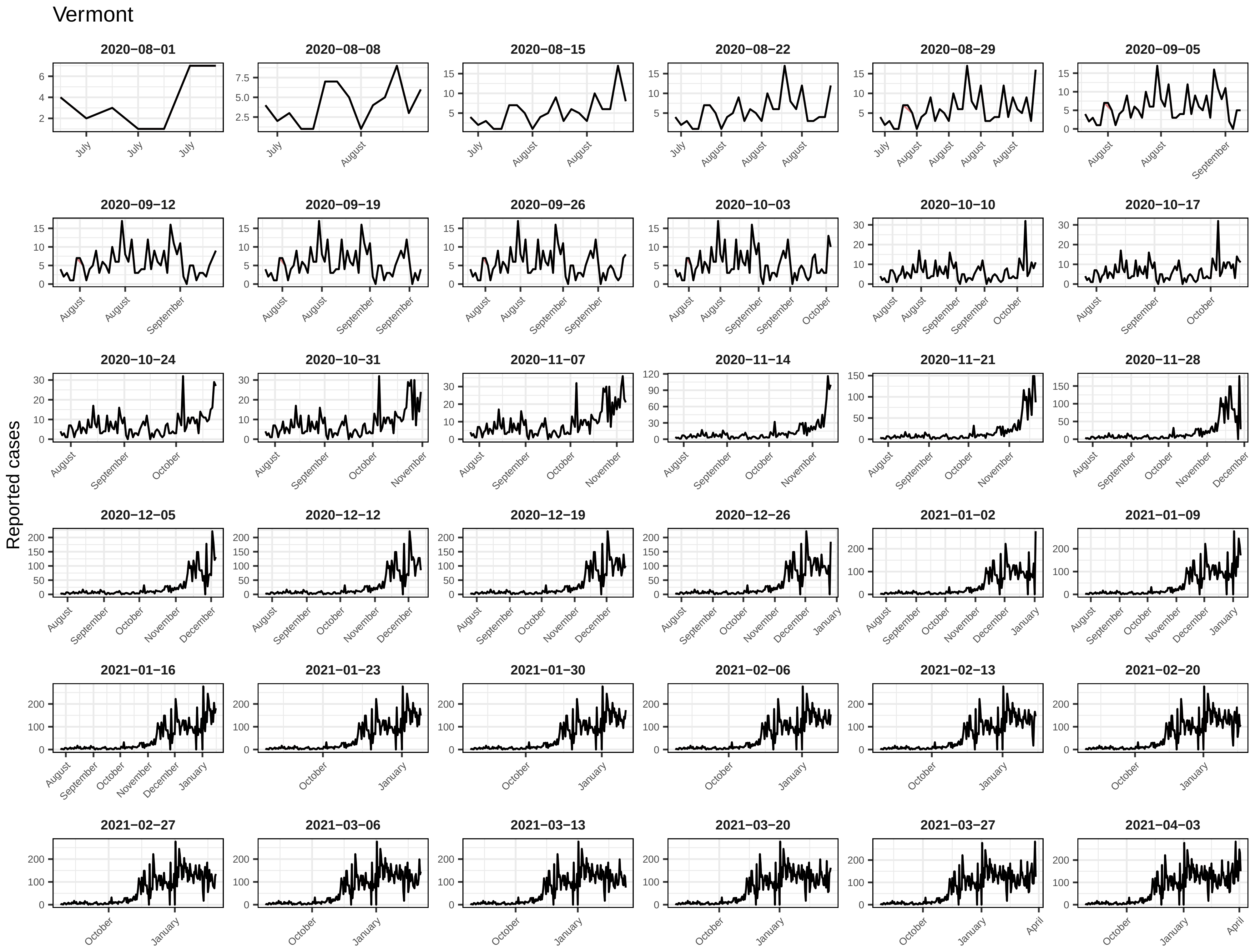

Vermont
Reported cases
2020−08−01
2020−08−08
2020−08−15
2020−08−22
2020−08−29
2020−09−05
2020−09−12
2020−09−19
2020−09−26
2020−10−03
2020−10−10
2020−10−17
2020−10−24
2020−10−31
2020−11−07
2020−11−14
2020−11−21
2020−11−28
2020−12−05
2020−12−12
2020−12−19
2020−12−26
2021−01−02
2021−01−09
2021−01−16
2021−01−23
2021−01−30
2021−02−06
2021−02−13
2021−02−20
2021−02−27
2021−03−06
2021−03−13
2021−03−20
2021−03−27
2021−04−03

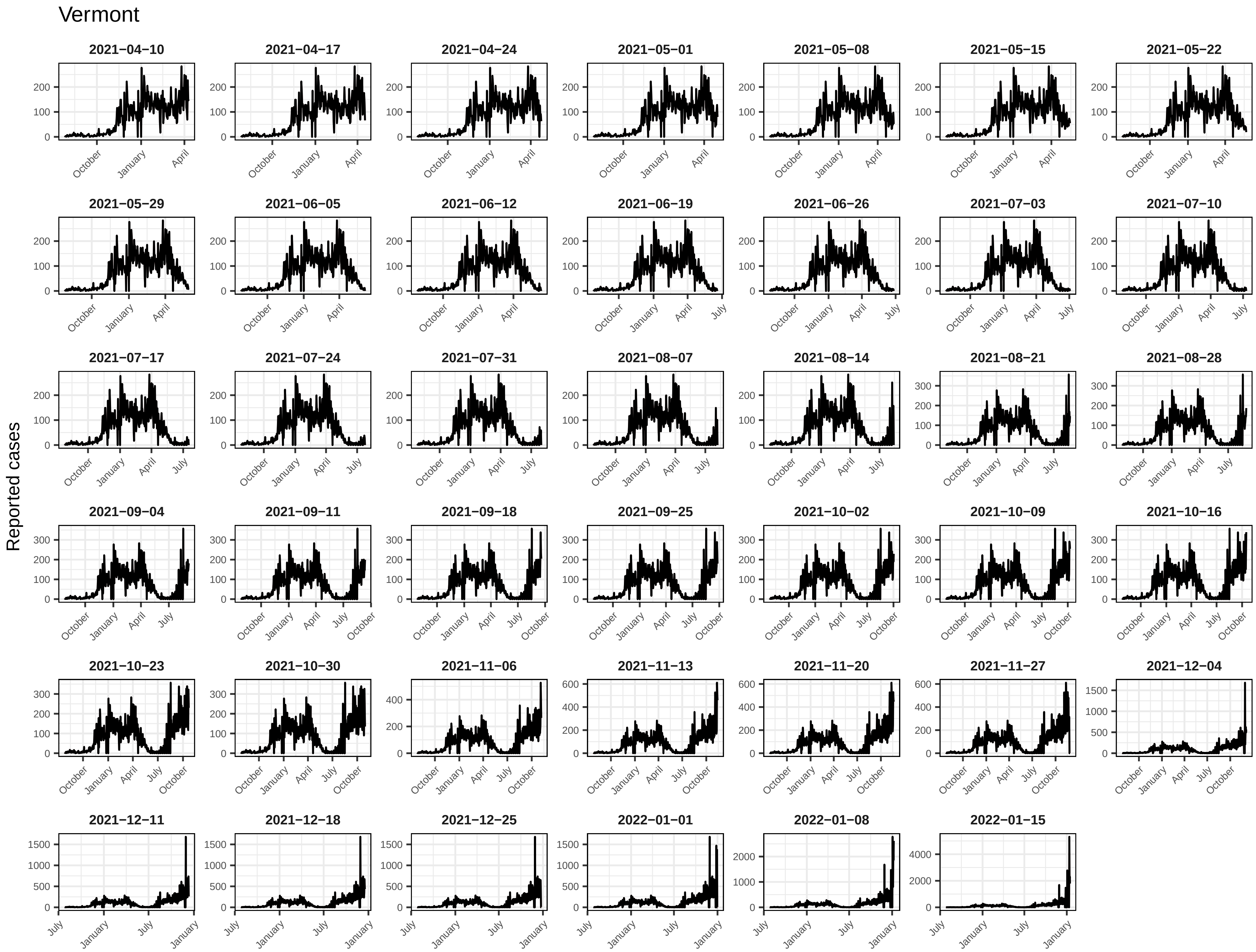
Vermont
Reported cases
2021−04−10
2021−04−17
2021−04−24
2021−05−01
2021−05−08
2021−05−15
2021−05−22
2021−05−29
2021−06−05
2021−06−12
2021−06−19
2021−06−26
2021−07−03
2021−07−10
2021−07−17
2021−07−24
2021−07−31
2021−08−07
2021−08−14
2021−08−21
2021−08−28
2021−09−04
2021−09−11
2021−09−18
2021−09−25
2021−10−02
2021−10−09
2021−10−16
2021−10−23
2021−10−30
2021−11−06
2021−11−13
2021−11−20
2021−11−27
2021−12−04
2021−12−11
2021−12−18
2021−12−25
2022−01−01
2022−01−08
2022−01−15

Virginia

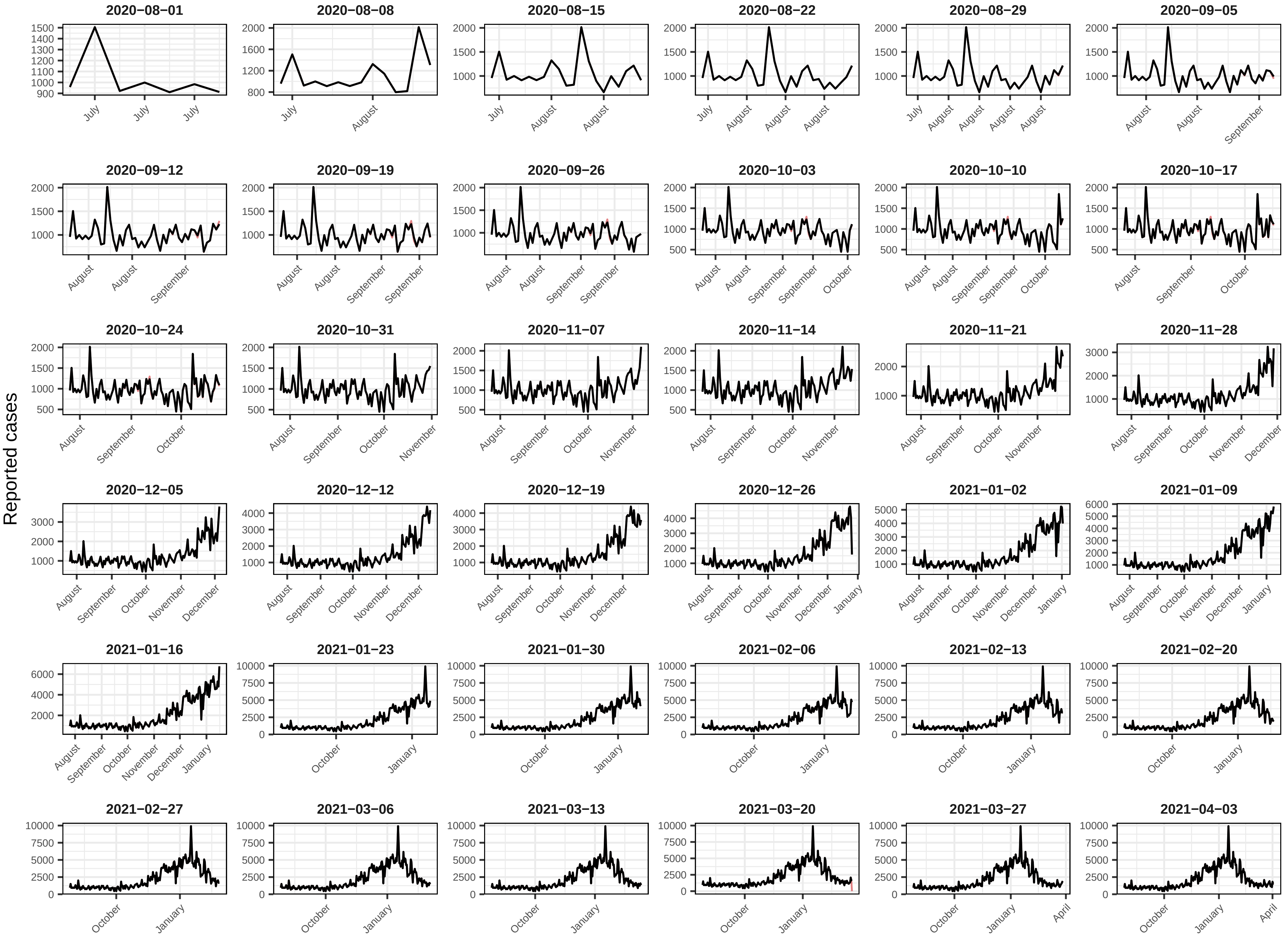

Reported cases
2020−08−01
2020−08−08
2020−08−15
2020−08−22
2020−08−29
2020−09−05
2020−09−12
2020−09−19
2020−09−26
2020−10−03
2020−10−10
2020−10−17
2020−10−24
2020−10−31
2020−11−07
2020−11−14
2020−11−21
2020−11−28
2020−12−05
2020−12−12
2020−12−19
2020−12−26
2021−01−02
2021−01−09
2021−01−16
2021−01−23
2021−01−30
2021−02−06
2021−02−13
2021−02−20
2021−02−27
2021−03−06
2021−03−13
2021−03−20
2021−03−27
2021−04−03

Virginia

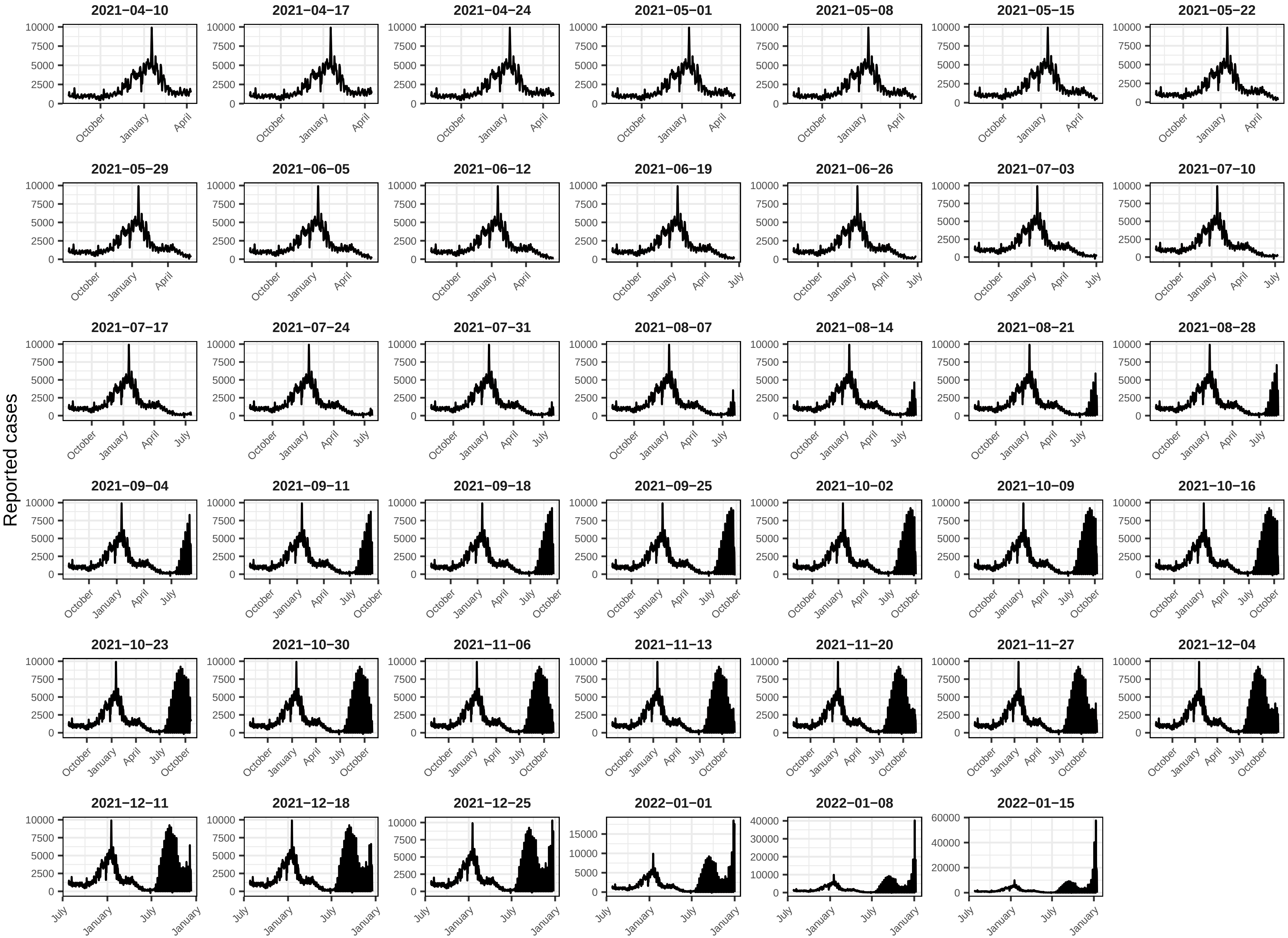
Reported cases
2021−04−10
2021−04−17
2021−04−24
2021−05−01
2021−05−08
2021−05−15
2021−05−22
2021−05−29
2021−06−05
2021−06−12
2021−06−19
2021−06−26
2021−07−03
2021−07−10
2021−07−17
2021−07−24
2021−07−31
2021−08−07
2021−08−14
2021−08−21
2021−08−28
2021−09−04
2021−09−11
2021−09−18
2021−09−25
2021−10−02
2021−10−09
2021−10−16
2021−10−23
2021−10−30
2021−11−06
2021−11−13
2021−11−20
2021−11−27
2021−12−04
2021−12−11
2021−12−18
2021−12−25
2022−01−01
2022−01−08
2022−01−15
July
October
January
April

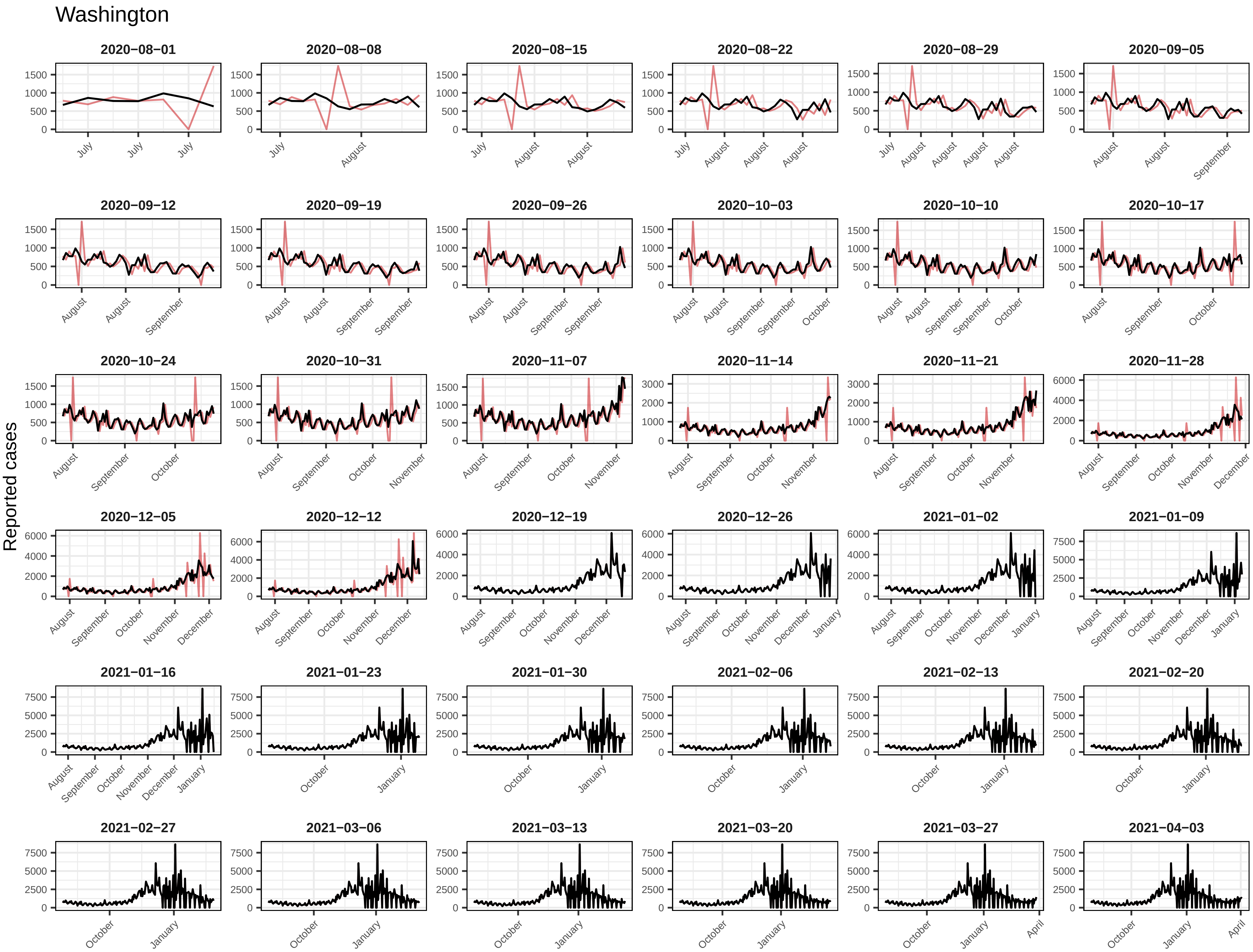
Washington
Reported cases
2020−08−01
2020−08−08
2020−08−15
2020−08−22
2020−08−29
2020−09−05
2020−09−12
2020−09−19
2020−09−26
2020−10−03
2020−10−10
2020−10−17
2020−10−24
2020−10−31
2020−11−07
2020−11−14
2020−11−21
2020−11−28
2020−12−05
2020−12−12
2020−12−19
2020−12−26
2021−01−02
2021−01−09
2021−01−16
2021−01−23
2021−01−30
2021−02−06
2021−02−13
2021−02−20
2021−02−27
2021−03−06
2021−03−13
2021−03−20
2021−03−27
2021−04−03

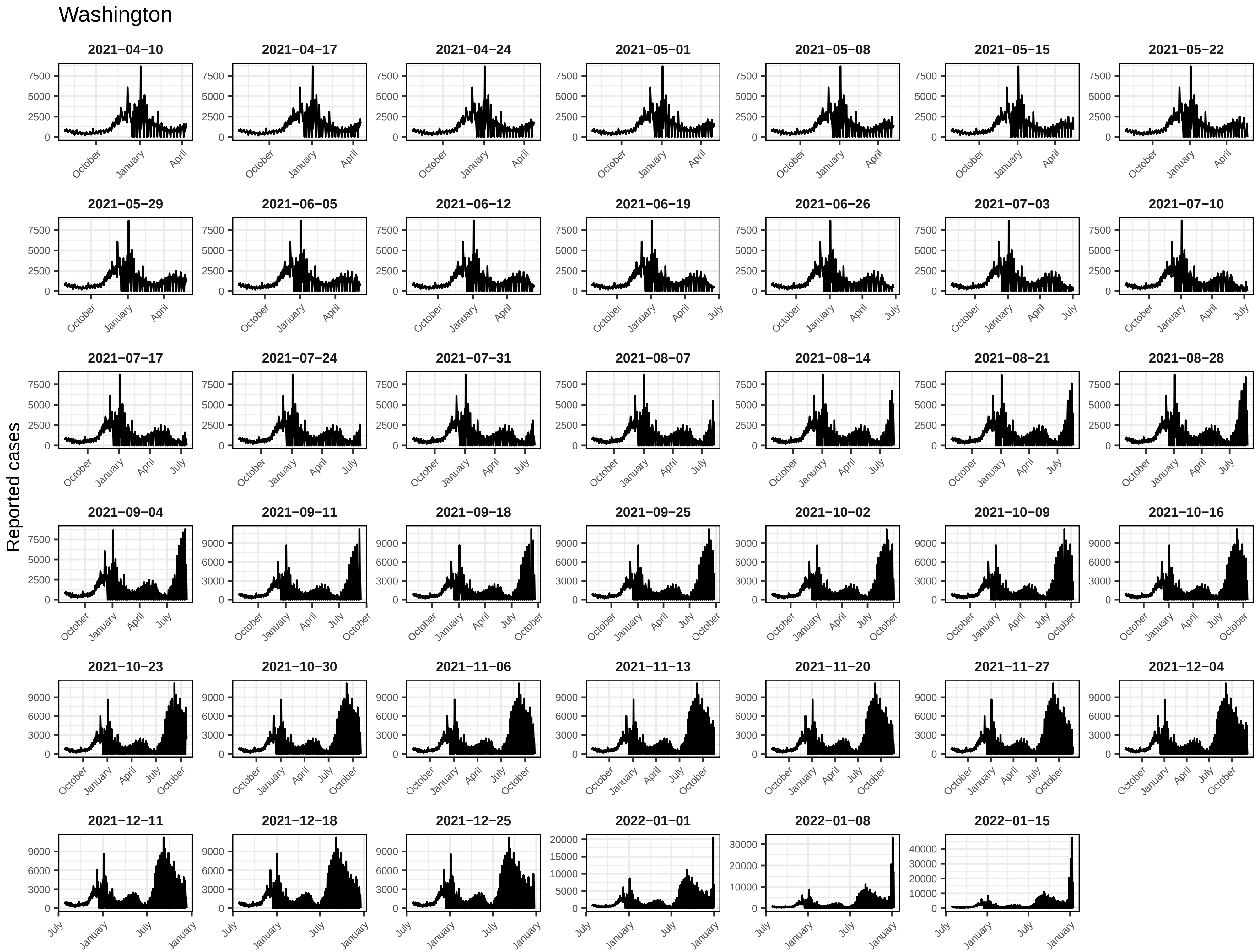
Washington
Reported cases
2021−04−10
2021−04−17
2021−04−24
2021−05−01
2021−05−08
2021−05−15
2021−05−22
2021−05−29
2021−06−05
2021−06−12
2021−06−19
2021−06−26
2021−07−03
2021−07−10
2021−07−17
2021−07−24
2021−07−31
2021−08−07
2021−08−14
2021−08−21
2021−08−28
2021−09−04
2021−09−11
2021−09−18
2021−09−25
2021−10−02
2021−10−09
2021−10−16
2021−10−23
2021−10−30
2021−11−06
2021−11−13
2021−11−20
2021−11−27
2021−12−04
2021−12−11
2021−12−18
2021−12−25
2022−01−01
2022−01−08
2022−01−15

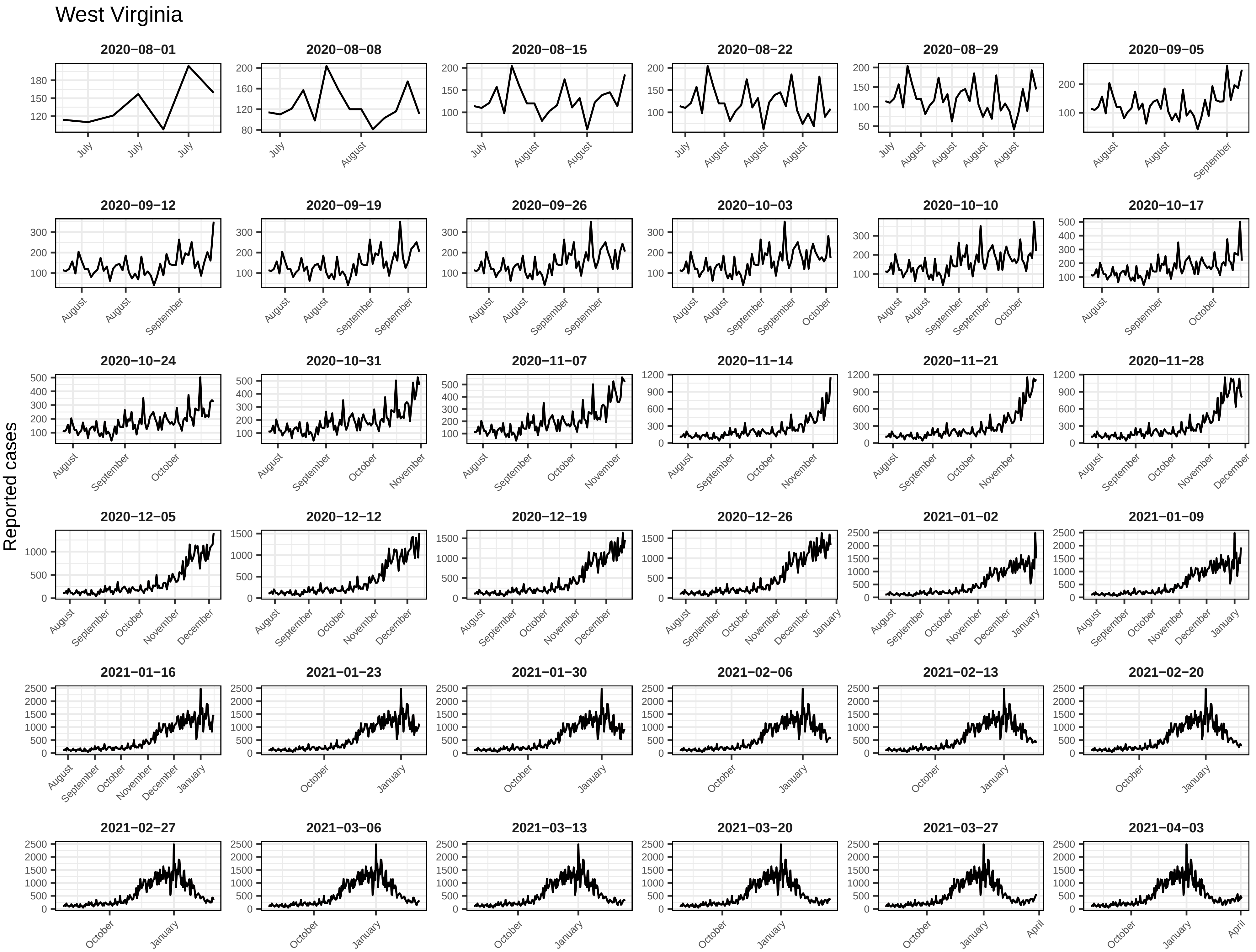

West Virginia
Reported cases
2020−08−01
2020−08−08
2020−08−15
2020−08−22
2020−08−29
2020−09−05
2020−09−12
2020−09−19
2020−09−26
2020−10−03
2020−10−10
2020−10−17
2020−10−24
2020−10−31
2020−11−07
2020−11−14
2020−11−21
2020−11−28
2020−12−05
2020−12−12
2020−12−19
2020−12−26
2021−01−02
2021−01−09
2021−01−16
2021−01−23
2021−01−30
2021−02−06
2021−02−13
2021−02−20
2021−02−27
2021−03−06
2021−03−13
2021−03−20
2021−03−27
2021−04−03

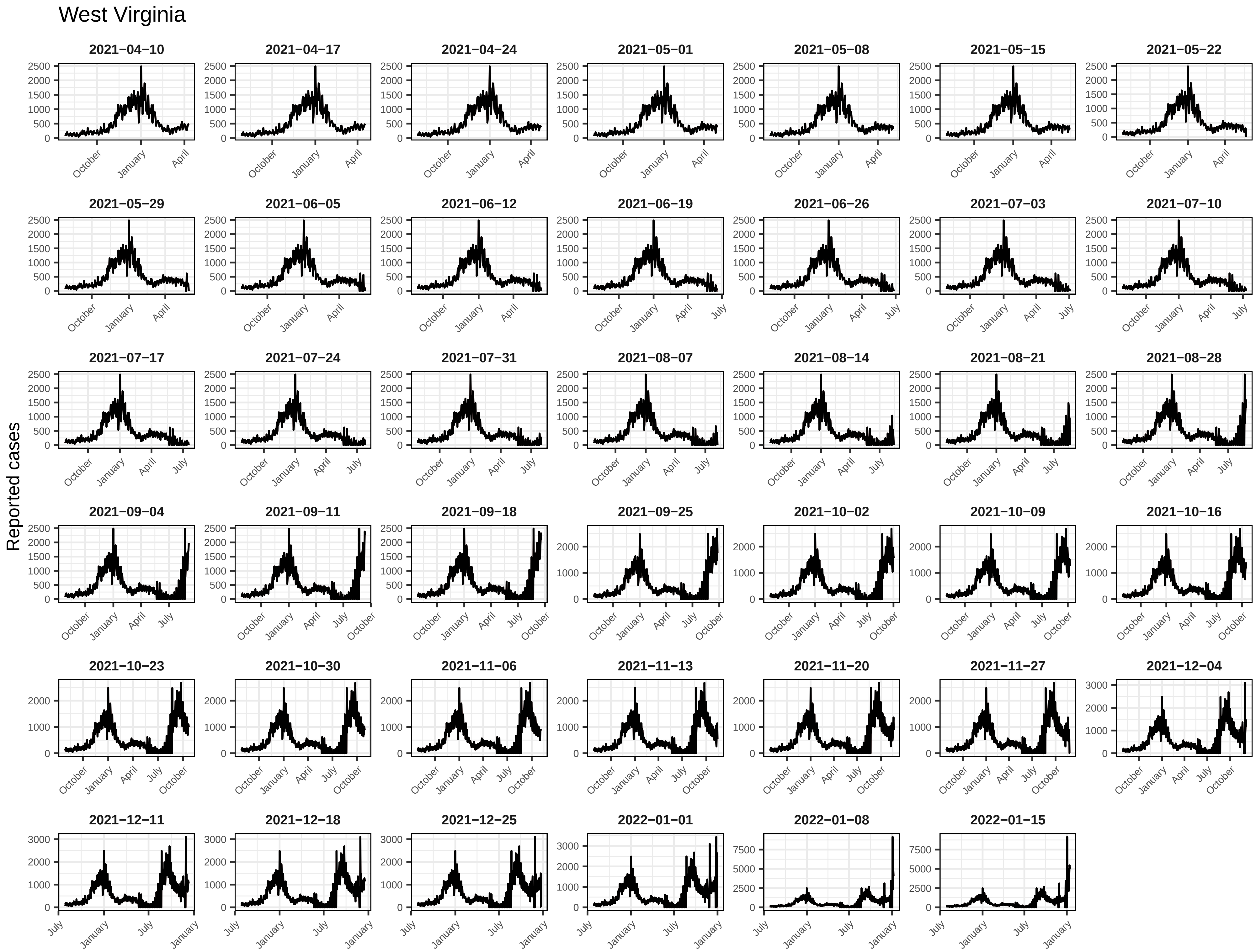
West Virginia
Reported cases
2021−04−10
2021−04−17
2021−04−24
2021−05−01
2021−05−08
2021−05−15
2021−05−22
2021−05−29
2021−06−05
2021−06−12
2021−06−19
2021−06−26
2021−07−03
2021−07−10
2021−07−17
2021−07−24
2021−07−31
2021−08−07
2021−08−14
2021−08−21
2021−08−28
2021−09−04
2021−09−11
2021−09−18
2021−09−25
2021−10−02
2021−10−09
2021−10−16
2021−10−23
2021−10−30
2021−11−06
2021−11−13
2021−11−20
2021−11−27
2021−12−04
2021−12−11
2021−12−18
2021−12−25
2022−01−01
2022−01−08
2022−01−15
October
January
April
July

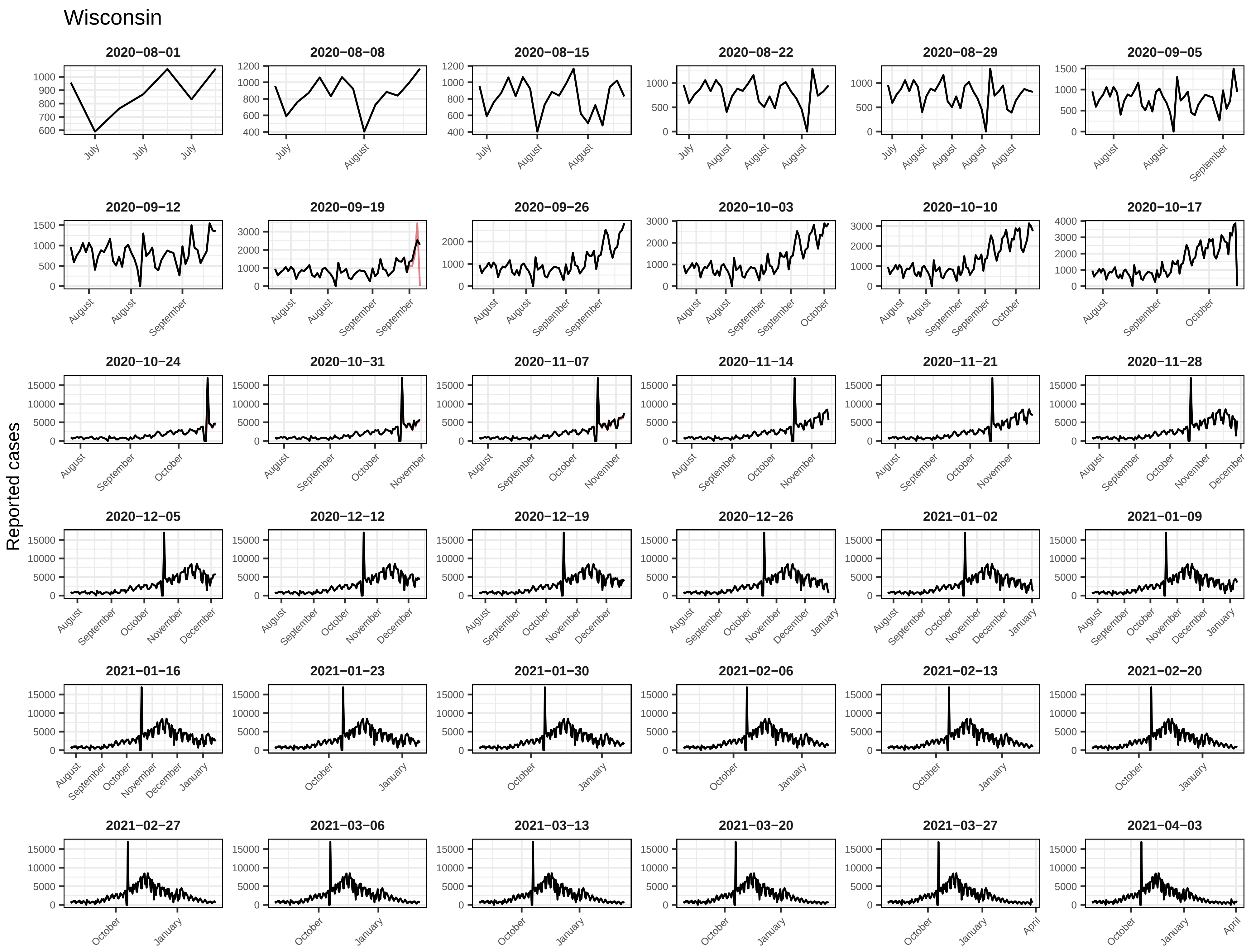
Wisconsin
Reported cases
2020−08−01
2020−08−08
2020−08−15
2020−08−22
2020−08−29
2020−09−05
2020−09−12
2020−09−19
2020−09−26
2020−10−03
2020−10−10
2020−10−17
2020−10−24
2020−10−31
2020−11−07
2020−11−14
2020−11−21
2020−11−28
2020−12−05
2020−12−12
2020−12−19
2020−12−26
2021−01−02
2021−01−09
2021−01−16
2021−01−23
2021−01−30
2021−02−06
2021−02−13
2021−02−20
2021−02−27
2021−03−06
2021−03−13
2021−03−20
2021−03−27
2021−04−03

Wisconsin

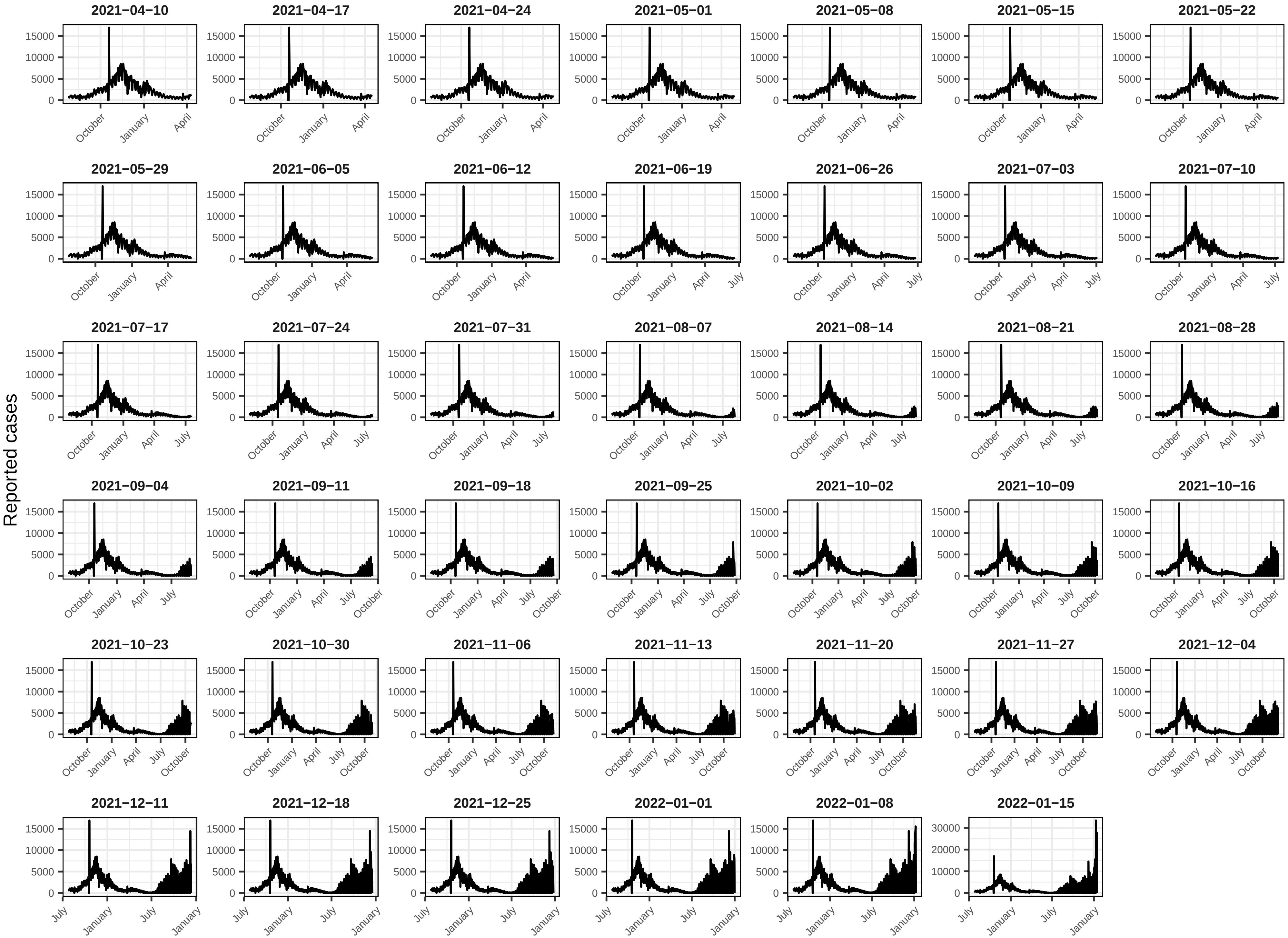

Reported cases
2021−04−10
2021−04−17
2021−04−24
2021−05−01
2021−05−08
2021−05−15
2021−05−22
2021−05−29
2021−06−05
2021−06−12
2021−06−19
2021−06−26
2021−07−03
2021−07−10
2021−07−17
2021−07−24
2021−07−31
2021−08−07
2021−08−14
2021−08−21
2021−08−28
2021−09−04
2021−09−11
2021−09−18
2021−09−25
2021−10−02
2021−10−09
2021−10−16
2021−10−23
2021−10−30
2021−11−06
2021−11−13
2021−11−20
2021−11−27
2021−12−04
2021−12−11
2021−12−18
2021−12−25
2022−01−01
2022−01−08
2022−01−15

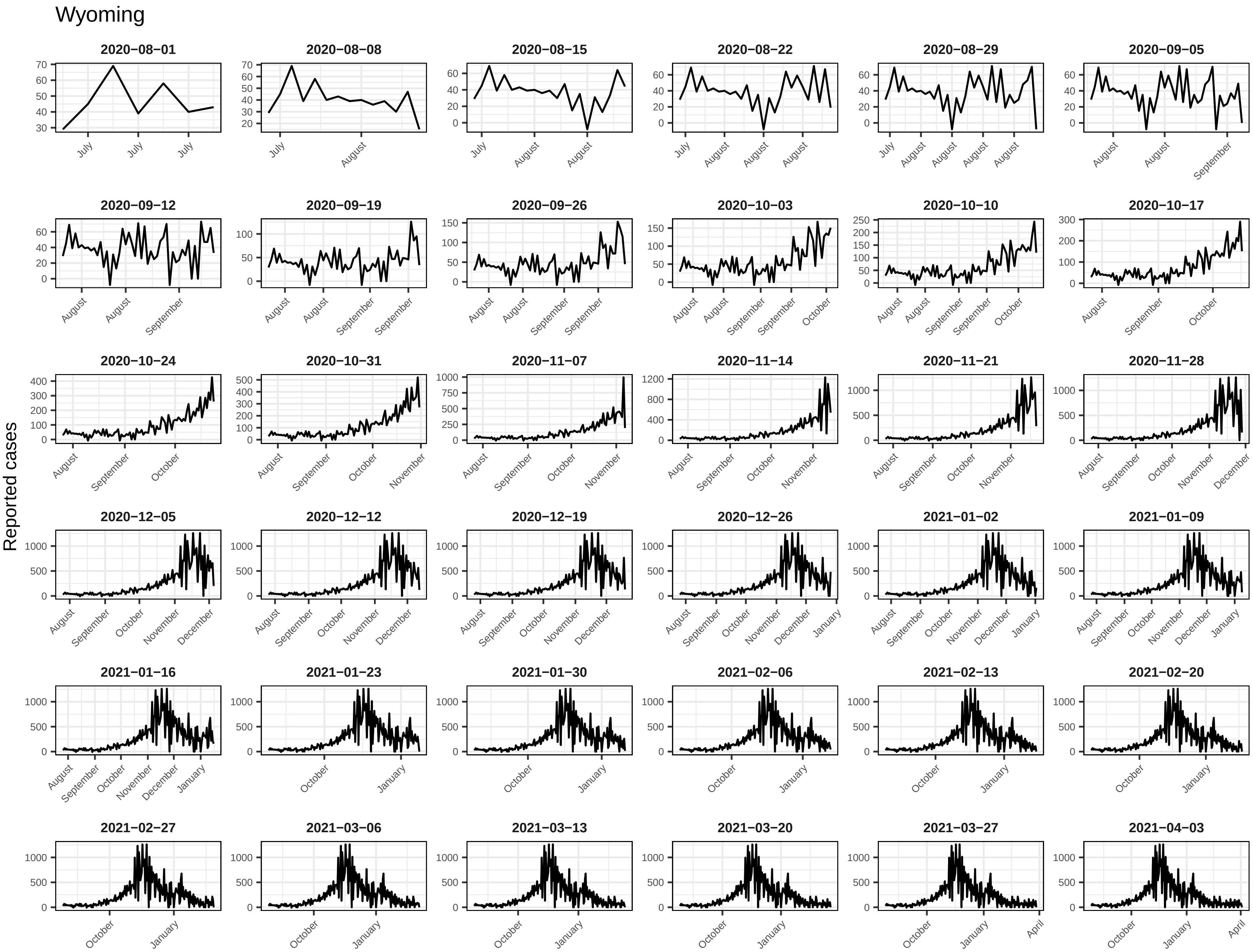
Wyoming
Reported cases
2020−08−01
2020−08−08
2020−08−15
2020−08−22
2020−08−29
2020−09−05
2020−09−12
2020−09−19
2020−09−26
2020−10−03
2020−10−10
2020−10−17
2020−10−24
2020−10−31
2020−11−07
2020−11−14
2020−11−21
2020−11−28
2020−12−05
2020−12−12
2020−12−19
2020−12−26
2021−01−02
2021−01−09
2021−01−16
2021−01−23
2021−01−30
2021−02−06
2021−02−13
2021−02−20
2021−02−27
2021−03−06
2021−03−13
2021−03−20
2021−03−27
2021−04−03

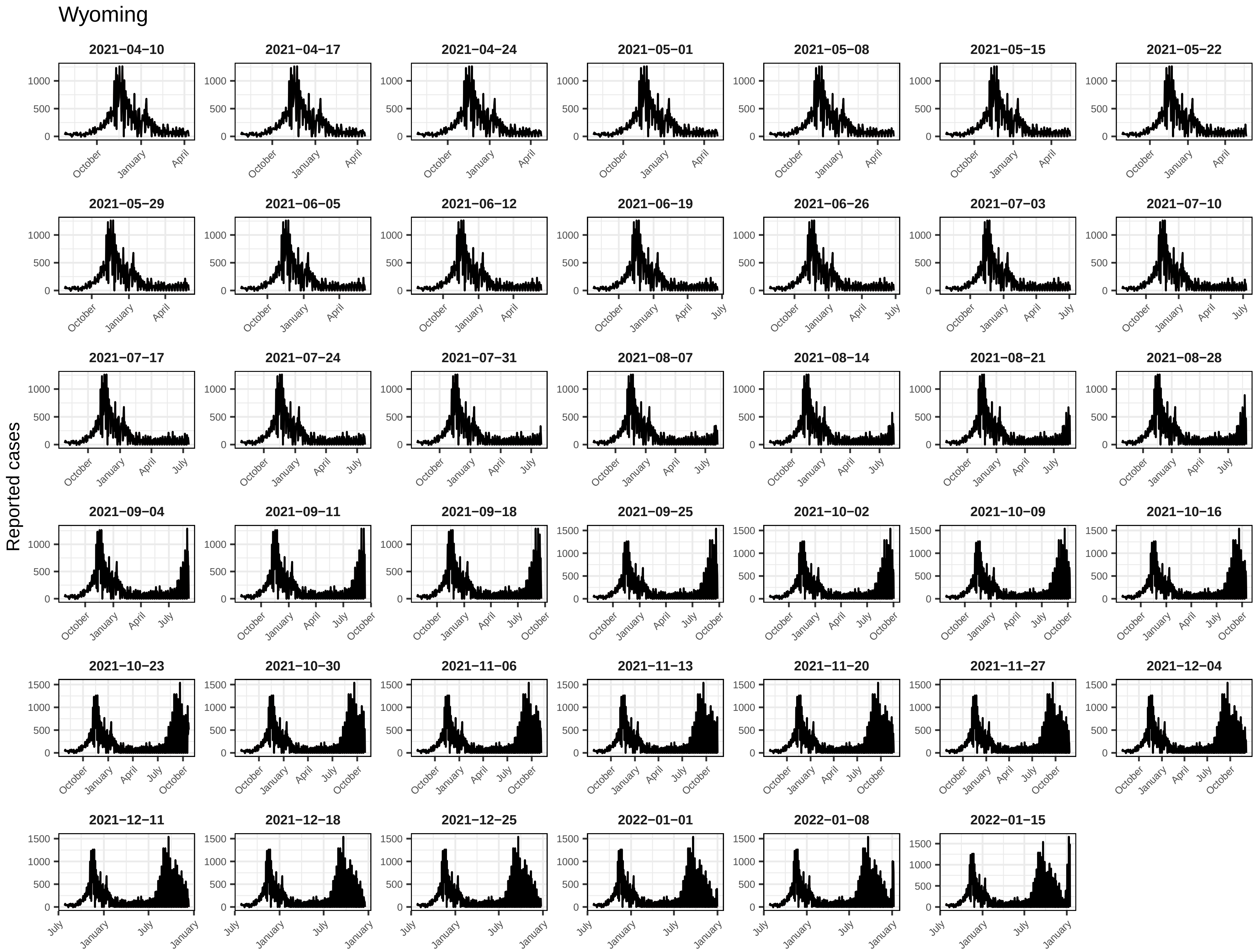

Wyoming
Reported cases
2021−04−10
2021−04−17
2021−04−24
2021−05−01
2021−05−08
2021−05−15
2021−05−22
2021−05−29
2021−06−05
2021−06−12
2021−06−19
2021−06−26
2021−07−03
2021−07−10
2021−07−17
2021−07−24
2021−07−31
2021−08−07
2021−08−14
2021−08−21
2021−08−28
2021−09−04
2021−09−11
2021−09−18
2021−09−25
2021−10−02
2021−10−09
2021−10−16
2021−10−23
2021−10−30
2021−11−06
2021−11−13
2021−11−20
2021−11−27
2021−12−04
2021−12−11
2021−12−18
2021−12−25
2022−01−01
2022−01−08
2022−01−15
0
500
1000
1500
July
October
January
April

S2 Fig B. After identifying weeks with revised case counts, we then excluded them from the dataset and reran the GEE models and estimated the marginal mean Weighted Interval Score (WIS). Panel 1 shows the estimated marginal mean WIS and 95% confidence intervals for mean cases from team-specific GEE models for all 48 jurisdictions from this sensitivity analysis. The 95% confidence intervals for the COVIDhub-baseline model are shown in dashed red vertical lines. Panel 2 presents each team's estimated marginal mean WIS per phase, scaled to the COVIDhub-baseline model's estimated marginal mean WIS for all epidemic phases, using the dataset with excluded week. Teams with higher estimated marginal mean WIS values (i.e., greater than 1.0) are presented in shades of orange while teams with lower estimated marginal mean WIS (i.e., less than 1.0) are shown in shades of green**.** Team forecasts are denoted with an asterisk (*) if the 80% confidence interval of the expected WIS outcome (normalized and on the log scale) was estimated by a model to be lower than the expected WIS of the COVIDhub-baseline model for all phases.

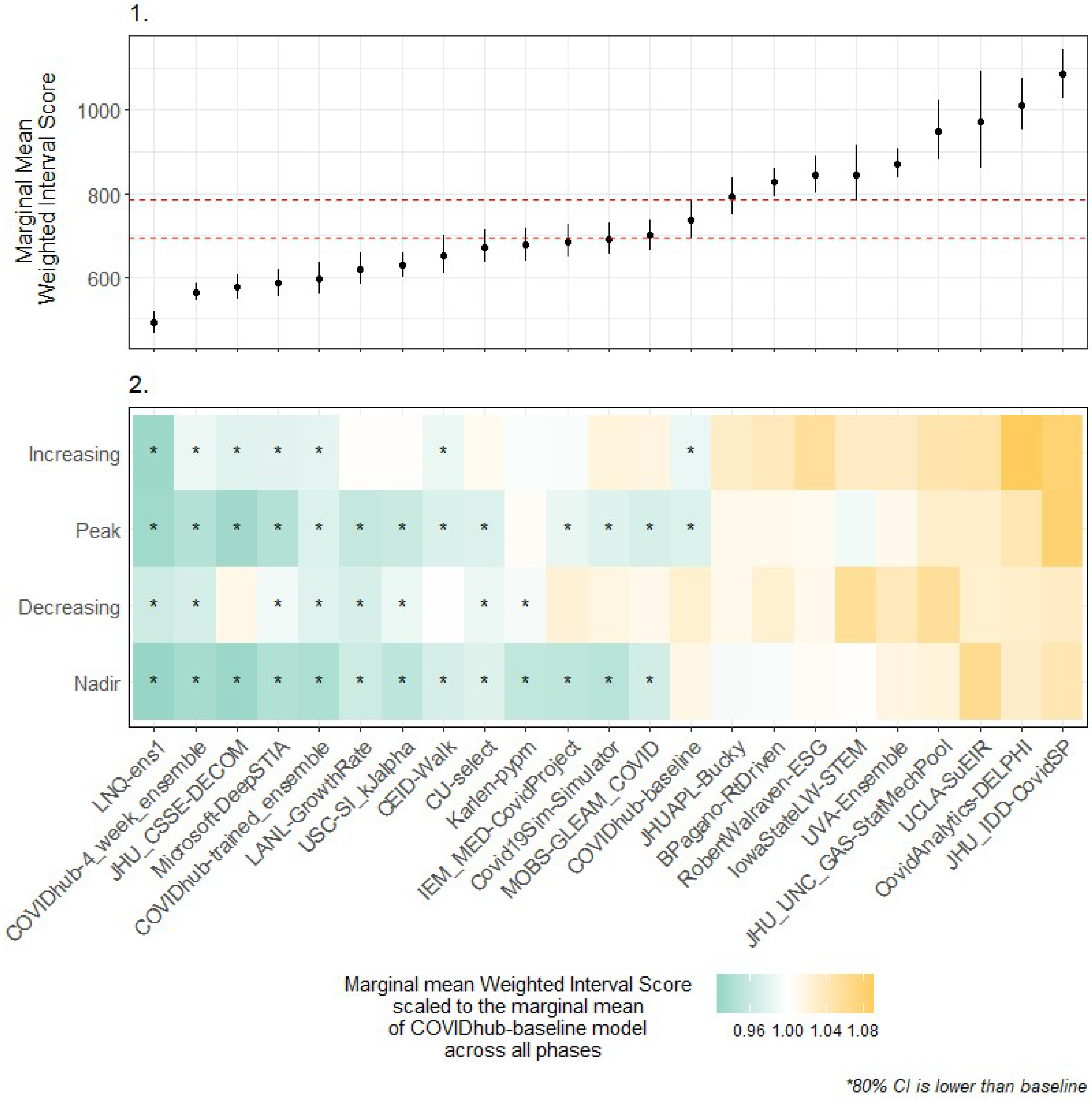

S2 Fig C. Outliers were defined as non-revised reported case counts that were outside of the expected range by at least two of the three algorithms: a rolling median, a seasonal trend decomposition, and a seasonal trend decomposition without a seasonality term. Each method used a 21-day window. Approximately three percent of weeks (686 of 27,489 total weeks in the analysis period) had at least one day of reported cases identified as an outlier.

After identifying outlier counts, we then excluded weeks with outliers and the week following an outlier from the dataset and reran the GEE models and estimated the marginal mean Weighted Interval Score (WIS). Panel 1 shows the estimated marginal mean WIS and 95% confidence intervals for mean cases from team-specific GEE models for all 51 jurisdictions from this sensitivity analysis. The 95% confidence intervals for the COVIDhub-baseline model are shown in dashed red vertical lines. Panel 2 presents each team's estimated marginal mean WIS per phase, scaled to the COVIDhub-baseline model's estimated marginal mean WIS for all epidemic phases, using the dataset with excluded week. Teams with higher estimated marginal mean WIS values (i.e., greater than 1.0) are presented in shades of orange while teams with lower estimated marginal mean WIS (i.e., less than 1.0) are shown in shades of green**.** WIS values are denoted with an asterisk (*) if the 80% confidence interval of the expected WIS outcome (normalized and on the log scale) was estimated by a model to be lower than the expected WIS of the COVIDhub-baseline model for all phases.

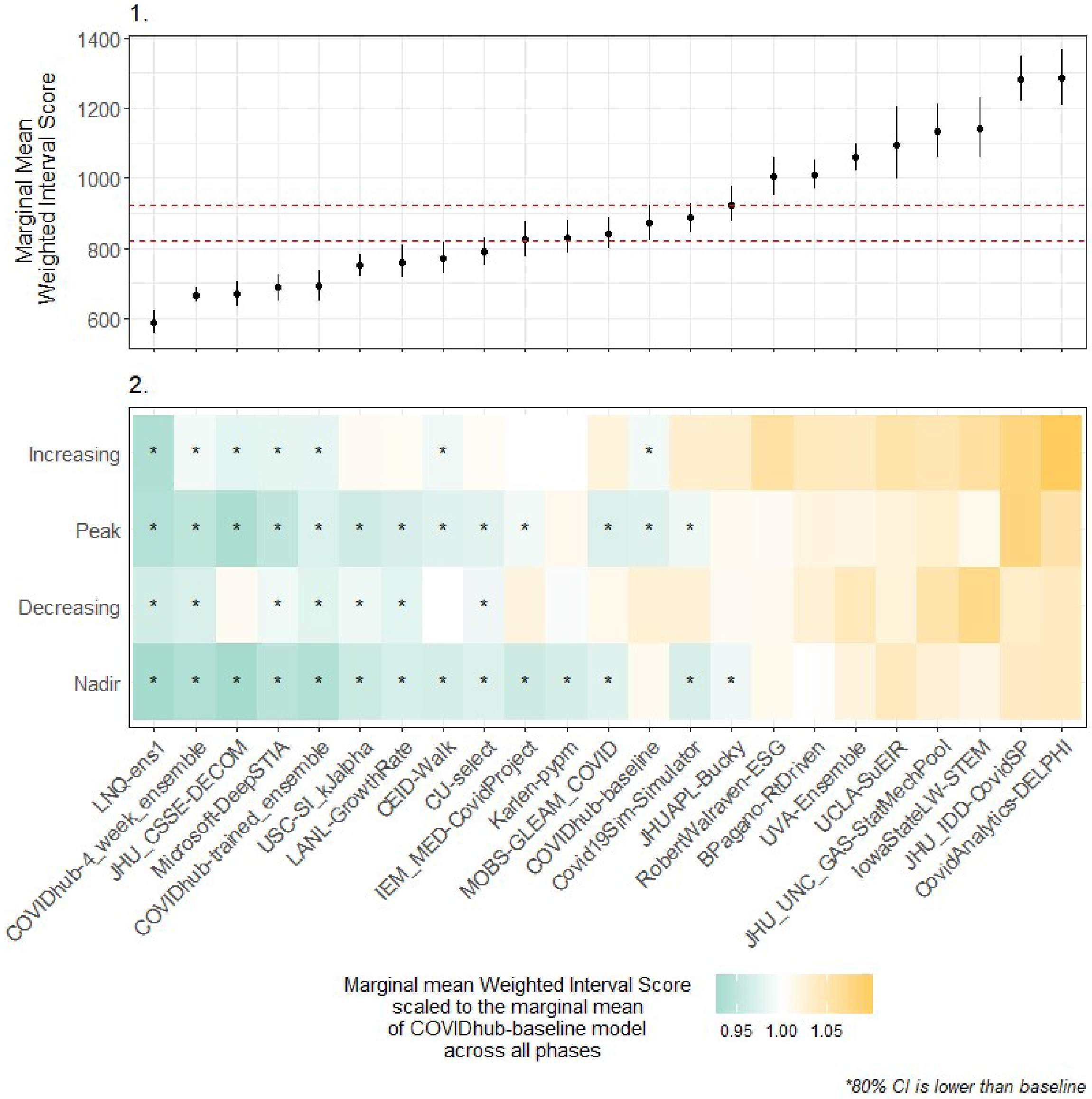

Supplement: S2 Appendix — Fig A. To assess the influence of data revisions on our evaluation of forecast skill, we compared daily differences in cumulative reported cases during the week they were first reported to reported case counts for the same week in the complete data as of April 2, 2022. In total 721 weeks had at least one day with a revised case count (17% of all weeks, n = 4,241 weeks) and revisions occurred in 43 of 51 jurisdictions. These jurisdiction specific plots compare cases reported as of the date in the subtitle (in red) compared to cases reported as of April 2, 2022 (in black). Fig B. After identifying weeks with revised case counts, we then excluded them from the dataset and reran the GEE models and estimated the marginal mean Weighted Interval Score (WIS). Panel 1 shows the estimated marginal mean WIS and 95% confidence intervals for mean cases from team-specific GEE models for all 48 jurisdictions from this sensitivity analysis. The 95% confidence intervals for the COVIDhub-baseline model are shown in dashed red vertical lines. Panel 2 presents each team’s estimated marginal mean WIS per phase, scaled to the COVIDhub-baseline model’s estimated marginal mean WIS for all epidemic phases, using the dataset with excluded weeks. Teams with higher estimated marginal mean WIS values (i.e., greater than 1.0) are presented in shades of orange while teams with lower estimated marginal mean WIS (i.e., less than 1.0) are shown in shades of green. Team forecasts are denoted with an asterisk (*) if the 80% confidence interval of the expected WIS outcome (normalized and on the log scale) was estimated by a model to be lower than the average expected WIS of the COVIDhub-baseline model across all phases. Fig C. Outliers were defined as non-revised reported case counts that were outside of the expected range by at least two of the three algorithms: a rolling median, a seasonal trend decomposition, and a seasonal trend decomposition without a seasonality term. Each method used a 21-day wi [file pcbi.1011200.s002.pdf]
