# Supplementary material for: Challenges of COVID-19 Case Forecasting in the US, 2020–2021
Source: PLoS Comput Biol. 2024 May 6;20(5):e1011200. doi: 10.1371/journal.pcbi.1011200 (PMC11098513; doi:10.1371/journal.pcbi.1011200)

**S4 Appendix.** Spatial correlation between forecasts skill

**Fig A**. Moran’s I for each team’s state-level scaled, pairwise relative Weighted Interval Score (see *Methods* for description) in the contiguous United States. Moran’s I was estimated using queen’s contingency and equal weights for nearest neighbors.


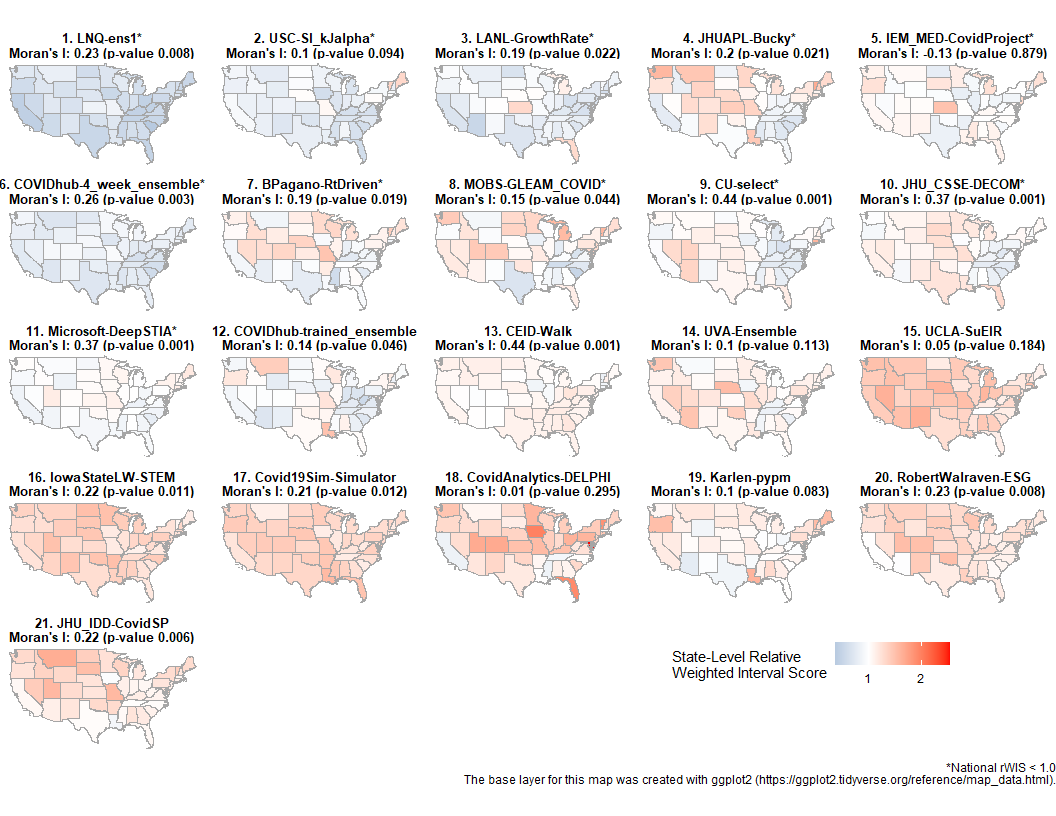

Supplement: S4 Appendix — Fig A. Moran’s I for each team’s state-level relative Weighted Interval Score in the contiguous United States. (DOCX) [file pcbi.1011200.s004.docx]
