# Supplementary material for: Challenges of COVID-19 Case Forecasting in the US, 2020–2021
Source: PLoS Comput Biol. 2024 May 6;20(5):e1011200. doi: 10.1371/journal.pcbi.1011200 (PMC11098513; doi:10.1371/journal.pcbi.1011200)
Supplement: S5 Appendix — Proportion of weeks in each classified epidemic phase (Fig A), and the estimated time-varying reproduction number and epidemic phase classifications (Fig B). Fig A. The proportion of weeks in each classified epidemic phase per state. Fig B. For each state, the top panel shows the median Rt and median upper and lower 90% credible interval over time in red. The bottom panel shows reported case counts over time. Both plots have vertical bands representing the epidemic phase of each forecast week: increasing, peak, decreasing, nadir. (PDF) [file pcbi.1011200.s005.pdf]

<u>Supporting Information 5</u>: Proportion of weeks in each classified epidemic phase (S5 Figure A), and the Estimated time-varying reproduction number and epidemic phase classifications (S5 Figure B). For each state, the top panel shows the median R_t and median upper and lower 90% credible interval over time in red. The bottom panel shows reported case counts over time. Both plots have vertical bands representing the epidemic phase of each forecast week: *increasing*, *peak*, *decreasing*, *nadir.*

S5 Fig A. The proportion of weeks in each classified epidemic phase per state.

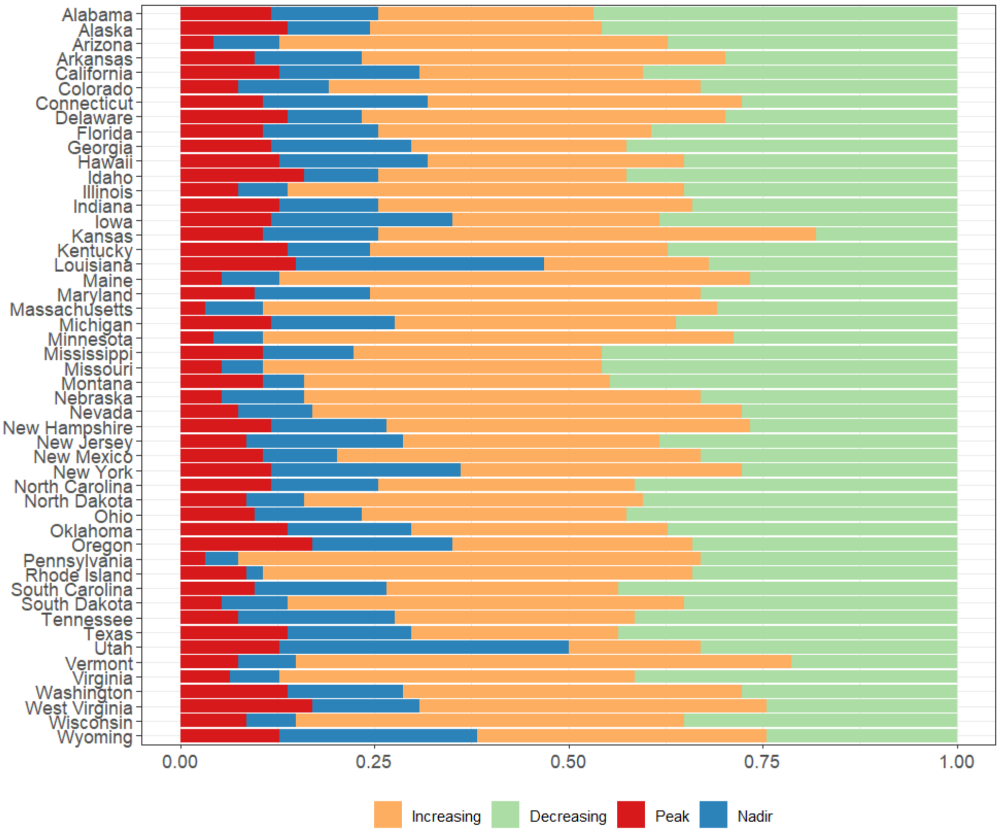

S5 Fig B. For each state, the top panel shows the median R_t and median upper and lower 90% credible interval over time in red. The bottom panel shows reported case counts over time. Both plots have vertical bands representing the epidemic phase of each forecast week: *increasing*, *peak*, *decreasing*, *nadir.*

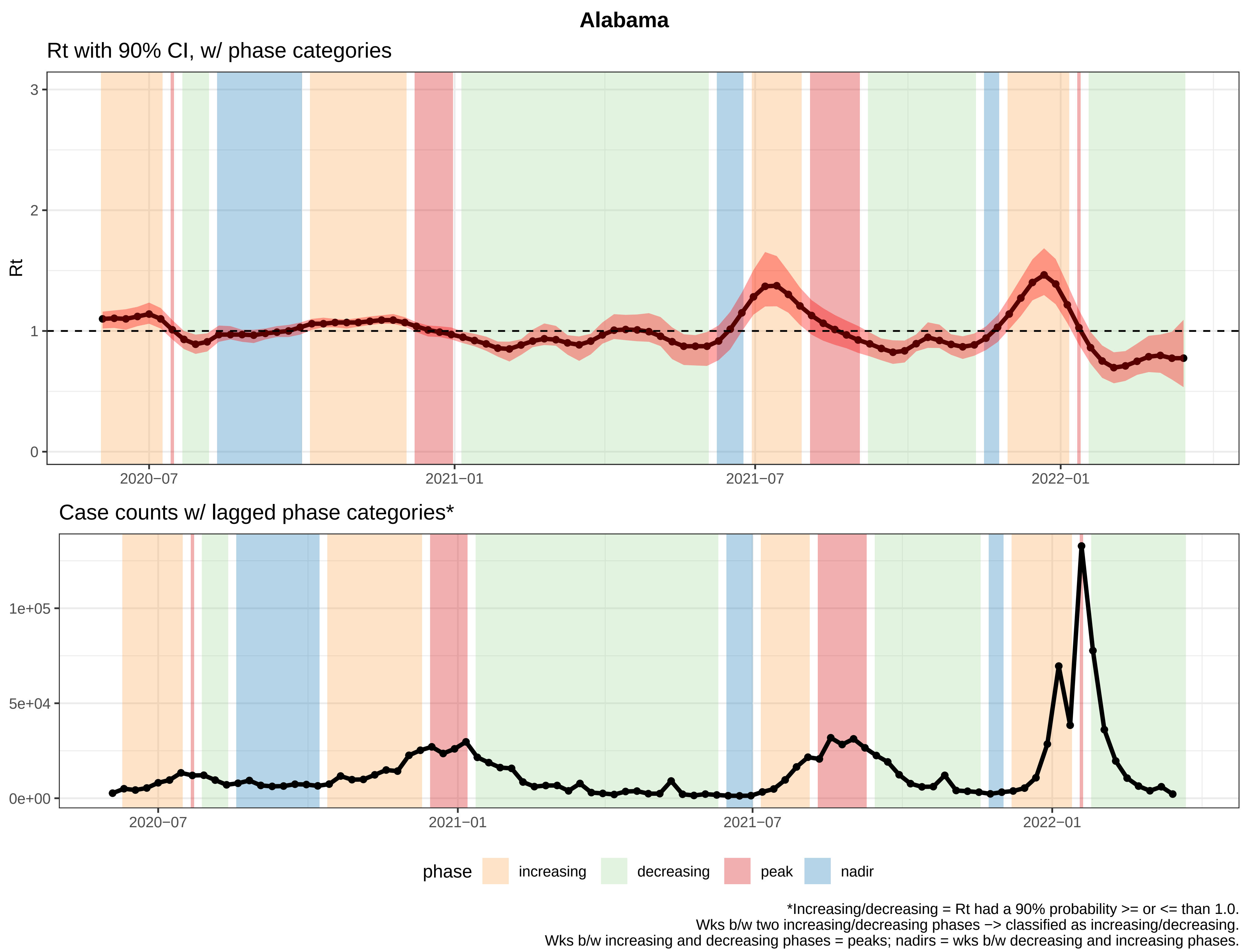
Alabama
Rt with 90% CI, w/ phase categories
Rt
0
1
2
3
2020−07
2021−01
2021−07
2022−01
Case counts w/ lagged phase categories*
0e+00
5e+04
1e+05
2020−07
2021−01
2021−07
2022−01
phase
increasing
decreasing
peak
nadir
*Increasing/decreasing = Rt had a 90% probability >= or <= than 1.0.
Wks b/w two increasing/decreasing phases −> classified as increasing/decreasing.
Wks b/w increasing and decreasing phases = peaks; nadirs = wks b/w decreasing and increasing phases.

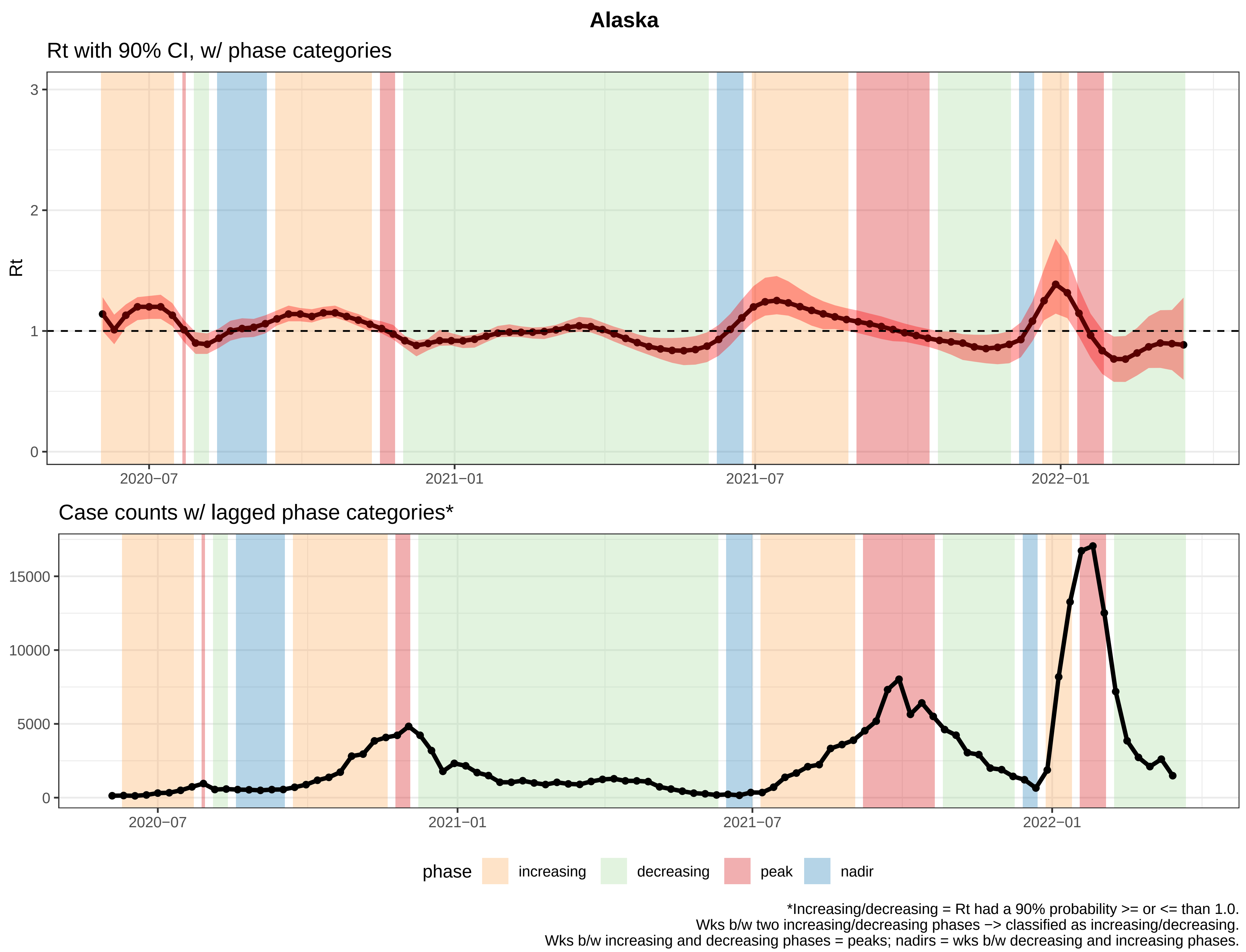
Alaska
Rt with 90% CI, w/ phase categories
Rt
0
1
2
3
2020−07
2021−01
2021−07
2022−01
Case counts w/ lagged phase categories*
0
5000
10000
15000
2020−07
2021−01
2021−07
2022−01
phase
increasing
decreasing
peak
nadir
*Increasing/decreasing = Rt had a 90% probability >= or <= than 1.0.
Wks b/w two increasing/decreasing phases −> classified as increasing/decreasing.
Wks b/w increasing and decreasing phases = peaks; nadirs = wks b/w decreasing and increasing phases.

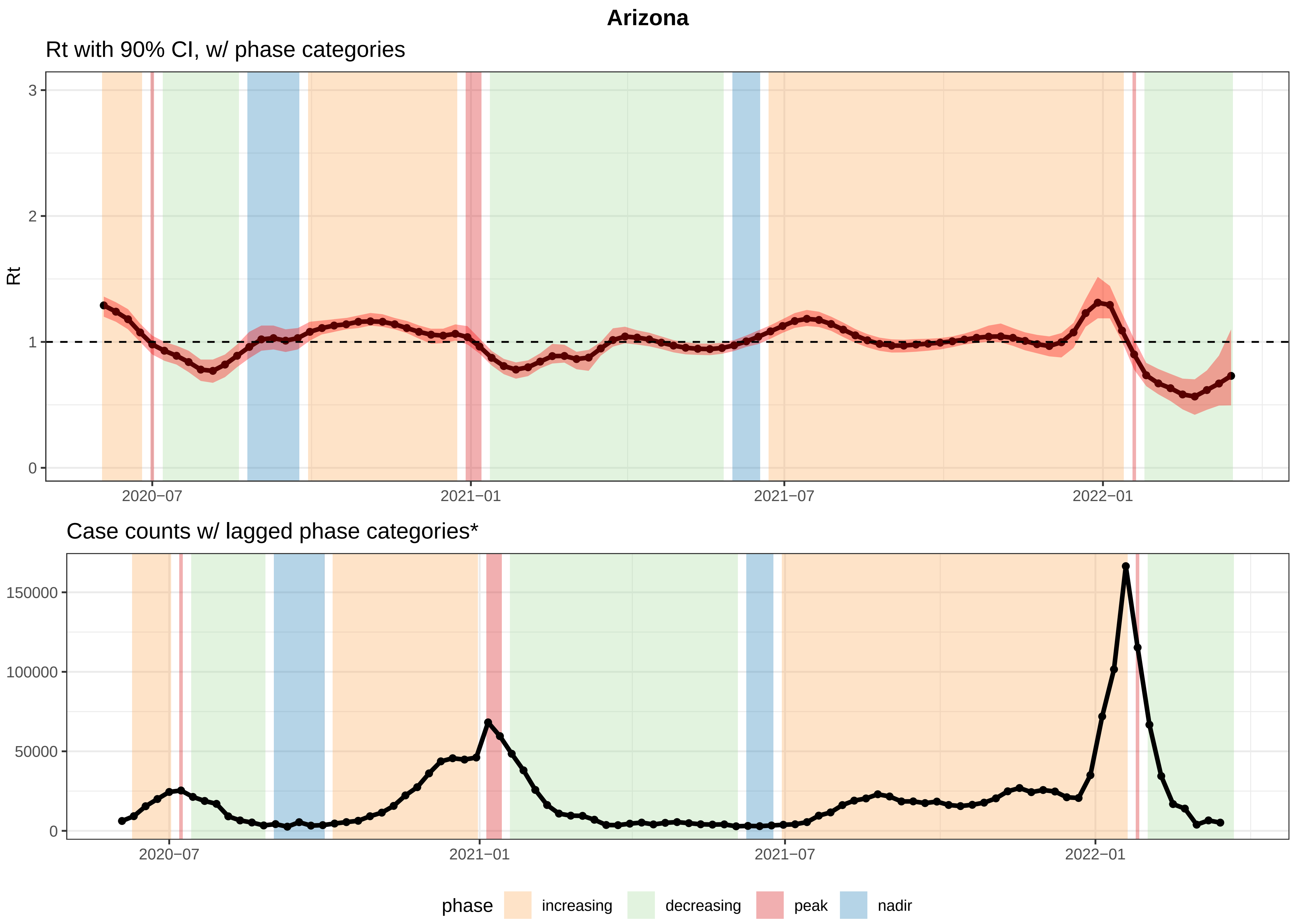

*Increasing/decreasing = Rt had a 90% probability >= or <= than 1.0.
Wks b/w two increasing/decreasing phases −> classified as increasing/decreasing.
Wks b/w increasing and decreasing phases = peaks; nadirs = wks b/w decreasing and increasing phases.

Arkansas

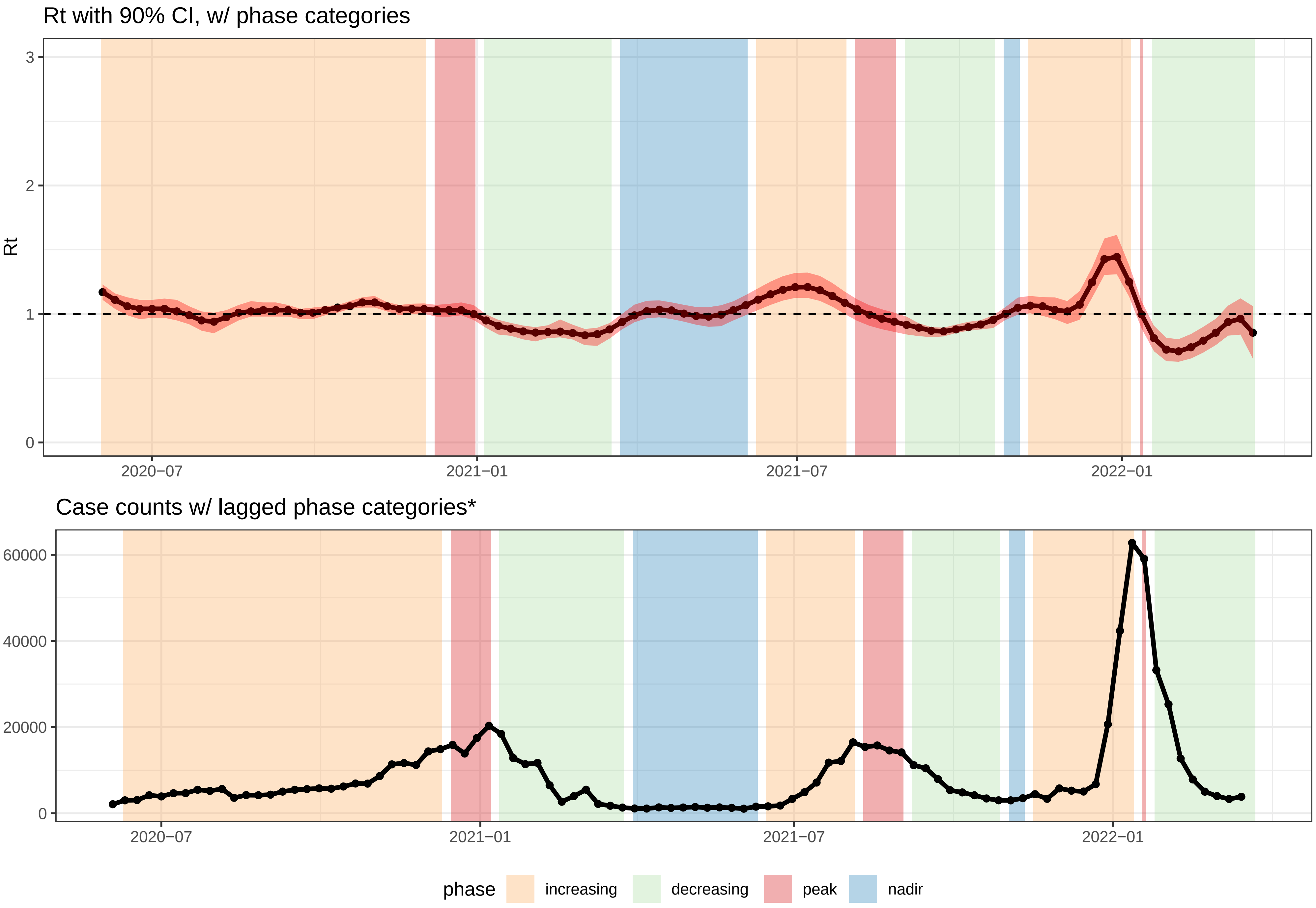

*Increasing/decreasing = Rt had a 90% probability >= or <= than 1.0.
Wks b/w two increasing/decreasing phases −> classified as increasing/decreasing.
Wks b/w increasing and decreasing phases = peaks; nadirs = wks b/w decreasing and increasing phases.

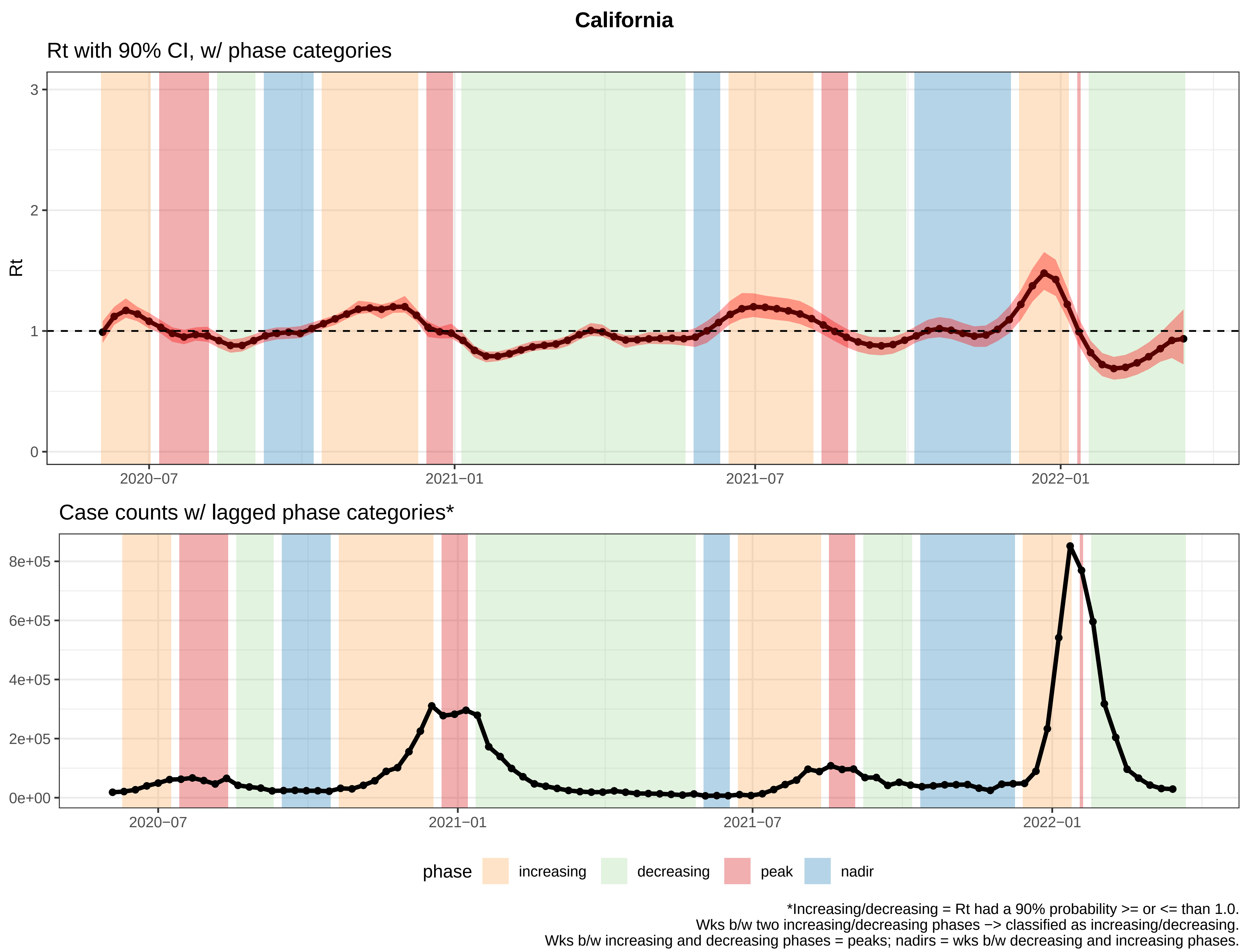
California
Rt with 90% CI, w/ phase categories
Rt
0
1
2
3
2020−07
2021−01
2021−07
2022−01
Case counts w/ lagged phase categories*
0e+00
2e+05
4e+05
6e+05
8e+05
2020−07
2021−01
2021−07
2022−01
phase
increasing
decreasing
peak
nadir
*Increasing/decreasing = Rt had a 90% probability >= or <= than 1.0.
Wks b/w two increasing/decreasing phases −> classified as increasing/decreasing.
Wks b/w increasing and decreasing phases = peaks; nadirs = wks b/w decreasing and increasing phases.

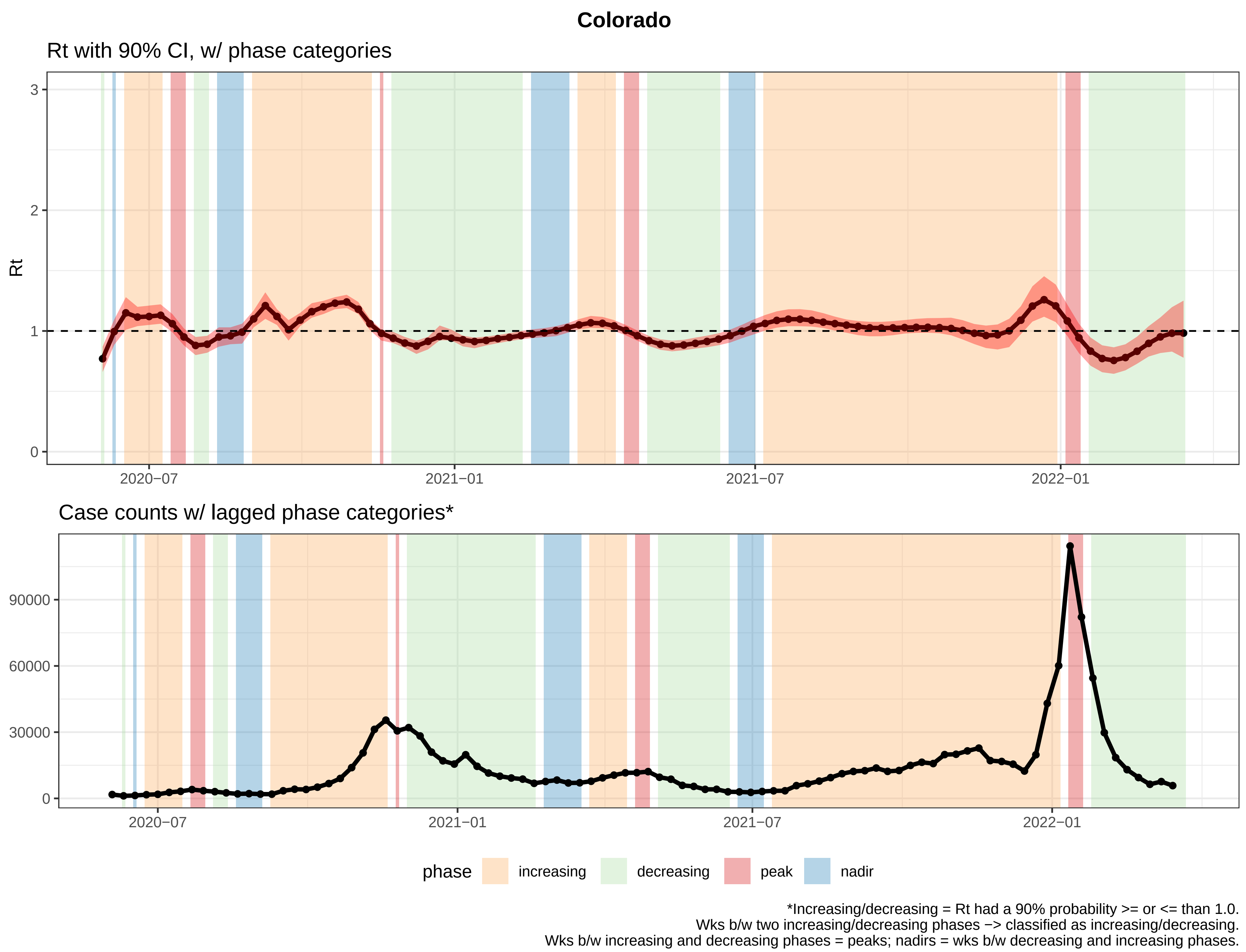
Colorado
Rt with 90% CI, w/ phase categories
Rt
3
2
1
0
2020−07
2021−01
2021−07
2022−01
Case counts w/ lagged phase categories*
90000
60000
30000
0
2020−07
2021−01
2021−07
2022−01
phase
increasing
decreasing
peak
nadir
*Increasing/decreasing = Rt had a 90% probability >= or <= than 1.0.
Wks b/w two increasing/decreasing phases −> classified as increasing/decreasing.
Wks b/w increasing and decreasing phases = peaks; nadirs = wks b/w decreasing and increasing phases.

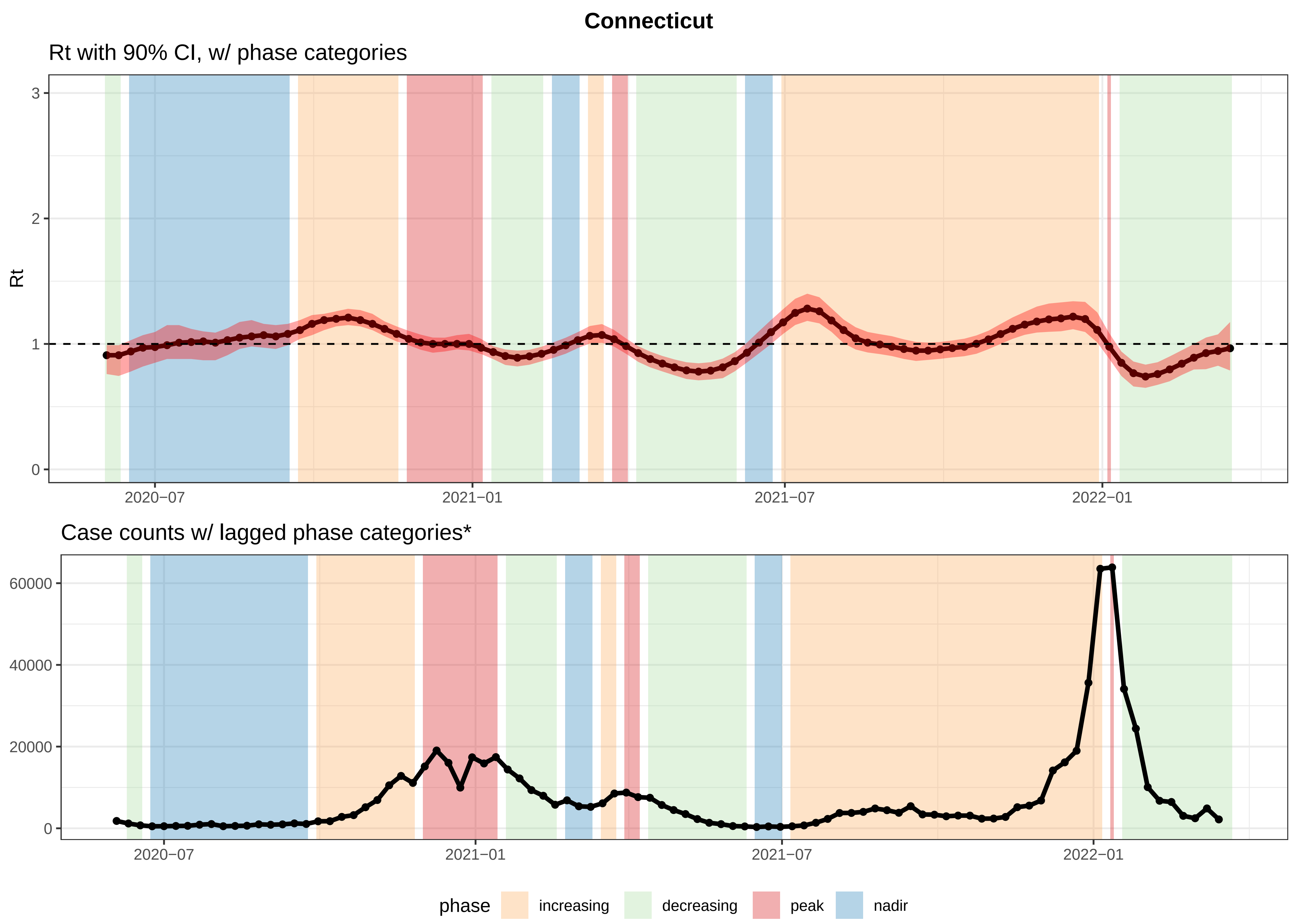

*Increasing/decreasing = Rt had a 90% probability >= or <= than 1.0.
Wks b/w two increasing/decreasing phases −> classified as increasing/decreasing.
Wks b/w increasing and decreasing phases = peaks; nadirs = wks b/w decreasing and increasing phases.

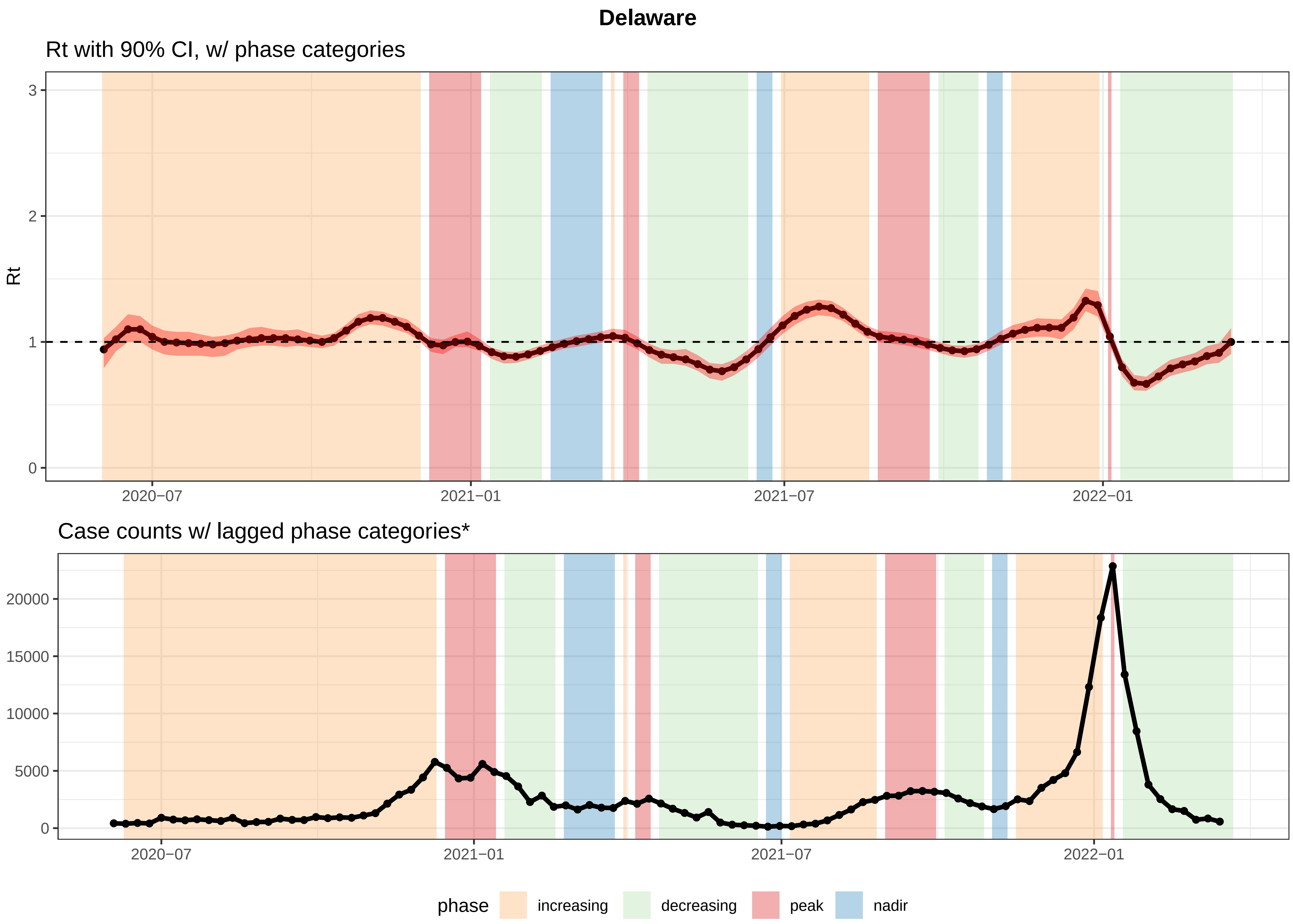

*Increasing/decreasing = Rt had a 90% probability >= or <= than 1.0.
Wks b/w two increasing/decreasing phases −> classified as increasing/decreasing.
Wks b/w increasing and decreasing phases = peaks; nadirs = wks b/w decreasing and increasing phases.

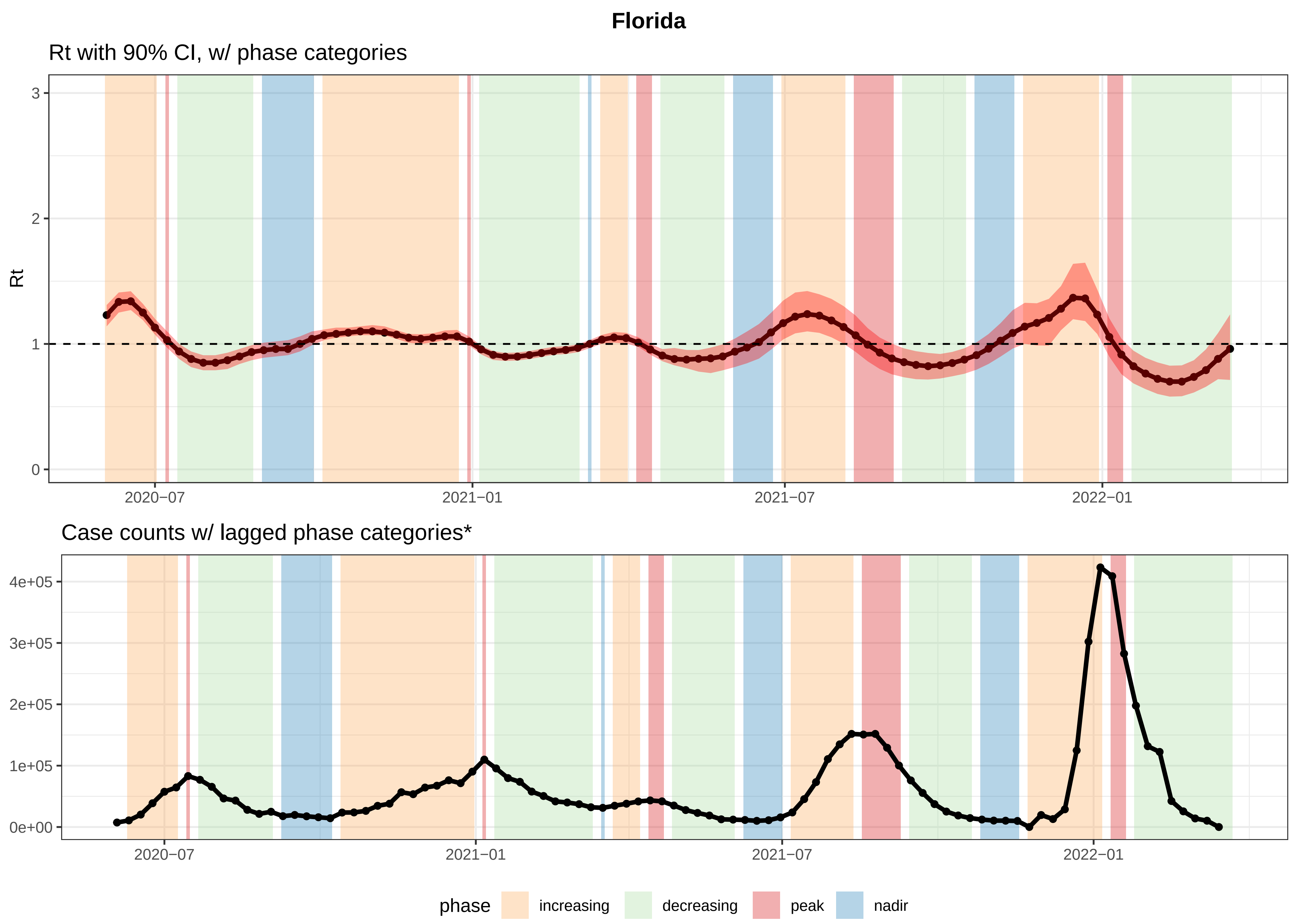

*Increasing/decreasing = Rt had a 90% probability >= or <= than 1.0.
Wks b/w two increasing/decreasing phases −> classified as increasing/decreasing.
Wks b/w increasing and decreasing phases = peaks; nadirs = wks b/w decreasing and increasing phases.

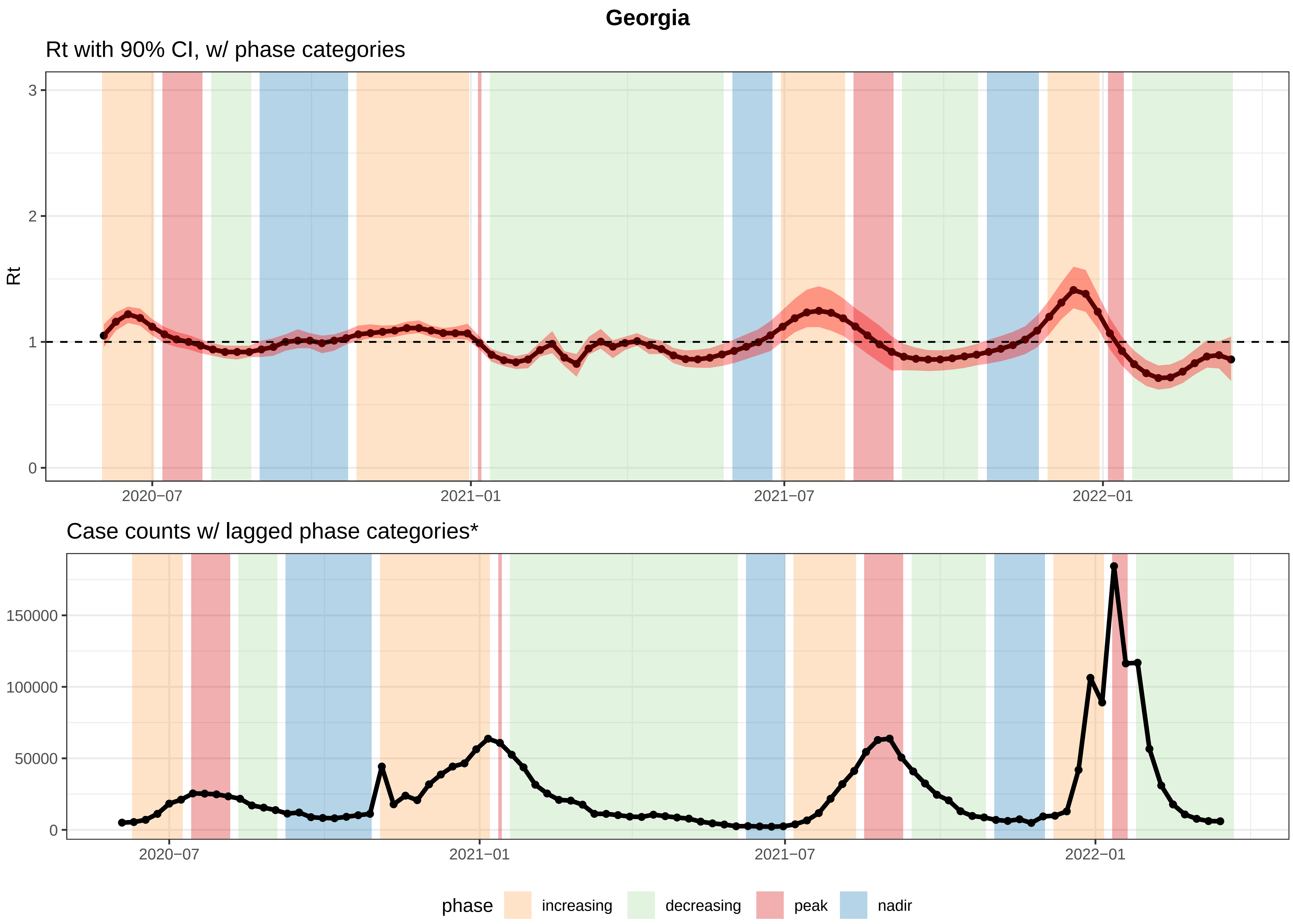

*Increasing/decreasing = Rt had a 90% probability >= or <= than 1.0.
Wks b/w two increasing/decreasing phases −> classified as increasing/decreasing.
Wks b/w increasing and decreasing phases = peaks; nadirs = wks b/w decreasing and increasing phases.

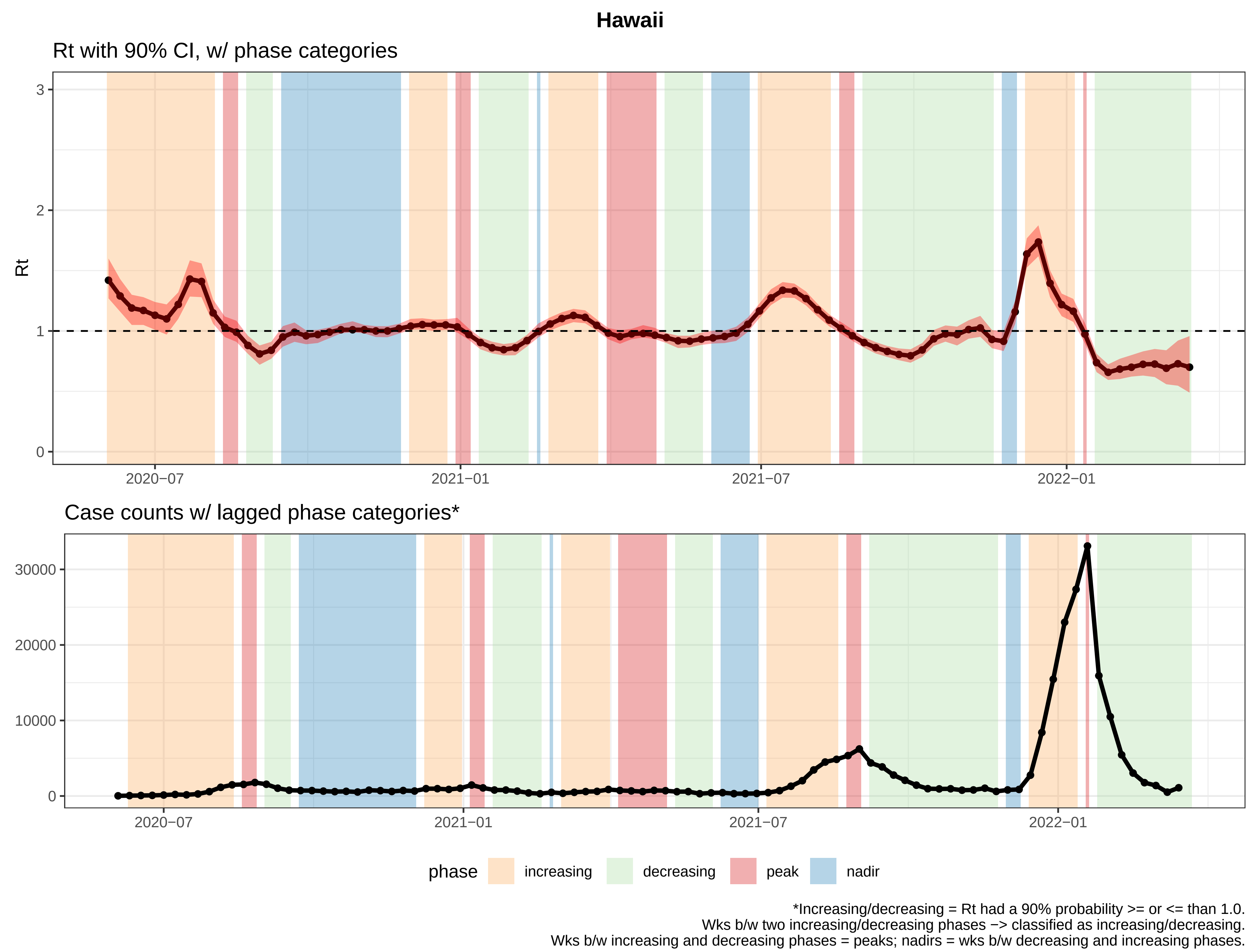
Hawaii
Rt with 90% CI, w/ phase categories
Rt
3
2
1
0
2020−07
2021−01
2021−07
2022−01
Case counts w/ lagged phase categories*
30000
20000
10000
0
2020−07
2021−01
2021−07
2022−01
phase
increasing
decreasing
peak
nadir
*Increasing/decreasing = Rt had a 90% probability >= or <= than 1.0.
Wks b/w two increasing/decreasing phases −> classified as increasing/decreasing.
Wks b/w increasing and decreasing phases = peaks; nadirs = wks b/w decreasing and increasing phases.

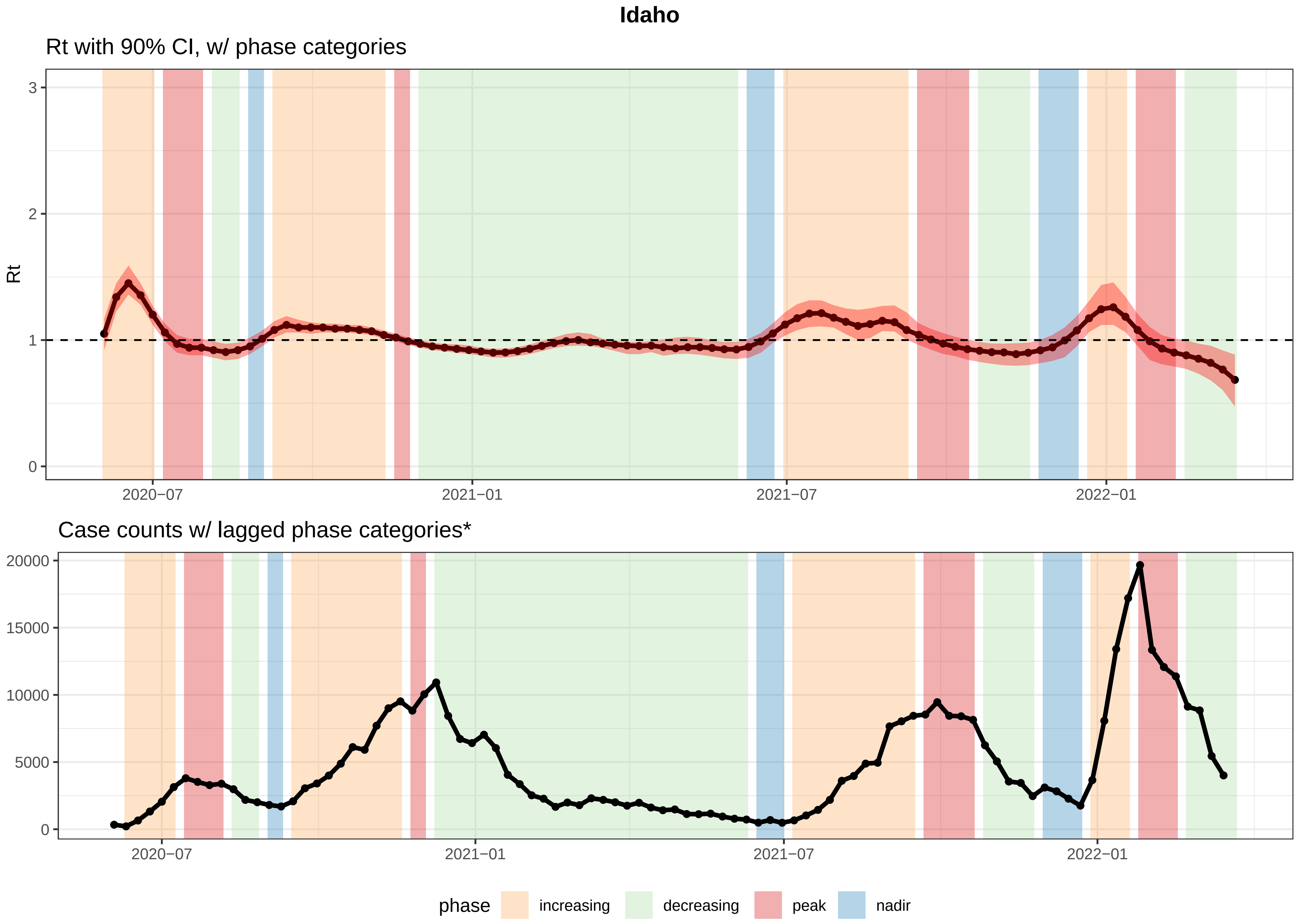

*Increasing/decreasing = Rt had a 90% probability >= or <= than 1.0.
Wks b/w two increasing/decreasing phases −> classified as increasing/decreasing.
Wks b/w increasing and decreasing phases = peaks; nadirs = wks b/w decreasing and increasing phases.

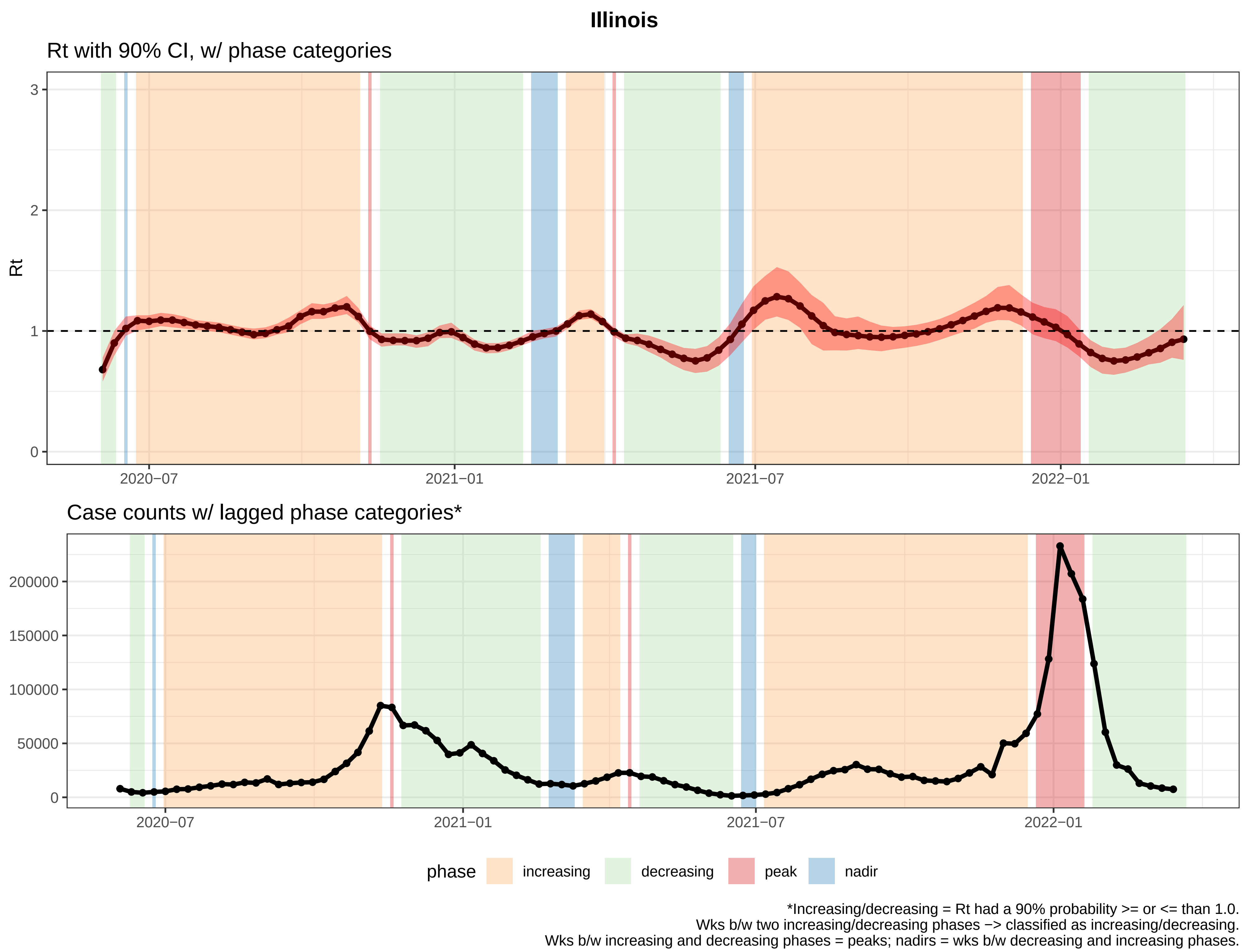
Illinois
Rt with 90% CI, w/ phase categories
Rt
3
2
1
0
2020−07
2021−01
2021−07
2022−01
Case counts w/ lagged phase categories*
200000
150000
100000
50000
0
2020−07
2021−01
2021−07
2022−01
phase
increasing
decreasing
peak
nadir
*Increasing/decreasing = Rt had a 90% probability >= or <= than 1.0.
Wks b/w two increasing/decreasing phases −> classified as increasing/decreasing.
Wks b/w increasing and decreasing phases = peaks; nadirs = wks b/w decreasing and increasing phases.

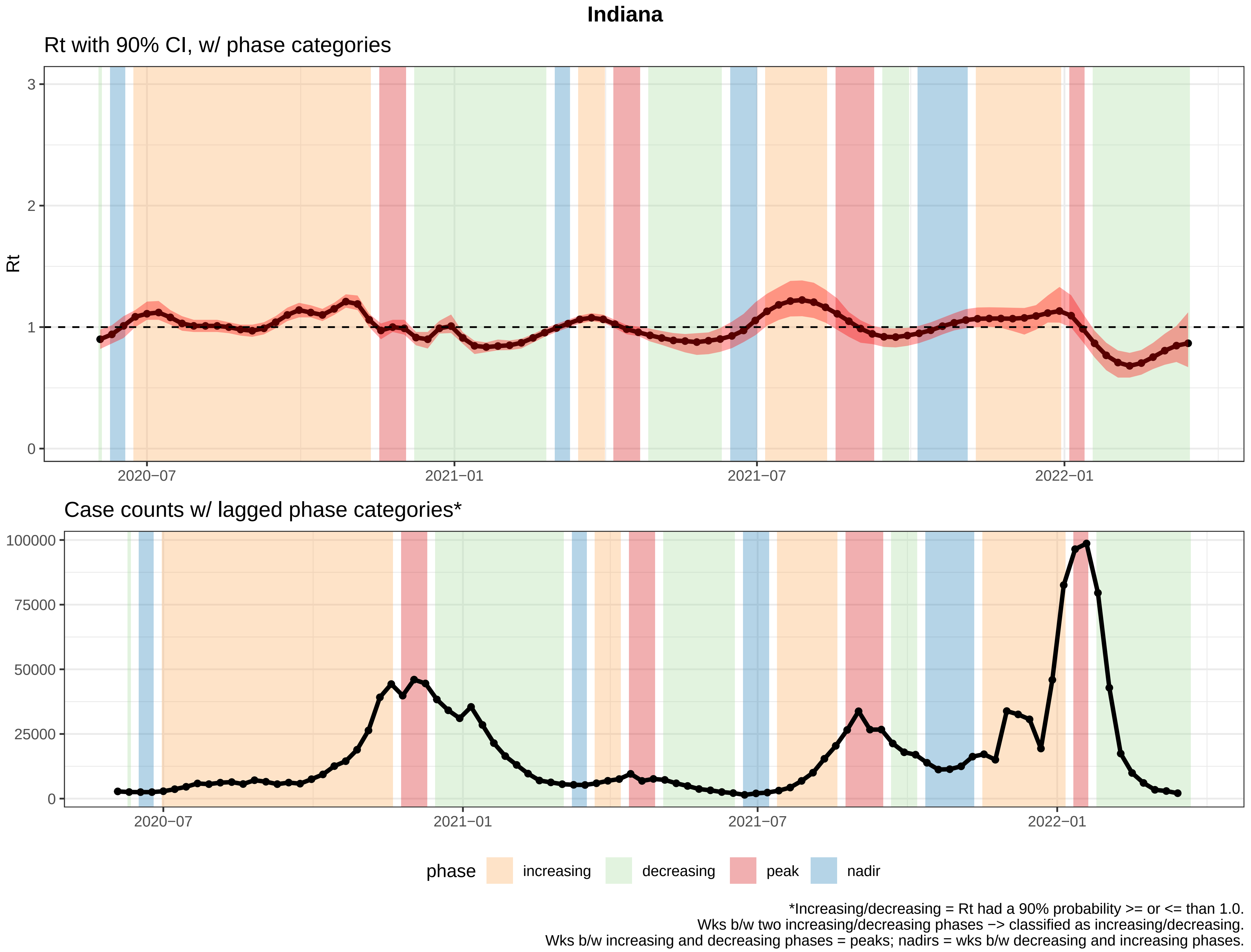
Indiana
Rt with 90% CI, w/ phase categories
Rt
0
1
2
3
2020−07
2021−01
2021−07
2022−01
Case counts w/ lagged phase categories*
0
25000
50000
75000
100000
2020−07
2021−01
2021−07
2022−01
phase
increasing
decreasing
peak
nadir
*Increasing/decreasing = Rt had a 90% probability >= or <= than 1.0.
Wks b/w two increasing/decreasing phases −> classified as increasing/decreasing.
Wks b/w increasing and decreasing phases = peaks; nadirs = wks b/w decreasing and increasing phases.

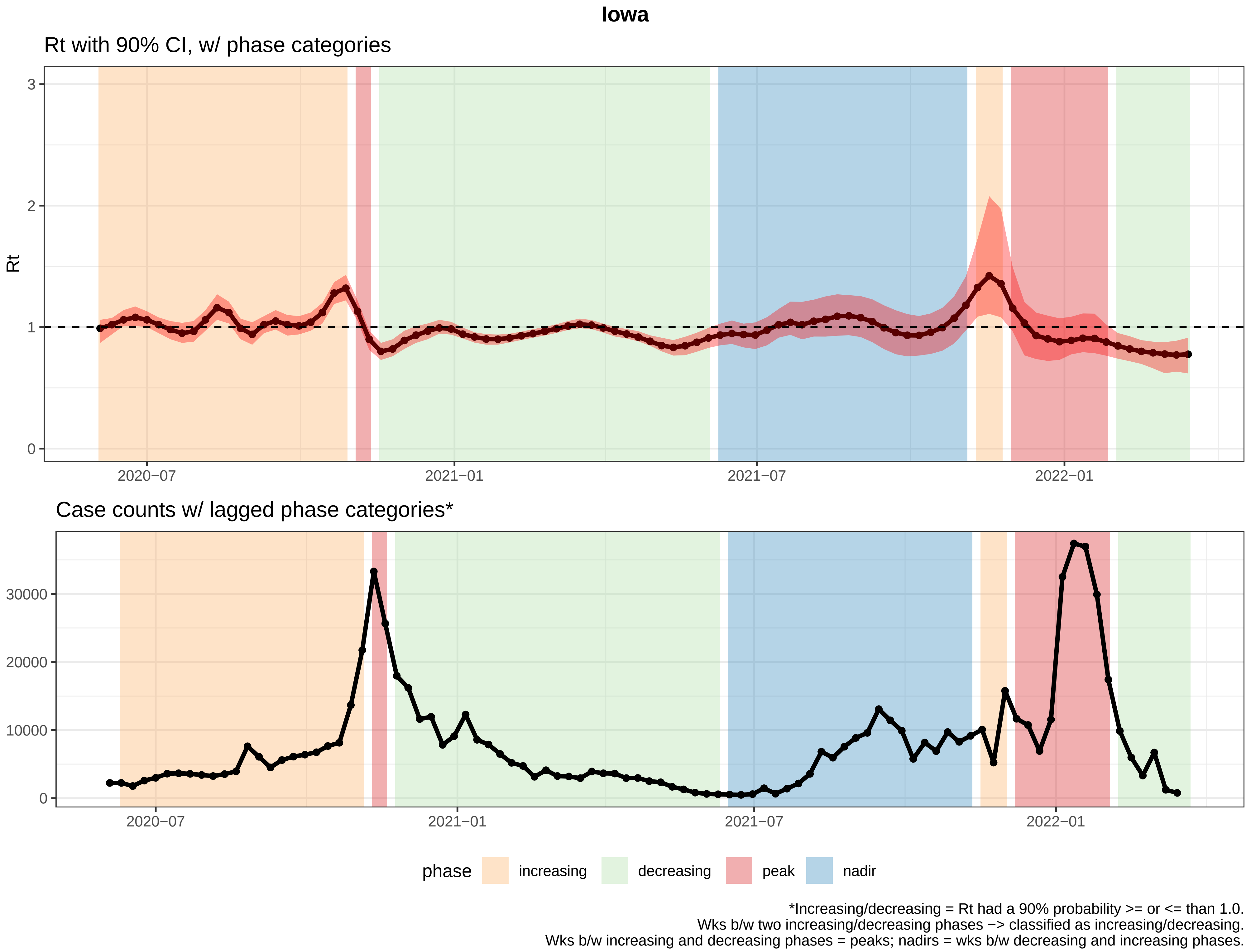
Iowa
Rt with 90% CI, w/ phase categories
Rt
3
2
1
0
2020−07
2021−01
2021−07
2022−01
Case counts w/ lagged phase categories*
30000
20000
10000
0
2020−07
2021−01
2021−07
2022−01
phase
increasing
decreasing
peak
nadir
*Increasing/decreasing = Rt had a 90% probability >= or <= than 1.0.
Wks b/w two increasing/decreasing phases −> classified as increasing/decreasing.
Wks b/w increasing and decreasing phases = peaks; nadirs = wks b/w decreasing and increasing phases.

Kansas

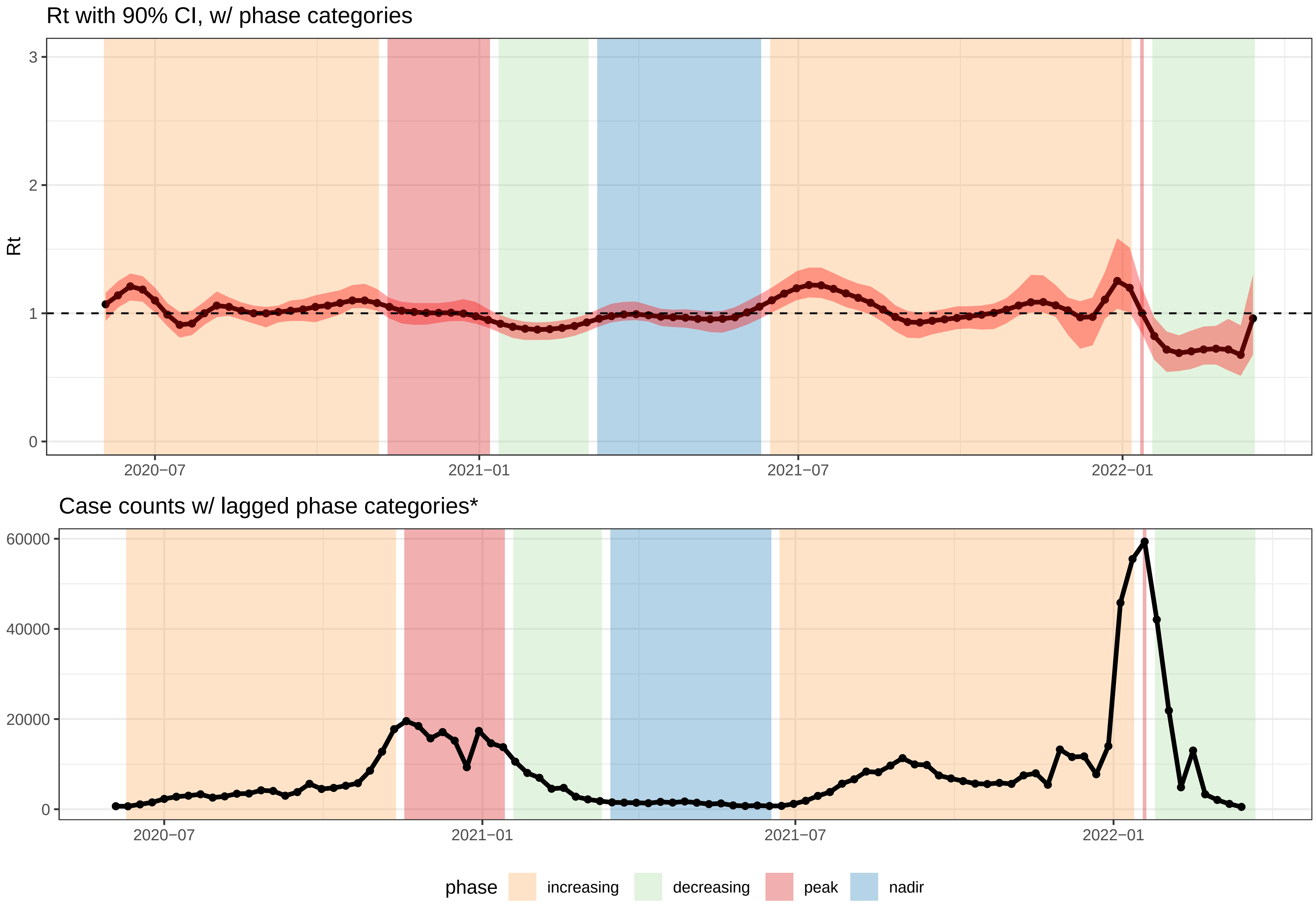

*Increasing/decreasing = Rt had a 90% probability >= or <= than 1.0.
Wks b/w two increasing/decreasing phases −> classified as increasing/decreasing.
Wks b/w increasing and decreasing phases = peaks; nadirs = wks b/w decreasing and increasing phases.

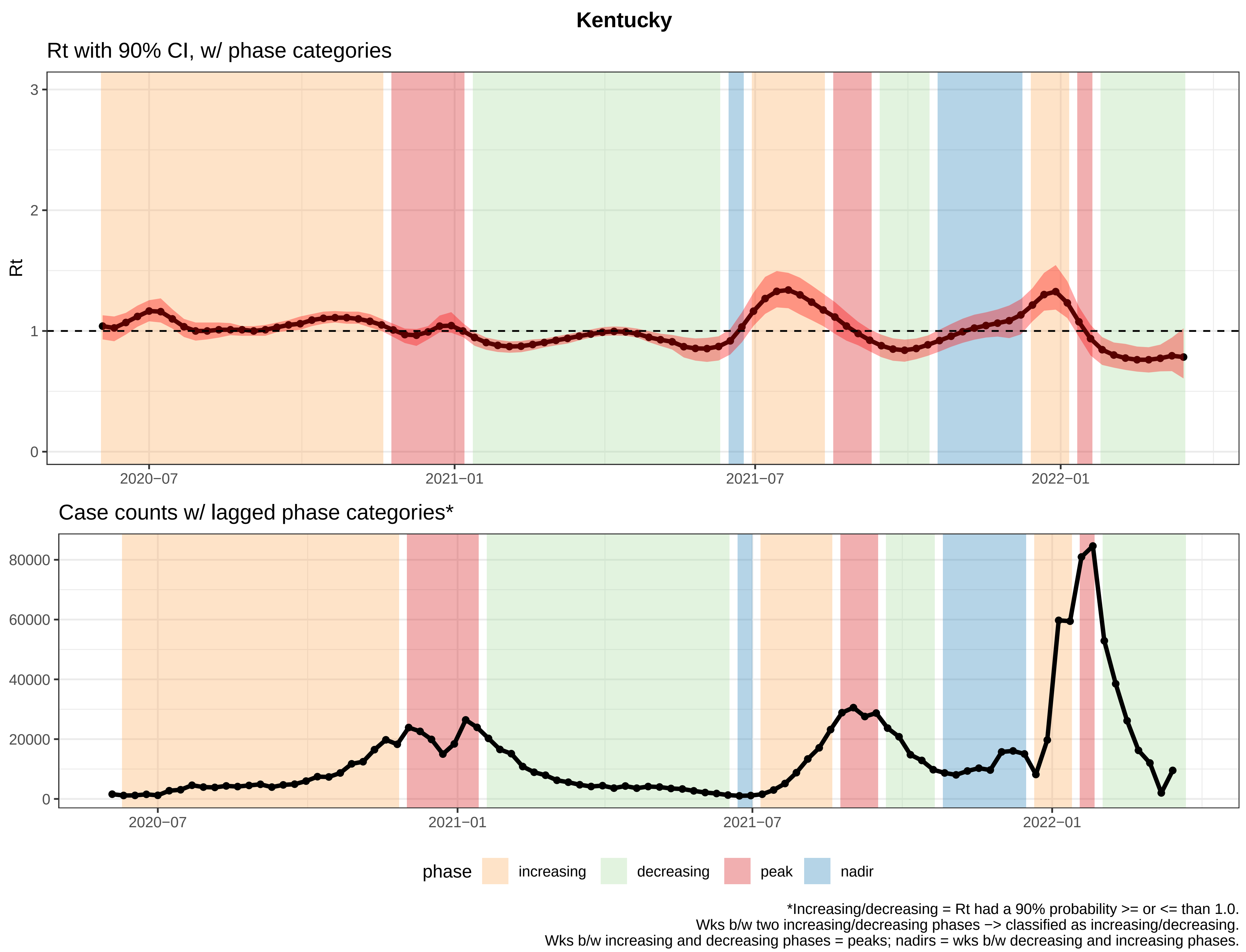
Kentucky
Rt with 90% CI, w/ phase categories
Rt
3
2
1
0
2020−07
2021−01
2021−07
2022−01
Case counts w/ lagged phase categories*
80000
60000
40000
20000
0
2020−07
2021−01
2021−07
2022−01
phase
increasing
decreasing
peak
nadir
*Increasing/decreasing = Rt had a 90% probability >= or <= than 1.0.
Wks b/w two increasing/decreasing phases −> classified as increasing/decreasing.
Wks b/w increasing and decreasing phases = peaks; nadirs = wks b/w decreasing and increasing phases.

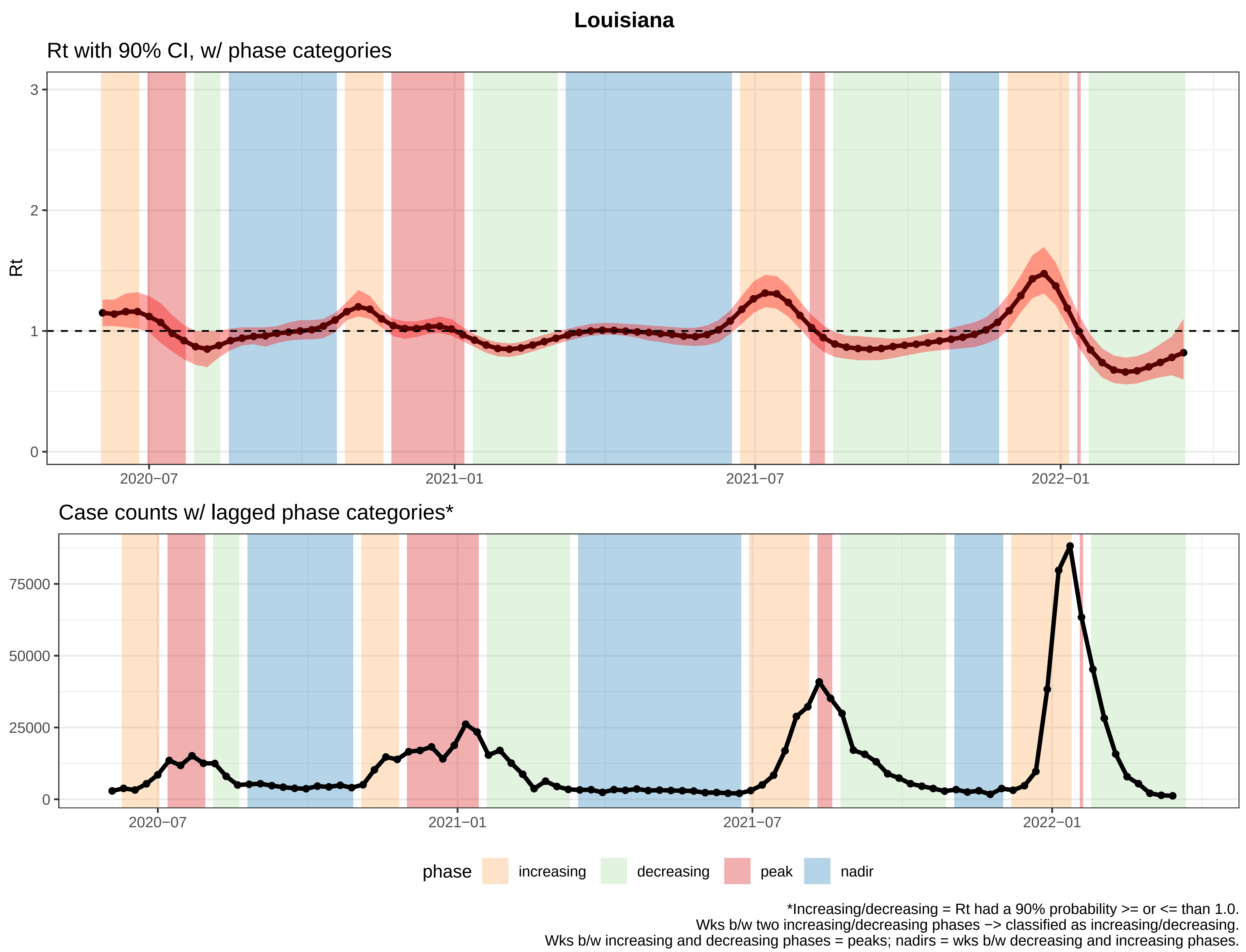
Louisiana
Rt with 90% CI, w/ phase categories
Rt
3
2
1
0
2020−07
2021−01
2021−07
2022−01
Case counts w/ lagged phase categories*
75000
50000
25000
0
2020−07
2021−01
2021−07
2022−01
phase
increasing
decreasing
peak
nadir
*Increasing/decreasing = Rt had a 90% probability >= or <= than 1.0.
Wks b/w two increasing/decreasing phases −> classified as increasing/decreasing.
Wks b/w increasing and decreasing phases = peaks; nadirs = wks b/w decreasing and increasing phases.

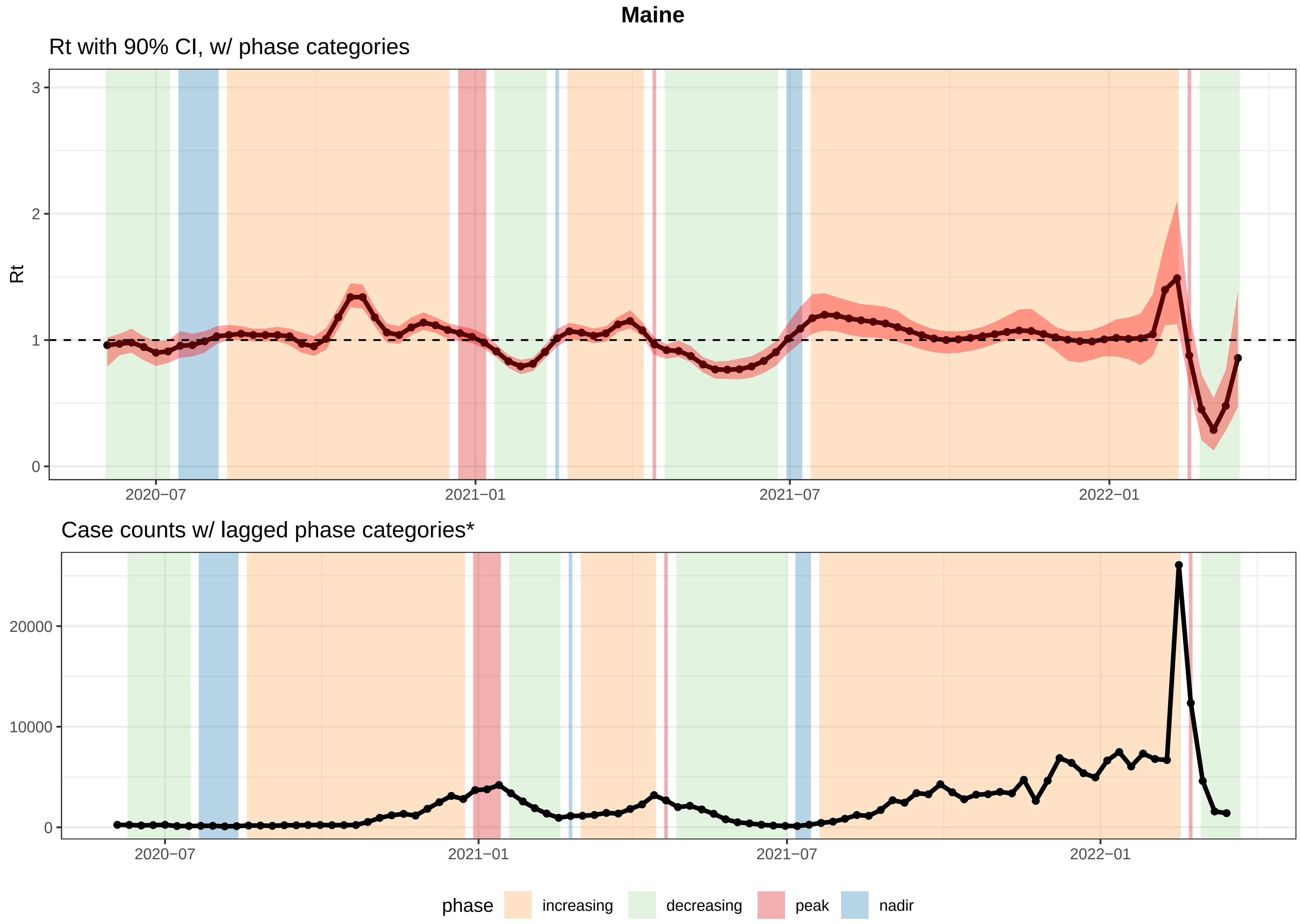

*Increasing/decreasing = Rt had a 90% probability >= or <= than 1.0.
Wks b/w two increasing/decreasing phases −> classified as increasing/decreasing.
Wks b/w increasing and decreasing phases = peaks; nadirs = wks b/w decreasing and increasing phases.

Maryland

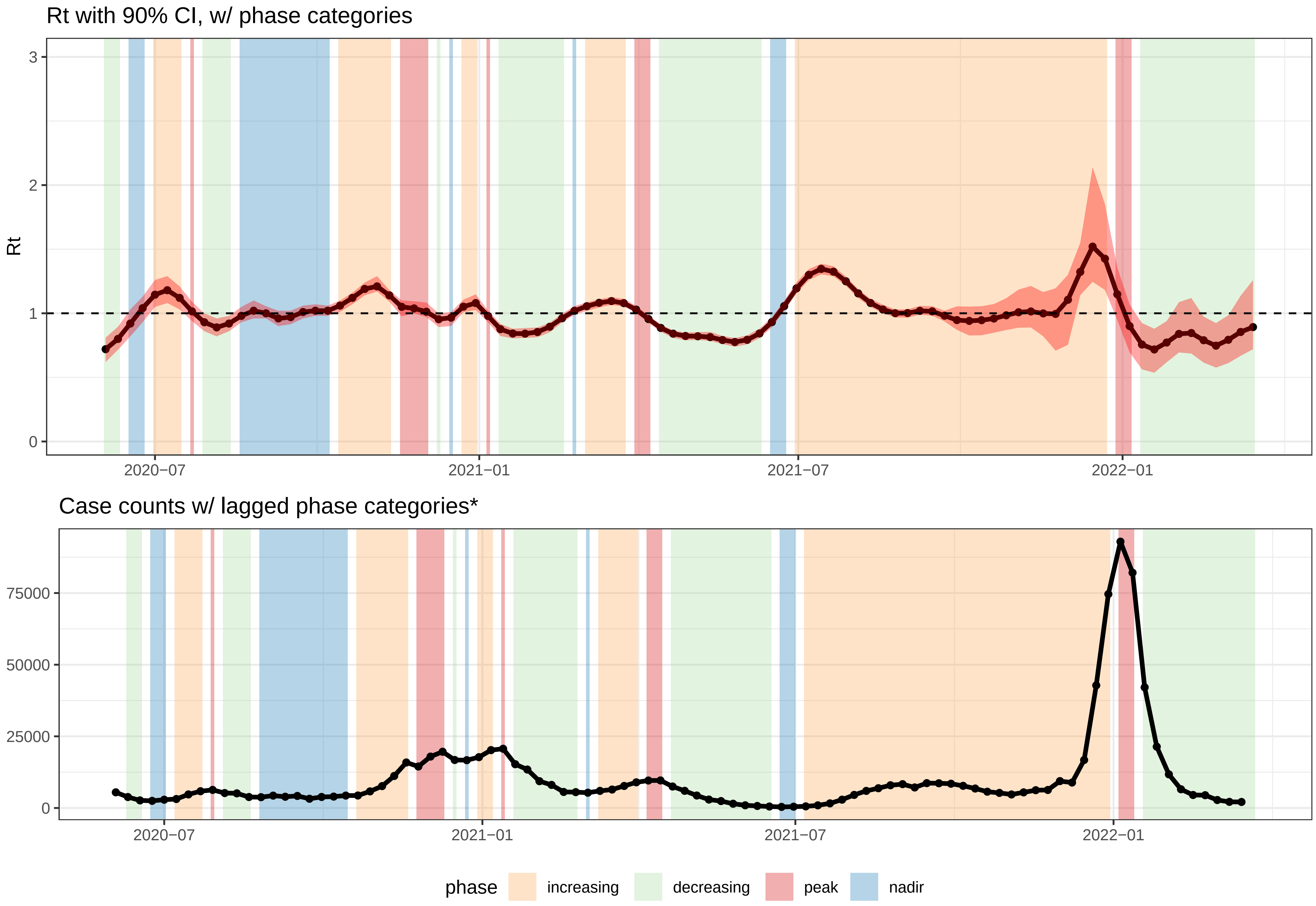

*Increasing/decreasing = Rt had a 90% probability >= or <= than 1.0.
Wks b/w two increasing/decreasing phases −> classified as increasing/decreasing.
Wks b/w increasing and decreasing phases = peaks; nadirs = wks b/w decreasing and increasing phases.

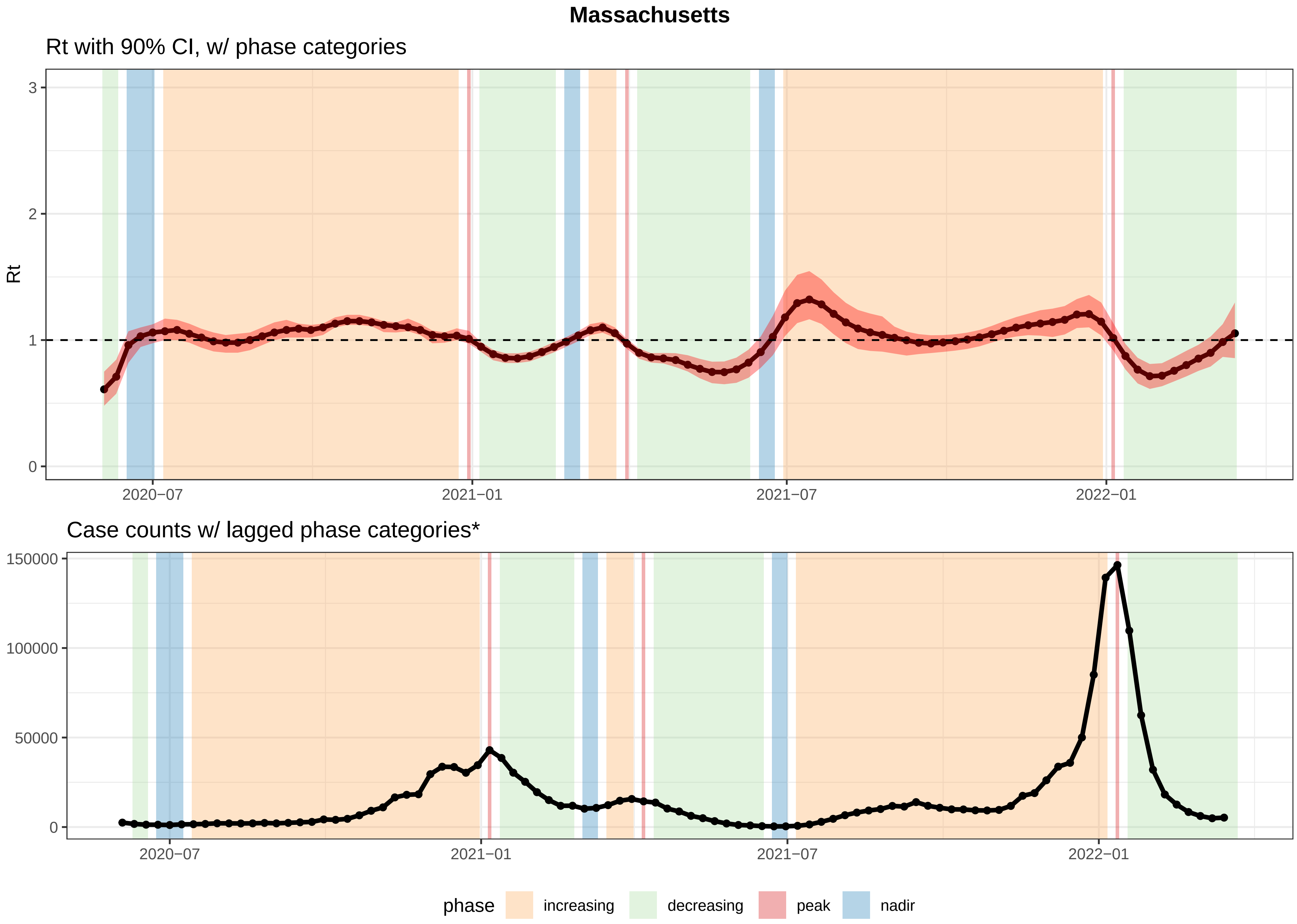

*Increasing/decreasing = Rt had a 90% probability >= or <= than 1.0.
Wks b/w two increasing/decreasing phases −> classified as increasing/decreasing.
Wks b/w increasing and decreasing phases = peaks; nadirs = wks b/w decreasing and increasing phases.

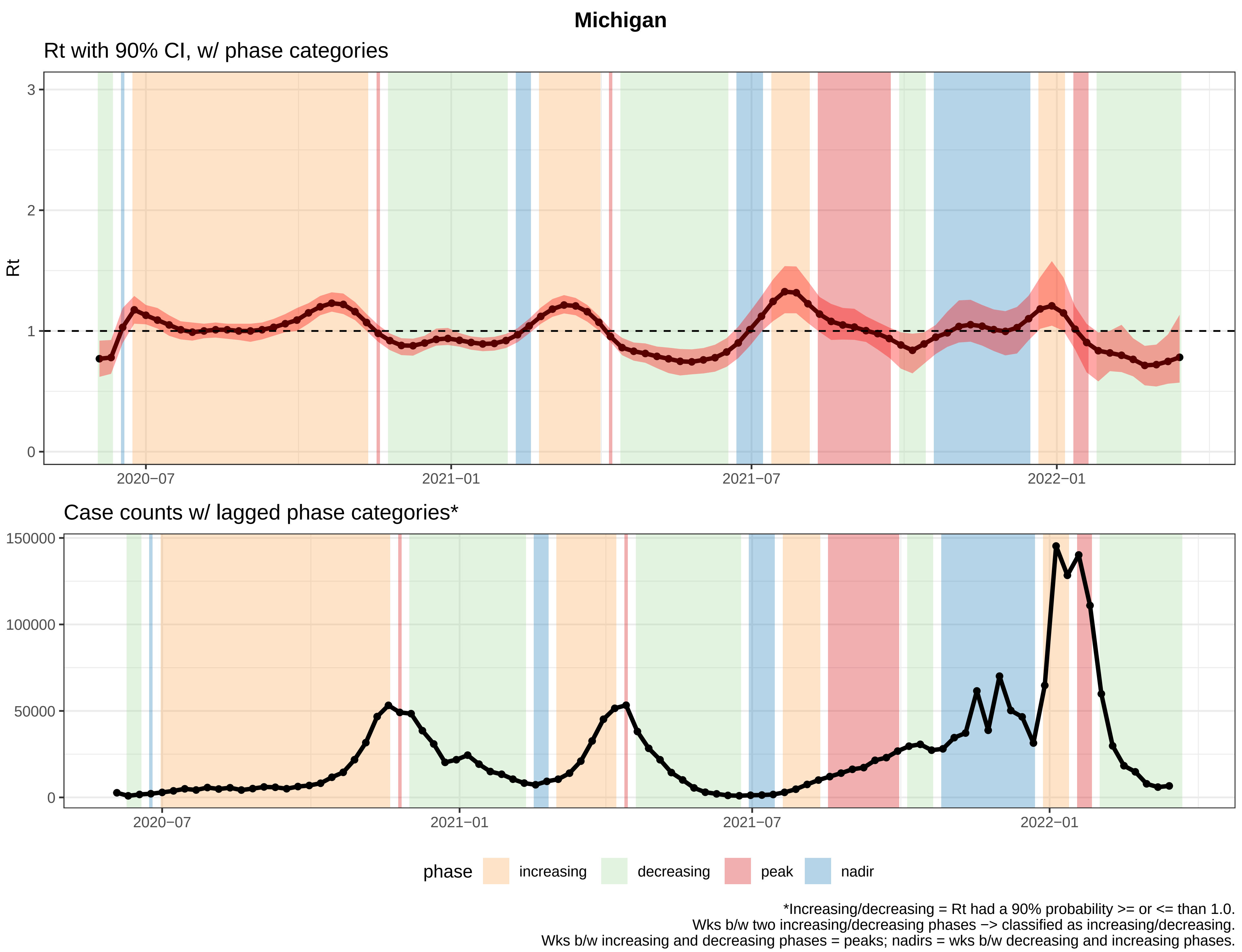
Michigan
Rt with 90% CI, w/ phase categories
Rt
3
2
1
0
2020−07
2021−01
2021−07
2022−01
Case counts w/ lagged phase categories*
150000
100000
50000
0
2020−07
2021−01
2021−07
2022−01
phase
increasing
decreasing
peak
nadir
*Increasing/decreasing = Rt had a 90% probability >= or <= than 1.0.
Wks b/w two increasing/decreasing phases −> classified as increasing/decreasing.
Wks b/w increasing and decreasing phases = peaks; nadirs = wks b/w decreasing and increasing phases.

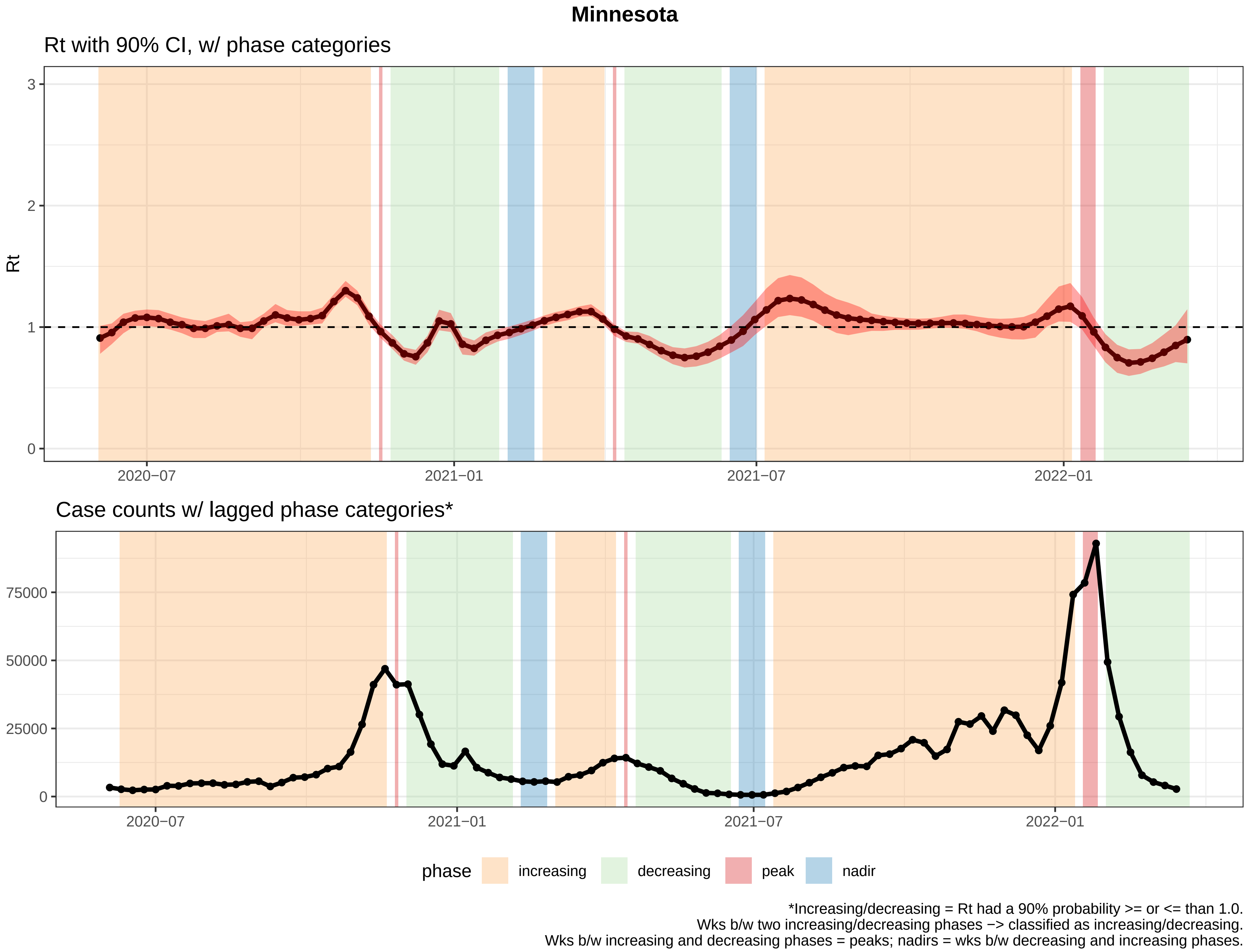
Minnesota
Rt with 90% CI, w/ phase categories
Rt
3
2
1
0
2020−07
2021−01
2021−07
2022−01
Case counts w/ lagged phase categories*
75000
50000
25000
0
2020−07
2021−01
2021−07
2022−01
phase
increasing
decreasing
peak
nadir
*Increasing/decreasing = Rt had a 90% probability >= or <= than 1.0.
Wks b/w two increasing/decreasing phases −> classified as increasing/decreasing.
Wks b/w increasing and decreasing phases = peaks; nadirs = wks b/w decreasing and increasing phases.

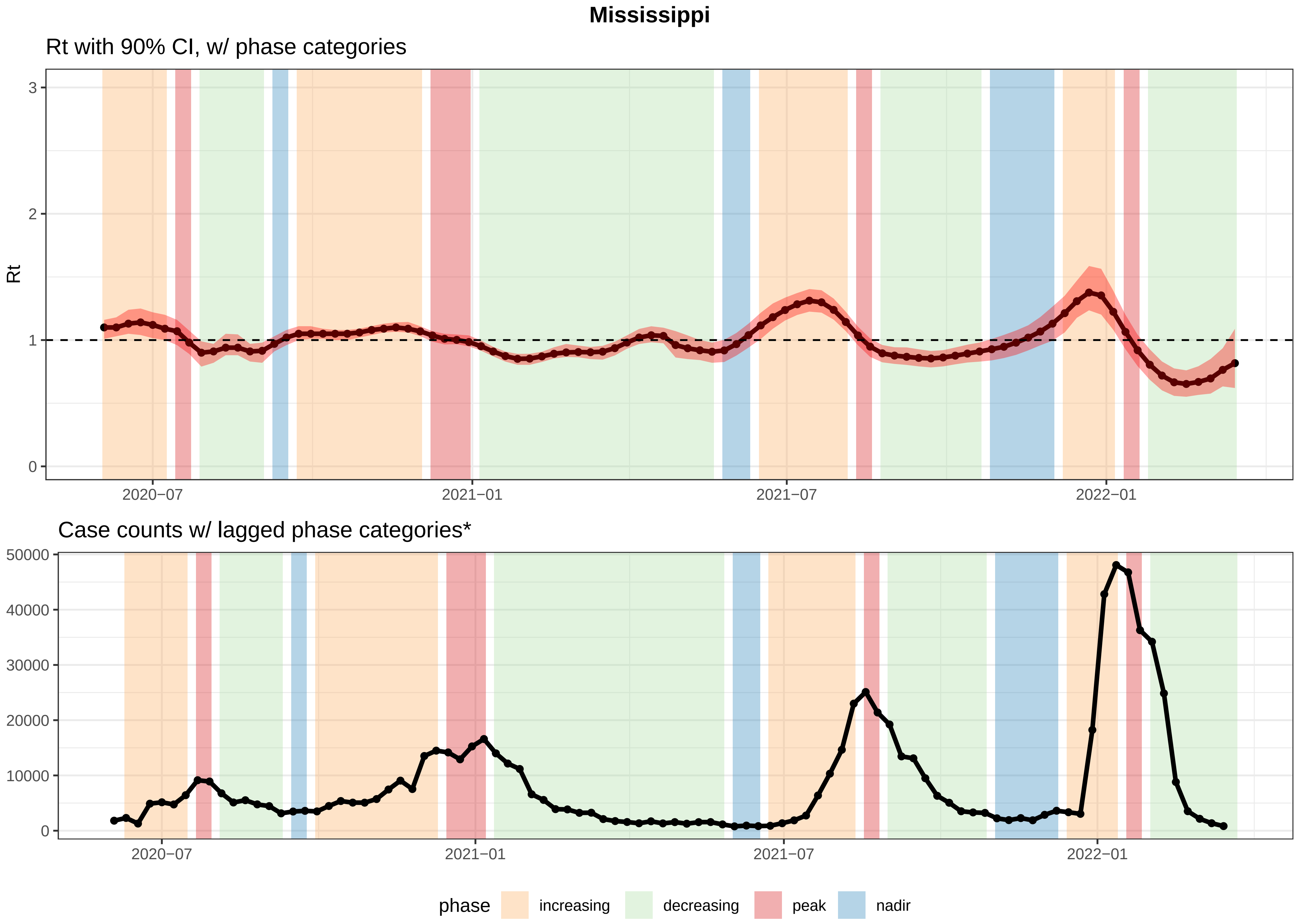

*Increasing/decreasing = Rt had a 90% probability >= or <= than 1.0.
Wks b/w two increasing/decreasing phases −> classified as increasing/decreasing.
Wks b/w increasing and decreasing phases = peaks; nadirs = wks b/w decreasing and increasing phases.

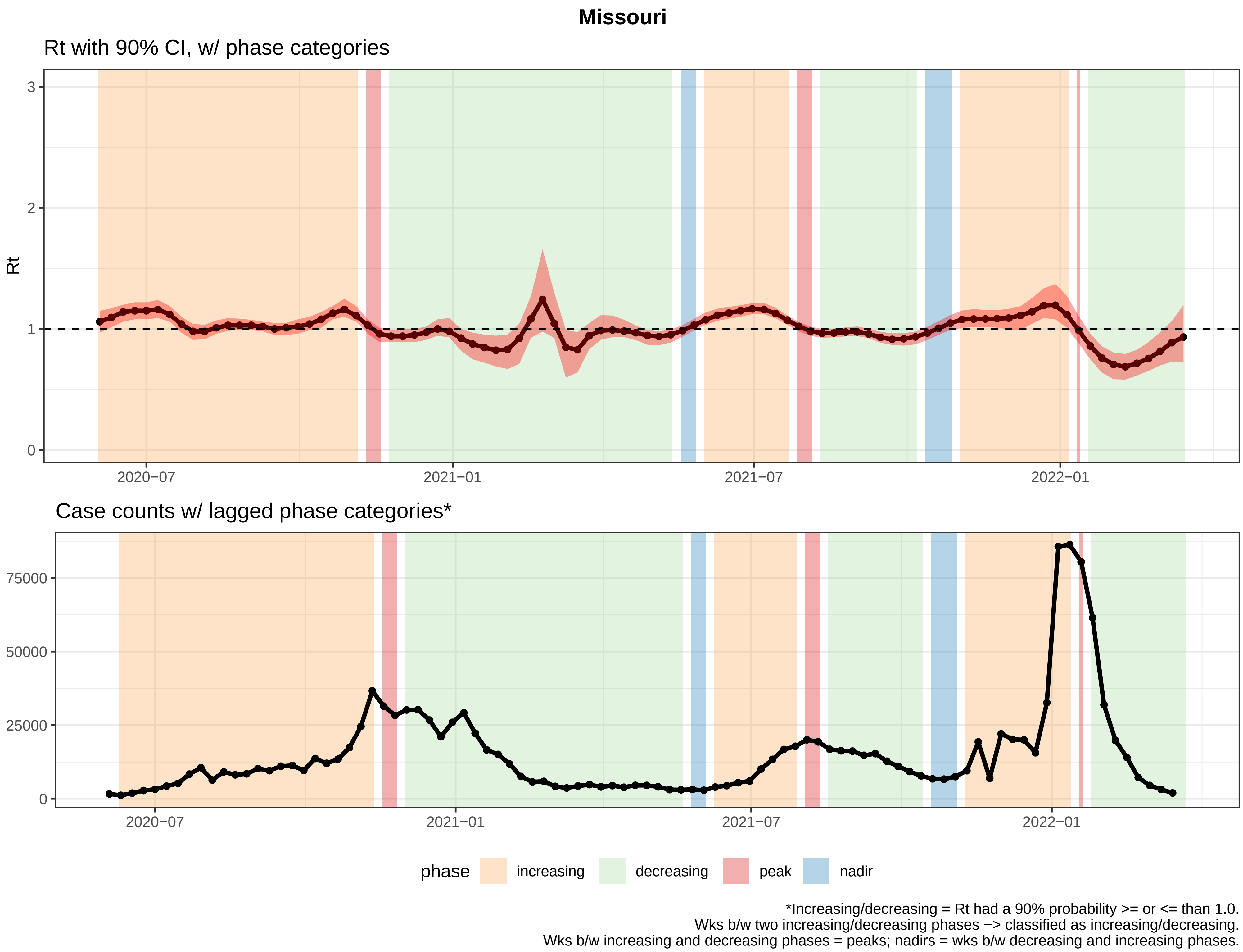
Missouri
Rt with 90% CI, w/ phase categories
Rt
3
2
1
0
2020−07
2021−01
2021−07
2022−01
Case counts w/ lagged phase categories*
75000
50000
25000
0
2020−07
2021−01
2021−07
2022−01
phase
increasing
decreasing
peak
nadir
*Increasing/decreasing = Rt had a 90% probability >= or <= than 1.0.
Wks b/w two increasing/decreasing phases −> classified as increasing/decreasing.
Wks b/w increasing and decreasing phases = peaks; nadirs = wks b/w decreasing and increasing phases.

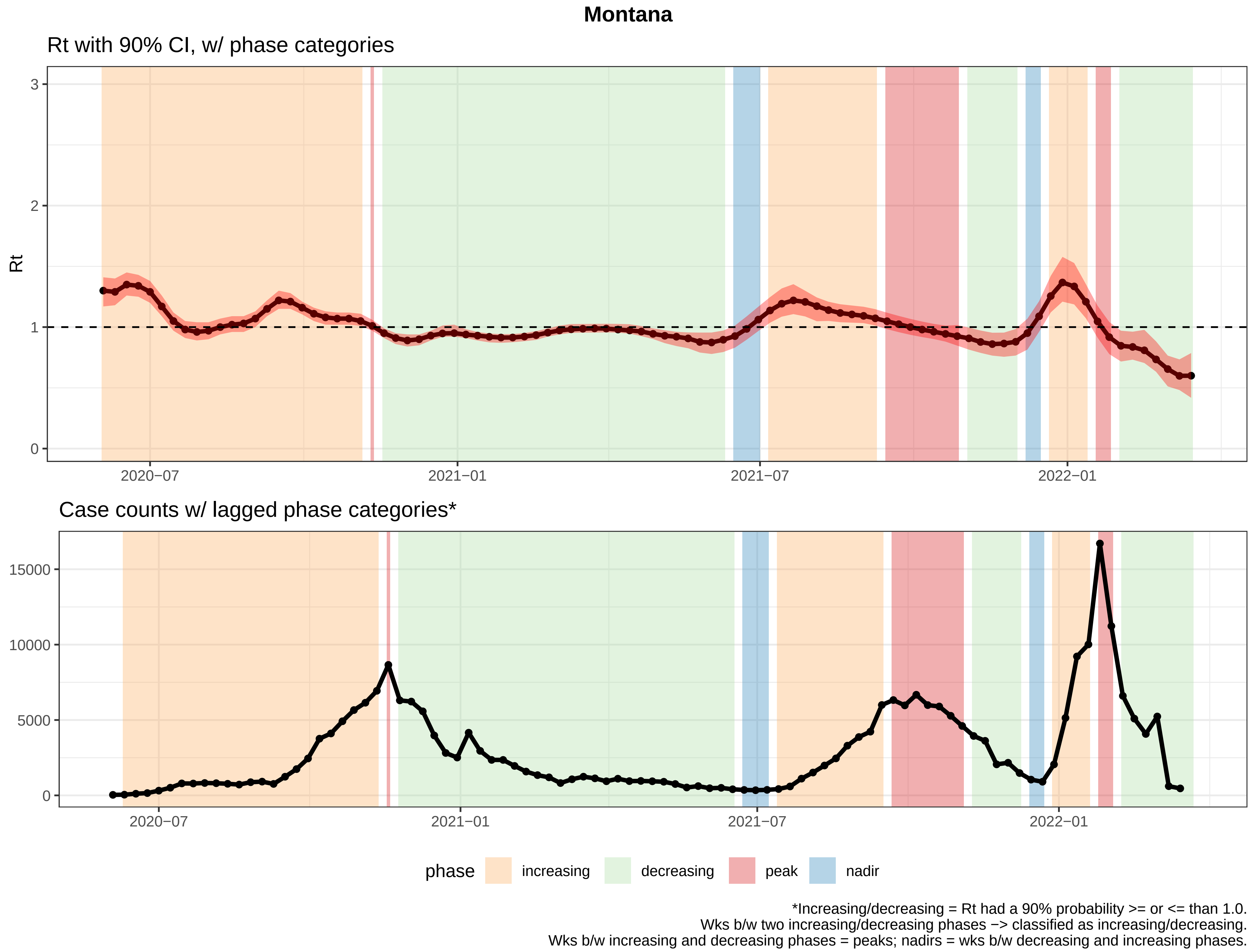
Montana
Rt with 90% CI, w/ phase categories
Rt
3
2
1
0
2020−07
2021−01
2021−07
2022−01
Case counts w/ lagged phase categories*
15000
10000
5000
0
2020−07
2021−01
2021−07
2022−01
phase
increasing
decreasing
peak
nadir
*Increasing/decreasing = Rt had a 90% probability >= or <= than 1.0.
Wks b/w two increasing/decreasing phases −> classified as increasing/decreasing.
Wks b/w increasing and decreasing phases = peaks; nadirs = wks b/w decreasing and increasing phases.

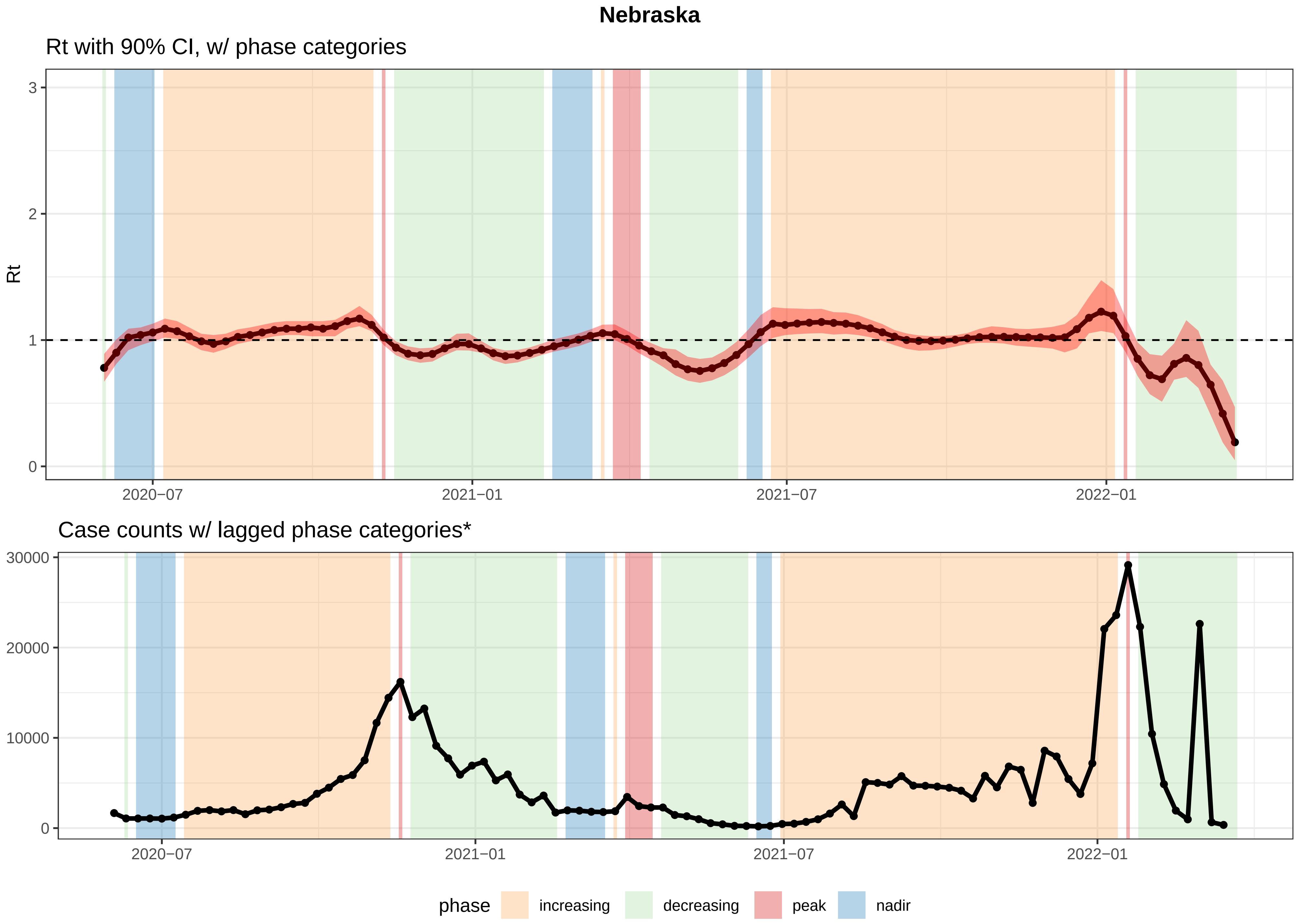

*Increasing/decreasing = Rt had a 90% probability >= or <= than 1.0.
Wks b/w two increasing/decreasing phases −> classified as increasing/decreasing.
Wks b/w increasing and decreasing phases = peaks; nadirs = wks b/w decreasing and increasing phases.

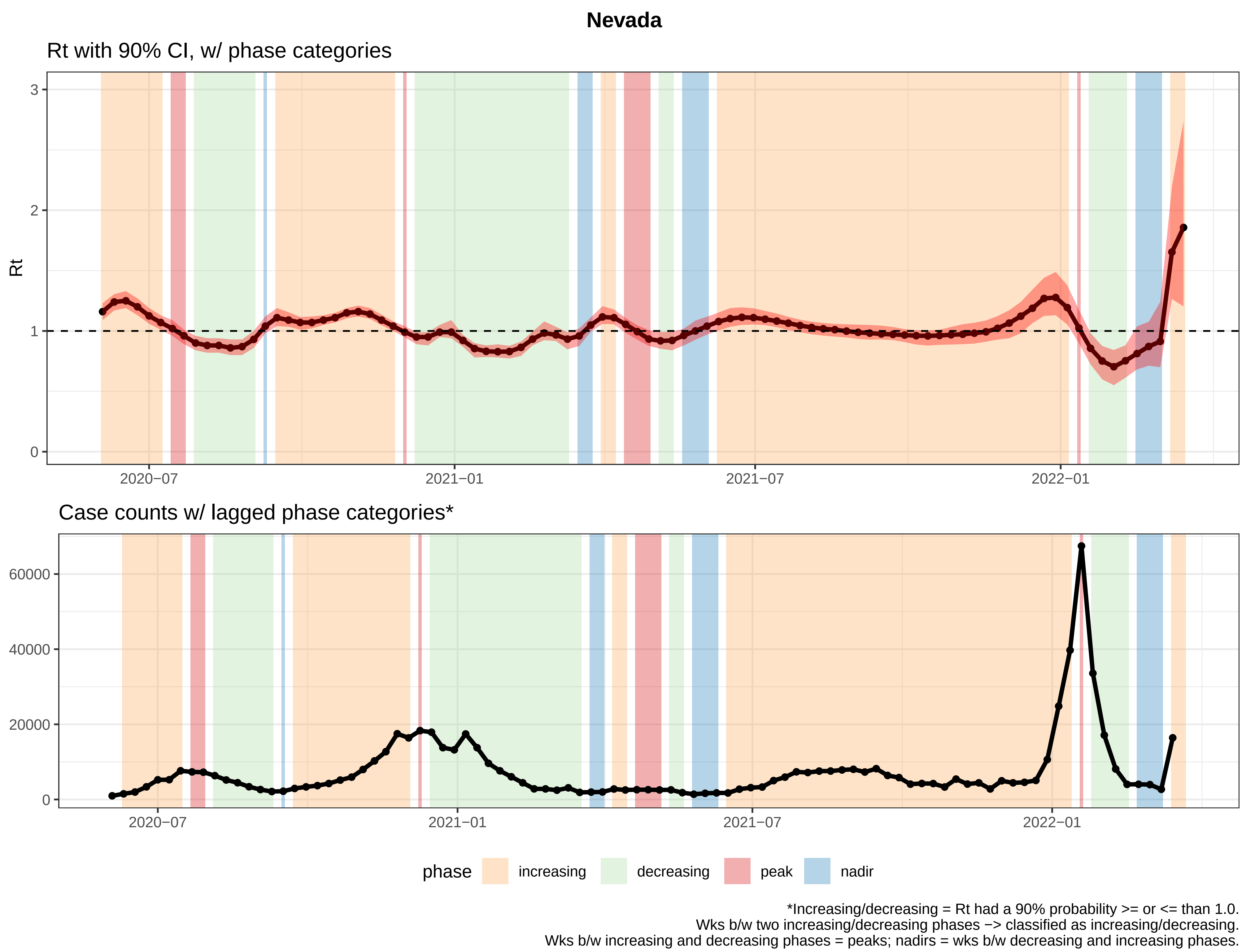
Nevada
Rt with 90% CI, w/ phase categories
Rt
0
1
2
3
2020−07
2021−01
2021−07
2022−01
Case counts w/ lagged phase categories*
0
20000
40000
60000
2020−07
2021−01
2021−07
2022−01
phase
increasing
decreasing
peak
nadir
*Increasing/decreasing = Rt had a 90% probability >= or <= than 1.0.
Wks b/w two increasing/decreasing phases −> classified as increasing/decreasing.
Wks b/w increasing and decreasing phases = peaks; nadirs = wks b/w decreasing and increasing phases.

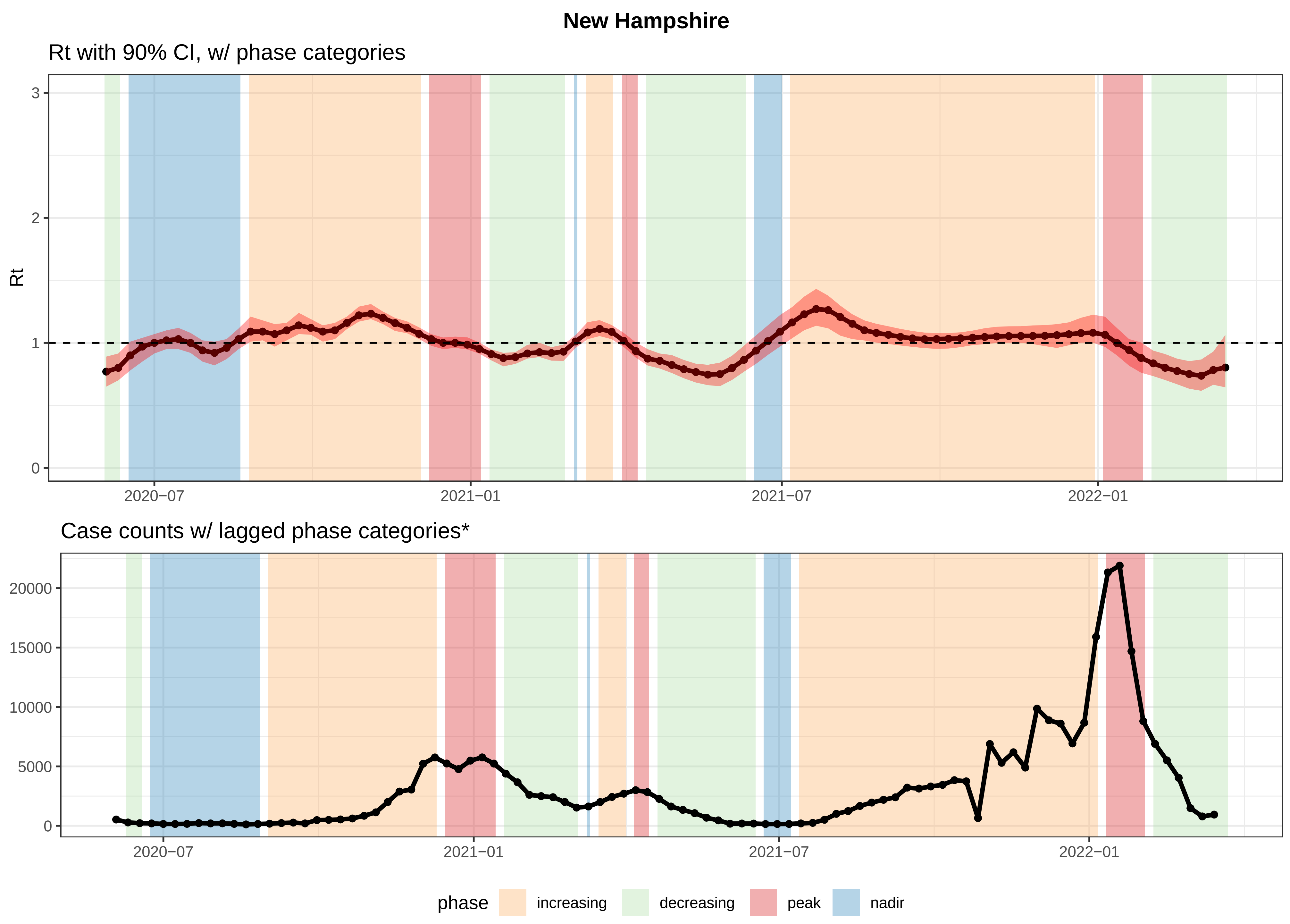

*Increasing/decreasing = Rt had a 90% probability >= or <= than 1.0.
Wks b/w two increasing/decreasing phases −> classified as increasing/decreasing.
Wks b/w increasing and decreasing phases = peaks; nadirs = wks b/w decreasing and increasing phases.

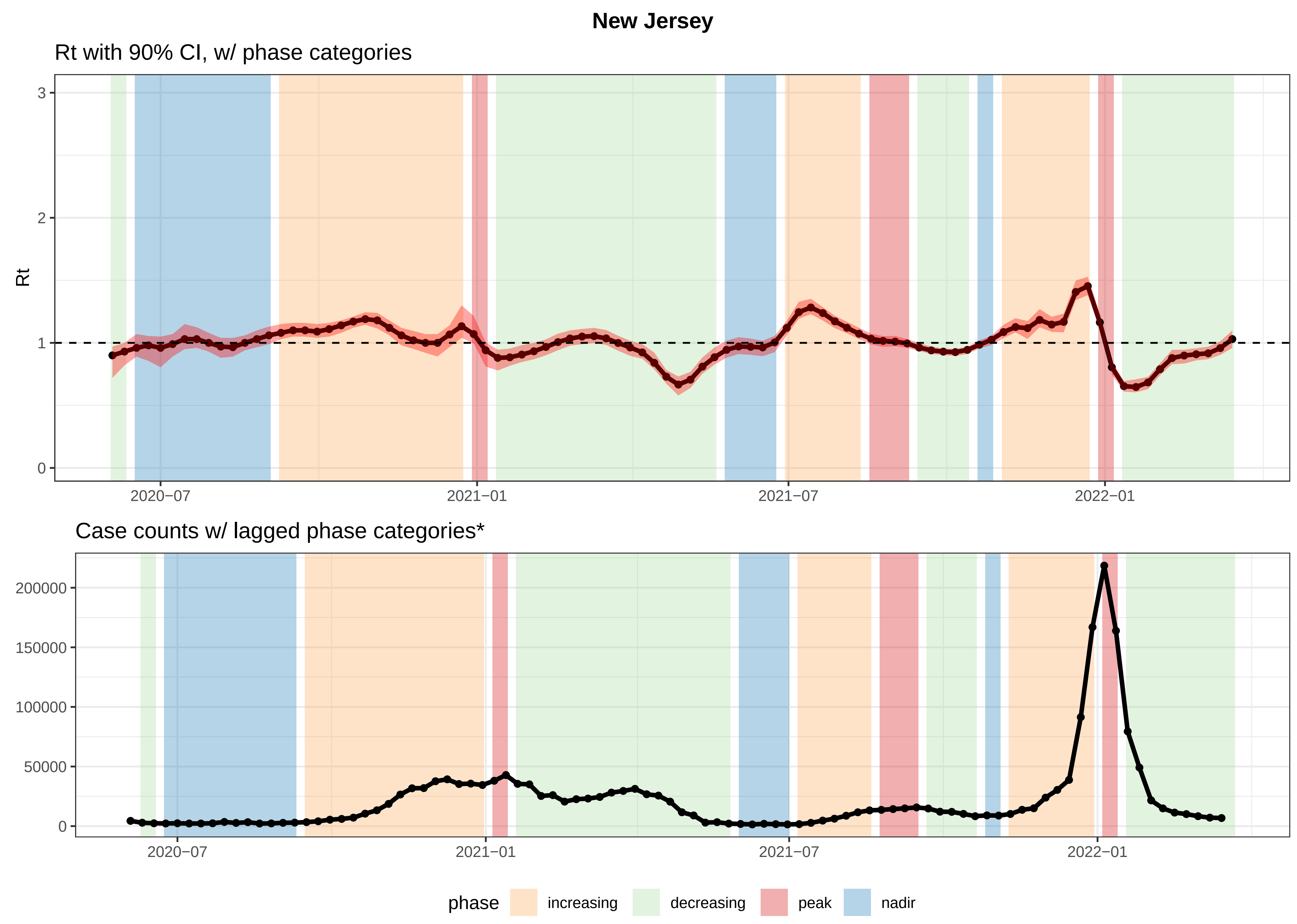

*Increasing/decreasing = Rt had a 90% probability >= or <= than 1.0.
Wks b/w two increasing/decreasing phases −> classified as increasing/decreasing.
Wks b/w increasing and decreasing phases = peaks; nadirs = wks b/w decreasing and increasing phases.

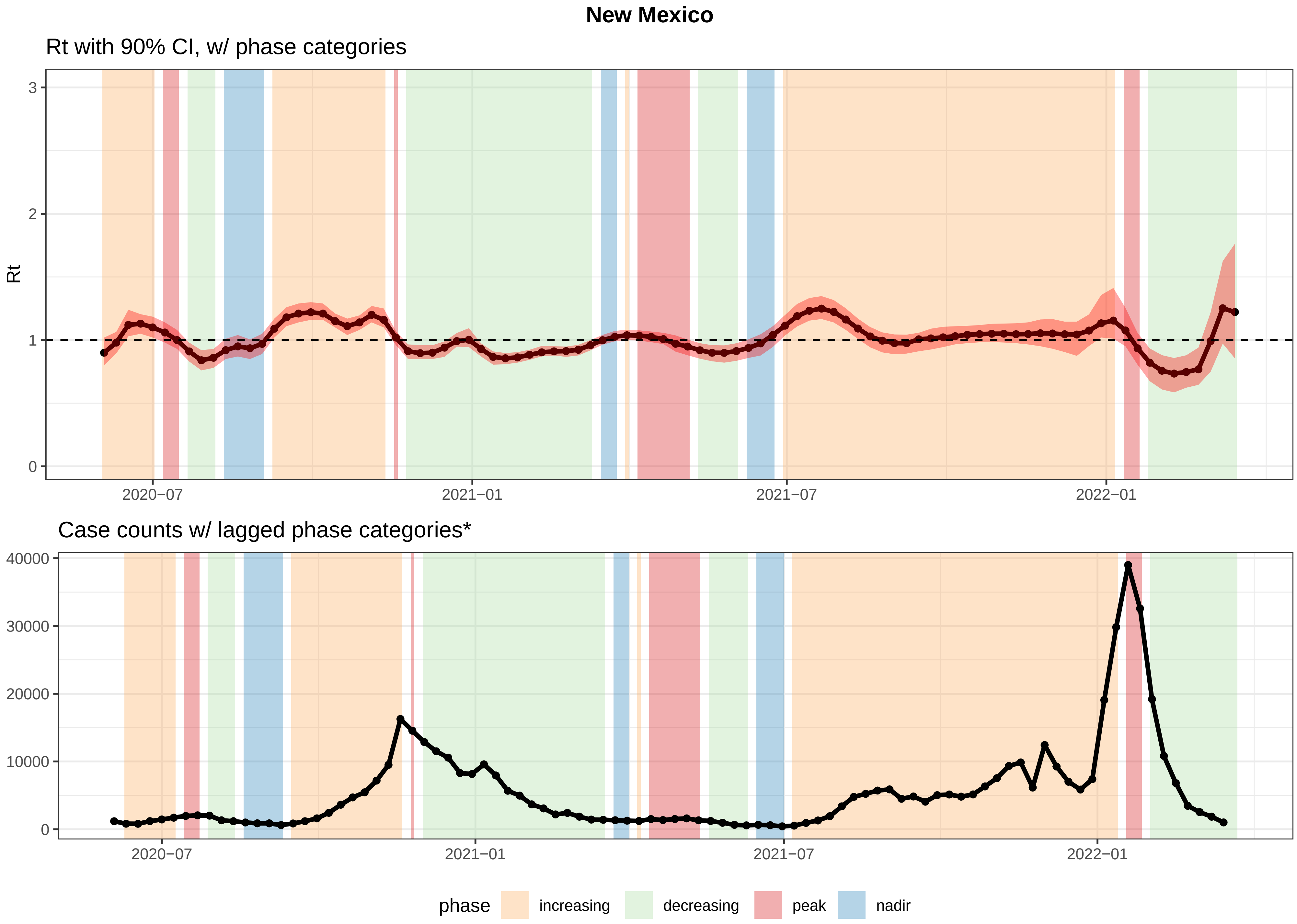

*Increasing/decreasing = Rt had a 90% probability >= or <= than 1.0.
Wks b/w two increasing/decreasing phases −> classified as increasing/decreasing.
Wks b/w increasing and decreasing phases = peaks; nadirs = wks b/w decreasing and increasing phases.

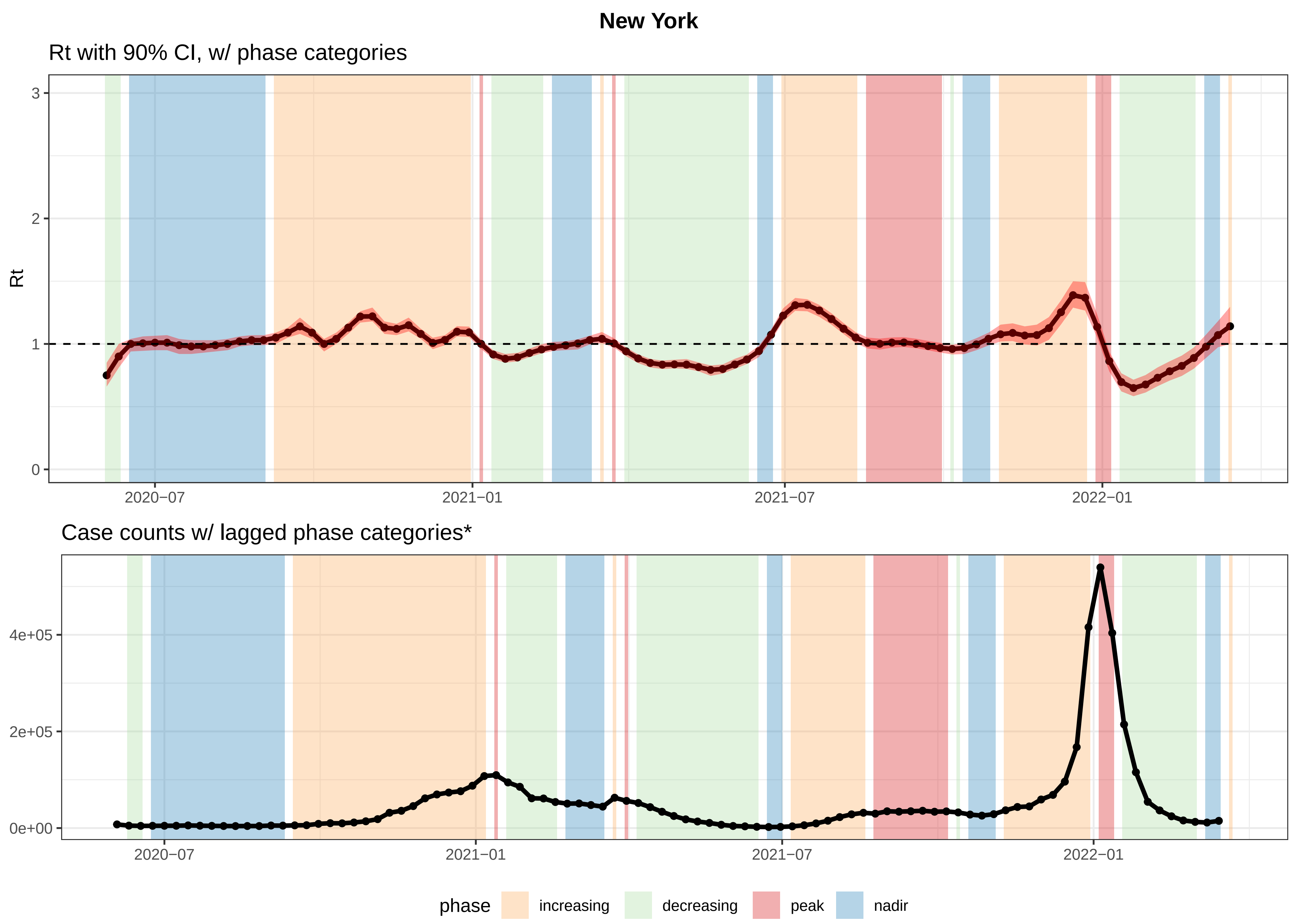

*Increasing/decreasing = Rt had a 90% probability >= or <= than 1.0.
Wks b/w two increasing/decreasing phases −> classified as increasing/decreasing.
Wks b/w increasing and decreasing phases = peaks; nadirs = wks b/w decreasing and increasing phases.

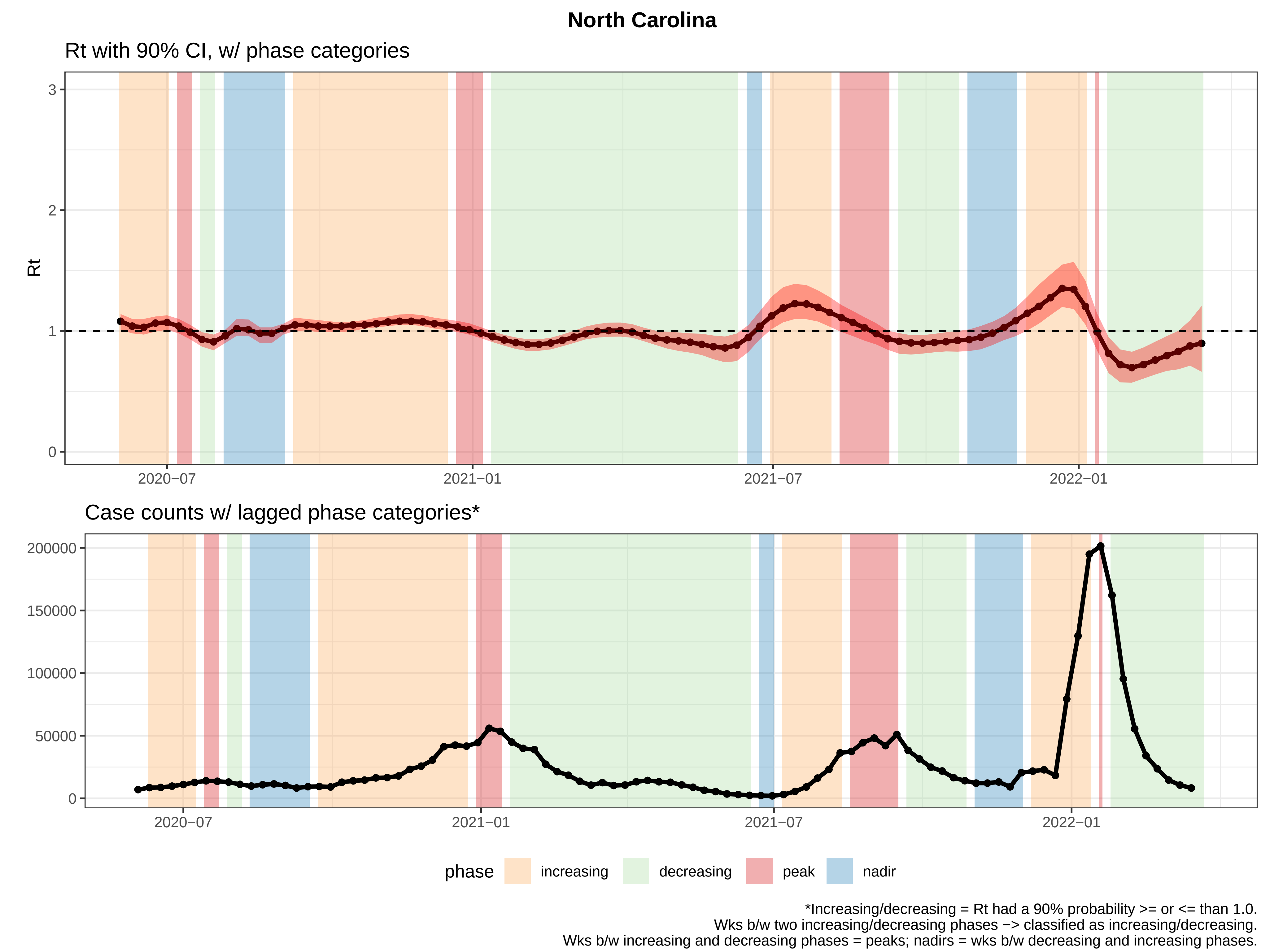
North Carolina
Rt with 90% CI, w/ phase categories
Rt
3
2
1
0
2020−07
2021−01
2021−07
2022−01
Case counts w/ lagged phase categories*
200000
150000
100000
50000
0
2020−07
2021−01
2021−07
2022−01
phase
increasing
decreasing
peak
nadir
*Increasing/decreasing = Rt had a 90% probability >= or <= than 1.0.
Wks b/w two increasing/decreasing phases −> classified as increasing/decreasing.
Wks b/w increasing and decreasing phases = peaks; nadirs = wks b/w decreasing and increasing phases.

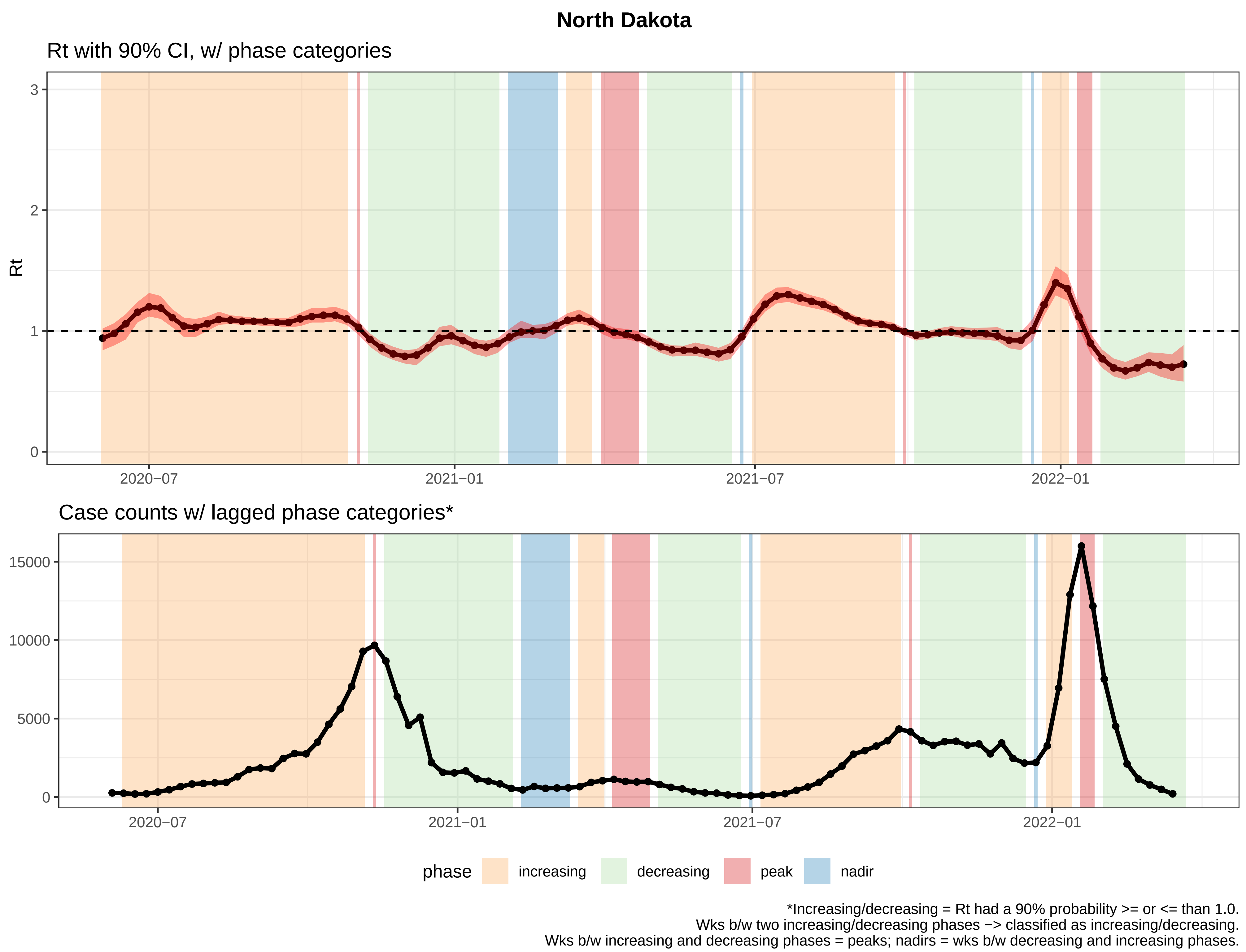
North Dakota
Rt with 90% CI, w/ phase categories
Rt
0
1
2
3
2020−07
2021−01
2021−07
2022−01
Case counts w/ lagged phase categories*
0
5000
10000
15000
2020−07
2021−01
2021−07
2022−01
phase
increasing
decreasing
peak
nadir
*Increasing/decreasing = Rt had a 90% probability >= or <= than 1.0.
Wks b/w two increasing/decreasing phases −> classified as increasing/decreasing.
Wks b/w increasing and decreasing phases = peaks; nadirs = wks b/w decreasing and increasing phases.

Ohio

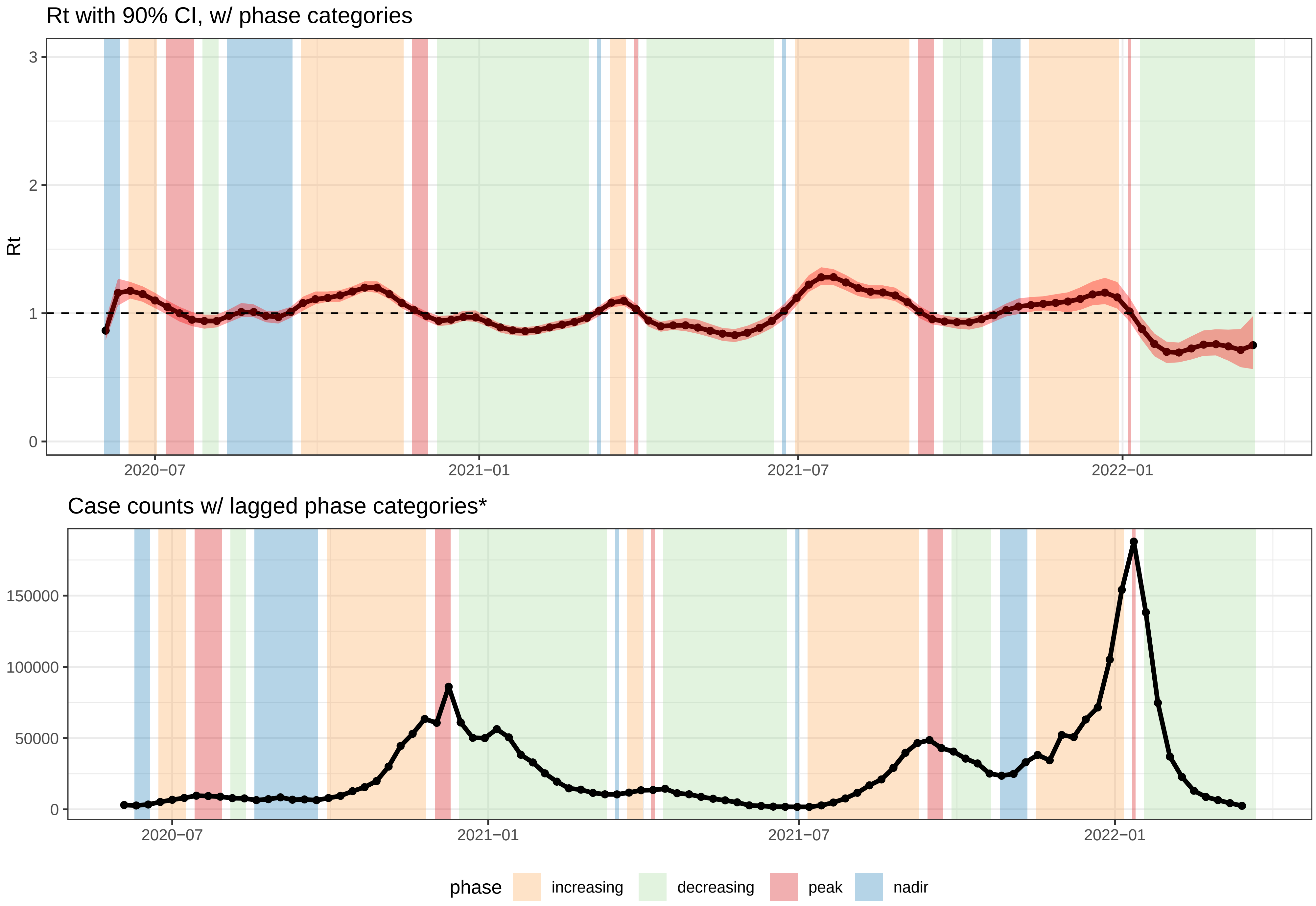

*Increasing/decreasing = Rt had a 90% probability >= or <= than 1.0.
Wks b/w two increasing/decreasing phases −> classified as increasing/decreasing.
Wks b/w increasing and decreasing phases = peaks; nadirs = wks b/w decreasing and increasing phases.

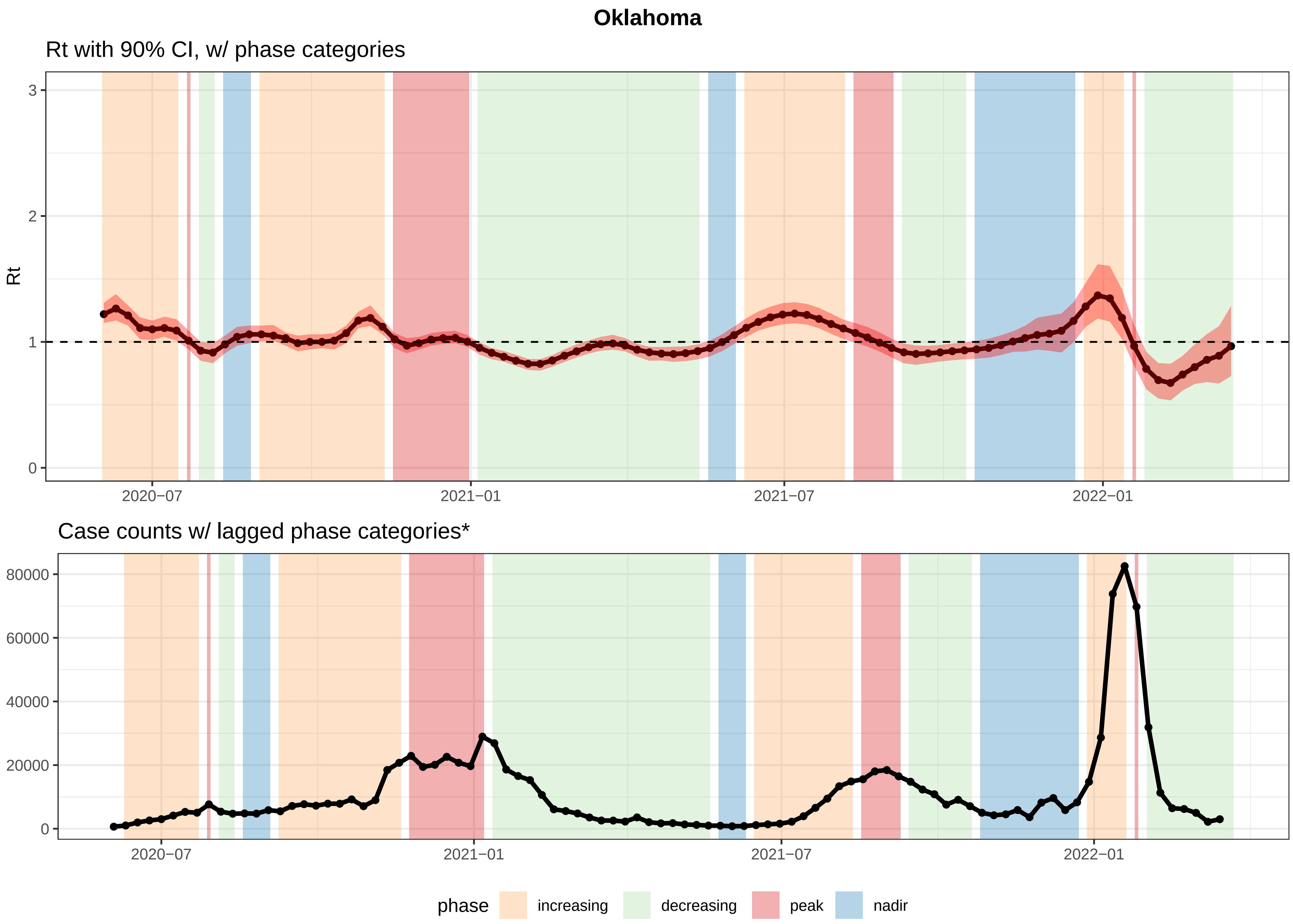

*Increasing/decreasing = Rt had a 90% probability >= or <= than 1.0.
Wks b/w two increasing/decreasing phases −> classified as increasing/decreasing.
Wks b/w increasing and decreasing phases = peaks; nadirs = wks b/w decreasing and increasing phases.

Oregon

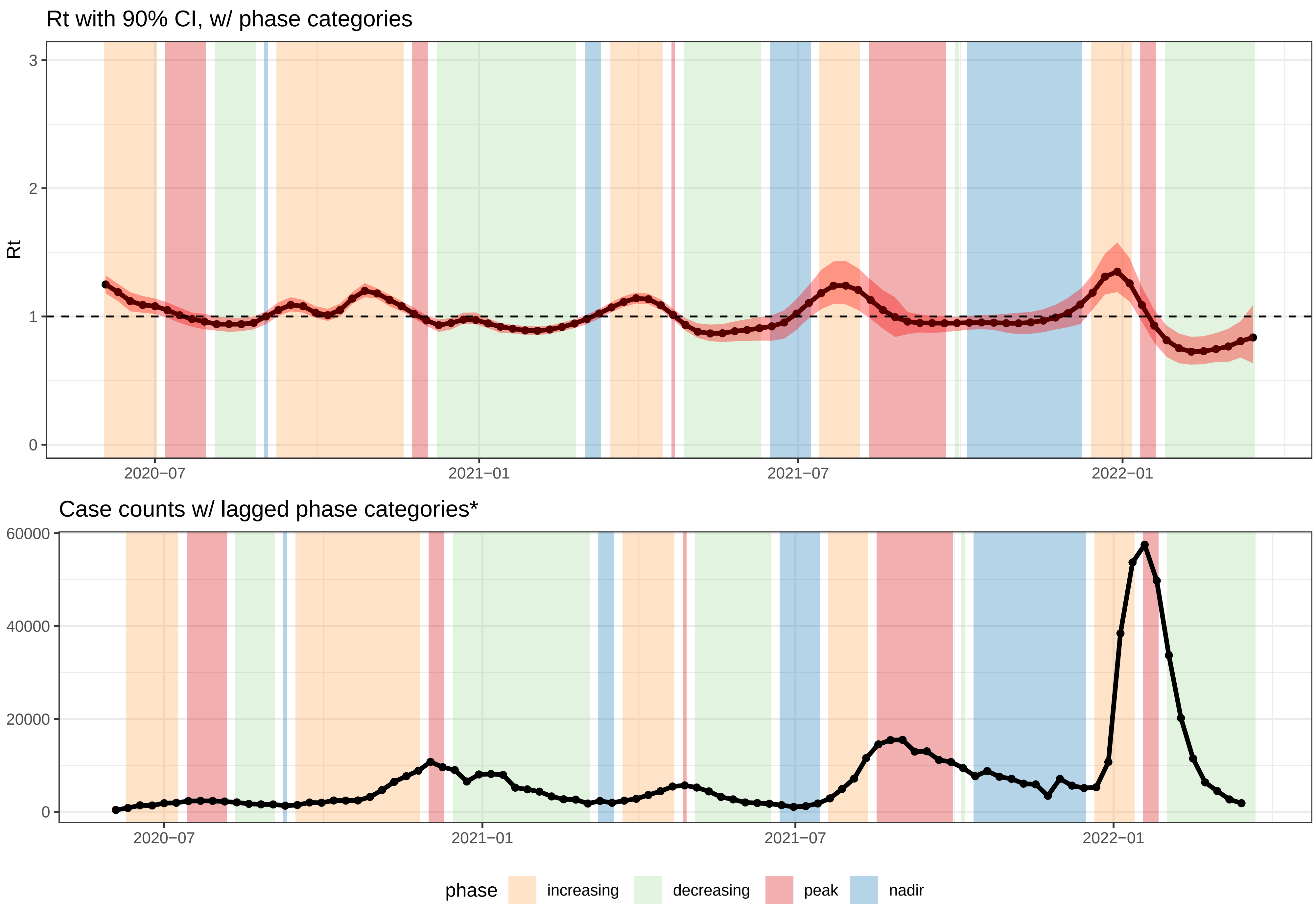

*Increasing/decreasing = Rt had a 90% probability >= or <= than 1.0.
Wks b/w two increasing/decreasing phases −> classified as increasing/decreasing.
Wks b/w increasing and decreasing phases = peaks; nadirs = wks b/w decreasing and increasing phases.

Pennsylvania

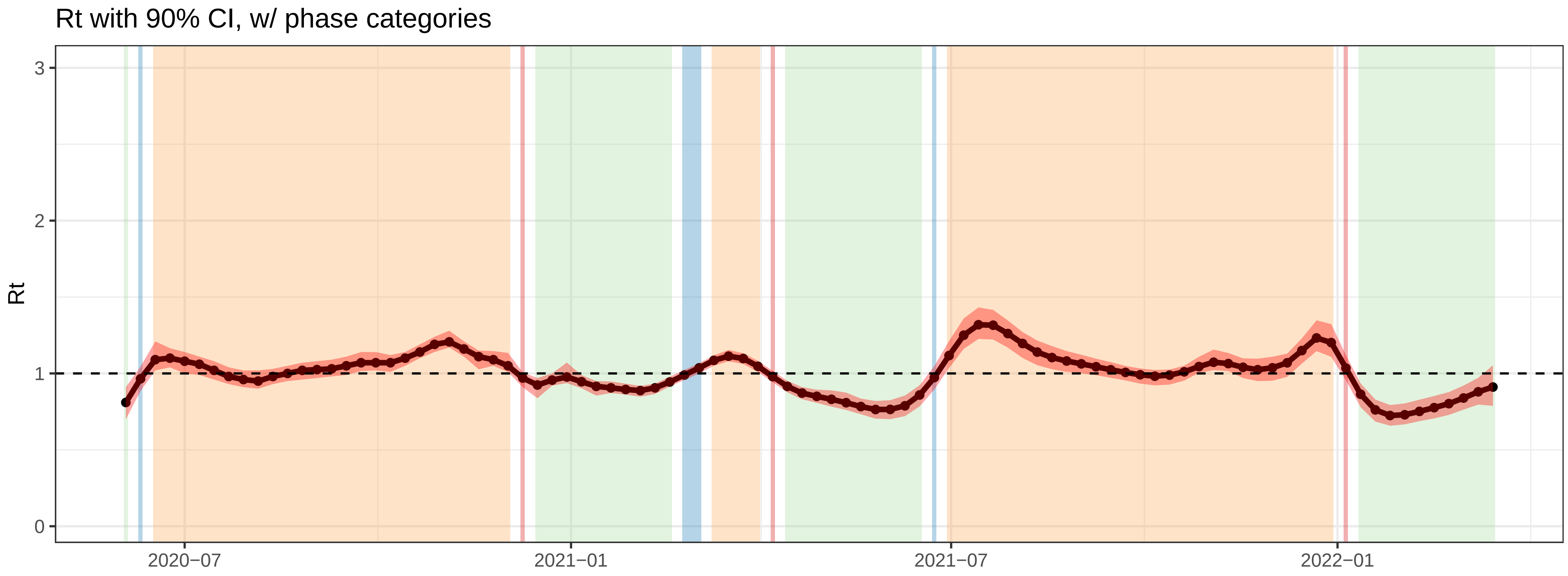

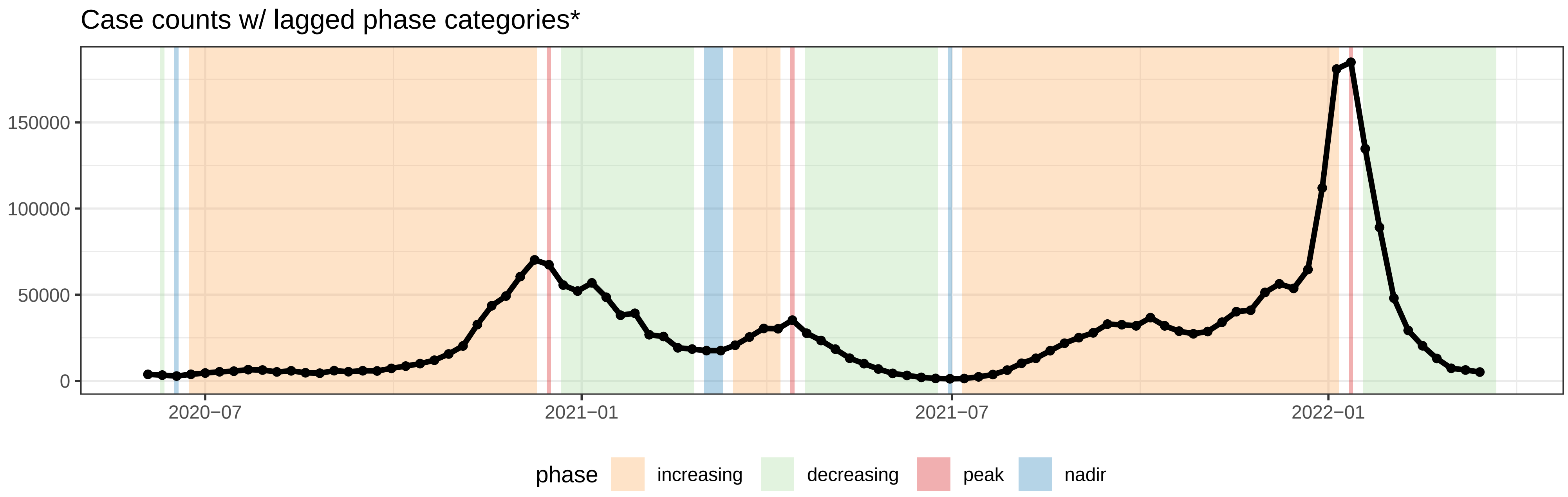

*Increasing/decreasing = Rt had a 90% probability >= or <= than 1.0.
Wks b/w two increasing/decreasing phases −> classified as increasing/decreasing.
Wks b/w increasing and decreasing phases = peaks; nadirs = wks b/w decreasing and increasing phases.

Rhode Island

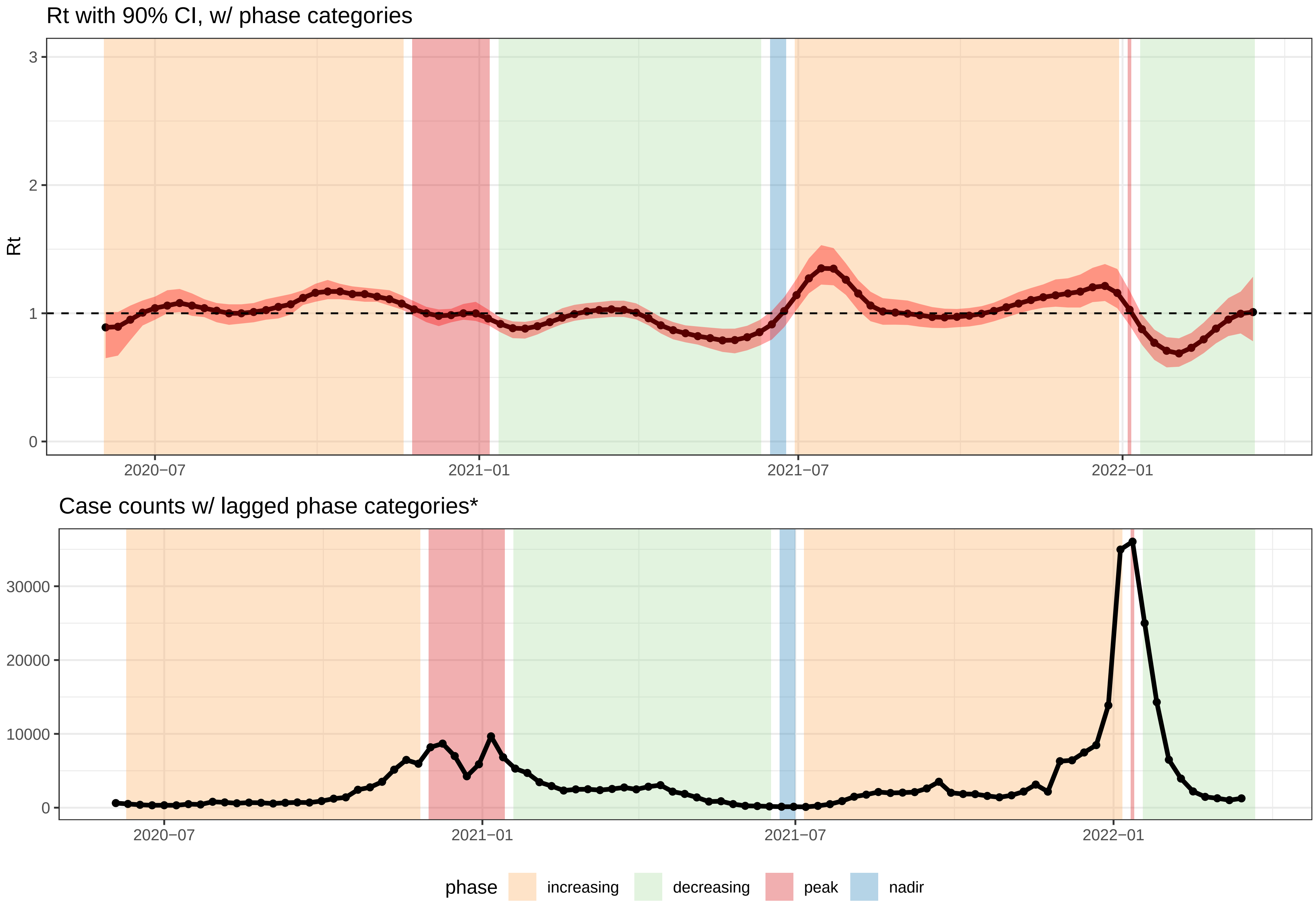

*Increasing/decreasing = Rt had a 90% probability >= or <= than 1.0.
Wks b/w two increasing/decreasing phases −> classified as increasing/decreasing.
Wks b/w increasing and decreasing phases = peaks; nadirs = wks b/w decreasing and increasing phases.

South Carolina

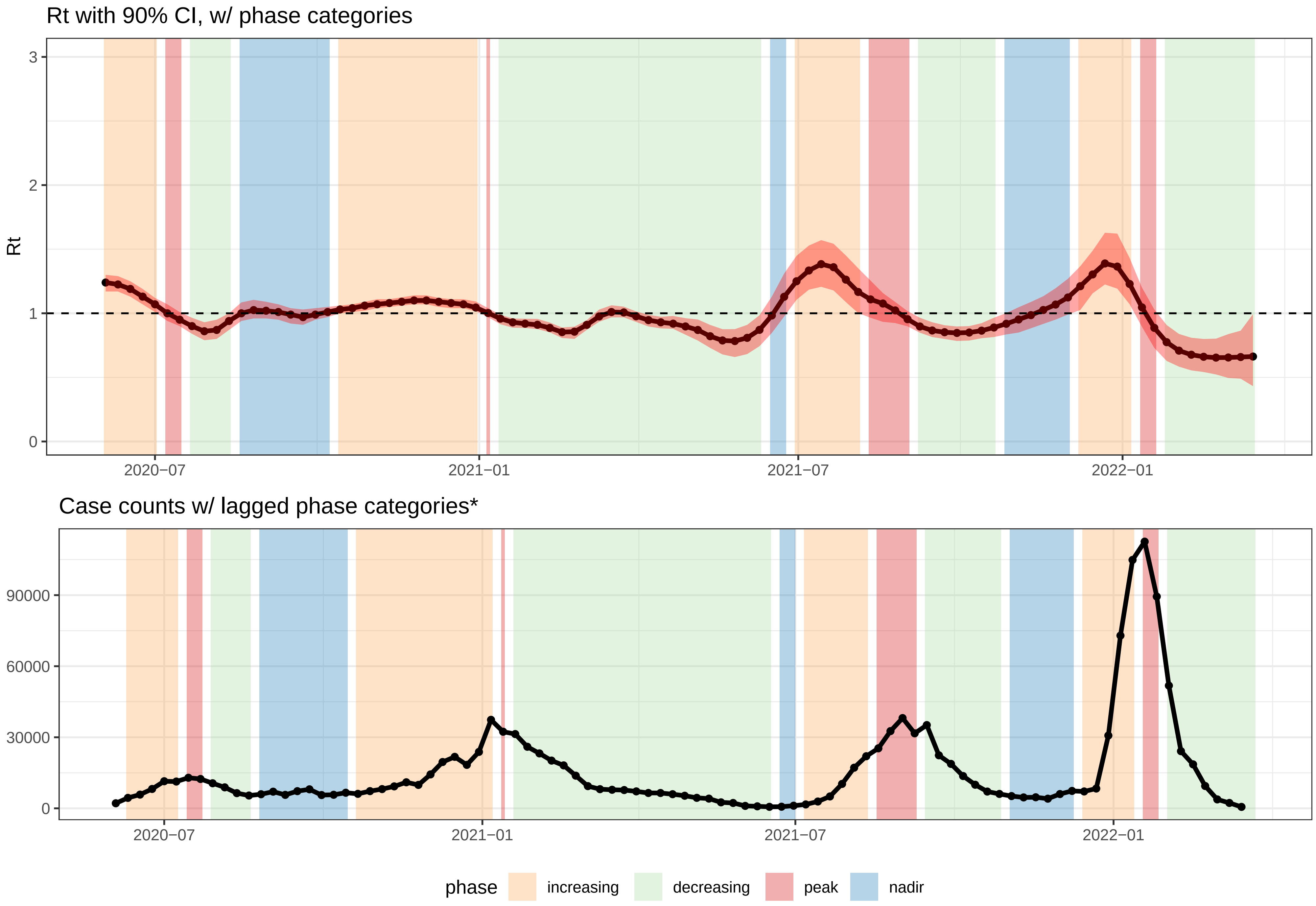

*Increasing/decreasing = Rt had a 90% probability >= or <= than 1.0.
Wks b/w two increasing/decreasing phases −> classified as increasing/decreasing.
Wks b/w increasing and decreasing phases = peaks; nadirs = wks b/w decreasing and increasing phases.

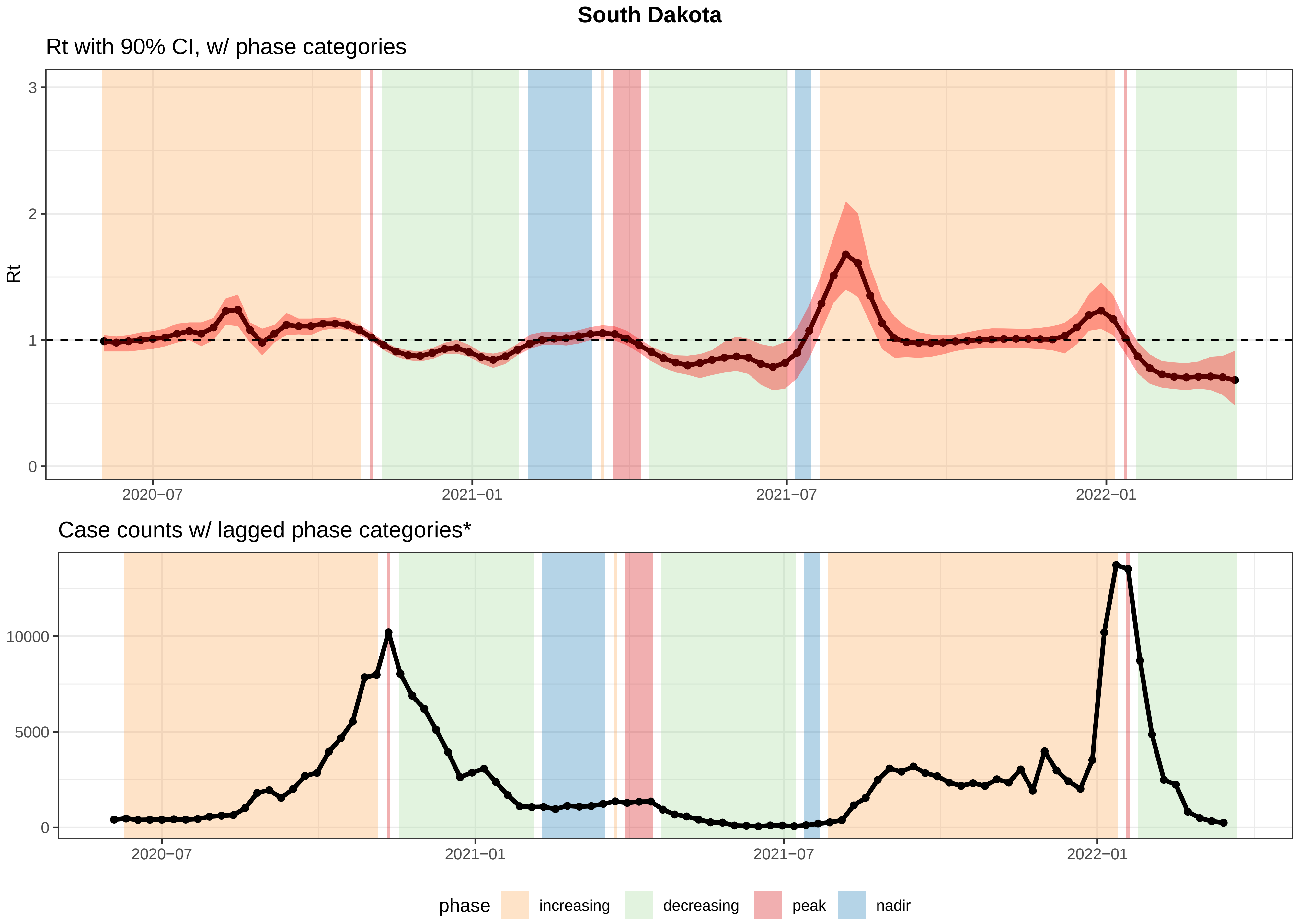

*Increasing/decreasing = Rt had a 90% probability >= or <= than 1.0.
Wks b/w two increasing/decreasing phases −> classified as increasing/decreasing.
Wks b/w increasing and decreasing phases = peaks; nadirs = wks b/w decreasing and increasing phases.

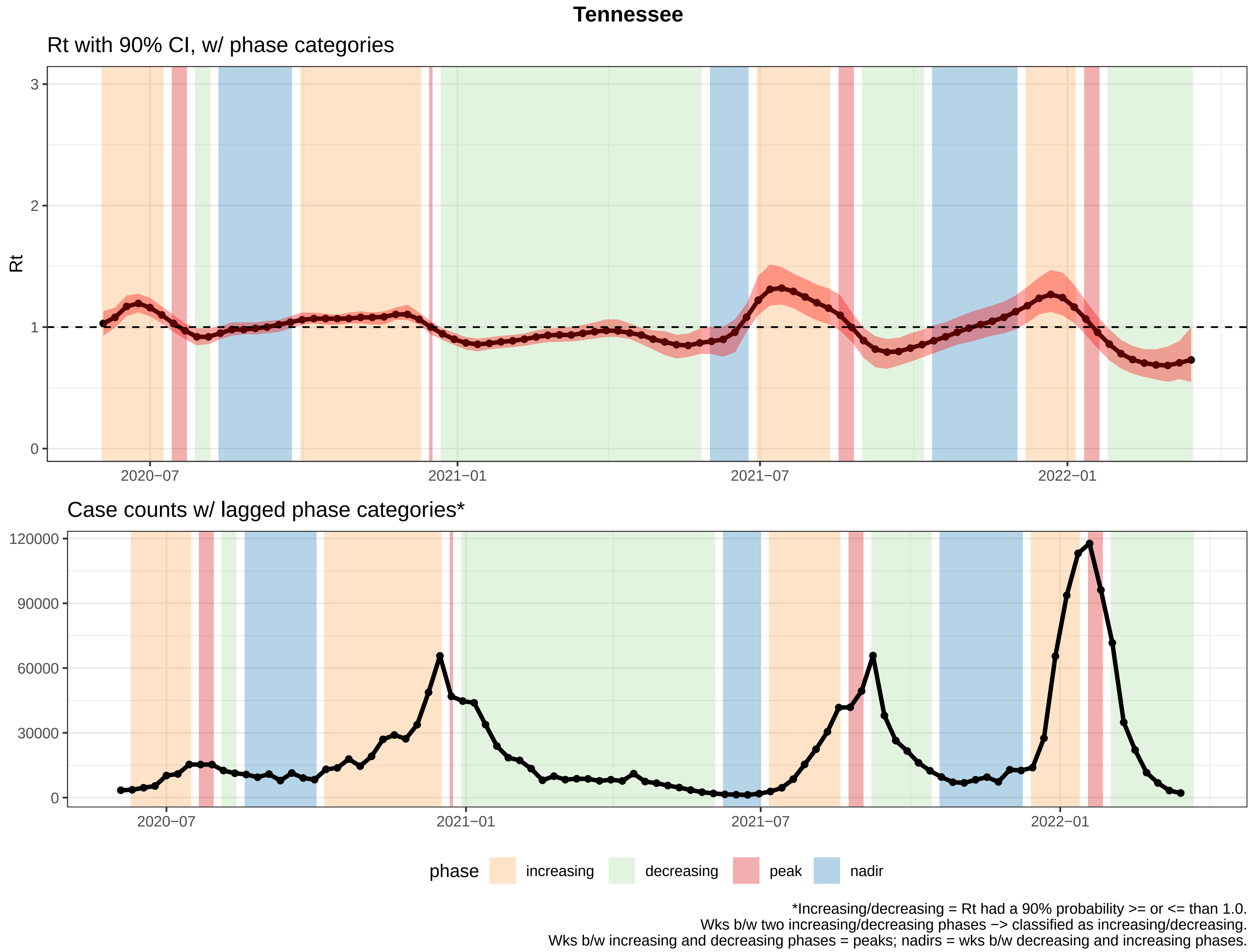
Tennessee
Rt with 90% CI, w/ phase categories
Rt
3
2
1
0
2020−07
2021−01
2021−07
2022−01
Case counts w/ lagged phase categories*
120000
90000
60000
30000
0
2020−07
2021−01
2021−07
2022−01
phase
increasing
decreasing
peak
nadir
*Increasing/decreasing = Rt had a 90% probability >= or <= than 1.0.
Wks b/w two increasing/decreasing phases −> classified as increasing/decreasing.
Wks b/w increasing and decreasing phases = peaks; nadirs = wks b/w decreasing and increasing phases.

Texas

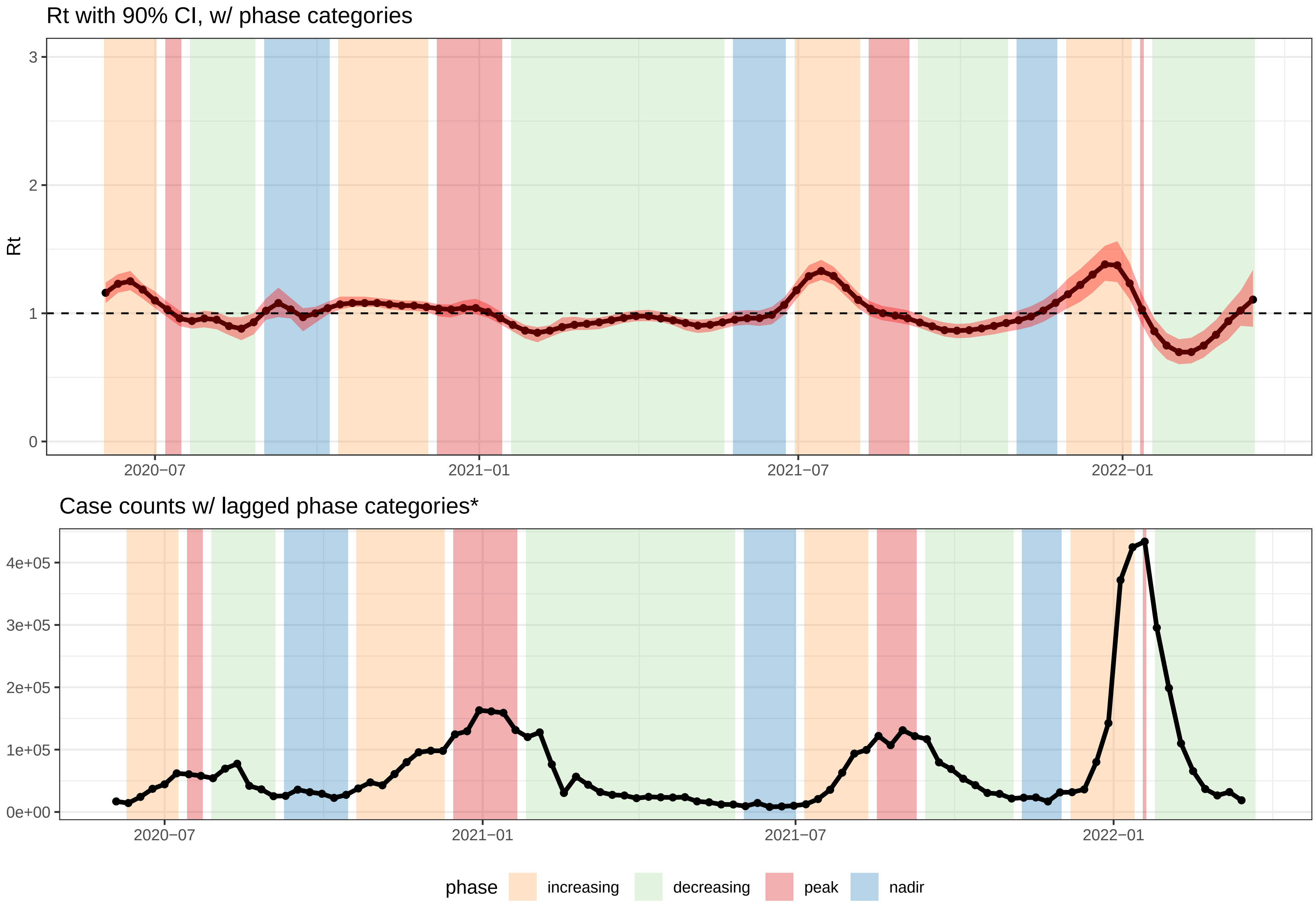

*Increasing/decreasing = Rt had a 90% probability >= or <= than 1.0.
Wks b/w two increasing/decreasing phases −> classified as increasing/decreasing.
Wks b/w increasing and decreasing phases = peaks; nadirs = wks b/w decreasing and increasing phases.

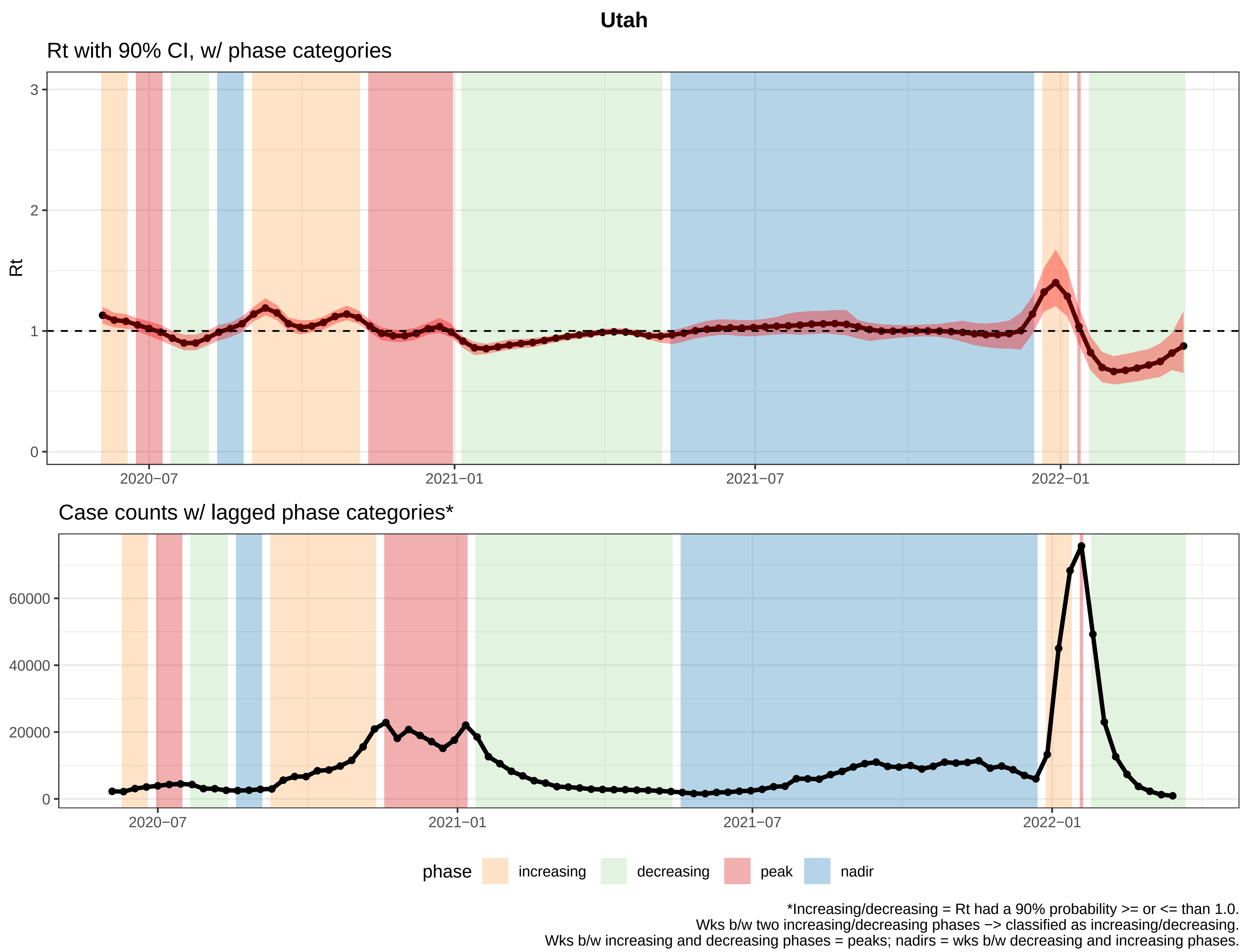
Utah
Rt with 90% CI, w/ phase categories
Rt
3
2
1
0
2020−07
2021−01
2021−07
2022−01
Case counts w/ lagged phase categories*
60000
40000
20000
0
2020−07
2021−01
2021−07
2022−01
phase
increasing
decreasing
peak
nadir
*Increasing/decreasing = Rt had a 90% probability >= or <= than 1.0.
Wks b/w two increasing/decreasing phases −> classified as increasing/decreasing.
Wks b/w increasing and decreasing phases = peaks; nadirs = wks b/w decreasing and increasing phases.

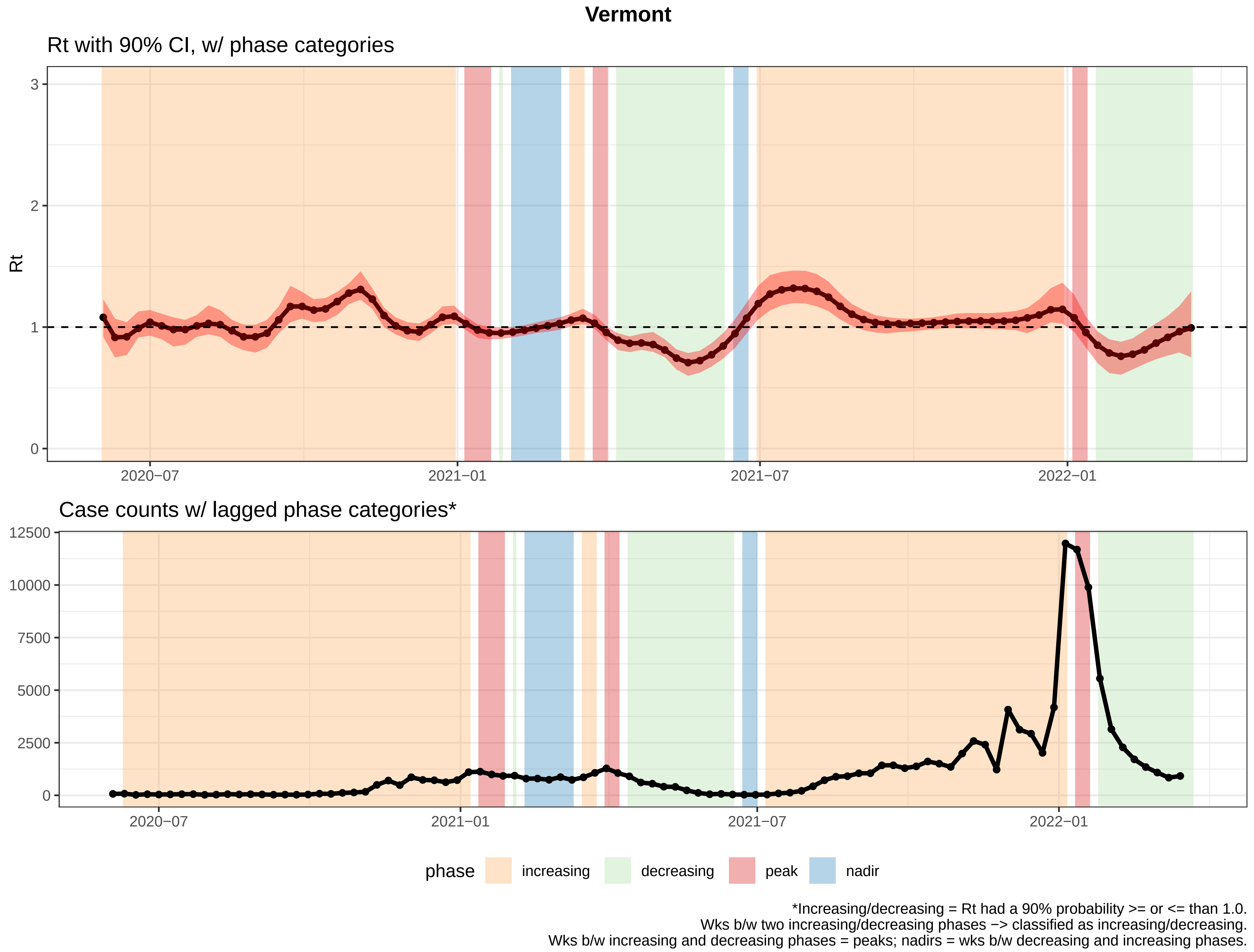
Vermont
Rt with 90% CI, w/ phase categories
Rt
3
2
1
0
2020−07
2021−01
2021−07
2022−01
Case counts w/ lagged phase categories*
12500
10000
7500
5000
2500
0
2020−07
2021−01
2021−07
2022−01
phase
increasing
decreasing
peak
nadir
*Increasing/decreasing = Rt had a 90% probability >= or <= than 1.0.
Wks b/w two increasing/decreasing phases −> classified as increasing/decreasing.
Wks b/w increasing and decreasing phases = peaks; nadirs = wks b/w decreasing and increasing phases.

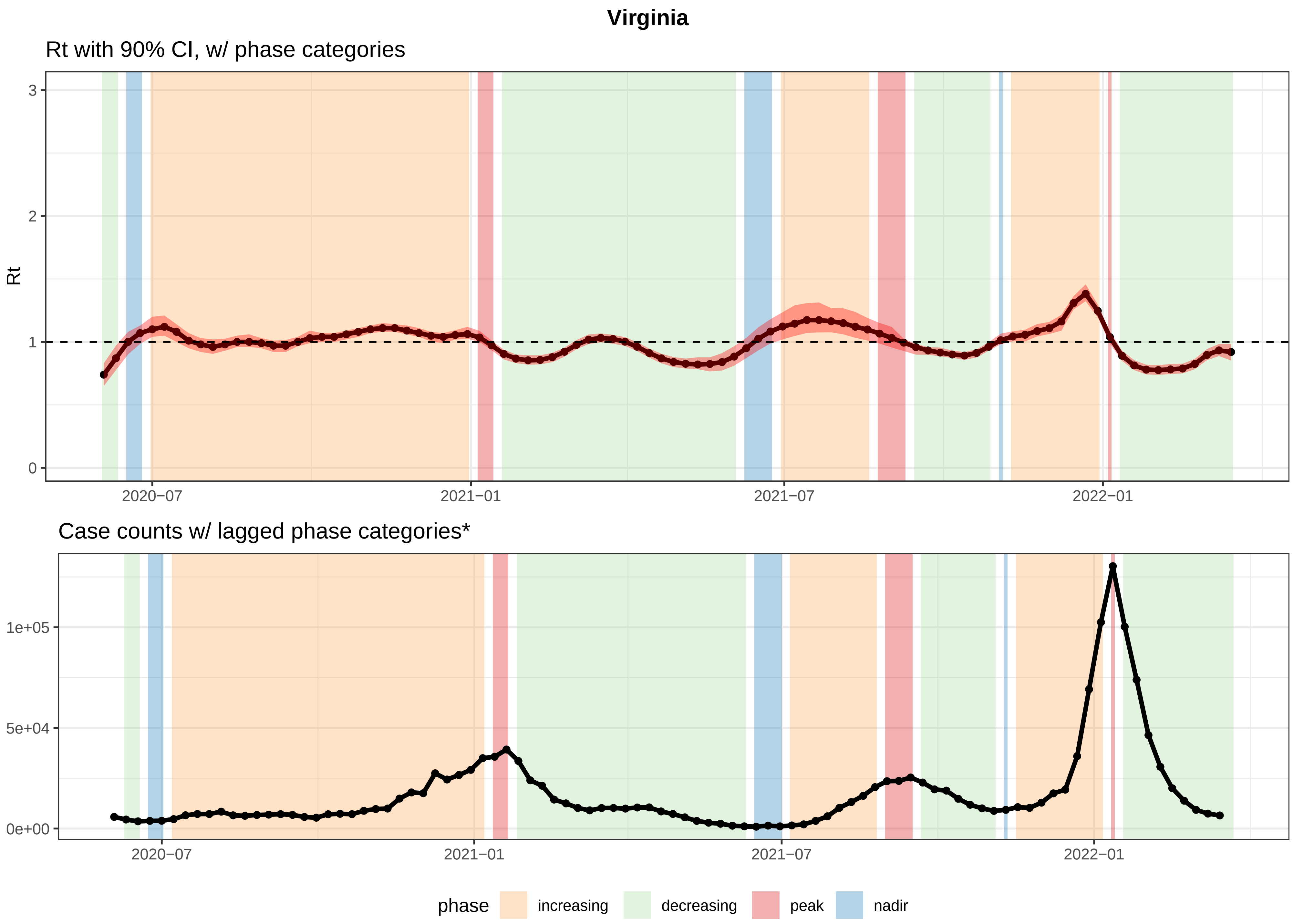

*Increasing/decreasing = Rt had a 90% probability >= or <= than 1.0.
Wks b/w two increasing/decreasing phases −> classified as increasing/decreasing.
Wks b/w increasing and decreasing phases = peaks; nadirs = wks b/w decreasing and increasing phases.

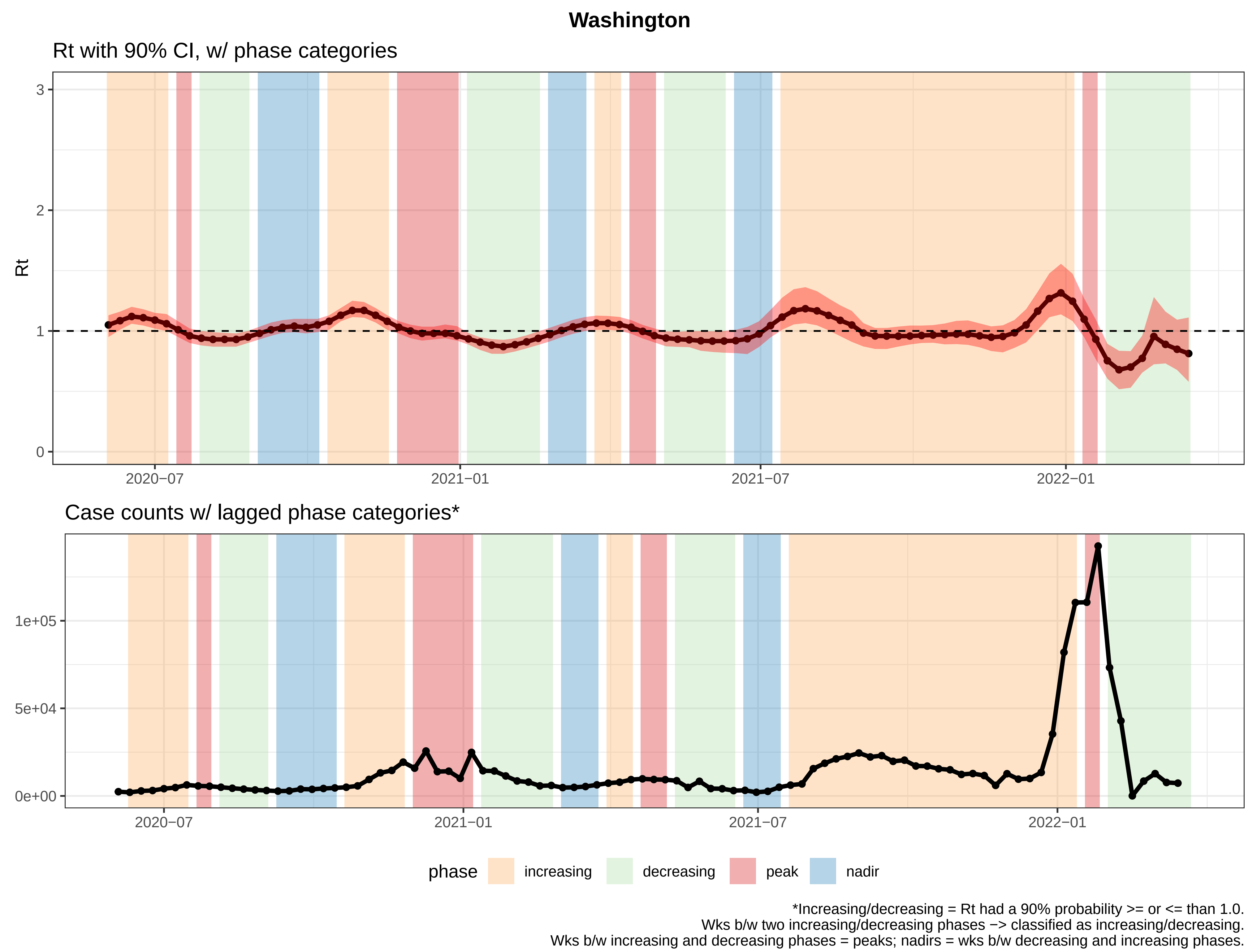

Washington
Rt with 90% CI, w/ phase categories
Rt
3
2
1
0
2020−07
2021−01
2021−07
2022−01
Case counts w/ lagged phase categories*
1e+05
5e+04
0e+00
2020−07
2021−01
2021−07
2022−01
phase
increasing
decreasing
peak
nadir
*Increasing/decreasing = Rt had a 90% probability >= or <= than 1.0.
Wks b/w two increasing/decreasing phases −> classified as increasing/decreasing.
Wks b/w increasing and decreasing phases = peaks; nadirs = wks b/w decreasing and increasing phases.

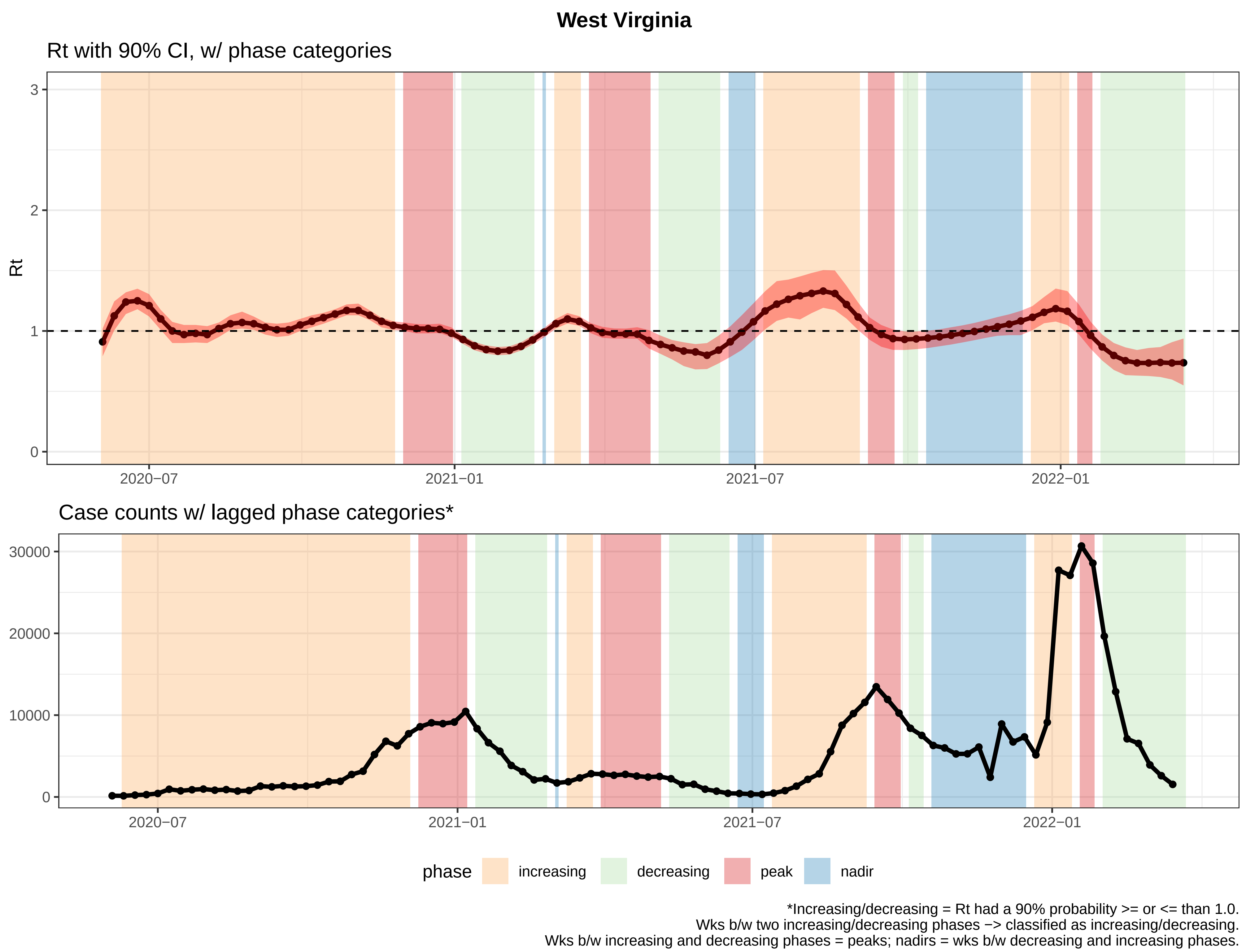
West Virginia
Rt with 90% CI, w/ phase categories
Rt
0
1
2
3
2020−07
2021−01
2021−07
2022−01
Case counts w/ lagged phase categories*
0
10000
20000
30000
2020−07
2021−01
2021−07
2022−01
phase
increasing
decreasing
peak
nadir
*Increasing/decreasing = Rt had a 90% probability >= or <= than 1.0.
Wks b/w two increasing/decreasing phases −> classified as increasing/decreasing.
Wks b/w increasing and decreasing phases = peaks; nadirs = wks b/w decreasing and increasing phases.

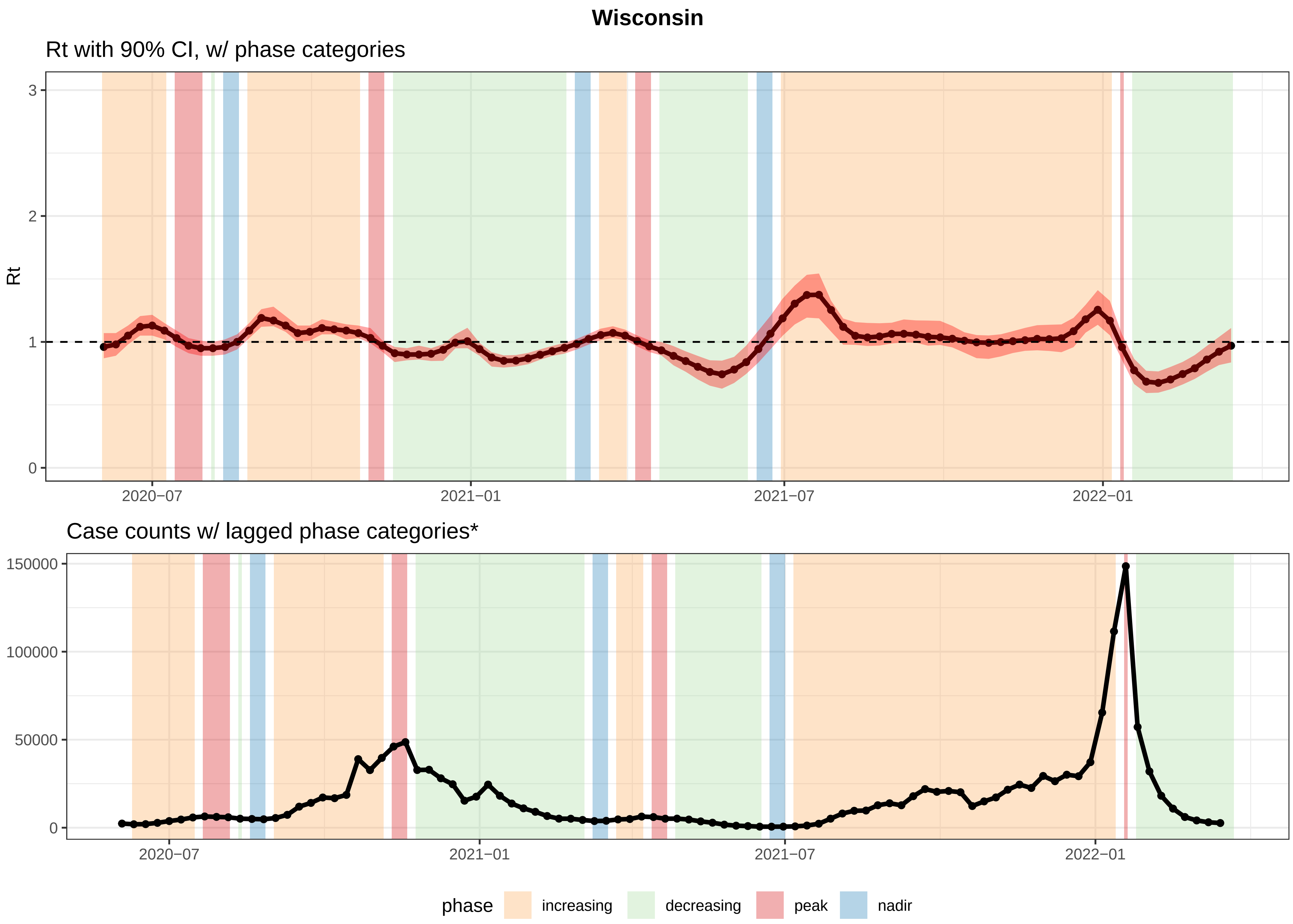

*Increasing/decreasing = Rt had a 90% probability >= or <= than 1.0.
Wks b/w two increasing/decreasing phases −> classified as increasing/decreasing.
Wks b/w increasing and decreasing phases = peaks; nadirs = wks b/w decreasing and increasing phases.

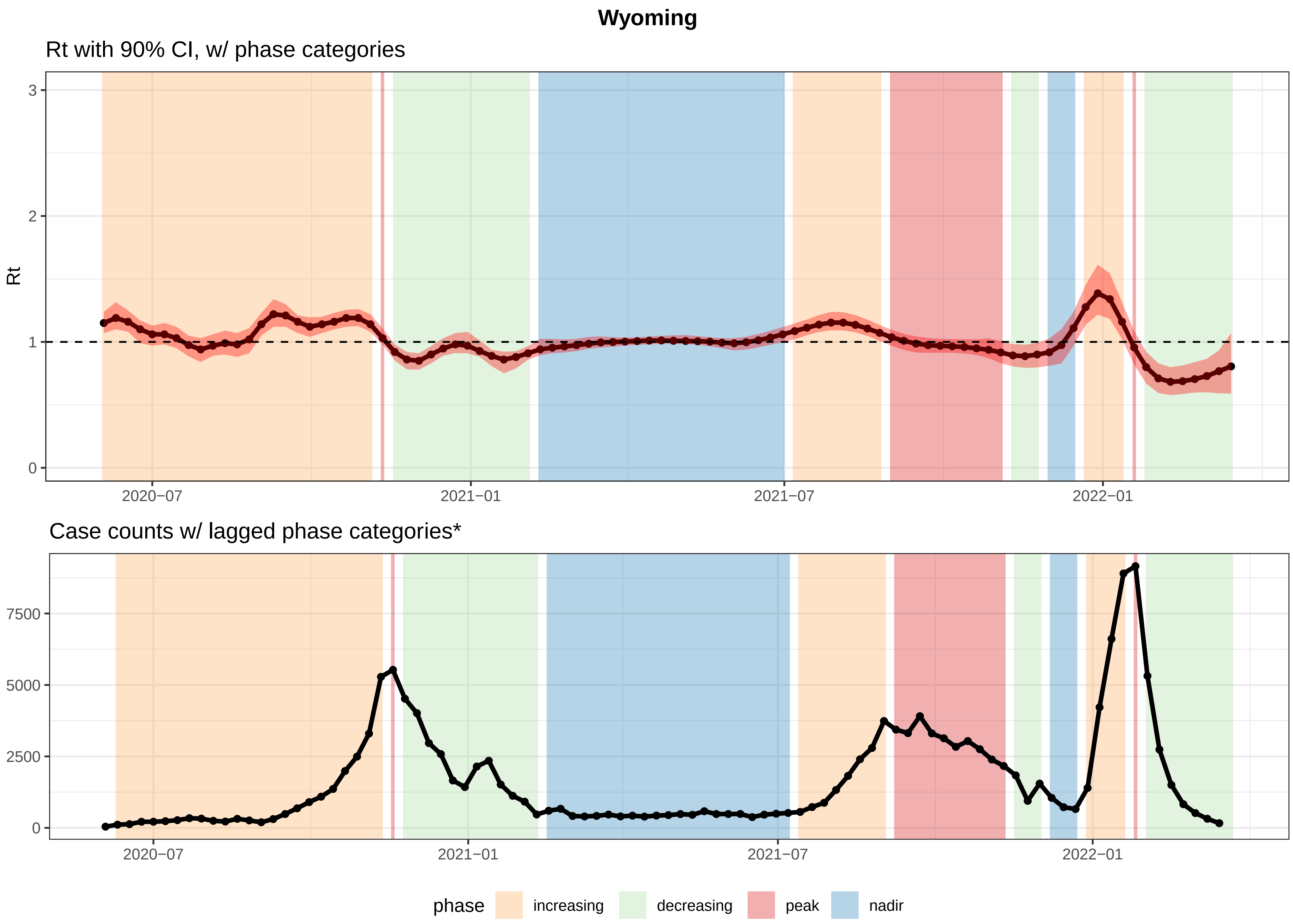

*Increasing/decreasing = Rt had a 90% probability >= or <= than 1.0.
Wks b/w two increasing/decreasing phases −> classified as increasing/decreasing.
Wks b/w increasing and decreasing phases = peaks; nadirs = wks b/w decreasing and increasing phases.
