# Supplementary material for: Challenges of COVID-19 Case Forecasting in the US, 2020–2021
Source: PLoS Comput Biol. 2024 May 6;20(5):e1011200. doi: 10.1371/journal.pcbi.1011200 (PMC11098513; doi:10.1371/journal.pcbi.1011200)

**S7 Appendix.** Phase- specific marginal mean Weighted Interval Score (WIS) over range of reported cases

**Fig A.** Each team model’s estimated marginal mean Weighted Interval Score (WIS) over range of reported case counts per epidemic phase. Marginal mean WIS was estimated from GEE model results and reflect values across the 95% confidence interval of mean reported cases. Case counts differ per team model as each team forecasted a different set of locations over a different range of possible dates.


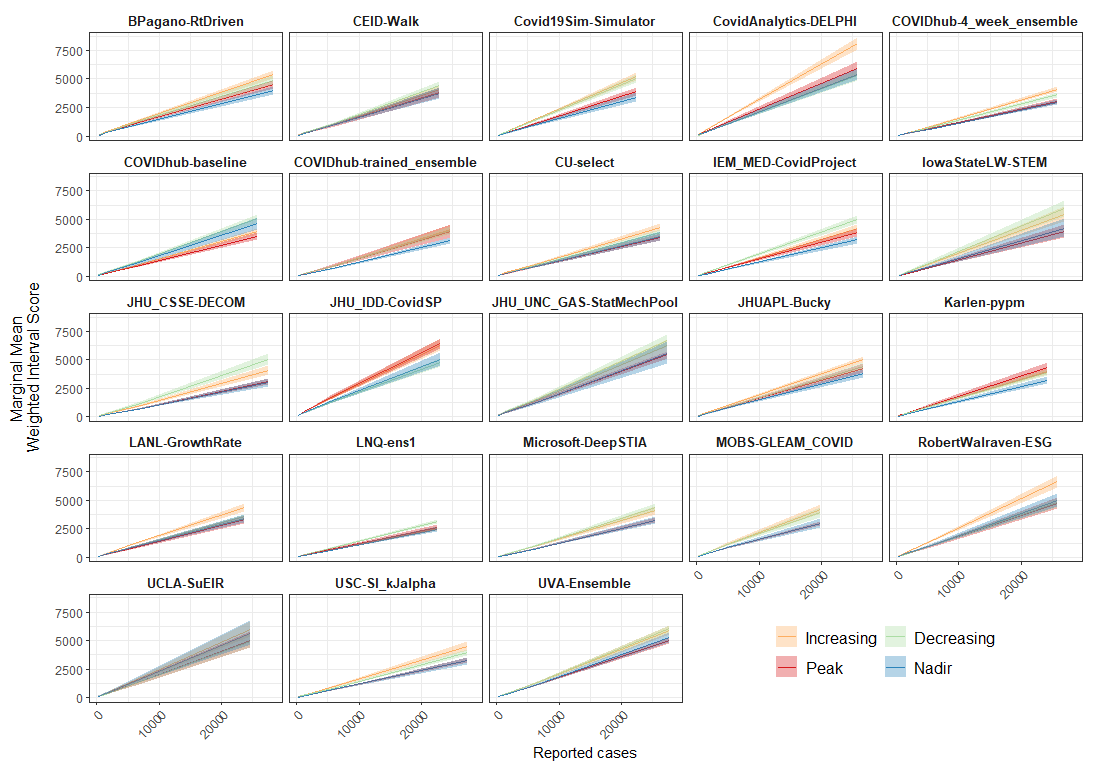

Supplement: S7 Appendix — Fig A. Each team model’s estimated marginal mean Weighted Interval Score (WIS) over a range of reported case counts per epidemic phase. Marginal mean WIS was estimated from GEE model results and reflects values across the 95% confidence interval of mean reported cases. Case counts differ per team model as each team forecasted a different set of locations over a different range of possible dates. (DOCX) [file pcbi.1011200.s007.docx]
