# Supplementary material for: Challenges of COVID-19 Case Forecasting in the US, 2020–2021
Source: PLoS Comput Biol. 2024 May 6;20(5):e1011200. doi: 10.1371/journal.pcbi.1011200 (PMC11098513; doi:10.1371/journal.pcbi.1011200)
Supplement: Attachment — Submitted filename: Reviewer responses_PCOMPBIOL-D-23-00808.docx [file pcbi.1011200.s009.docx]

**The reviewer comments have improved the quality of the manuscript, and we’re grateful for their time and thoughtful critique. We’ve addressed all comments from each reviewer and made appropriate changes in the resubmitted manuscript text. All line numbers below refer to the track-changes version of the updated manuscript.**

**Reviewer #1:** This paper analyzes the retrospective performance of COVID-19 case forecasts submitted to the forecast Hub. It describes the performance of these forecasts over time and across geographic units, and contrasts the performance of individual forecasts with that of ensemble models. It identifies that case forecasts were relatively unreliable (e.g., had low coverage rates for confidence intervals), particularly during periods of rapid increase, though ensemble models typically at least outperform simple baselines. It discusses directions that may be explored to improve future forecasting performance.

This is a much-needed and well executed paper. The fact that COVID case forecasts did not work well at key points during the pandemic is well known in the forecasting community, and a systematic attempt to document and understand this is an important part of moving the field forward. Overall, the analysis is quite thorough and most of my natural questions were answered in the supplemental material. The writing is clear throughout.

A couple of questions for the authors to consider:

(1) The results showed variation in forecast performance across locations. Is forecast performance spatially correlated? Either at the state level, or for counties within states? It would be quite interesting to see a choropleth of forecast skill and if there are properties of locations where forecasts performed better/worse. Beyond this, are residuals (as opposed to skill) spatially correlated? If so, this would argue in favor of models that incorporate explicit spatial structure, which is one of the recommendations made in the discussion section.

**Thank you for bringing up this point. Spatial correlation is intrinsic to COVID-19 spread and is also likely reflected in the forecasts. Consequently, it is challenging to parse out how spatial correlation influences the interpretation of forecast skill. We added an additional supplemental figure to assess the spatial correlation in rWIS (to minimize the scale-specific effects) and found that spatial correlation in rWIS was present in some but not all models (see new Supporting Information 4). We refer to this result in the main text at line 248, “Spatial correlation is intrinsic to COVID-19 spread making correlation in the forecasts likely regardless of forecast skill. We found some evidence of spatial correlation in rWIS for many team models (ensemble Moran’s I: 0.36, p-value =0.001, S4).” We also added to the discussion (lines 398-399), “Many of the forecasts here, including the top performing ones, showed indications of possible spatial correlation in state-level performance, suggesting that spatial dynamics may not be accounted for fully.”**

(2) Is there a measure of directional correctness for predictions? I.e., at the start of periods marked as increasing/decreasing, what fraction of the time did forecasters successfully guess the direction compared to the success rate of the baseline model? This would provide some concrete evidence about whether epidemic phase was "known" at the time to forecasters and they missed on magnitude, or whether there was also significant uncertainty about phase as well.

**Unfortunately, there is not a standard metric of correct predicted direction that we can apply to the forecast data we collected (i.e., quantiles) . To determine the direction of the forecasts, we compared each 50% prediction interval (PI) to the last known incidence value (also, the baseline model median). Because the baseline predicts a flat line, if any model’s 50% PI lower limit was greater than the baseline median values, it was considered an “increasing prediction” as this indicates a 75% or greater chance of that value exceeding the most recent reported data. Forecasts with the upper limit less than the baseline were considered “decreasing predictions” (75% of the outcome being lower than the last value). And forecasts with 50% PIs that contained the baseline median were considered “stable or uncertain trajectory”. Using these direction classifications, we calculated the percent of forecasts with each predicted direction that fell into eventually observed epidemic phases (see new Figure 8). We added a description of this approach in the Methods (see lines 644-647):**

**“To determine the direction of the forecast predictions, each model’s 50% PI was compared to the last known incidence value. Forecasts were categorized as *increasing predictions* when the lower limit of their 50% PI was greater than the last known value, *decreasing predictions* when the upper limit of their 50% PI was less than the last known value, and *stable/uncertain* if their 50% PI contained the last known value.”.**

**We also included the following text in the Results (see lines 307-311):**

**“We classified each forecast as increasing, decreasing, or stable or uncertain based on the 50% prediction interval relative to the most recent observed value. If the forecasts were able to correctly predict the direction of the epidemic phase, we would expect a high percentage of forecasts with an increasing trajectory to occur in an increasing epidemic phase, and likewise, a high percent of stable/uncertain trajectory forecasts in the peak and nadir phases and a high percent of forecasts with a decreasing trajectory in the decreasing epidemic phase. Increasing forecasts were most commonly made for the peak phase (predicting continued increases rather than a peak) followed by the increasing phase (Figure 8A), though overall the most common forecasts were for stable or uncertainty trajectories (Figure 8B). While the percent of decreasing forecasts for the decreasing epidemic phase was high, forecasts often predicted declines for the nadir as well and some teams also frequently predicted decreases for the increasing phase (Figure 8C). ”.**

Minor typo: on line 360 there is a missing word in the phrase "clear association with forecast.."

**Thank you- this typo has been fixed. It was missing the word “skill”.**

**Reviewer #2: Minor comments.**
1. It is a shame that ignorance score (log likelihood) are not provided, since they are also proper scores and have the locality property. It could help in future systematic reviews of forecasting. Could the authors give us some idea about how a different choice of proper score might have changed any conclusions?

**Our analysis, like other COVID-19 forecasting work, evaluated forecast performance using Weighted Interval Scores, which approximates the Continuous Ranked Probability Score (CRPS), a common proper score used in the forecasting literature. We agree with the reviewer that including log scores could provide additional insight into forecast performance. However, we chose not to use log scores because of the submitted forecast formats. Forecasts were collected as quantiles rather than probability distributions to facilitate the production of forecasts from a variety of model types, outcomes, and outcome scales. The choice of quantiles means that log scores cannot be directly calculated from the submissions, without making potentially consequential assumptions about the form of the tails of the distribution outside of the provided quantiles. The selection of scoring criteria is important and is addressed elsewhere (e.g., Bracher et al., Bosse et al.) and we have added a note about this in the Discussion (lines 424-426):**

**“First and foremost, due to the submitted format of the forecasts and the overall goal of soliciting diverse models, our analysis was limited to WIS and prediction interval coverage. Other proper scoring metrics with different characteristics have been used in other challenges and may provide different insights.”**

**Nevertheless, the forecast data are publicly available and individuals may reanalyze them using log scores if desired.**

2. About 1/3 of the submitted forecasts were excluded, but it is not clear that this exclusion is justified. it would be worthwhile knowing how those other forecasts performed.

**We expanded this to be clearer. There were two general categories of excluded forecasts: those that did not have the full 4 weeks of predictions and those that only forecasted for limited dates or locations. Given the open nature of the Forecast Hub, team submissions were variable by location and over time (see Supporting Figures 1.1). The pairwise relative WIS calculation adjusts for forecasting difficulty for teams that don’t forecast all the same dates. While this partially adjusts for minor differences in periods covered, the difference can be exacerbated when there are long periods of missing forecasts. Thus, we attempted to create a comparable set of forecasts for comparison by setting inclusion criteria to reduce any bias resulting from the lack of generalizability across submissions (e.g., teams that submitted only in periods with flat trajectories could have better scores). Similar inclusion criteria have been implemented by others evaluating open forecasting challenges (see Cramer et al, 2022, for example).**

**We clarified this reason in the Results section (lines 155-157): “We first assessed the locations, horizons, and periods forecasted by each team to ensure that forecasts included all required quantiles and horizons and to limit comparisons to models with substantial overlapping spatiotemporal coverage.” Also on lines 518-519, in the Methods section: “Application of these inclusion criteria provided a more comparable set of forecasts for scoring with the attempt to reduce biased scores if teams only forecasted for a limited number of locations or weeks.”**

3. In the discussion, it is worth mentioning that some very influential forecasts or projections (call them what you will) at the beginning of the pandemic were worse than worthless. It is not necessary to mention the IHME projections by name, but their projection envelopes made in the spring of 2020 went down near zero for late summer 2020. People listened.

**We agree with the reviewer that this is a critical point, and explicitly reference the influence of forecast performance on policy and behavioral choices (see Introduction, lines 116-117). Transparent and public evaluation is important for pushing this science forward, holding all models accountable for what they said at critical moments, and is part of our overall goal with this manuscript.**

**In regards to IHME’s forecast performance, IHME did not submit sufficient forecasts to be analyzed here, but they did submit death forecasts to the COVID-19 Forecast Hub and we found poor forecast performance in general with further degradation at longer horizons ( see** [**Cramer et al, 2022**](https://www.pnas.org/doi/10.1073/pnas.2113561119)**). We added a reference to our forecast horizons findings to the Results (lines 219-220); “We also found that calibration, WIS, and prediction interval coverage were all worse at 4-week horizons compared to 1-week horizons (S6).” And modified the Discussion to make a broad comment on this (lines 4665-466):** “**While the COVID-19 case ensemble forecasts were good leading indicators at many points in time, they were highly variable across teams and unreliable at longer horizons and during periods of rapid change.”**

4. We don't get a full sense of why these forecasts were or were not performing well. One suspects that much of what was going on in the first year for sure was related to behavior-driven changes. People relented in their precautions, and a surge happened. People then became more careful. After the first year, vaccination and changes in immunity and viral evolution must have been important contributors.

**We agree with the Reviewer that we have limited evidence on why forecast performance was overall poor. While your suggestions are in line with our thinking, these components were addressed in some way in some of the models. For example, three models included some version of short-term behavior change (see Results). So it is hard to piece out the key drivers. We tried to clarify this in the Discussion (lines 367-375):**

**“Seasonal changes in transmission biology and human behavior, emergence of variants, and changing mitigation behavior all contribute to transmission dynamics. While some forecasting models incorporate seasonality and variants, only three models included some version of short-term behavior change and characterizing the interaction between behavior and transmission has lagged** [**(24–26)**](https://www.zotero.org/google-docs/?oTTQD4)**. Nevertheless, even with the benefit of additional data, it is challenging to build transmission models that can capture all of the rapid change-points in cases, which were one of the foremost leading indicators.”**

**We also outlined possible research avenues for future work to tease apart this question (see lines 365 and 459-462).**

5. It is hard to tell whether or not the data analysis fully adjusts for potential nonindependence of the outcomes. This needs to be very carefully explicated and made clear beyond doubt. (note line 563).

**Thank you for pointing out the need for clarity here. We initially explored GEE models with autoregressive-1 correlation structure, however, these models did not converge for all teams. Hence, we opted for a more flexible, but equally robust, model with an independent working correlation structure (see Liang and Zeger, 1986 for a discussion of robust GEE standard errors with minimal differences resulting from correlation structure selection). Each team-specific GEE model is estimated at the state level to account for the correlated nature of the data. We have made this clearer on line 614, “This structure assumes that observations are correlated within a state (denoted as** $l$ **in the equations below), but not correlated over time in said state.”**

**Liang, K. Y., & Zeger, S. L. (1986). Longitudinal data analysis using generalized linear models. *Biometrika*, *73*(1), 13-22**

[**https://academic.oup.com/biomet/article/73/1/13/246001**](https://academic.oup.com/biomet/article/73/1/13/246001)

6. The paper says it contains all the code for figures and tables, but is there anything else in the results that involves unshared code?

**The GitHub repository provides all code needed to score the forecasts, run the regression models, and create the figures. While we cannot regenerate the team forecast data, we have added code for pulling all submitted forecast data from a cloned branch of the US COVID-19 Forecast Hub repository.**

**Reviewer #3: ** General comments**

This paper investigates case forecasts made in the US COVID-19 forecasting hub between July 2020 and December 2021. As similar previous and contemporary papers of this nature it is struggling to come to very firm conclusions on what determines performance but serves as an important record of these efforts, of the failure to make reliable forecasts (especially compared to death forecasts), and of performance by model, geography and time.

I was wondering if one of the main conclusions (forecasts performed worst in periods of rapid changes in reported cases, i.e. high absolute value of the reproduction number) is really borne out in the results. This statement is supported with two pieces of evidence: a decline in PI average and a greater marginal mean WIS compared to the baseline model. On the first (PI), there does indeed seem to be the relationship described but it is a bit hard to match on e.g. plots 1A and 6D when exactly coverage declines and which periods this correlates with. Perhaps this could be shown a bit clearer in a single plot, or more formally explored?

**Thank you for the suggestion to make the 95% prediction interval coverage clearer. We agree that this would strengthen the paper and have added Figure 7C to the results. This figure shows each team’s mean 95% prediction interval coverage per epidemic phase, which are referenced in the Results as follows (291-292):**

**“Likewise, 95% prediction interval coverage was highest in the nadir phase for nearly all teams while coverage in other phases was mostly lower than 95%. LNQ-ens1 and the COVIDhub ensemble had better WIS values than the baseline model in all epidemic phases between August 1, 2020 and January 15, 2022, with LNQ-ens1 also exhibiting close to nominal coverage across all phases.”**

**We also added Figure 8, which shows the direction of the prediction (increasing, decreasing, stable/uncertain) in epidemic phases. We think this provides some additional detail on how forecasts are missing in each phase.**

**Figure 7.C**

For the greater marginal mean WIS compared to the baseline model, this is based on reproduction numbers that are used to define increasing/decreasing/turning phases. The turning phases should have a reproduction number around 0, in which case I'd expect the baseline model to perform better than in the other phases. Is what we are seeing in the changes in marginal mean WIS compared to the baseline really the failure of the models to make good forecasts or is it the improved performance of the baseline? Perhaps it would be worth looking at how the marginal mean WIS values overall perform by phase, not just compared to the baseline?

**The baseline model’s predicted flat trajectory does give relatively strong performance at peaks as seen in Figure 7B. However, we scaled the phase-specific marginal mean WIS values to the average baseline marginal mean WIS across all phases. This means that while the performance is standardized relative to the baseline, relative performance between models or within models is not tied to the phase-specific baseline model performance This is noted on lines 641-642, which we clarified: “…was less than the average baseline model across all phases”.**

The code repository needs some documentation. Also some of the code points to local files rendering the work non-reproducible. It would be great to fix this.

**We have 1) expanded the repository to include code for downloading data from a cloned branch of the US COVID-19 Forecast Hub GitHub repository, 2) added truth data used for the evaluation, 3) added Rt data pulled from** [**https://github.com/epiforecasts/covid-rt-estimates**](https://github.com/epiforecasts/covid-rt-estimates)**, and 4) updated the ReadMe to describe the repository organization. These changes allow a user to re-run the presented analyses.**

** Specific comments

l.114 and 118: I did not see how reference (11) shows that county-level case forecasts were used to inform vaccine trial selection (it very vaguely references both vaccine trial site selection and vaccine distribution but without spatial scale or evidence/description of what exactly happened), or how (13) shows that incorrect forecasts lead to erosion of trust. Can you be more specific in what aspects of these papers you are referring to here?

**There are specific parts of each of these papers that refer to the use of forecasts, but as you noted they are not the focus of the papers nor are they well documented. We decided to remove this sentence and refer only to the levels of interest and potential utility.**

l.140 combines

**Thank you- this typo has been fixed.**

l.172: "Three assumed that social distancing...changed during the prediction period." - can you be more specific on what distinguished these three models from the others?

**While an interesting addition, this level of detail is beyond the scope of the analysis. We have added which three teams included social distancing in their models within the text, however, so that the reader can search the Supporting Information Table 1.1 for details.**

l.238-240: performance of the baseline - similar to the reasoning above, are these jurisdictions where the trajectory was flatter? Are you really seeing varying quality of forecasts here or just variation in the shape of the epidemics?

**This is an insightful question and one that is challenging to answer. All states had variability between phases. We added a new figure to show the distribution for each state in addition to the supplementary figure which shows the case data and phase classifications for each state.**

**To further examine state epidemic trajectories, we added a figure to Supporting Information 4 (Supporting Information 5, Figure 5.1), which shows the proportion of weeks in each classified epidemic phase per state. We state in the Methods (lines 590-592): “The proportion of weeks classified as each epidemic phase and graphical depictions of R_t_ are provided in S5. The proportion of weeks suggests non-stable Rt trajectories in most locations and there is general concordance between R_t_ and reported cases.”**

**In the plot, Louisiana and Utah had approximately half of the weeks near R_t_ =0, whereas all other states had the majority of weeks in increasing or decreasing phases. This finding suggests non-stable epidemic trends for nearly all states. Hence, we added a reference to the plot in Results, line 279.**

**We did not find a discernable pattern between jurisdiction-specific forecast skill relative to the baseline and jurisdiction-specific dynamics. We added some additional text to the Discussion in an attempt to clarify the relationship to the baseline forecasts (lines 352-362):**

**“Second, and likely related to the challenge of cases being an early indicator, the models had substantial variation in skill between epidemic phases and between states. The baseline model performed relatively well in times of peaks and worse in nadirs because of relatively high uncertainty and in increasing and decreasing phases because it forecasted a flat trend. Comparing relative skill of other forecasts in different phases with the mean baseline performance across all phases, we found that forecast skill was worst for the increasing phase followed by the decreasing phase for most teams. Classifying forecasted trajectories based on at least a 75% chance of increase or decrease, we found that a minority of forecasts for increasing and decreasing epidemic phases had confident predictions of the trajectory and forecasts for peaks and nadirs were more likely to be increasing and decreasing forecasts, respectively. Teams generally underpredicted cases in the increasing phase and overpredicted in the decreasing phase. Underprediction also carried into the peak forecasts for most teams, although several teams overpredicted peaks. In many of the periods with high WIS (e.g., the 2020-2021 winter, Delta, and Omicron waves), the COVIDhub ensemble and other teams predicted possible or probable increases or decreases, but not at the rate that occurred.”**

**Supporting Information 5, Figure 5.1**

l.248-249: seems to mix up rWIS and WIS, citing behaviour in the second as an example for behaviour int he first

**Thank you for noting this inconsistency. We modified the sentence to remove this comparison. We used the rWIS for the spatial comparison (to remove the impact of state-specific scale differences) and the WIS for the temporal comparison to more directly show the variation over time on the raw WIS values.**

l.252: "both metrics": which ones?

**We’ve edited the text to clarify that we’re referring to WIS and coverage here.**

l.375: "forecasts of deaths have generally been more reliable": can you be more specific on how you quantify this?

**Thank you for the suggestion. We have updated to add more detail here (lines 412-414): “... forecasts of deaths generally showed better forecast skill (e.g., most team death forecasts outperformed the baseline, ensemble rWIS was lower, and ensemble prediction coverage was higher)”**

l.399 "the e open"

**Thank you- this typo has been fixed.**

l.419 "sable"

**Thank you- this typo has been fixed.**

l.468-474 Inclusion criteria: can you give a rationale for these?

**Reviewer #2 posed the same question.** **We added the following text to lines 521-522: “Application of these inclusion criteria provided a more comparable set of forecasts for scoring with the attempt to reduce biased scores if teams only forecasted for a handful of locations or a limited number of weeks.”**

l.516 "We excluded...because they were not forecasted by most sets of team forecasts" is a strange sentence (do forecasts forecast? is forecasted a word?); also, can you give numbers?

**These counties were newly created in 2019 and split from a larger county, which is likely why most teams did not forecast them. We updated the sentence for clarity (lines 518-519): “We excluded two relatively new (2019) counties in Alaska (Federal Information Processing Standard code 02063 and 02066) because they were not included by most teams.”**

l.530 I think you want <https://github.com/epiforecasts/covid-rt-estimates>

**Thank you- we’ve updated the repository to the correct one.**

l.539: "Thus, we classified..." Does it matter in which order these operations were applied? E.g. what did you do if you had increasing-decreasing-increasing-decreasing?

**We first classified increasing/decreasing for all time points with probability >= 0.9 and uncertain if probability < 0.9. If there were one-week changes (e.g., increasing-increasing-decreasing-increasing- increasing, or increasing-increasing-uncertain-increasing- increasing), we changed the classification to the dominant trend (‘increasing’ in the example). When the dominant trend changed (e.g., increasing-increasing-decreasing-increasing-decreasing-dec), then the middle ones were changed to ‘peak’. By classifying increasing/decreasing/uncertain first, we were able to identify differences between sustained changes in trends (separated by peaks and nadirs) and short-term changes which may have been relatively minor stochastic shifts. All the classifications are shown in S5.**

l.570: please define all variables/symbols used in the equation

**Thank you for noting this. We added definitions for the intercept and error terms, which were the only variables not defined.**

l.575: can you explain what exactly you mean by "formal inference" here and why it can't be done with rWIS values?

**In this sentence, we’re referring to a lack of statistical hypothesis test or interval-based inference to assess differences in rWIS. We’ve updated the sentence in the manuscript to reflect this limitation, which now reads, “Comparisons of rWIS, in contrast, do not allow for formal inference with statistical hypothesis testing or interval-based inference.** **”.**
